# Transcriptional Profiling of *Plasmodium falciparum* Parasites from Patients with Severe Malaria Identifies Distinct Low vs. High Parasitemic Clusters

**DOI:** 10.1371/journal.pone.0040739

**Published:** 2012-07-18

**Authors:** Danny A. Milner, Nathalie Pochet, Malkie Krupka, Chris Williams, Karl Seydel, Terrie E. Taylor, Yves Van de Peer, Aviv Regev, Dyann Wirth, Johanna P. Daily, Jill P. Mesirov

**Affiliations:** 1 The Brigham and Women’s Hospital, Department of Pathology, Boston, Massachusetts, United States of America; 2 Harvard School of Public Health, Department of Immunology and Infectious Disease, Boston, Massachusetts, United States of America; 3 The Broad Institute, Infectious Disease Initiative, Cambridge, Massachusetts, United States of America; 4 University of Malawi College of Medicine, The Blantyre Malaria Project, Blantyre, Malawi; 5 Ghent University, Department of Plant Systems Biology, Ghent, Belgium; 6 Albert Einstein College of Medicine, Department of Medicine and Department of Microbiology and Immunology, Bronx, New York, United States of America; 7 Michigan State University, Department of Internal Medicine, College of Osteopathic Medicine, East Lansing, Michigan, United States of America; Barcelona Centre for International Health Research/Hospital Clinic/IDIBAPS/University of Barcelona, Spain

## Abstract

**Background:**

In the past decade, estimates of malaria infections have dropped from 500 million to 225 million per year; likewise, mortality rates have dropped from 3 million to 791,000 per year. However, approximately 90% of these deaths continue to occur in sub-Saharan Africa, and 85% involve children less than 5 years of age. Malaria mortality in children generally results from one or more of the following clinical syndromes: severe anemia, acidosis, and cerebral malaria. Although much is known about the clinical and pathological manifestations of CM, insights into the biology of the malaria parasite, specifically transcription during this manifestation of severe infection, are lacking.

**Methods and Findings:**

We collected peripheral blood from children meeting the clinical case definition of cerebral malaria from a cohort in Malawi, examined the patients for the presence or absence of malaria retinopathy, and performed whole genome transcriptional profiling for Plasmodium falciparum using a custom designed Affymetrix array. We identified two distinct physiological states that showed highly significant association with the level of parasitemia. We compared both groups of Malawi expression profiles with our previously acquired ex vivo expression profiles of parasites derived from infected patients with mild disease; a large collection of in vitro Plasmodium falciparum life cycle gene expression profiles; and an extensively annotated compendium of expression data from *Saccharomyces cerevisiae*. The high parasitemia patient group demonstrated a unique biology with elevated expression of Hrd1, a member of endoplasmic reticulum-associated protein degradation system.

**Conclusions:**

The presence of a unique high parasitemia state may be indicative of the parasite biology of the clinically recognized hyperparasitemic severe disease syndrome.

## Introduction

Malaria infection estimates have dropped from 500 million to 225 million per year; likewise, mortality rates have dropped from 3 million to 791,000 per year [1], [2]. Approximately 90% of these deaths occur in sub-Saharan Africa, and, despite changing global incidence, 85% still involve children less than 5 years of age [1], [3]. Malaria mortality in children generally results from one or more of the following clinical syndromes: severe anemia, acidosis, and cerebral malaria (CM) [4]. As knowledge increases about the clinical and pathological manifestations of CM, insights into the biology of the malaria parasite during this manifestation of severe infection are lacking.

Transcription profiling of *Plasmodium falciparum* from *in vitro* culture results in a reproducible cascade of expression over the 48-hour life cycle that can be shortened, lengthened, or arrested by the introduction of drug treatments and various experimental conditions [5], [6], [7], [8]. However, a shift in physiology of the parasite away from glycolysis, DNA replication, and merozoite production for re-invasion has not been demonstrated *in vitro*. Our previous analyses of *ex vivo* expression profiles (i.e., mRNA extracted directly from peripheral blood) demonstrated that in low endemnicity areas with mild malaria, there is induction of cell surface proteins and three distinct physiological states compared to *in vitro,* stage-matched, cultivated isolates [9], [10]. These observations of *ex vivo* induction of cell surface proteins were also reported in freshly adapted isolates compared to long term laboratory cultivated isolates [11].

To determine if there was specific parasite biology related to severe disease we characterized the transcriptional programs of parasites derived from children with severe disease, specifically CM. We examined the *ex vivo* expression profiles of *P. falciparum* parasites from 58 children (median age 39 months IQR [29–57 months]) meeting the clinical case definition of CM either with or without signs of malaria retinopathy. A summary of the patients and their clinical parameters is provided in Table 1. We hypothesized that distinct physiological states would exist and be related to important clinical variables in children with severe malaria. Here we demonstrate distinct parasite transcriptional states derived from severe malaria patients that are associated with the level of parasitemia, an important clinical indicator. Most importantly, a subset of samples in the high parasitemia state exhibited a transcriptional profile that suggests unique biology.

**Table 1:** A summary of the parameters for the patients included in the study.

Variable		Units		Retinopaty Positive	Retinopaty Negative	P-value*
Age	(	months, med [IQR]	)	39 [28–55 ]	35 [30–78 ]	0.8575
Gender	(	% Male	)	45	53	0.4010
Weight	(	kg, med [IQR]	)	12.0 [10.9–14.0 ]	11.5 [10.8–17.0 ]	0.5704
Height	(	cm, med [IQR]	)	94 [86–105 ]	90 [79–113 ]	0.8648
Pulse	(	beats/min, med [IQR]	)	151 [134.5–179 ]	160 [147–183 ]	0.3691
Blood Pressure (systolic)	(	systolic, med [IQR]	)	95 [90.5–106 ]	99 [85–117 ]	0.9135
Respirations	(	resp/min, med [IQR]	)	51.5 [38–57 ]	44 [32–52 ]	0.0846
Survived	(	% Yes	)	80	80	0.6340
Seizures (on admission)	(	% Yes	)	8	13	0.4280
Seizures (over admission)	(	% Yes	)	36	53	0.1950
Bednet Use	(	% Yes	)	72	40	0.0330
History of Lumefantrine	(	% Yes	)	26	20	0.4800
History of Anti-malarials	(	% Yes	)	62	47	0.2470
Respiratory Distress	(	% Yes	)	73	87	0.1110
Temperature	(	°C, med [IQR]	)	39 [38.2–39.7 ]	38.9 [38.2–39.9 ]	0.8499
Parasitemia	(	×10^3^ p/uL, med [IQR]	)	86.7 [28.8–402.5 ]	61.1 [37.1–337.1 ]	0.9397
Hematocrit	(	%, med [IQR]	)	19.2 [17.2–25.6 ]	29.1 [21.8–33.9 ]	0.0023
Hemoglobin	(	g/dL, med [IQR]	)	6.6 [5.9–9.1 ]	10.1 [7.4–12.3 ]	0.0033
White Blood Count	(	×10^3^ c/uL, med [IQR]	)	10.3 [7.4–16 ]	10.8 [6.9–16.5 ]	0.6269
Platelets	(	×10^3^ p/uL, med [IQR]	)	63 [40–99 ]	223 [29–301 ]	0.0758
Glucose	(	mg/dL, med [IQR]	)	6.3 [4.4–7.7 ]	9.9 [5.2–11.8 ]	0.0147
Lactate	(	mg/dL, med [IQR]	)	5.65 [3.05–10.75 ]	6.4 [3.3–10.8 ]	0.5328
HIV status	(	% Positive	)	15	0	0.1630
Barcode (# of het calls)	(	med [IQR]	)	1 [0–2 ]	1 [0–4 ]	0.5695

## Results

### Parasite Subgroups

**Figure 1:**
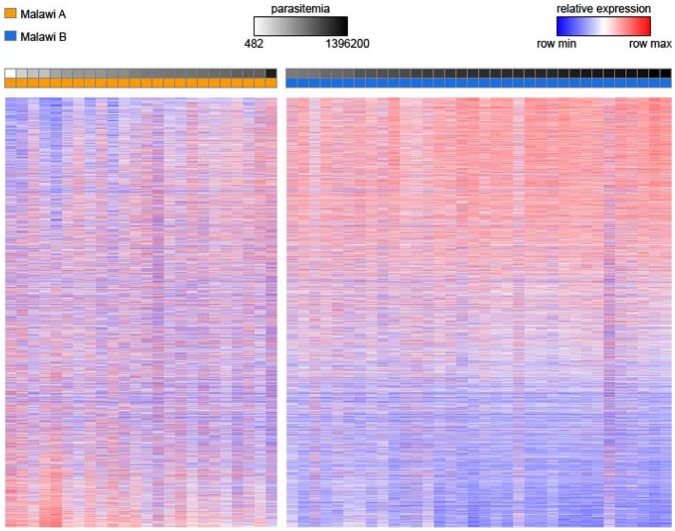
Heatmap showing expression profiles after identification of two distinct physiological states, the “low parasitemia” Cluster A (orange) and the “high parasitemia” Cluster B (blue). Samples were sorted by parasitemia within each class. Parasitemia is indicated at the top in log10 scale, ranging from low (white) to high (black). Genes are sorted by their degree of differential expression between clusters A and B.

To identify transcriptional diversity in children with severe malaria from Malawi**,** we performed an unsupervised analysis on 58 expression profiles using Non-negative matrix factorization (NMF) as previously described [9], [12]. We identified two distinct groups (Figure 1 & and Figure S1), which we defined as Clusters A and B. Cluster A contained 24 samples with more diverse or “heterogeneous” expression profiles (mean correlation = 0.811±0.072) while the 34 sample profiles in Cluster B were more highly correlated or “homogeneous” (mean correlation = 0.907±0.035) (Figure 1). Differential expression analysis between these two clusters demonstrated 1060 genes induced in Cluster A relative to Cluster B with 61 highly induced genes; similarly, 1462 genes were induced in Cluster B relative to Cluster A with 35 highly induced (Table S1).

### Clinical Variable Association

To identify host factors linked with the two parasite states, we analyzed the associated clinical and laboratory variables. We identified three host parameters that were statistically associated with Cluster A compared to Cluster B (Table 2). Cluster A parasites were significantly associated with a lower median parasitemia (29070 parasites/uL [IQR: 11300–43100]) as compared to samples in Cluster B (331100 [114700–556900]; Wilcoxon rank sum test p-value = 5.36e–008; Table S2). We also noted that when the data was regressed to account for the differences in parasitemia levels, there was a 75% overlap in the identity of the clusters, supporting our finding of parasitemia as the driver of the two cluster model (Figure S2). Cluster A patients also had statistically higher bed net use and a lower hematocrit. There were non-statistically significant associations of a higher number of gametocytes, longer duration of coma, and lower hemoglobin.

**Table 2:** Association of clinical variables to Clusters A and B.

Clinical variable	Cluster A(median+/−stdev or proportion)	Cluster B(median+/−stdev or proportion)	P-value
Parasites (#/ul)	29070+/−105794	331100+/−352244	5.36E−08
Hematocrit (admit)	19+/−5.62	25.4+/−7.45	0.04
Hemoglobin (admit)	6.3+/−2.21	8.6+/−2.60	0.08
Duration of coma (hours)	7+/−14.2	5+/−7.7	0.08
Bednet	76.2%	47.2%	0.05
Gametocytes	64.3%	34.1%	0.06

Patients in Cluster B have significantly higher levels of parasitemia and hematocrit, while a higher proportion of patients in Cluster A use bed nets.

### Correlations with Life Cycle Stages

To understand the relationship between our Malawi *ex vivo* samples and the *in vitro Plasmodium falciparum* life cycle stages, we studied the correlations between the 58 Malawi samples and three previously published life cycle data sets (Figure S3) [6], [7], [8], [13], [14]. We found that all of the Malawi samples showed strong correlation to the *in vitro* early ring stage (Spearman rank correlation of 0.68±0.06). Additionally, four transcriptomes in Cluster A demonstrated some correlation to the gametocyte stage (Spearman rank correlation of 0.41±0.05 versus 0.27±0.11 across all time points, Wilcoxon rank sum test p-value = 0.00097 at last time point). This may represent rings committed to a gametocyte program, dysynchronous cycles, or possibly contamination of the published datasets with late stage trophozoites.

### Parasitemia Driven Underlying Physiology

**Figure 2:**
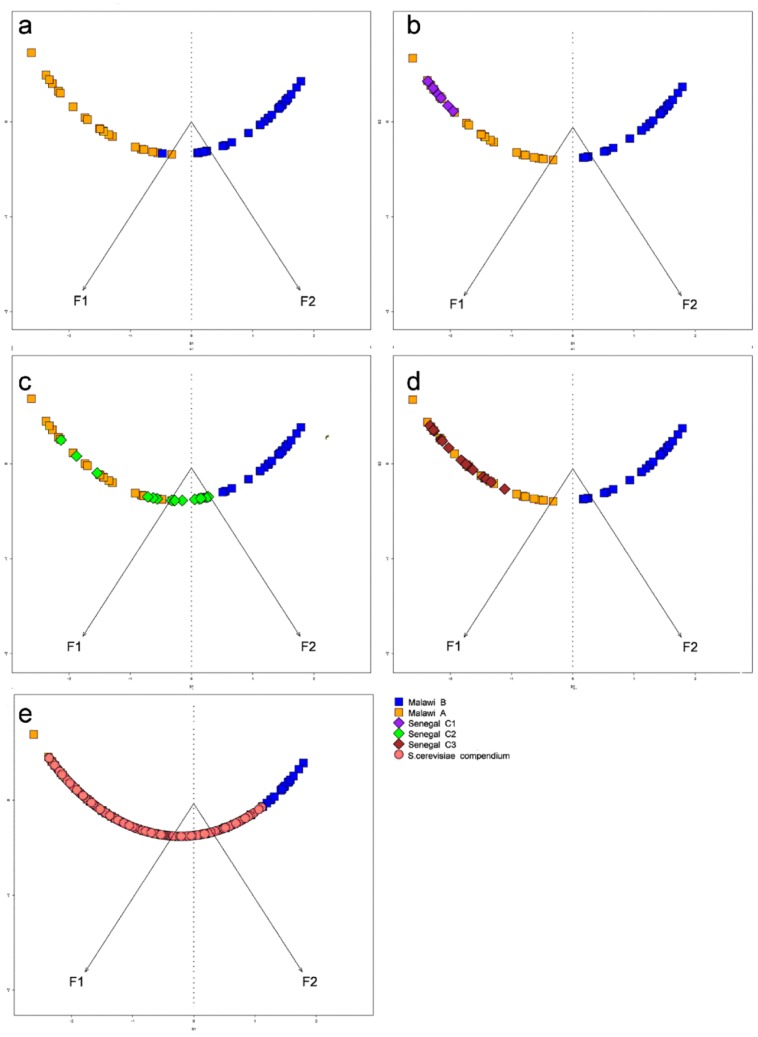
Metagene projection of 58 Malawi *ex vivo* samples, 43 Senegal *ex vivo* samples, as well as yeast and *in vitro* treated *P. falciparum* compendia onto the two-cluster Malawi model. (a) Metagene projection of 58 Malawi samples onto the two-cluster Malawi model. F1 and F2 represent the two metagene axes with F1 corresponding to the “low parasitemia” Cluster A (orange) and F2 corresponding to the “high parasitemia” Cluster B (blue). (b-d) Metagene projection of 43 Senegal samples (from Daily *et al*.) by cluster designation. Senegal Clusters 1 and 3 project onto the “low parasitemia” Malawi Cluster A while Senegal Cluster 2 projects either on Cluster A or at the transition between Clusters A and B. (e) Metagene projection of 1,439 yeast expression profiles onto the Malawi space. Enrichments of yeast experiments in Clusters A and B are consistent with projections of Senegal samples. There is a distinct space in Cluster B that is not covered by any yeast experiments, thus representing novel biology in the high parasitemia Malawi samples.

To investigate the relation between our 58 samples derived from the severe disease cohort in Malawi and our previous 43 samples from the mild disease cohort in Senegal, we compared these samples’ *ex vivo* expression profiles (Figure S4). The Senegal analysis identified three predominant parasite transcriptional clusters, each associated with a yeast phenotype: C1 ‘starvation’, C2 ‘fermentation’, and C3 ‘stress response’. To compare these profiles we used our previously described metagene projection (Figure 2a). Briefly this method allows the comparison of transcription profiles in independent experiments or across species by projection into a much lower dimensional space that captures the most distinguishing features or variability in expression (see Methods) [9], [12]. Projection of the 3 Senegal Clusters C1 (Figure 2b), C2 (Figure 2c), and C3 (Figure 2d), revealed that these samples predominantly mapped to the low parasitemia Cluster A space. This is consistent with the fact that the Senegal samples are characterized by low parasitemia [5.5%±6.2%]. Specifically, we observed that samples from Senegal Cluster C1, Cluster C2 and Cluster C3 only map to Malawi Cluster A, while some of Senegal Cluster 2 (fermentation) samples also project to a transition region between Clusters A and B. The uncovered Malawi samples from Cluster B, associated with high parasitemia, may reflect a novel biological state.

To explore the physiological basis of the Malawi transcriptional states, we compared the 58 Malawi transcriptomes to our compendium of 1,439 published expression profiles from the yeast *Saccharomyces cerevisiae* using the same approach. Projection of the yeast expression profiles revealed that these libraries primarily map to Cluster A with a small portion mapping onto Cluster B (Figure 2e). Strikingly, none of the yeast profiles map to over half the samples in Cluster B, supporting our hypothesis of unique biology.

To then explore yeast biology associated with each cluster, we used the manual and automated (gene set-based) annotations of the yeast samples to determine which conditions are enriched in each cluster [9]. Enriched in the Cluster A are stress, starvation, amino acid and nitrogen starvation, respiration, and limiting growth (Table 3). This is consistent with Senegal Clusters C1 and C3 projecting on Malawi Cluster A. Moreover, we observed a significantly higher number of samples with late stage circulating gametocytes (as visualized on peripheral blood smear) in Cluster A (64% vs. 34%, Fisher’s exact test p-value = 0.06) compared to Cluster B. Enriched in Cluster B are fermentation, perturbation in protein biosynthesis, and knock-outs on Yeast Peptone Dextrose (YPD) medium within those samples covered by the yeast projection (Table 3). This is consistent with a subset of Senegal Cluster C2 samples projecting onto the transition region between Clusters A and B.

**Table 3:** Enrichment of general categories derived from metagene projections of 1,439 yeast expression profiles onto the two-cluster Malawi model.

Set	Enriched Set	Pvalue	Set Hits	Set Size	Set Hits (%)	Total Hits	Total Size	Total Hits (%)
Stress	A	8.42E−11	578	607	95.23	1285	1439	89.3
AA and nitrogen	A	3.98E−05	126	128	98.44	1285	1439	89.3
Starvation	A	7.13E−04	167	174	95.98	1285	1439	89.3
Respiration	A	8.02E−04	213	224	95.09	1285	1439	89.3
Limiting growth	A	0.00154847	56	56	100	1285	1439	89.3
Fermentative (Happy growing yeast)	B	7.04E−07	71	411	17.28	154	1439	10.71
Perturbation in protein biosynthesis	B	1.88E−05	17	54	31.49	154	1439	10.71
Knock-outs in yeast	B	0.00165827	45	283	15.91	154	1439	10.71

Additionally, we used the manual and automated (gene set-based) annotations of the yeast samples to determine yeast conditions enriched in Clusters A and B. We observed that the yeast experiments fully cover the “low parasitemia” Cluster A and only a subset – the lower parasitemia region – of the “high parasitemia” Cluster B. Enriched in Cluster B are fermentation (happy growing yeast), perturbation in protein biosynthesis, and knock-outs in yeast peptome dextrose medium. Enriched in Cluster A are stress, starvation, amino acid and nitrogen starvation, respiration, and limiting growth.

### Pathway Analysis

**Figure 3:**
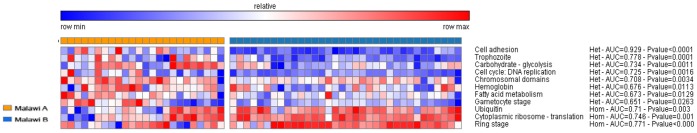
Heatmap showing the enrichment scores from single sample GSEA analysis using our general gene set categories.

To elucidate the biological processes underlying the two transcriptional states in Clusters A and B, we used our previously described Gene Set Enrichment (GSEA) approach [9] as well as a more recent reformulation of a single sample GSEA (ssGSEA) which evaluates process activation in a single sample [15] to study induction and repression of a large collection of gene sets. The original GSEA approach revealed that Cluster A showed induction of gene sets associated with cell adhesion, carbohydrate - glycolysis, cell cycle - DNA replication, and gametocyte stage (Table 4). By contrast, Cluster B showed induction of gene sets associated with ubiquitin and cytoplasmic ribosome - translation. Using ssGSEA confirmed that Cluster A showed induction of gene sets associated with cell adhesion, carbohydrate - glycolysis, cell cycle - DNA replication, chromosomal domains, hemoglobin, fatty acid metabolism, and gametocyte stage, while Cluster B showed induction of gene sets associated to cytoplasmic ribosome - translation and ubiquitin (Figure 3).

**Table 4:** Results from GSEA analysis using our general gene set categories.

Induced in Cluster A compared to Cluster B:					
GENE SETS	SIZE	ES	NES	NOMp-val	FDRq-val
CELL ADHESION	138	0.590446	2.362688	0	0
TROPHOZOITE	869	0.328596	1.603429	0	0.082741
CARBOHYDRATE - GLYCOLYSIS	178	0.363295	1.565505	0	0.06621
CELL CYCLE: DNA REPLICATION	26	0.510745	1.565032	0	0.049658
GAMETOCYTE STAGE	122	0.326327	1.309566	0.056338	0.225847
**Induced in Cluster B compared to Cluster A:**					
**GENE SETS**	**SIZE**	**ES**	**NES**	**NOM** **p-val**	**FDR** **q-val**
RING STAGE	23	−0.54059	−1.70631	0	0.01
UBIQUITIN	72	−0.38729	−1.63473	0	0.022639

### Unique Parasite Biology

We examined the biology of the parasites in the samples not covered/covered by the projection of yeast data (non-yeast/yeast space) by comparing the Malawi samples in the Cluster B non-yeast space to those in the Cluster B yeast space by studying the induction and repression of specific pathways and genes. Significantly induced in the Cluster B non-yeast space are genes associated with invasion, ring stage, gametocyte stage, and cell cycle – DNA replication. Significantly induced in the Cluster B yeast space are genes associated with carbohydrate - glycolysis, cell adhesion, protein folding, and amino acids, purines and nitrogen. Differential expression analysis at the individual gene level revealed that 631 genes were significantly induced in the Cluster B non-yeast space while 567 genes were significantly induced in the Cluster B yeast space (Table S3). The most highly induced gene in the Cluster B non-yeast space was PF14_0215, an ubiquitin ligase (Hrd1) which, in other systems, is part of the endoplasmic reticulum-associated protein degradation system.

To understand the potential effect of the difference in physiological patterns at the patient level, we compared the clinical characteristics between the Malawi samples that projected on the Cluster B non-yeast and the Cluster B yeast spaces (Table S4). We found that high parasitemia, high temperature, high hematocrit, low white blood cell count, and the absence of late stage gametocytes described the non-yeast space group relative to the yeast space group. In a multivariate logistic regression, only parasitemia (as log [parasitemia]; OR 11 [3]–[44]) and temperature (OR 4 [1.3–13]) remained significant with an AUC of 0.9576. This implies that high fever in the setting of high parasitemia is indicative of the non-yeast space and is consistent with severe malaria disease, a rare clinical pattern in our previous Senegalese data.

### Clinical Variable Pathway Analysis

We also sought to elucidate how biological processes might be associated with clinical variables (Figure S5). Serum glucose levels were positively associated with growth related gene sets and negatively related to mitochondrial sets, consistent with our previous hypothesis that host factors impact parasite biology [9]. Invasion related gene sets were positively related to platelet count and fever but negatively related to glucose, lactate, and hematocrit levels.

### Retinopathy Clinical Variable Evaluation

Abnormalities in the retina, termed malaria retinopathy, are highly associated with parasite brain sequestration and provides a robust clinical marker for CM [16], [17], [18], [19], [20], [21], [22]. Thus, we examined the relationship of retinopathy and parasite transcription to identify parasite biology associated with CM. We first looked at biological processes and found that retinopathy was associated with induction of invasion and DNA replication sets (Figure S5h). In the patient group without retinopathy, there was repression of ubiquitin pathways. We next analyzed a heat map of the 55 samples for which the retinopathy phenotype was available (40 retinopathy positive and 15 retinopathy negative) and did not observe any obvious pattern of differential expression.

To further explore the biology of parasites in retinopathy positive patients, we further examined only the 40 retinopathy samples which resulted in two clusters designated Retinopathy I and II. We compared the clinical variables between these two using Fisher’s exact test, Wilcoxon rank sum or T tests (Table S5). There were clinical variables which distinguished the retinopathy positive patients from the retinopathy negative patients, as expected, but the only clinical variable which was statistically different between Retinopathy I and II was parasitemia, consistent with the Cluster A and B patterns.

## Discussion

This is the first characterization of *ex vivo* parasite transcriptional analysis derived from patients with coma and *P. falciparum* malaria. We observed two distinct parasite transcriptional patterns, which are associated with peripheral blood parasite burden. Many of the Malawian transcriptomes and associated biology are similar to previously identified *ex vivo* transcriptomes from Senegal; however, we also identified unique transcriptomes representing unique parasite biology. Although each patient in this data set represents a single time point (a “snap shot”) during severe disease, the general observations about the pathway differences are biologically intriguing and provide insights into the host-pathogen interaction in severe disease.

NMF consensus clustering identified two transcriptional states with optimal cophenetic coefficient, Cluster A and Cluster B. To dissect the host-pathogen interaction, we analyzed clinical factors associated with both clusters and found that patients with Cluster A parasites have lower parasitemia and higher reported usage of bed nets compared to patients with Cluster B parasites. Previous studies have reported that bed net use is significantly associated with a lower mean intensity of *P. falciparum* infections [23]. In areas with high endemicity like Malawi, an older age is associated with lower parasite densities [24]. Cluster A patients mean age (47±27 months) was slightly higher than Cluster B (43±21 months), but this did not reach statistical significance (p-value = 0.282). Interestingly, the GSEA analysis revealed that Cluster A showed enrichment of cell adhesion molecules compared to Cluster B and the yeast projections suggest these Cluster A parasites were undergoing a stress response. Changes in the cell adhesion via *var* gene expression have been shown as a response to environmental stress such as nitric oxide and may suggest that the Cluster A patient environments represent an active immune response resulting in parasite stress adaptations [25]. Furthermore, the GSEA analysis reveals that the “high parasitemia” Cluster B showed enrichment of “ring stage” (a gene set described primarily by prior experiments rather than by unique ring biology) compared to Cluster A and the yeast projection reveal an association with fermentation. This suggests a parasite physiology similar to the *in vitro* biology, in that the ring stage transcriptomes were derived from cultured parasites not subject to stress, and undergoing glucose utilization and fermentative growth. Host factors mediating parasite biology may be challenging to identify and multi-factorial. Larger cohorts to test the associations identified here with full characterization of host stress attributes can further de-convolve the host parasite relationship responses and consequential biology.

Unlike the other human malarias, *P. falciparum* sequesters late stage parasites out of the peripheral blood compartment, leaving only ring stages. Our stage-specific life cycle correlations reflect this phenomenon with the vast majority showing highest correlation to *in vitro* ring stage parasites. A few of the samples in Cluster A where more gametocytes were seen by microscopy showed some correlation with *in vitro* gametocyte transcriptomes.

There are few other transcriptional analyses derived directly from naturally infected patient parasite samples. Thus, we wanted to determine if there were shared transcriptional and biological features with a dataset derived from Senegal. There are many differences between the two cohorts including marked differences in transmission intensity, with Senegal representing a low transmission site [26], [27] and Malawi a very high transmission site [28]. Geographically they are distinct–the majority of Senegalese patients who presented with mild malaria were treated as outpatients. In contrast, the present cohort required hospitalization and most patients had retinopathy-associated cerebral malaria. Despite these differences, we observed that some of the Malawian parasite transcriptomes were similar to the Senegalese parasites. Further evidence was derived through the projection onto Cluster A and a subset of Cluster B with yeast experiments enriched in Senegal Clusters C1 and C3 that were associated to stress, amino acid and nitrogen use, starvation, and respiration.

We observed, however, a number of transcriptomes in Cluster B that were not covered by the prior Senegalese samples or by the yeast experiments. This may suggest that additional unique parasite biological states exist in nature and further sampling will be needed to define the full spectrum of parasite transcriptional and biological diversity. These novel transcriptomes were associated with invasion and cell cycle – DNA replication, which could reflect parasites with a higher capacity to invade and cycle; in fact, the hosts of these parasites had significantly higher parasite loads (Table S4). Further studies would be needed to test the hypothesis that changes in parasite densities can impact parasite biology.

The most highly induced gene in the Cluster B non-yeast space as compared to the Cluster B yeast space was *P. falciparum* Hrd1 (PfHrd1, PF14_0215), which previously has been identified as a putative PfHrd1 ubiquitin ligase using bioinformatics approaches [29]. Hrd1, in other systems, is part of the endoplasmic reticulum-associated protein degradation (ERAD) complex and thus involved in retrotranslocation and degradation of proteins from the endoplasmic reticulum, a process conserved in all eukaryotes [30], [31]. Several studies have shown induction of Hrd1 to be an important response to the accumulation of unfolded or mutated proteins during endoplasmic reticulum (ER) stress, thereby protecting cells against ER stress-induced apoptosis [32], [33], [34], [35]. Although PfHrd1 has yet not been proven to have the same function, it is possible that the induction of PfHrd1 seen in parasites from patients who sustain a significantly higher temperature may reflect an adaptive stress response to be able to survive.

When children present with malaria and coma, it is critical to identify the underlying cause to provide correct treatment. The presence of malaria retinopathy signifies that parasites are sequestered in the brain and has been shown to be specific for cerebral malaria [36]. Children who have malaria and coma without retinopathy may require alternative therapies to resolve their coma. We sought to identify parasite biology associated with retinopathy positive cerebral malaria and thus divided the transcriptional analysis into two phenotypes: retinopathy positive (true CM) and retinopathy negative (those with parasitemia and coma of other causes). GSEA identified invasion, cell cycle, adhesion GO sets with retinopathy while the retinopathy negative GSEA GO reflect *in vitro* grown parasite biology including ring stage and growth. These studies can only suggest associations, and by the nature of descriptive studies, confounding factors may exist, but these data suggest that alternative non-*in vitro* biology may be involved in changes resulting in parasite tissue sequestration.

Our study provides further characterization of parasite *in vivo* biology. We also identify parasite physiology previously described from Senegalese transcriptomes. Our cohort is unique in that we are able to discriminate children with retinopathy confirmed CM, which are associated with distinctive parasite biology compared to samples derived from children without CM. Further investigation of the role of this biology may provide insight into parasite mediators of brain sequestration. A subset of samples demonstrates additional unique states. Thus our analysis provides more insight into parasite biology during natural infection and provides a framework to characterize host-pathogen relationship in the setting of severe malarial disease.

## Methods

### Patient Population

The institutional review boards of the University of Malawi College of Medicine, Albert Einstein College of Medicine, and the Brigham and Women’s Hospital approved all aspects of this study which includes informed written consent from the parents/guardians of all patients. The Malaria Research Ward (MRW) located in the Queen Elizabeth Central Hospital (Blantyre, Malawi) admits pediatric patients to an observational study of malaria pathogenesis. All patients admitted during the 2009 malaria season, from January to June, were considered for this study. Diagnostic criteria, clinical management, laboratory investigations and treatment protocols have been previously described [36]. All patients in this study met the clinical case definition of CM. The presence or absence of malarial retinopathy, defined as the presence in both eyes of vessel whitening, peripheral whitening, and/or hemorrhages, was assessed after admission using direct and indirect ophthalmoscopy and patients were classified as positive or negative for retinopathy [16], [17]. The mortality rate in this population is 15–20%; therefore, death was used as a clinical outcome.

### Patient Sampling

At admission, after informed consent, 3 mLs of peripheral blood were placed immediately in Tri reagent BD and shaken for 1 minute before being frozen at −80°C.

### Selection of Samples by Genotyping

In our previous work using a 24-SNP molecular barcode, we classified the multiplicity of infection in clinical samples by the number of heterozygous calls. Because the parasite cell cycle has an effect on the predominant expression signature seen in peripheral blood, we chose to use predominantly infections with the smallest number of heterozygous calls (0, 1, or 2) to minimize variation in the data set for expression analysis. We included a subset of samples from both retinopathy positive and retinopathy negative with a high number of heterozygous calls to determine if there was effect on expression patterns. Prior to performing peripheral blood mRNA expression, a design balanced across infection complexity by parasite genotypes was included in the study.

### RNA Extraction and Quantification

The samples were shipped from the field in liquid nitrogen, thawed at room temperature and total RNA was isolated in accordance with the manufacturer’s protocol (Tri reagent BD). Fifty-eight samples that demonstrated sharp ribosomal bands on a denaturing agarose gel stained with ethidium bromide were selected for hybridization.

### Expression Array

A novel *Plasmodium* gene expression microarray (PlasmoFBs520596) was designed in collaboration with PlasmoDB.org (David Roos) and the Broad Institute. The array was modeled after a previous multifunctional array for *Toxoplasma* [37]. The array was in the 169 format and included 2.2 million total probes with 11 probes per transcript based on the extracted annotation of *Plasmodium* species. Probes which were not unique to the *Plasmodium* genomes were excluded and additionally the sets were pruned against both human and mouse genomes. The final array contains the following elements: *P. falciparum* expression probe set, containing 5,476 unique probe sets representing 5,476 Pf genes; *P. berghei* expression probe set, containing 10,803 unique probe sets representing 10,803 Pb genes [38]; *P. falciparum* RNA gene probe set, containing 131 probe sets representing 119 Pf RNA gene sequences; *P. berghei/P. yoelli* RNA gene probe set, containing 59 probe sets representing 59 Pb and Py RNA genes (where there is considerable overlap); *P. falciparum* genotyping probes (2,377 probes); *P. falciparum* var gene probes (331 probes). The chip is commercially available from Affymetrix (Part Number: 520596, SO number: 1018048) as a wafer (168 arrays). In this analysis, only the *P. falciparum* expression probe set was analyzed and then used for comparisons with prior experiments.

### Preprocessing

Steady-state parasite mRNA levels for the 58 Malawi samples were determined with a custom-made *P. falciparum* Affymetrix chip as described above. Hybridizations were performed on two separate dates, where each batch contained two duplicate samples as control. All expression profiles were processed using RMA, implemented by the ExpressionFileCreator module in GenePattern [39], [40], [41], [42]. We set expression threshold levels from below and above to 5 and 100000, respectively, we filtered expression levels requiring a fold and delta difference of 2 and 50, respectively, and we rank normalized expression levels to correct for differences in amounts of RNA across the samples. Out of 5471 genes, 4562 genes were retained. Alternatively, to correct for differences in amounts of RNA across the samples, as we observed that the average expression in each sample is linearly correlated with parasitemia (log10 scale), we fitted a linear regression of average expression in each sample versus parasitemia (log10 scale) to correct for differences in amounts of RNA by regressing the average expression in all samples to a parasitemia level of 5 (log10 scale). In this case, all 5471 genes were retained.

All 43 previously published Senegal RNA samples were hybridized on a different custom-made *Plasmodium falciparum* Affymetrix chip [9]. Expression profiles were processed using MOID normalization as described previously [43]. We set expression threshold levels from below and above to 50 and 100000, respectively, we filtered expression levels requiring a fold and delta difference of 3 and 100, respectively, and we rank normalized expression levels.

All 1439 yeast expression profiles were taken as preprocessed in the original papers as described in our previous publication [9]. Each gene was centered and scaled to have zero mean and unit standard deviation in each data set separately. We then rank normalized expression levels.

### Non-Negative Matrix Factorization (NMF) with Consensus Clustering

To determine the optimal number of clusters, we applied NMF consensus clustering on the filtered and ranked data as previously using the Non-negativeMatrixFactorization module in GenePattern [12]. We identified the optimal number of clusters in our data based on the cophenetic coefficient. This resulted in the identification of two clusters with an optimal cophenetic coefficient of 0.9853 based on 20 clustering results and using the divergence error function.

### Metagene Projection Model

As described in our previous paper [44] and using the MetageneProjection module in GenePattern, we generated a projection map into the two distinct physiological states in our Malawi samples using NMF as above from which we calculated a projection map. The projection was followed by support vector machine (SVM) classification with a radial basis function (RBF) kernel (with refinement and post normalization, divergence error function, 2000 iterations, Brier confidence score threshold 0.30, RBF kernel parameter gamma 0.05, and regularization parameter cost 1). We used the resulting model to project several other data sets.

### Association of Clinical Variables

We estimated whether medians or proportions were significantly different between distinct physiological states using the Wilcoxon rank sum and Fisher exact tests, respectively.

### Correlations with Life Cycle Stages

We calculated Pearson linear and Spearman rank correlations between our 58 Malawi *ex vivo* samples and previously published *in vitro* life cycle data sets, including data from Llinas/DeRisi (Figure S3a) [7] and Young/LeRoch (Figure S3b) [6], [14] and (Figure S3c) [8], [13]. These data sets represent expression profiles derived from laboratory adapted strains that are grown in culture after synchronization and measured at different time points across the *in vitro Plasmodium falciparum* life cycle.

### Pathway Analysis

As previously [9], we studied the induction and repression of 755 sets from *P. falciparum* and 328 sets from *S. cerevisiae* using our approach based on gene set enrichment analysis (GSEA) [42], [45], [46]. We selected pathways that were significantly differentially expressed between distinct physiological states based on a nominal p-value smaller than 0.05 and an FDR q-value smaller than 0.25. Additionally, we used a more recent reformulation, i.e., a “single sample” extension of GSEA (ssGSEA [23]), to assess the induction and repression of these same gene sets. Here we required the nominal p-value to be smaller than 0.05 and the AUC larger than 0.65.

### Differential Gene Expression

Genes significantly differentially expressed between distinct physiological states were selected using the ComparitiveMarkerSelection module in GenePattern according to the following criteria: we required the permutation t test p-value and the FDR to be smaller than 0.05.

### Clinical Variable Pathway Analysis

For continuous clinical variables, i.e., parasitemia, platelets, glucose, lactate, temperature, white blood count, and hematocrit, we first ranked all genes by their Pearson linear correlation to that variable, and then we used an appropriate version of GSEA to determine which pathways were correlated with each variable (Figure S5a–g). For retinopathy, a binary variable, we used the standard GSEA (Figure S5h).

### Visualization of Expression Heatmaps

Expression profiles were visualized using Gene-E [47].

## Supporting Information

Figure S1 Non-negative Matrix Factorization with consensus clustering. We observed that the data partitions in two clusters with an optimal cophenetic coefficient of 0.9853 based on 20 clustering results and using the divergence error function. Cluster A contains 24 samples and Cluster B contains 34 samples. Red indicates maximum correlation and blue indicates minimum correlation as defined by the NMF result.
(PDF)

Figure S2 Heat map showing parasitemia regressed expression profiles after identification of two distinct physiological states, the “low parasitemia” Cluster A (orange) and the “high parasitemia” Cluster B (blue). Samples were sorted by parasitemia within each class. Parasitemia is indicated are the top in log10 scale, ranging from low (white).
(PDF)

Figure S3 Pearson linear (top) and Spearman (bottom) rank correlation of 58 Malawi samples with life cycle data (a) from Llinas/DeRisi measured on a two-dye platform, after creating relative measurements by centering within each data set, (b) from Llinas/DeRisi measured on a two-dye platform, after creating relative measurements taking the asexual life cycle as reference, (c) from Young/LeRoch measured on a different Affymetrix platform. Ring stage is marked in yellow, trophozoite in green, schizont in blue, merozoite in light blue, and sporozoite and sexual stage in white. to high (black). Genes are sorted by their degree of differential expression between Clusters A and B.
(PDF)

Figure S4 Pearson linear (top) and Spearman (bottom) rank correlation of 58 Malawi samples with 43 *in vivo* Senegal samples measured on a different Affymetrix platform. Senegal Cluster C1 is marked in purple, C2 in dark green, and C3 in brown.
(PDF)

Figure S5 Pathway analysis for continuous clinical variables (a) parasitemia, (b) platelets, (c) glucose, (d) lactate, (e) temperature, (f) white blood count, (g) hematocrit and for categorical variable (h) retinopathy. Genes are sorted according to their association to each of these variables.
(PDF)

Table S1 The differential expression of genes by Cluster A and B. The distribution of the mean - standard deviation was computed for expression values of genes whose differential expression for A vs. B [yellow and white genes] and B vs. A [green and gray genes] was significant (FDR ≤0.05). “Highly induced” was defined by a positive value for [(mean of high) - (std of high) ] - [(mean of low) - (std of low)] which represents a non-overlapping expression range. Genes shown in yellow are highly induced in Cluster A relative to Cluster B. Genes in gray are induced in Cluster A relative to Cluster B. Genes shown in green are highly induced in Cluster B relative to Cluster A. Genes shown in white are induced in Cluster B relative to Cluster A.
(PDF)

Table S2 The peripheral blood parasitemias for all patients in the study by final cluster designation including both the log parasitemia and raw parasitemia (p/uL).
(PDF)

Table S3 The differential expression of genes by Cluster B for those that were not covered by yeast projections versus those that were covered by yeast projections. The distribution of the mean - standard deviation was computed for expression values of genes whose differential expression for non-yeast vs. yeast [yellow and white genes) and yeast vs. non-yeast [green and gray genes] was significant (FDR ≤0.05). “Highly induced” was defined by a positive value for [(mean of high) - (std of high)] - [(mean of low) - (std of low)] which represents a non-overlapping expression range. Genes shown in yellow are highly induced in non-yeast space relative to yeast space. Genes shown in gray are induced in non-yeast relative to yeast space. Genes shown in green are highly induced in non-yeast space relative to yeast space. Genes shown in white are induced in non-yeast relative to yeast space.
(PDF)

Table S4 Clinically relevant variables compared between the Malawi samples for which there was no projection of yeast experiments (No Projection) compared with the Malawi Cluster B samples for which there is projection (Projection) demonstrates that temperature and parasitemia remain significant after correction by multivariate logistic regression.
(PDF)

Table S5 After supervised clustering of samples which only have positive signs of malaria retinopathy, two resultant clusters emerged (I and II) for which clinical variables were compared. The retinopathy negative patients are shown for reference. The p-values are for the difference between Retinopathy I and II. The variables values shown in bold text were significantly different (less than 0.05) from the retinopathy negative group.
(PDF)
