# Supplementary material for: Transcriptional Profiling of Plasmodium falciparum Parasites from Patients with Severe Malaria Identifies Distinct Low vs. High Parasitemic Clusters
Source: PLoS One. 2012 Jul 18;7(7):e40739. doi: 10.1371/journal.pone.0040739 (PMC3399889; doi:10.1371/journal.pone.0040739)

Supplemental Figure 1: Non-negative Matrix Factorization with consensus clustering. We observed that the data partitions in two clusters with an optimal cophenetic coefficient of 0.9853 based on 20 clustering results and using the divergence error function. Cluster A contains 24 samples and Cluster B contains 34 samples. Red indicates maximum correlation and blue indicates minimum correlation as defined by the NMF result.

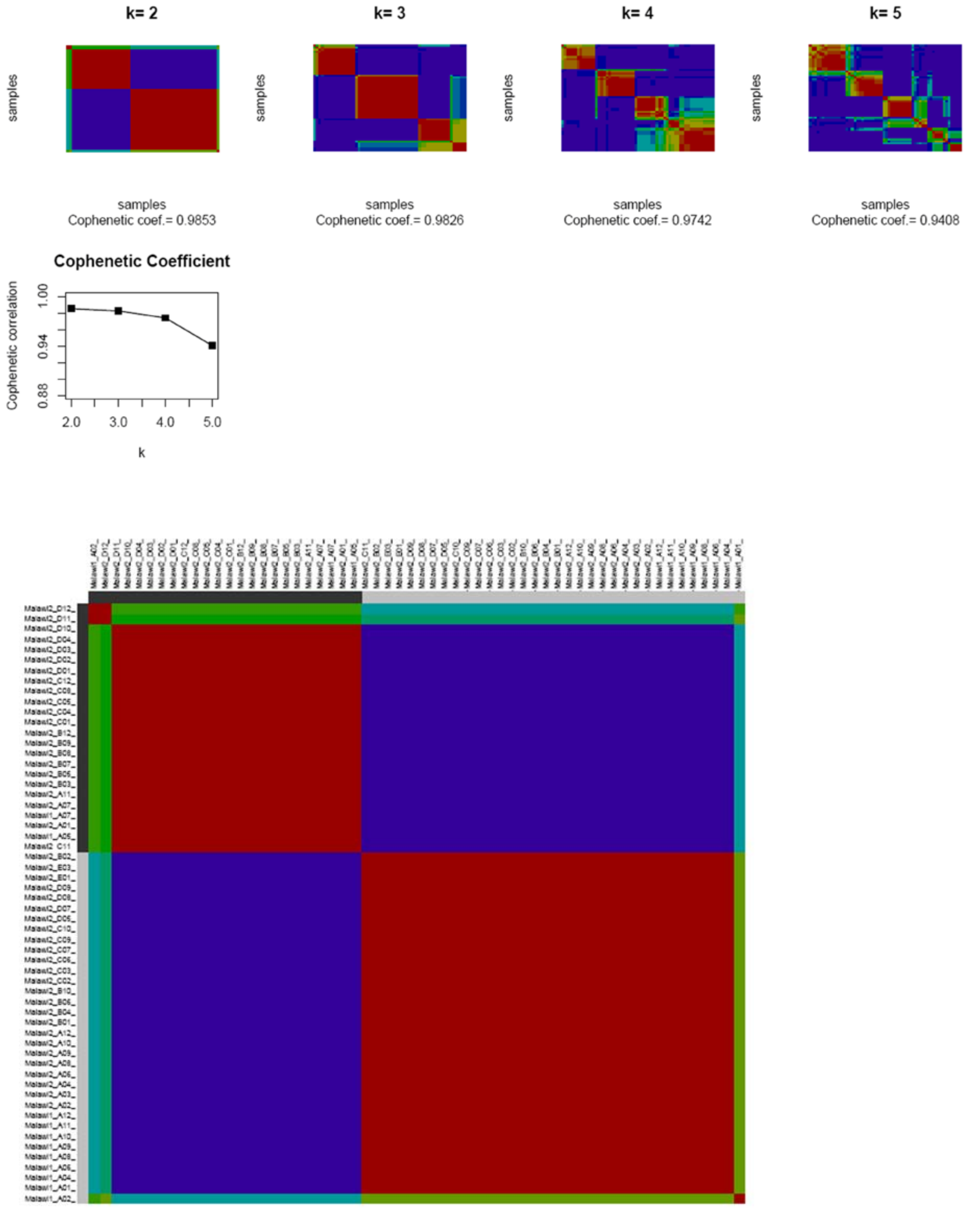

Supplement: Figure S1 — Non-negative Matrix Factorization with consensus clustering. We observed that the data partitions in two clusters with an optimal cophenetic coefficient of 0.9853 based on 20 clustering results and using the divergence error function. Cluster A contains 24 samples and Cluster B contains 34 samples. Red indicates maximum correlation and blue indicates minimum correlation as defined by the NMF result. (PDF) [file pone.0040739.s001.pdf]
