# Supplementary material for: Transcriptional Profiling of Plasmodium falciparum Parasites from Patients with Severe Malaria Identifies Distinct Low vs. High Parasitemic Clusters
Source: PLoS One. 2012 Jul 18;7(7):e40739. doi: 10.1371/journal.pone.0040739 (PMC3399889; doi:10.1371/journal.pone.0040739)

Supplemental Figure 2: Heatmap showing parasitemia regressed expression profiles after identification of two distinct physiological states, the “low parasitemia” Cluster A (orange) and the “high parasitemia” Cluster B (blue). Samples were sorted by parasitemia within each class. Parasitemia is indicated ar the top in log10 scale, ranging from low (white) to high (black). Genes are sorted by their degree of differential expression between Clusters A and B.

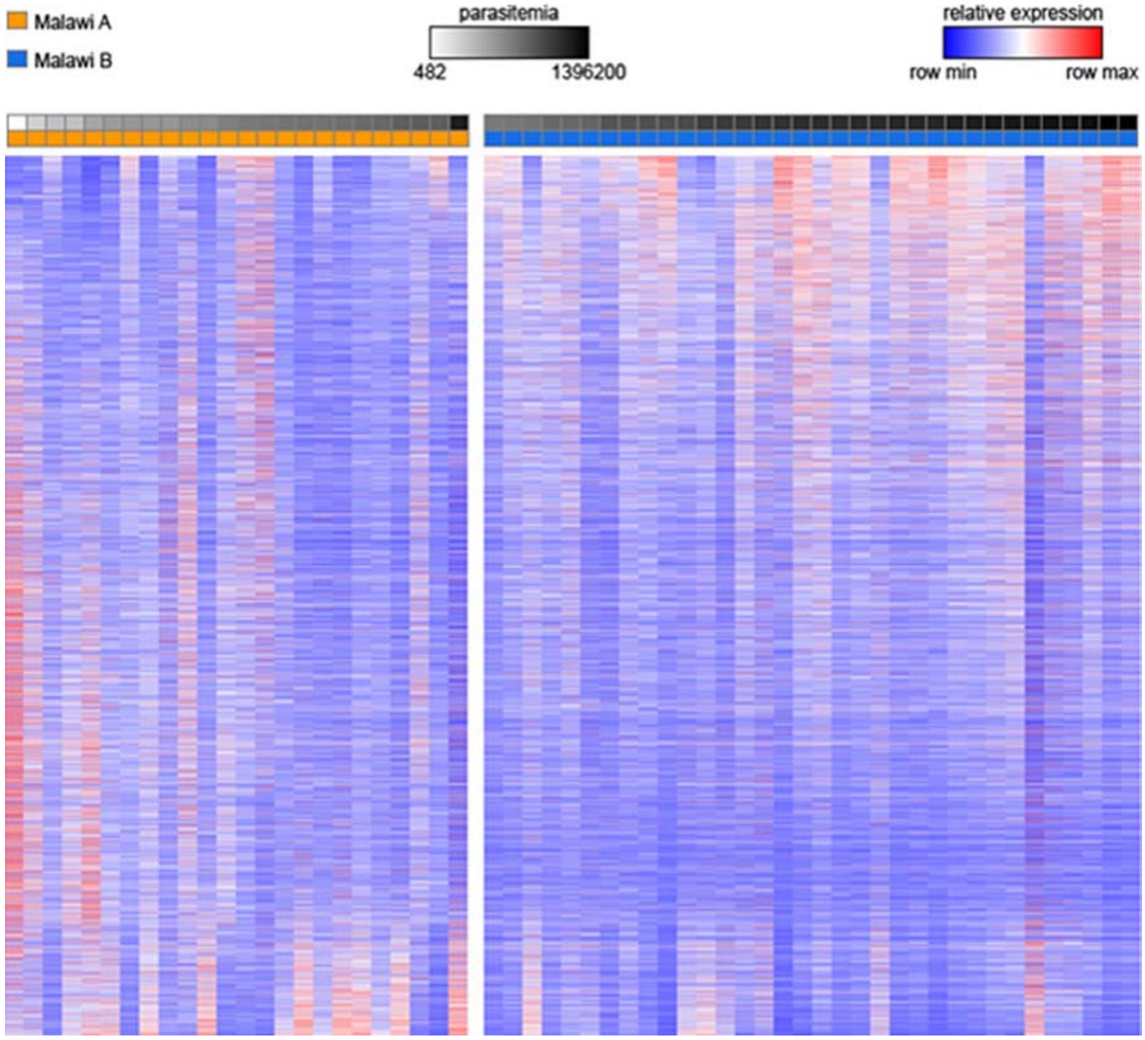

Supplement: Figure S2 — Heat map showing parasitemia regressed expression profiles after identification of two distinct physiological states, the “low parasitemia” Cluster A (orange) and the “high parasitemia” Cluster B (blue). Samples were sorted by parasitemia within each class. Parasitemia is indicated are the top in log10 scale, ranging from low (white). (PDF) [file pone.0040739.s002.pdf]
