# Supplementary material for: Transcriptional Profiling of Plasmodium falciparum Parasites from Patients with Severe Malaria Identifies Distinct Low vs. High Parasitemic Clusters
Source: PLoS One. 2012 Jul 18;7(7):e40739. doi: 10.1371/journal.pone.0040739 (PMC3399889; doi:10.1371/journal.pone.0040739)
Supplement: Figure S3 — Pearson linear (top) and Spearman (bottom) rank correlation of 58 Malawi samples with life cycle data (a) from Llinas/DeRisi measured on a two-dye platform, after creating relative measurements by centering within each data set, (b) from Llinas/DeRisi measured on a two-dye platform, after creating relative measurements taking the asexual life cycle as reference, (c) from Young/LeRoch measured on a different Affymetrix platform. Ring stage is marked in yellow, trophozoite in green, schizont in blue, merozoite in light blue, and sporozoite and sexual stage in white. to high (black). Genes are sorted by their degree of differential expression between Clusters A and B. (PDF) [file pone.0040739.s003.pdf]

Supplemental Figure 3: Pearson linear (top) and Spearman (bottom) rank correlation of 58 Malawi samples with life cycle data (a) from Llinas/DeRisi measured on a two-dye platform, after creating relative measurements by centering within each data set, (b) from Llinas/DeRisi measured on a two-dye platform, after creating relative measurements taking the asexual life cycle as reference, (c) from Young/LeRoch measured on a different Affymetrix platform. Ring stage is marked in yellow, trophozoite in green, schizont in blue, merozoite in light blue, and sporozoite and sexual stage in white.

a

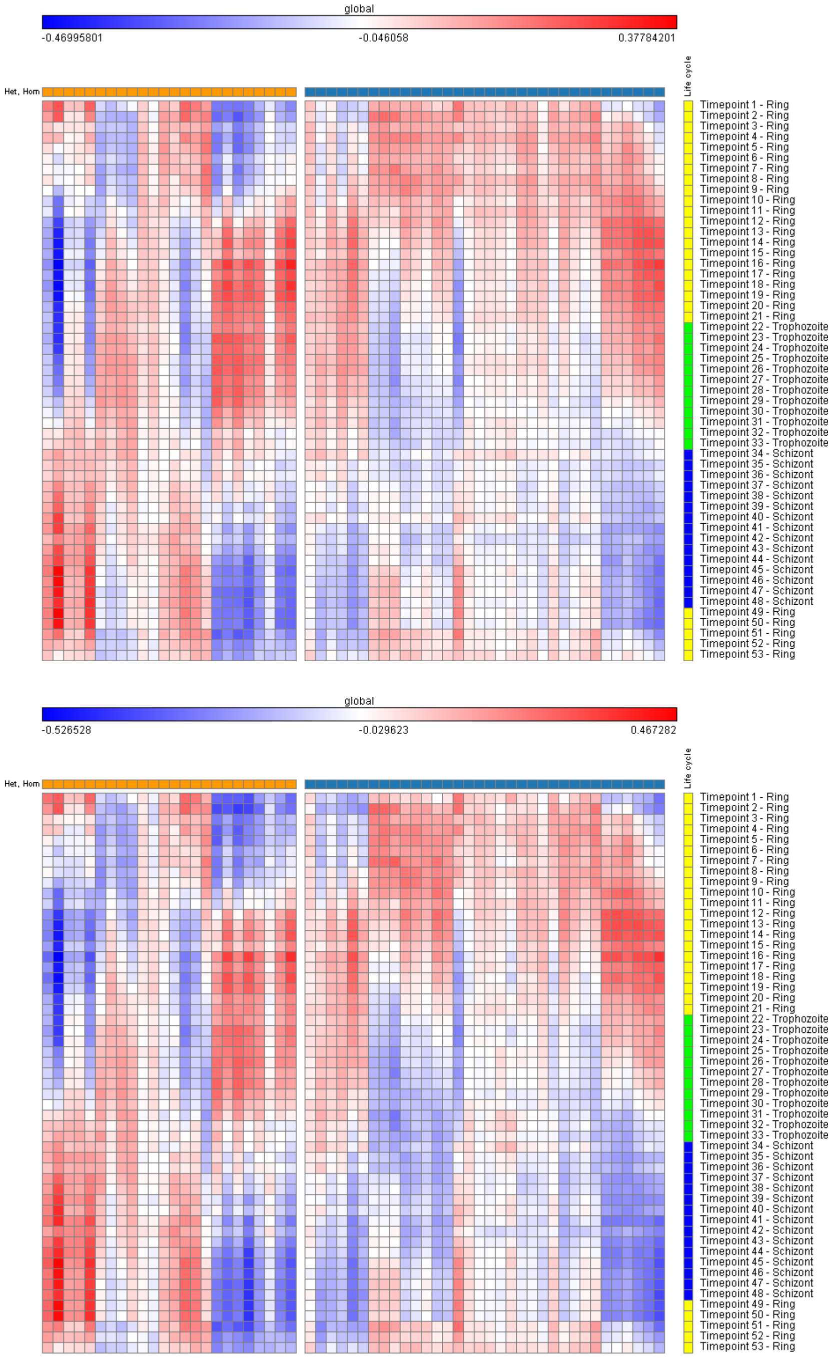

b

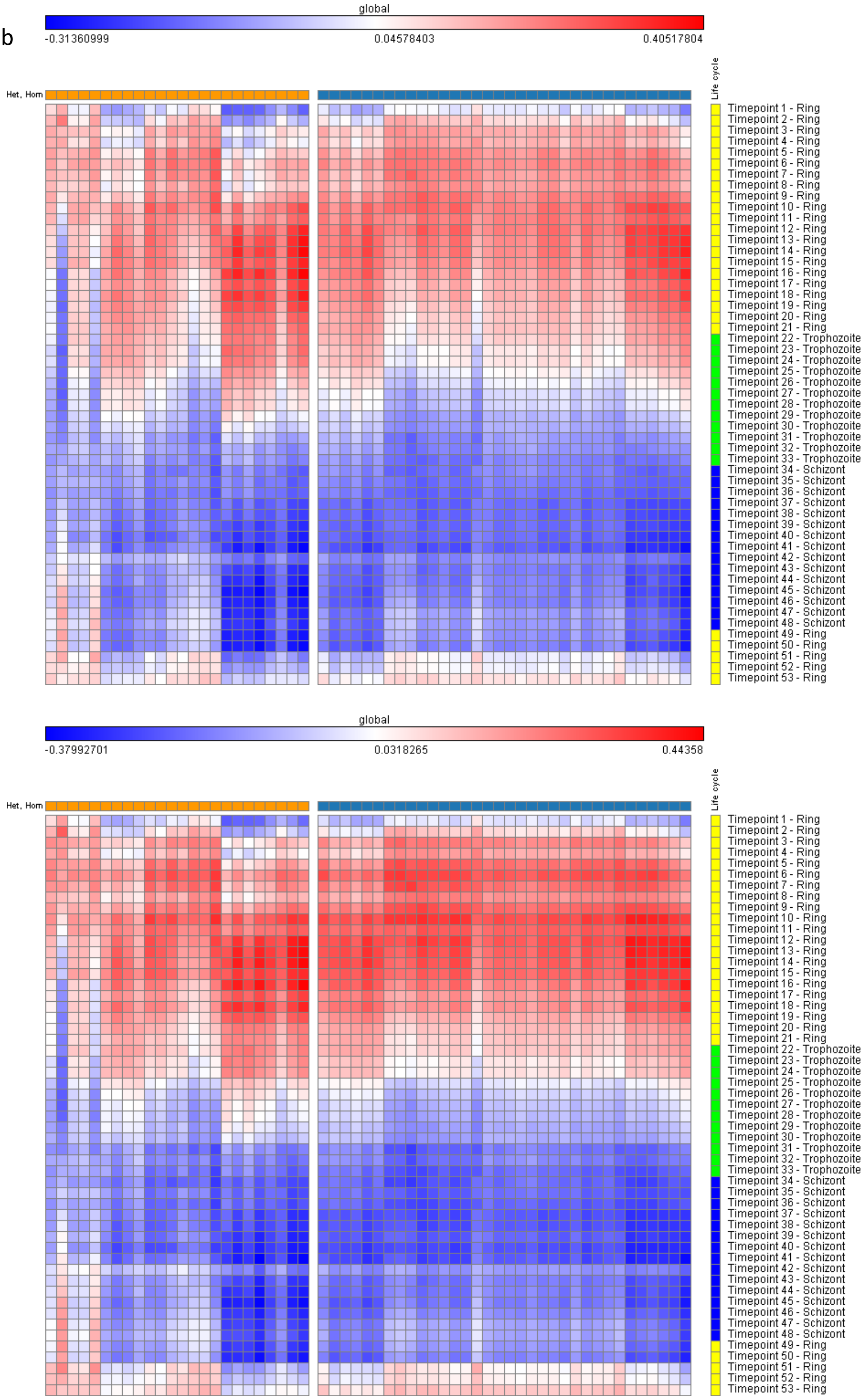

global
-0.31360999
0.04578403
0.40517804
Het, Hom
Life cycle
Timepoint 1 - Ring
Timepoint 2 - Ring
Timepoint 3 - Ring
Timepoint 4 - Ring
Timepoint 5 - Ring
Timepoint 6 - Ring
Timepoint 7 - Ring
Timepoint 8 - Ring
Timepoint 9 - Ring
Timepoint 10 - Ring
Timepoint 11 - Ring
Timepoint 12 - Ring
Timepoint 13 - Ring
Timepoint 14 - Ring
Timepoint 15 - Ring
Timepoint 16 - Ring
Timepoint 17 - Ring
Timepoint 18 - Ring
Timepoint 19 - Ring
Timepoint 20 - Ring
Timepoint 21 - Ring
Timepoint 22 - Trophozoite
Timepoint 23 - Trophozoite
Timepoint 24 - Trophozoite
Timepoint 25 - Trophozoite
Timepoint 26 - Trophozoite
Timepoint 27 - Trophozoite
Timepoint 28 - Trophozoite
Timepoint 29 - Trophozoite
Timepoint 30 - Trophozoite
Timepoint 31 - Trophozoite
Timepoint 32 - Trophozoite
Timepoint 33 - Trophozoite
Timepoint 34 - Schizont
Timepoint 35 - Schizont
Timepoint 36 - Schizont
Timepoint 37 - Schizont
Timepoint 38 - Schizont
Timepoint 39 - Schizont
Timepoint 40 - Schizont
Timepoint 41 - Schizont
Timepoint 42 - Schizont
Timepoint 43 - Schizont
Timepoint 44 - Schizont
Timepoint 45 - Schizont
Timepoint 46 - Schizont
Timepoint 47 - Schizont
Timepoint 48 - Schizont
Timepoint 49 - Ring
Timepoint 50 - Ring
Timepoint 51 - Ring
Timepoint 52 - Ring
Timepoint 53 - Ring
global
-0.37992701
0.0318265
0.44358
Het, Hom
Life cycle
Timepoint 1 - Ring
Timepoint 2 - Ring
Timepoint 3 - Ring
Timepoint 4 - Ring
Timepoint 5 - Ring
Timepoint 6 - Ring
Timepoint 7 - Ring
Timepoint 8 - Ring
Timepoint 9 - Ring
Timepoint 10 - Ring
Timepoint 11 - Ring
Timepoint 12 - Ring
Timepoint 13 - Ring
Timepoint 14 - Ring
Timepoint 15 - Ring
Timepoint 16 - Ring
Timepoint 17 - Ring
Timepoint 18 - Ring
Timepoint 19 - Ring
Timepoint 20 - Ring
Timepoint 21 - Ring
Timepoint 22 - Trophozoite
Timepoint 23 - Trophozoite
Timepoint 24 - Trophozoite
Timepoint 25 - Trophozoite
Timepoint 26 - Trophozoite
Timepoint 27 - Trophozoite
Timepoint 28 - Trophozoite
Timepoint 29 - Trophozoite
Timepoint 30 - Trophozoite
Timepoint 31 - Trophozoite
Timepoint 32 - Trophozoite
Timepoint 33 - Trophozoite
Timepoint 34 - Schizont
Timepoint 35 - Schizont
Timepoint 36 - Schizont
Timepoint 37 - Schizont
Timepoint 38 - Schizont
Timepoint 39 - Schizont
Timepoint 40 - Schizont
Timepoint 41 - Schizont
Timepoint 42 - Schizont
Timepoint 43 - Schizont
Timepoint 44 - Schizont
Timepoint 45 - Schizont
Timepoint 46 - Schizont
Timepoint 47 - Schizont
Timepoint 48 - Schizont
Timepoint 49 - Ring
Timepoint 50 - Ring
Timepoint 51 - Ring
Timepoint 52 - Ring
Timepoint 53 - Ring

C

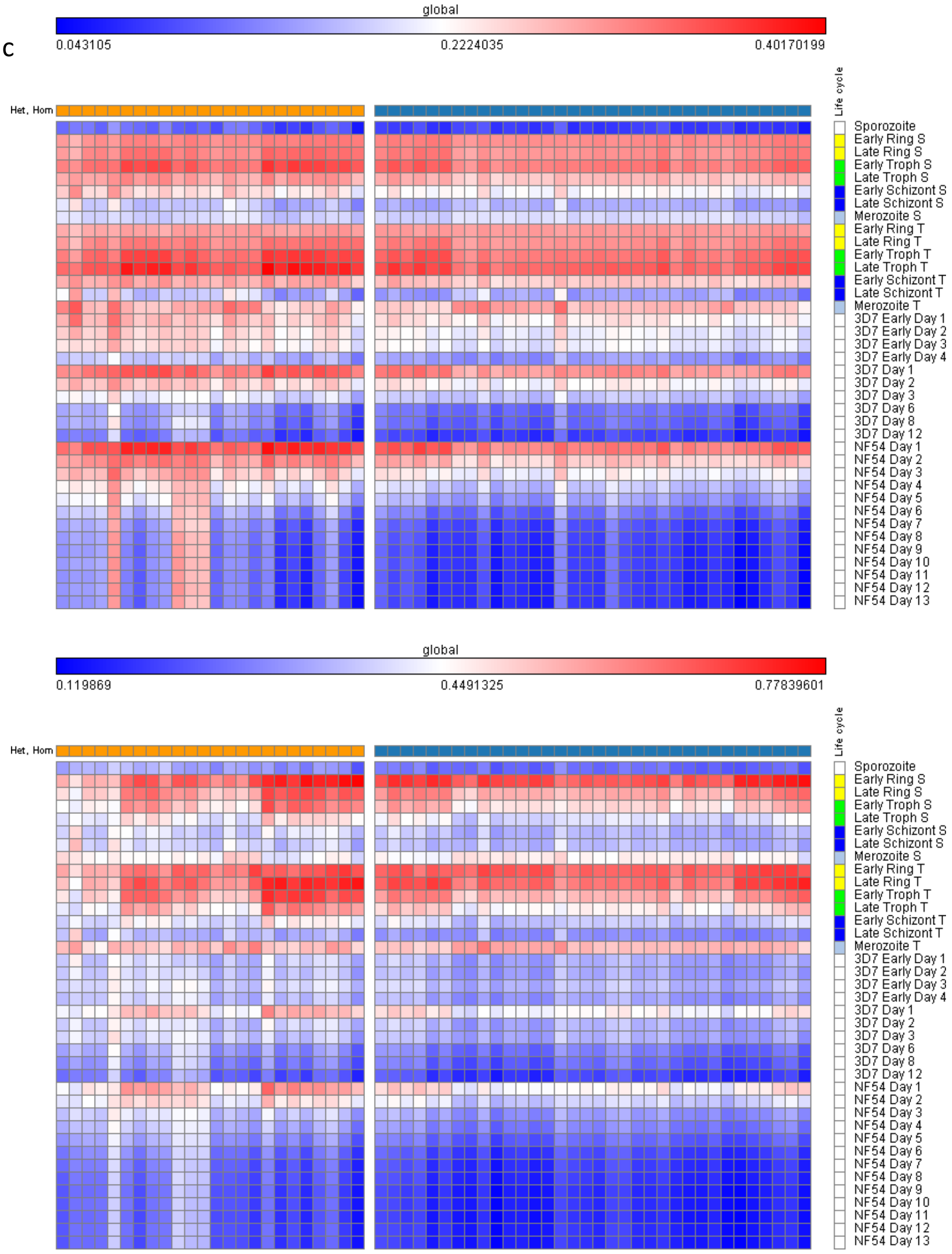
global
0.043105
0.2224035
0.40170199
Het, Hom
Life cycle
Sporozoite
Early Ring S
Late Ring S
Early Troph S
Late Troph S
Early Schizont S
Late Schizont S
Merozoite S
Early Ring T
Late Ring T
Early Troph T
Late Troph T
Early Schizont T
Late Schizont T
Merozoite T
3D7 Early Day 1
3D7 Early Day 2
3D7 Early Day 3
3D7 Early Day 4
3D7 Day 1
3D7 Day 2
3D7 Day 3
3D7 Day 6
3D7 Day 8
3D7 Day 12
NF54 Day 1
NF54 Day 2
NF54 Day 3
NF54 Day 4
NF54 Day 5
NF54 Day 6
NF54 Day 7
NF54 Day 8
NF54 Day 9
NF54 Day 10
NF54 Day 11
NF54 Day 12
NF54 Day 13
global
0.119869
0.4491325
0.77839601
Het, Hom
Life cycle
Sporozoite
Early Ring S
Late Ring S
Early Troph S
Late Troph S
Early Schizont S
Late Schizont S
Merozoite S
Early Ring T
Late Ring T
Early Troph T
Late Troph T
Early Schizont T
Late Schizont T
Merozoite T
3D7 Early Day 1
3D7 Early Day 2
3D7 Early Day 3
3D7 Early Day 4
3D7 Day 1
3D7 Day 2
3D7 Day 3
3D7 Day 6
3D7 Day 8
3D7 Day 12
NF54 Day 1
NF54 Day 2
NF54 Day 3
NF54 Day 4
NF54 Day 5
NF54 Day 6
NF54 Day 7
NF54 Day 8
NF54 Day 9
NF54 Day 10
NF54 Day 11
NF54 Day 12
NF54 Day 13
