# Supplementary material for: Transcriptional Profiling of Plasmodium falciparum Parasites from Patients with Severe Malaria Identifies Distinct Low vs. High Parasitemic Clusters
Source: PLoS One. 2012 Jul 18;7(7):e40739. doi: 10.1371/journal.pone.0040739 (PMC3399889; doi:10.1371/journal.pone.0040739)

Supplemental Figure 4: Pearson linear (top) and Spearman (bottom) rank correlation of 58 Malawi samples with 43 *in vivo* Senegal samples measured on a different Affymetrix platform. Senegal Cluster C1 is marked in purple, C2 in dark green, and C3 in brown.

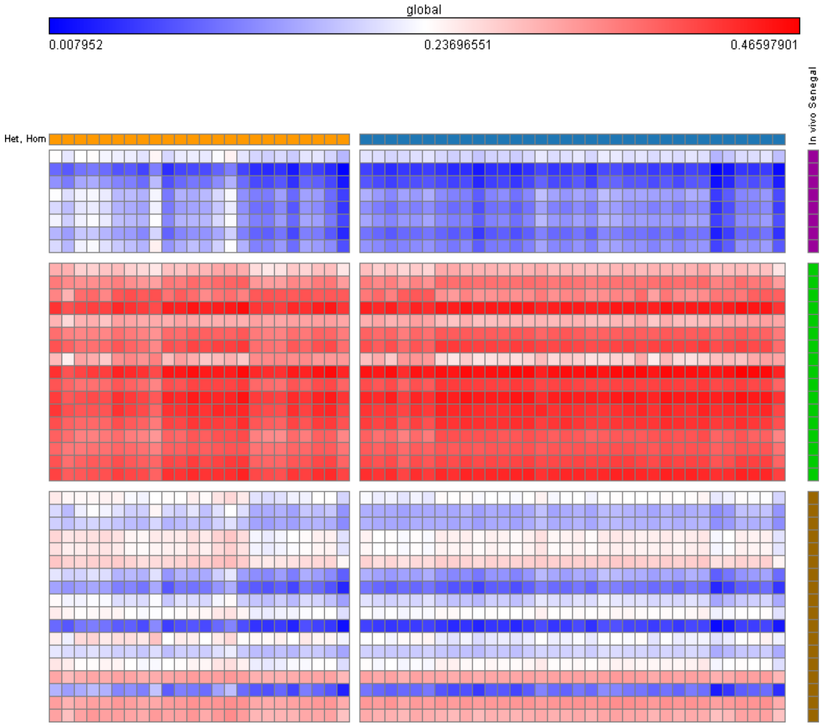

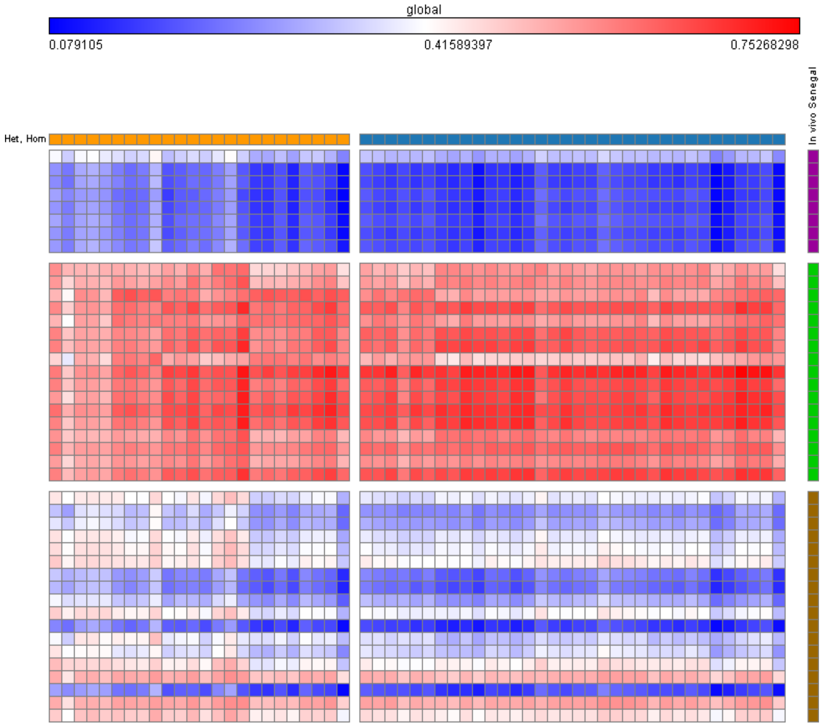

Supplement: Figure S4 — Pearson linear (top) and Spearman (bottom) rank correlation of 58 Malawi samples with 43 in vivo Senegal samples measured on a different Affymetrix platform. Senegal Cluster C1 is marked in purple, C2 in dark green, and C3 in brown. (PDF) [file pone.0040739.s004.pdf]
