# Supplementary material for: Transcriptional Profiling of Plasmodium falciparum Parasites from Patients with Severe Malaria Identifies Distinct Low vs. High Parasitemic Clusters
Source: PLoS One. 2012 Jul 18;7(7):e40739. doi: 10.1371/journal.pone.0040739 (PMC3399889; doi:10.1371/journal.pone.0040739)

Supplemental Figure 5: Pathway analysis for continuous clinical variables (a) parasitemia, (b) platelets, (c) glucose, (d) lactate, (e) temperature, (f) white blood count, (g) hematocrit and for categorical variable (h) retinopathy. Genes are sorted according to their association to each of these variables.

a

CORRELATED GENE SETS	SIZE	ES	NES	NOM p-val	FDR q-val
CYTOPLASMIC RIBOSOME - TRANSLATION	706	0.378649	2.358244	0	0
GROWTH	588	0.306781	1.882512	0	0.005545
TRANSCRIPTION	61	0.412743	1.863294	0	0.004576
RING STAGE	23	0.526222	1.846481	0.005164	0.003799
AA - PURINES - NITROGEN	173	0.278714	1.515567	0.003026	0.041351
UBIQUITIN	72	0.321361	1.475919	0.023179	0.045728

ANTI-CORRELATED GENE SETS	SIZE	ES	NES	NOM p-val	FDR q-val
CELL ADHESION	138	-0.54149	-3.04788	0	0
GAMETOCYTE STAGE	122	-0.40003	-2.17971	0	7.50E-04
CELL CYCLE: DNA REPLICATION	26	-0.43347	-1.66274	0.017857	0.037986
TROPHOZOITE	869	-0.19101	-1.30687	0.004808	0.278556

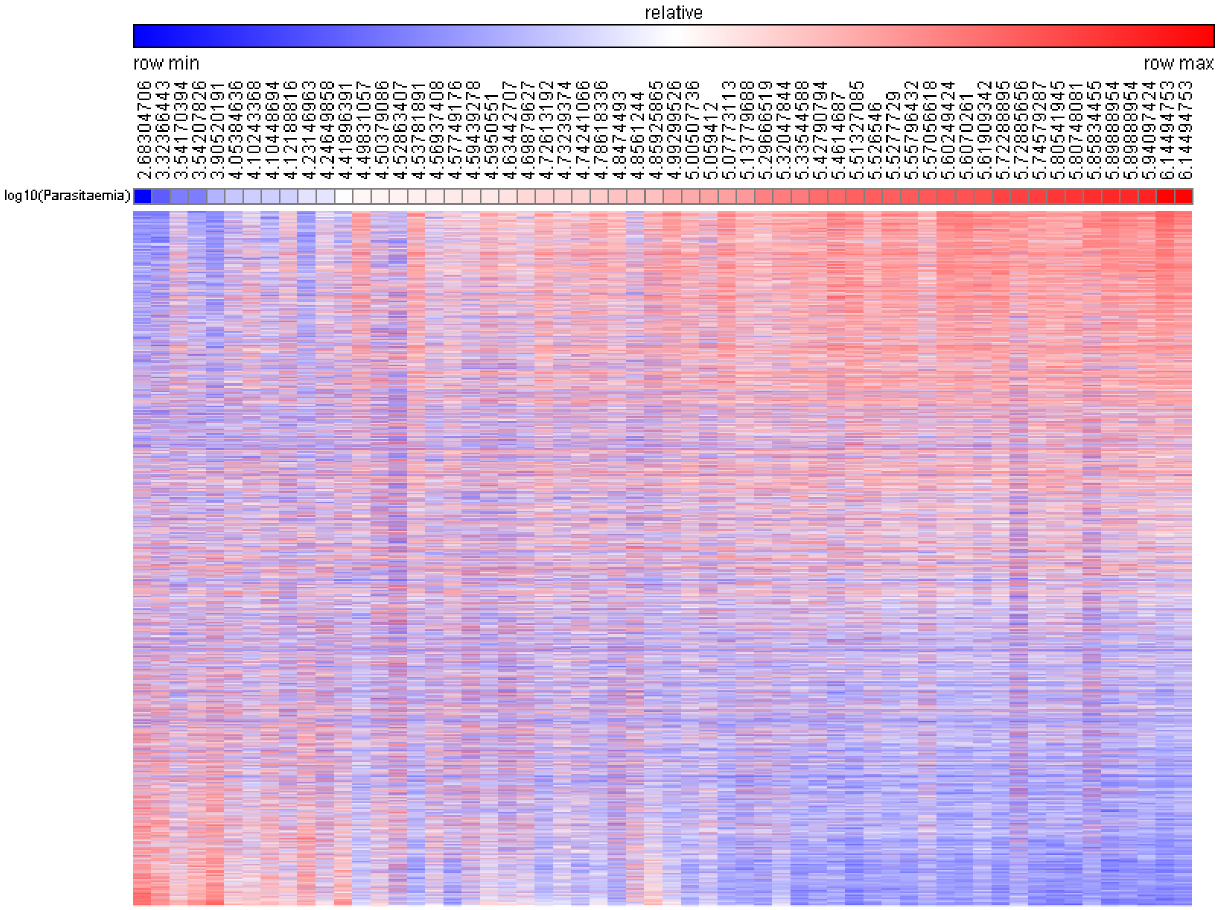

Supplemental Figure 5 (cont.)

b

CORRELATED GENE SETS	SIZE	ES	NES	NOM p-val	FDR q-val
INVASION	47	0.592969	2.232762	0	0
CELL ADHESION	138	0.354804	1.628695	0	0.063327
CELL CYCLE: MITOSIS	26	0.475189	1.576391	0.014028	0.062473
UBIQUITIN	72	0.365087	1.527464	0.011299	0.069222

ANTI-CORRELATED GENE SETS	SIZE	ES	NES	NOM p-val	FDR q-val
CYTOPLASMIC RIBOSOME - TRANSLATION	706	-0.39742	-2.25349	0	0
AA - PURINES - NITROGEN	173	-0.37002	-1.82871	0	0.004211
TRANSCRIPTION	61	-0.42944	-1.75536	0.002079	0.006548
PROTEIN FOLDING	94	-0.38121	-1.70887	0	0.008276
GROWTH	588	-0.30069	-1.66906	0	0.009205

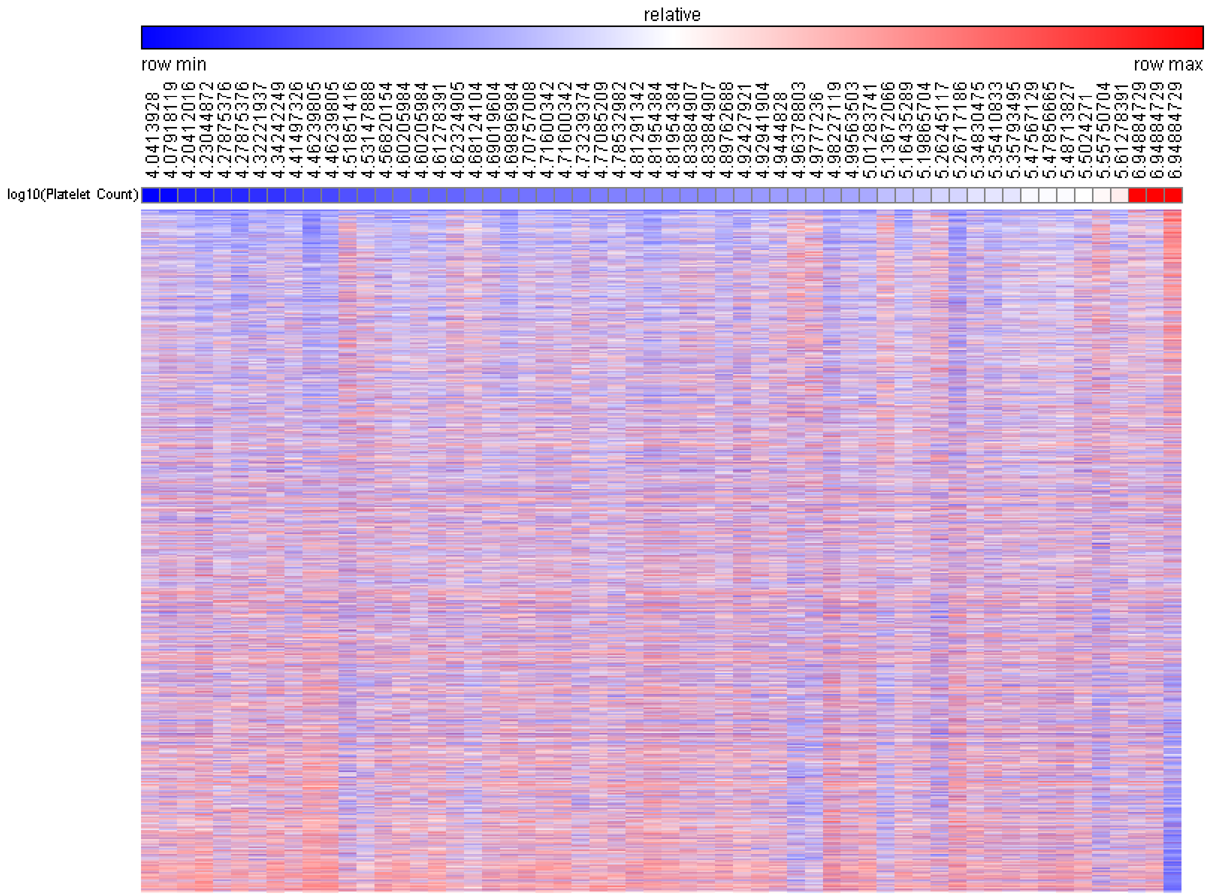

Supplemental Figure 5 (cont.)

C

**CORRELATED GENE SETS**	**SIZE**	**ES**	**NES**	**NOM p-val**	**FDR q-val**
GROWTH	588	0.33	1.94	0.000	0.006
AA - PURINES - NITROGEN	173	0.37	1.91	0.000	0.005
CYTOPLASMIC RIBOSOME - TRANSLATION	706	0.30	1.74	0.000	0.020
UBIQUITIN	72	0.38	1.65	0.004	0.028
TRANSCRIPTION	61	0.38	1.63	0.008	0.027
PROTEIN FOLDING	94	0.31	1.43	0.022	0.086
CARBOHYDRATE - GLYCOLYSIS	178	0.26	1.34	0.034	0.143

**ANTI-CORRELATED GENE SETS**	**SIZE**	**ES**	**NES**	**NOM p-val**	**FDR q-val**
INVASION	47	-0.53	-2.16	0.000	0.001
CELL CYCLE: DNA REPLICATION	26	-0.58	-2.06	0.000	0.001
MITOCHONDRIAL - PLASTID BIOGENESIS	27	-0.48	-1.72	0.013	0.013
TROPHOZOITE	869	-0.26	-1.62	0.000	0.019
GAMETOCYTE STAGE	122	-0.31	-1.51	0.006	0.037

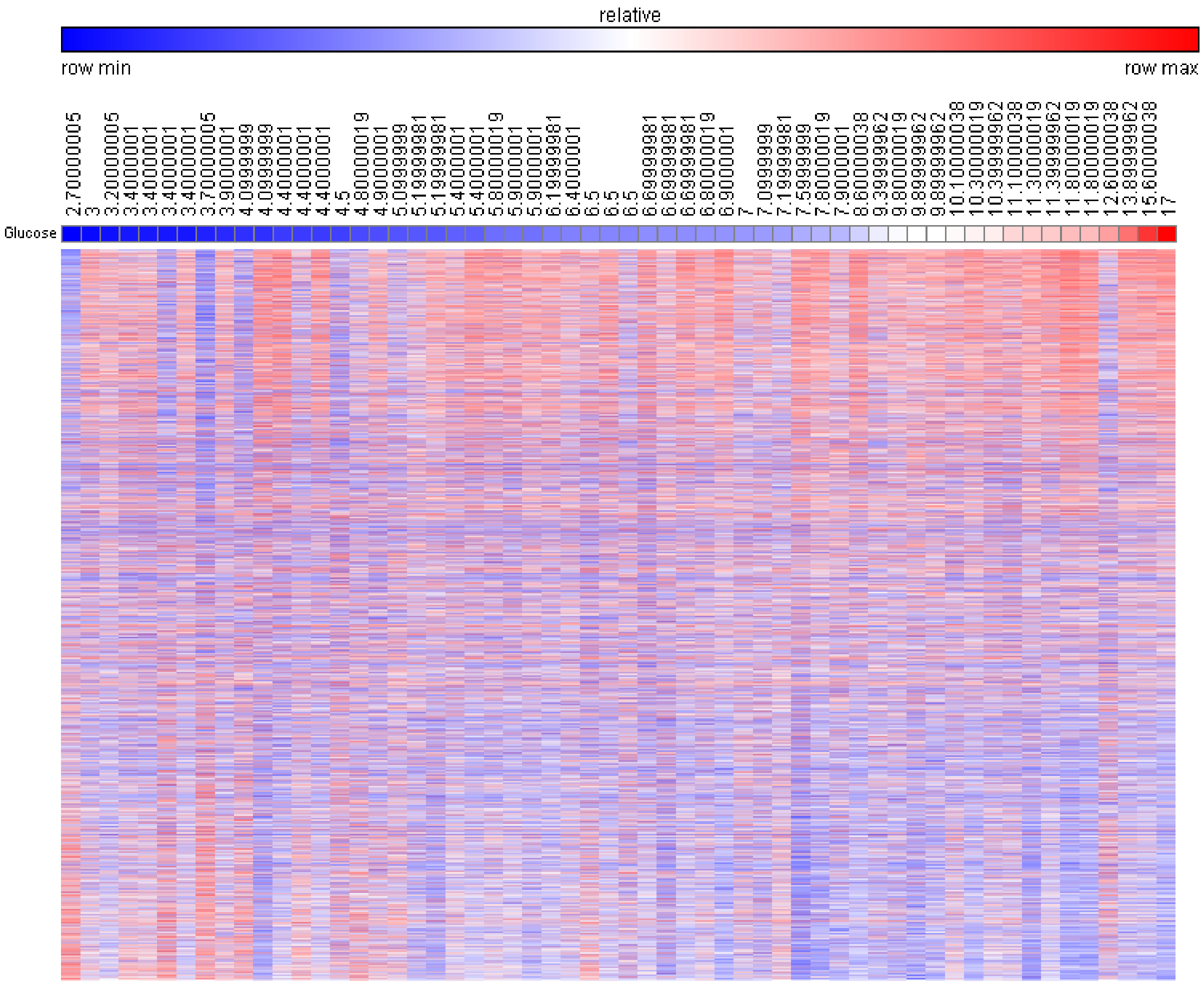

Supplemental Figure 5 (cont.)

d

CORRELATED GENE SETS	SIZE	ES	NES	NOM p-val	FDR q-val
CYTOPLASMIC RIBOSOME - TRANSLATION	706	0.42	2.36	0.000	0.000
TRANSCRIPTION	61	0.54	2.28	0.000	0.000
AA - PURINES - NITROGEN	173	0.43	2.12	0.000	0.000
GROWTH	588	0.37	2.06	0.000	0.000
PROTEIN FOLDING	94	0.37	1.67	0.003	0.016
CHROMOSOMAL DOMAINS	2900	0.21	1.28	0.000	0.205
CELL ADHESION	138	0.27	1.27	0.081	0.188

ANTI-CORRELATED GENE SETS	SIZE	ES	NES	NOM p-val	FDR q-val
INVASION	47	-0.58	-2.36	0.000	0.000
RESPIRATION - OXIDATIVE PHOSPHORYLATION	59	-0.31	-1.33	0.072	0.383

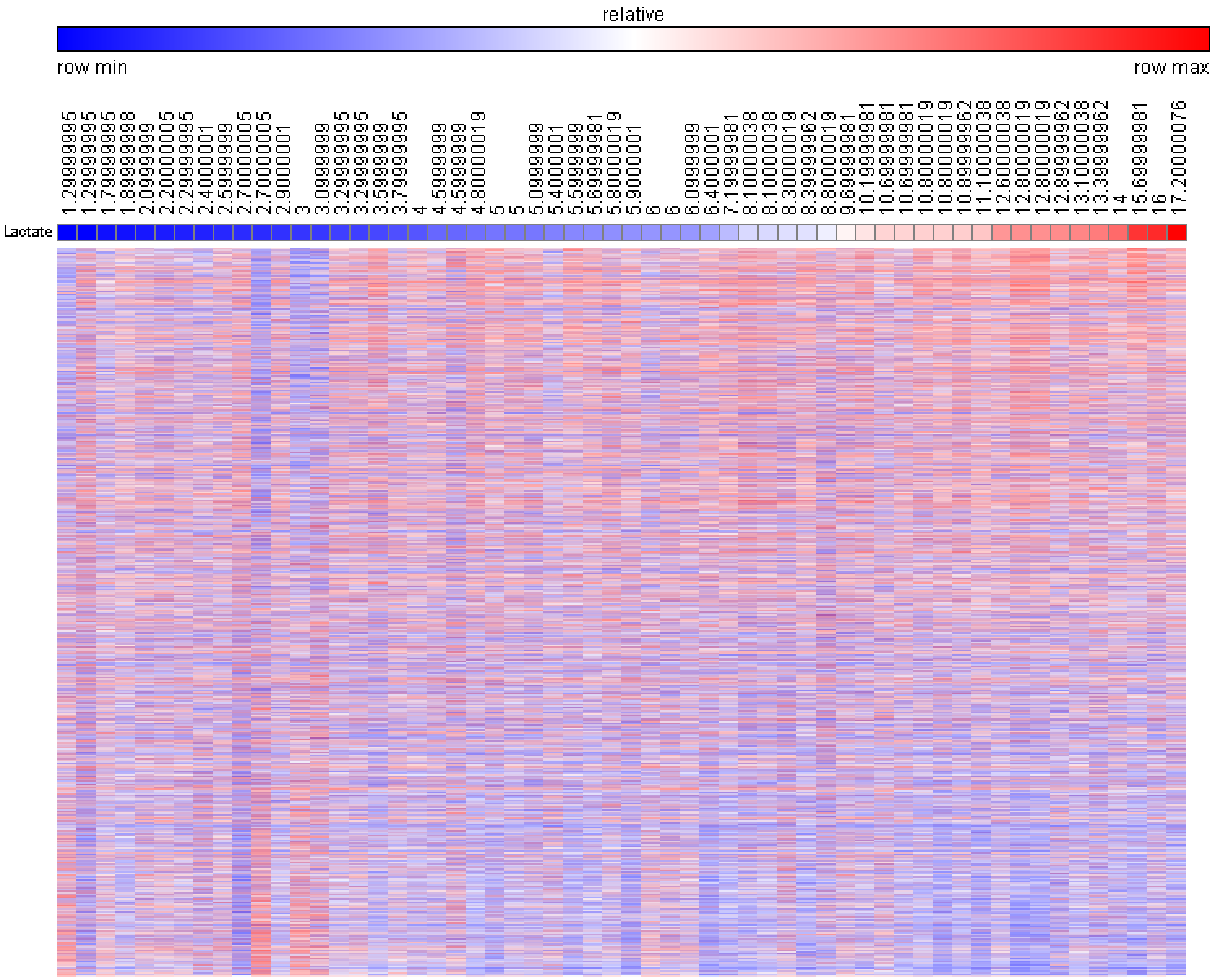

Supplemental Figure 5 (cont.)

e

CORRELATED GENE SETS	SIZE	ES	NES	NOM p-val	FDR q-val
INVASION	47	0.59	2.32	0.000	0.000
GAMETOCYTE STAGE	122	0.33	1.57	0.002	0.076
MITOCHONDRIAL - PLASTID BIOGENESIS	27	0.43	1.48	0.044	0.095
CELL CYCLE: MITOSIS	26	0.41	1.40	0.057	0.125

ANTI-CORRELATED GENE SETS	SIZE	ES	NES	NOM p-val	FDR q-val
CARBOHYDRATE - GLYCOLYSIS	178	-0.33	-1.72	0.000	0.054
GROWTH	588	-0.28	-1.65	0.000	0.038
UBIQUITIN	72	-0.37	-1.64	0.002	0.028
VACUOLE	7	-0.69	-1.62	0.028	0.024
AA - PURINES - NITROGEN	173	-0.30	-1.59	0.000	0.027
PROTEIN FOLDING	94	-0.32	-1.52	0.008	0.039
CYTOPLASMIC RIBOSOME - TRANSLATION	706	-0.24	-1.43	0.000	0.057

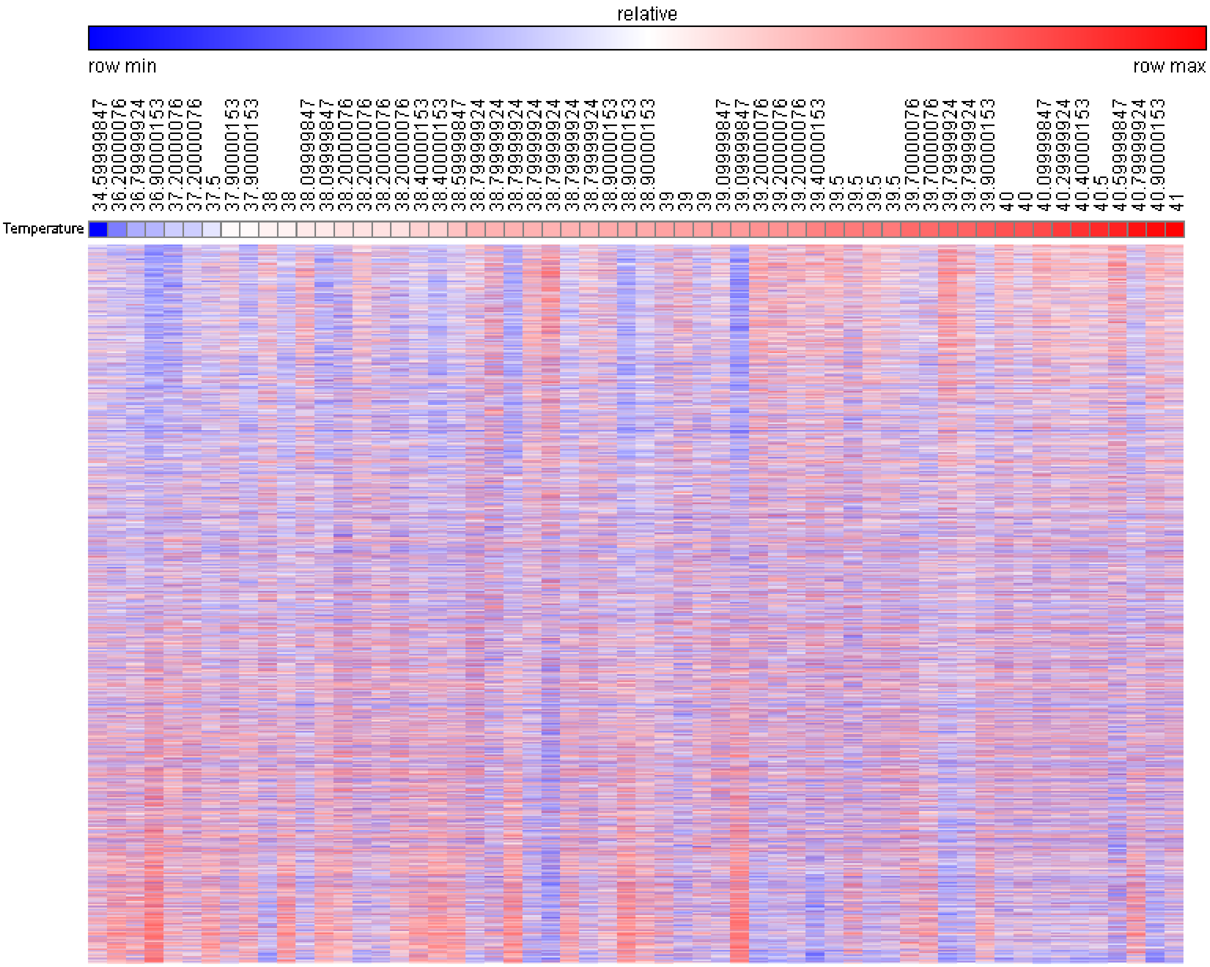

Supplemental Figure 5 (cont.)

f

CORRELATED GENE SETS	SIZE	ES	NES	NOM p-val	FDR q-val
RING STAGE	23	0.64	2.22	0.000	0.000
GAMETOCYTE STAGE	122	0.28	1.42	0.023	0.261
CYTOPLASMIC RIBOSOME - TRANSLATION	706	0.19	1.17	0.065	0.485

ANTI-CORRELATED GENE SETS	SIZE	ES	NES	NOM p-val	FDR q-val
CELL ADHESION	138	-0.42	-2.30	0.000	0.000
CARBOHYDRATE - GLYCOLYSIS	178	-0.40	-2.19	0.000	0.001
PROTEIN FOLDING	94	-0.38	-1.89	0.000	0.004
TROPHOZOITE	869	-0.24	-1.55	0.000	0.049
UBIQUITIN	72	-0.28	-1.30	0.077	0.177
AA - PURINES - NITROGEN	173	-0.22	-1.25	0.092	0.176

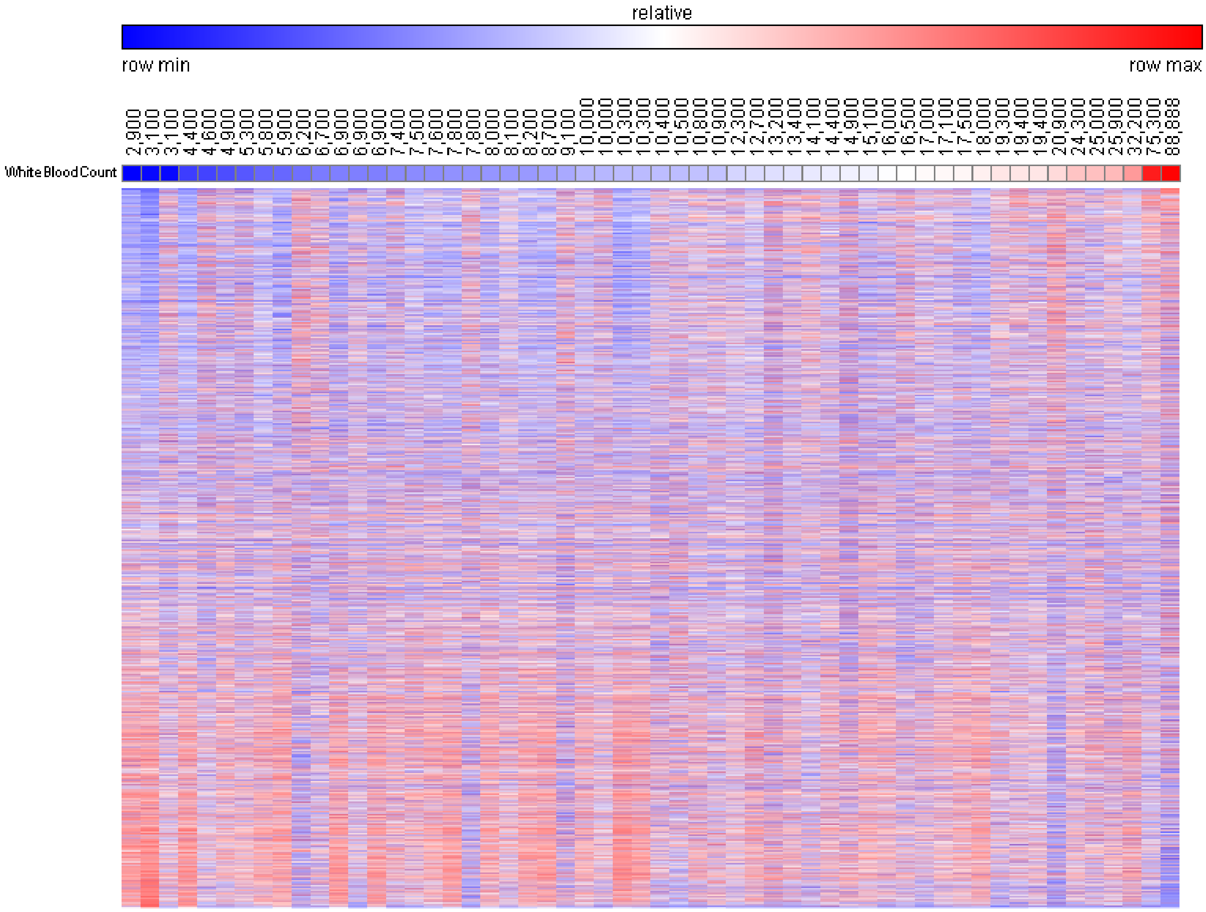

Supplemental Figure 5 (cont.)

g

CORRELATED GENE SETS	SIZE	ES	NES	NOM p-val	FDR q-val
CYTOPLASMIC RIBOSOME - TRANSLATION	706	0.34	1.99	0.000	0.003
GROWTH	588	0.27	1.54	0.000	0.081

ANTI-CORRELATED GENE SETS	SIZE	ES	NES	NOM p-val	FDR q-val
CELL ADHESION	138	-0.36	-1.95	0.000	0.008
INVASION	47	-0.44	-1.85	0.000	0.010
HEMOGLOBIN	21	-0.51	-1.77	0.005	0.016
CELL CYCLE: DNA REPLICATION	26	-0.46	-1.69	0.014	0.025
GAMETOCYTE STAGE	122	-0.28	-1.47	0.009	0.085
CARBOHYDRATE - GLYCOLYSIS	178	-0.25	-1.37	0.022	0.127
TROPHOZOITE	869	-0.18	-1.14	0.063	0.344

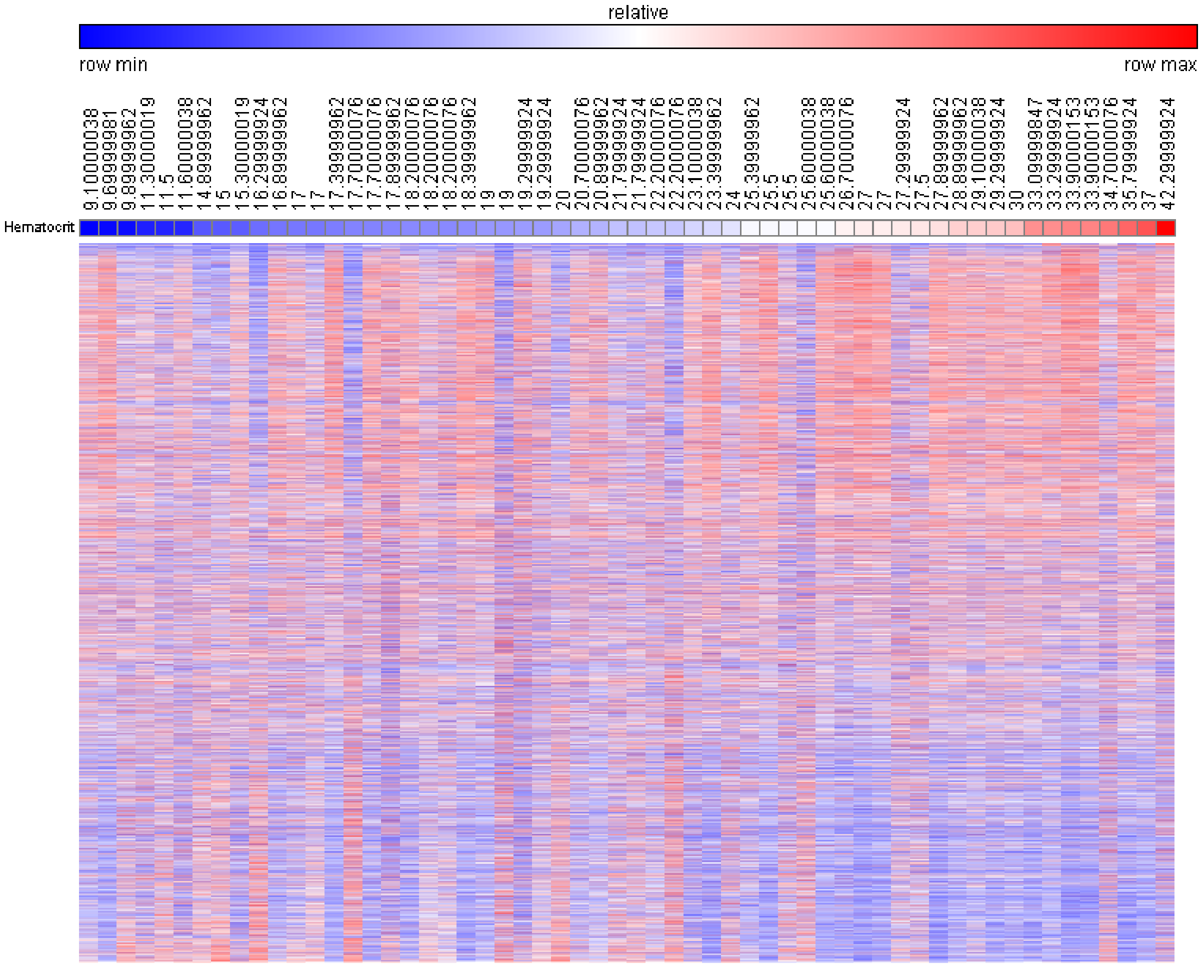

Supplemental Figure 5 (cont.)

h

CORRELATED GENE SETS	SIZE	ES	NES	NOM p-val	FDR q-val
INVASION	47	-0.75	-2.49	0.000	0.000
TROPHOZOITE	869	-0.38	-1.69	0.000	0.012
CELL CYCLE: DNA REPLICATION	26	-0.50	-1.51	0.047	0.055
MITOCHONDRIAL - PLASTID BIOGENESIS	27	-0.47	-1.41	0.065	0.099
CELL ADHESION	138	-0.33	-1.29	0.060	0.182

ANTI-CORRELATED GENE SETS	SIZE	ES	NES	NOM p-val	FDR q-val
UBIQUITIN	72	0.39	1.60	0.008	0.180
RING STAGE	23	0.44	1.39	0.064	0.323
GROWTH	588	0.24	1.30	0.000	0.366
CYTOPLASMIC RIBOSOME - TRANSLATION	706	0.21	1.10	0.063	0.665

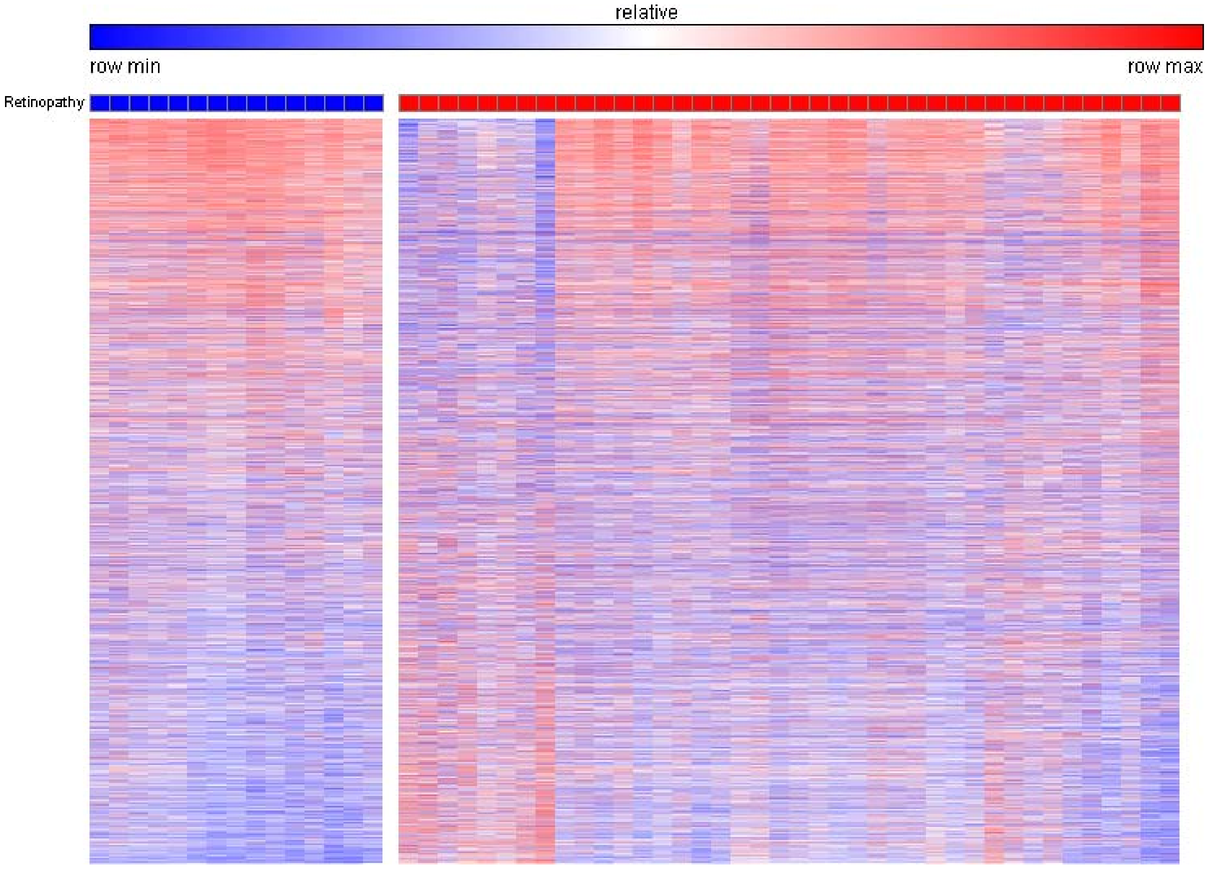

Supplement: Figure S5 — Pathway analysis for continuous clinical variables (a) parasitemia, (b) platelets, (c) glucose, (d) lactate, (e) temperature, (f) white blood count, (g) hematocrit and for categorical variable (h) retinopathy. Genes are sorted according to their association to each of these variables. (PDF) [file pone.0040739.s005.pdf]
