# Supplementary material for: Transcriptional Profiling of Plasmodium falciparum Parasites from Patients with Severe Malaria Identifies Distinct Low vs. High Parasitemic Clusters
Source: PLoS One. 2012 Jul 18;7(7):e40739. doi: 10.1371/journal.pone.0040739 (PMC3399889; doi:10.1371/journal.pone.0040739)
Supplement: Table S1 — The differential expression of genes by Cluster A and B. The distribution of the mean - standard deviation was computed for expression values of genes whose differential expression for A vs. B [yellow and white genes] and B vs. A [green and gray genes] was significant (FDR ≤0.05). “Highly induced” was defined by a positive value for [(mean of high) - (std of high) ] - [(mean of low) - (std of low)] which represents a non-overlapping expression range. Genes shown in yellow are highly induced in Cluster A relative to Cluster B. Genes in gray are induced in Cluster A relative to Cluster B. Genes shown in green are highly induced in Cluster B relative to Cluster A. Genes shown in white are induced in Cluster B relative to Cluster A. (PDF) [file pone.0040739.s006.pdf]

Supplemental Table 1: The differential expression of genes by Cluster A and B. The distribution of the mean - standard deviation was computed for expression values of genes whose differential expression for A vs. B [yellow and white genes] and B vs. A [green and gray genes] was significant (FDR ≤ 0.05). "Highly induced" was defined by a positive value for [(mean of high) - (std of high)] - [(mean of low) - (std of low)] which represents a non-overlapping expression range. Genes shown in yellow are highly induced in Cluster A relative to Cluster B. Genes in gray are induced in Cluster A relative to Cluster B. Genes shown in green are highly induced in Cluster B relative to Cluster A. Genes shown in white are induced in Cluster B relative to Cluster A.

Rank	Feature	Description	Score	Feature P	FDR(BH)	Q Value	Fold Change	Malawi_B Mean	Malawi_B Std	Malawi_A Mean	Malawi_A Std	Difference
Genes highly induced in Cluster A relative to Cluster B												
31	PFD1120c	early transcribed membrane protein 4	-8.22	0.0020	0.0055	0.0018	1.2	7318	1034	9110	621	136
50	PF07_0093	conserved Plasmodium protein, unknown function	-7.73	0.0020	0.0055	0.0018	1.2	7025	517	8185	593	49
11	PF10_0164	early transcribed membrane protein 10.3	-8.94	0.0020	0.0055	0.0018	1.4	6538	1259	8932	775	360
10	PF14_0493	sortilin, putative	-9.02	0.0020	0.0055	0.0018	1.3	5846	827	7746	764	309
94	PFL1885c	protein kinase 2	-7.08	0.0020	0.0055	0.0018	1.3	5645	693	7426	1085	3
29	PFF0665c	syntaxin binding protein, putative	-8.23	0.0020	0.0055	0.0018	1.4	5633	1088	7848	949	177
42	PFF0435w	ornithine aminotransferase	-7.81	0.0020	0.0055	0.0018	1.6	5313	1749	8591	1437	92
54	PFI0002w		-7.60	0.0020	0.0055	0.0018	1.5	5121	1054	7613	1340	98
5	PF13_0345	glycine cleavage T protein, putative	-9.76	0.0020	0.0055	0.0018	1.4	4975	795	7091	825	495
53	PF11_0257	ethanolamine kinase, putative	-7.62	0.0020	0.0055	0.0018	1.4	4792	975	6927	1102	58
28	PF11_0016		-8.25	0.0020	0.0055	0.0018	1.8	4592	2201	8273	1163	317
14	PF11_0172	folate/biopterin transporter, putative	-8.72	0.0020	0.0055	0.0018	1.6	4360	779	6872	1250	483
52	PFE0645w	conserved Plasmodium protein, unknown function	-7.67	0.0020	0.0055	0.0018	1.4	4060	929	5862	845	28
41	MAL13P1.313	conserved Plasmodium protein, unknown function	-7.86	0.0020	0.0055	0.0018	1.6	4017	942	6366	1232	175
18	PF13_0281	conserved Plasmodium protein, unknown function	-8.56	0.0020	0.0055	0.0018	1.5	3895	795	5660	759	212
33	PFD0795w	histone acetyltransferase, putative	-8.10	0.0020	0.0055	0.0018	1.6	3883	1033	6080	1006	157
61	PFD0990w	ribosome recycling factor, putative	-7.54	0.0020	0.0055	0.0018	1.6	3863	897	5992	1160	72
113	PF11_0385	UVB-resistance protein UVR8 homologue	-6.92	0.0020	0.0055	0.0018	1.5	3837	621	5799	1288	54
12	MAL13P1.290	high mobility group protein 4, putative	-8.86	0.0020	0.0055	0.0018	1.7	3652	986	6180	1127	416
110	PF13_0146	conserved Plasmodium protein, unknown function	-7.00	0.0020	0.0055	0.0018	1.8	3611	1010	6327	1700	5
60	PF11_0423	conserved Plasmodium protein, unknown function	-7.54	0.0020	0.0055	0.0018	1.5	3602	743	5288	900	43
32	MAL13P1.118	3',5'-cyclic nucleotide phosphodiesterase, putative	-8.14	0.0020	0.0055	0.0018	1.7	3531	739	5945	1314	361
96	PFF0255c	conserved Plasmodium protein, unknown function	-7.08	0.0020	0.0055	0.0018	1.5	3525	574	5324	1148	77
6	PFC0525c	glycogen synthase kinase 3	-9.66	0.0020	0.0055	0.0018	1.8	3480	1061	6426	1200	685
65	PF14_0152	conserved Plasmodium protein, unknown function	-7.49	0.0020	0.0055	0.0018	1.9	3210	1368	6177	1563	36
4	MAL13P1.139a	conserved Plasmodium protein, unknown function	-9.83	0.0020	0.0055	0.0018	1.9	3157	934	5995	1177	727
9	PFI0400c	conserved Plasmodium membrane protein, unknown fun	-9.07	0.0020	0.0055	0.0018	1.9	3115	873	6001	1376	638
46	MAL8P1.113	peptidase family C50, putative	-7.76	0.0020	0.0055	0.0018	1.6	3025	716	4897	1018	139
22	PF10_0291	RAP protein, putative	-8.50	0.0020	0.0055	0.0018	1.7	2886	674	4962	1054	348
100	PF13_0328	proliferating cell nuclear antigen	-7.06	0.0020	0.0055	0.0018	2.2	2756	961	5942	2059	167
24	PF11_0516	stevor	-8.44	0.0020	0.0055	0.0018	2.4	2666	1699	6420	1645	409
36	PFB0035c	rifin	-7.99	0.0020	0.0055	0.0018	2.3	2612	1396	5935	1665	262

45	PFE0840c	transcription factor with AP2 domain(s), putative	-7.78	0.0020	0.0055	0.0018	1.9	2604	791	4978	1340	244
108	PF08_0126	DNA repair protein rad54, putative	-7.02	0.0020	0.0055	0.0018	1.9	2574	693	4844	1473	103
7	PFL1685w	conserved Plasmodium protein, unknown function	-9.32	0.0020	0.0055	0.0018	2.3	2561	1048	5959	1554	796
1	PFC0070c	Plasmodium exported protein, unknown function	-10.30	0.0020	0.0055	0.0018	2.1	2540	910	5212	1015	747
30	PFA0010c	rifin	-8.23	0.0020	0.0055	0.0018	2.4	2474	1404	5974	1718	377
21	PF11_0010	rifin	-8.50	0.0020	0.0055	0.0018	2.4	2438	1393	5858	1586	442
90	PFL0415w	mitochondrial ACP precursor	-7.14	0.0020	0.0055	0.0018	1.9	2346	821	4568	1360	42
122	MAL7P1.157	conserved Plasmodium protein, unknown function	-6.87	0.0020	0.0055	0.0018	1.8	2341	680	4323	1293	9
2	PF14_0338		-10.25	0.0020	0.0055	0.0018	2.3	2331	789	5468	1346	1003
64	MAL8P1.96	CS domain protein, putative	-7.49	0.0020	0.0055	0.0018	2.0	2091	967	4152	1076	19
80	PFF0650w	apicoplast ribosomal protein L18 precursor, putative	-7.21	0.0020	0.0055	0.0018	1.9	2009	703	3742	1019	11
48	PFL2655w	rifin	-7.74	0.0020	0.0055	0.0018	2.8	2004	1379	5552	1924	245
8	MAL7P1.216	rifin	-9.09	0.0020	0.0055	0.0018	2.8	1999	1231	5690	1699	762
20	PFD0535w	conserved Plasmodium protein, unknown function	-8.51	0.0020	0.0055	0.0018	2.4	1936	1320	4725	1161	308
66	PFL0940c	erythrocyte membrane protein 1 (PfEMP1), pseudogene	-7.48	0.0020	0.0055	0.0018	2.6	1926	1103	4972	1765	177
25	PF10_0399	rifin	-8.42	0.0020	0.0055	0.0018	3.0	1687	1102	4980	1677	515
106	PFD0035c	stevor	-7.03	0.0020	0.0055	0.0018	2.7	1644	960	4415	1754	57
58	PFA0080c	rifin	-7.55	0.0020	0.0055	0.0018	3.1	1628	1273	5016	1920	195
85	PFC0340w	DNA polymerase epsilon subunit b, putative	-7.19	0.0020	0.0055	0.0018	2.1	1548	474	3251	1090	140
68	PFC0911c	conserved Plasmodium protein, unknown function	-7.41	0.0020	0.0055	0.0018	2.2	1543	546	3392	1132	170
123	PFD0170c	conserved Plasmodium protein, unknown function	-6.86	0.0020	0.0055	0.0018	2.1	1490	527	3192	1133	42
51	PFA0090c	stevor	-7.69	0.0020	0.0055	0.0018	3.2	1488	1050	4724	1864	322
71	PF14_0089		-7.31	0.0020	0.0055	0.0018	2.3	1410	730	3199	1029	30
44	PFC0705c	conserved Plasmodium protein, unknown function	-7.79	0.0020	0.0055	0.0018	2.3	1385	683	3190	979	142
87	PFC10_API0043	null	-7.17	0.0020	0.0055	0.0018	2.8	1258	726	3492	1400	109
99	PF08_0138	rifin	-7.07	0.0020	0.0055	0.0018	3.2	1033	655	3267	1447	132
83	PFI1665w	transcription factor with AP2 domain(s), putative	-7.20	0.0020	0.0055	0.0018	3.3	875	463	2870	1301	231
143	MAL7P1.219	rifin	-6.69	0.0020	0.0055	0.0018	3.5	867	674	3006	1461	4
125	MAL8P1.205	Plasmodium exported protein, unknown function	-6.84	0.0020	0.0055	0.0018	2.8	621	323	1766	774	47
Genes induced in Cluster A relative to Cluster B												
2399	PF08_0054	heat shock protein 70	-2.36	0.0240	0.0438	0.0115	1.0	9978	8	9984	9	-12
2405	PF07_0029	heat shock protein 90	-2.36	0.0200	0.0376	0.0099	1.0	9936	32	9953	23	-38
2521	PFB0100c	knob-associated histidine-rich protein	-2.24	0.0259	0.0469	0.0123	1.0	9868	225	9964	92	-221
1603	PF13_0228	40S ribosomal protein S6, putative	-3.26	0.0060	0.0136	0.0036	1.0	9806	65	9857	54	-68
1110	PFB0921c	Plasmodium exported protein, unknown function	-4.00	0.0020	0.0055	0.0018	1.0	9805	53	9876	75	-56
2045	PF14_0448	40S ribosomal protein S2, putative	-2.76	0.0220	0.0408	0.0107	1.0	9757	78	9813	75	-97
1198	PFL2515c	conserved Plasmodium protein, unknown function	-3.84	0.0020	0.0055	0.0018	1.0	9752	239	9935	118	-174
346	PF14_0678	exported protein 2	-5.60	0.0020	0.0055	0.0018	1.0	9675	87	9806	88	-44
610	PF14_0344	translocon component PTEX150	-4.94	0.0020	0.0055	0.0018	1.0	9672	93	9778	70	-57
1013	PFL2335w	conserved Plasmodium protein, unknown function	-4.15	0.0040	0.0098	0.0026	1.0	9585	63	9659	69	-59
2249	PFE0965c	vacuolar ATP synthetase	-2.52	0.0240	0.0438	0.0115	1.0	9548	126	9621	94	-147
2051	PFI0875w	heat shock protein 70	-2.75	0.0180	0.0344	0.0090	1.0	9509	272	9680	201	-302
2202	PFI0765w	conserved Plasmodium protein, unknown function	-2.58	0.0180	0.0344	0.0090	1.0	9444	123	9540	151	-178

1854	PFF1025c	SNO glutamine amidotransferase family protein	-2.97	0.0100	0.0208	0.0055	1.0	9422	244	9654	324	-335
1094	PF10_0121	hypoxanthine phosphoribosyltransferase	-4.03	0.0020	0.0055	0.0018	1.1	9383	680	9869	153	-346
976	PF10_0126	conserved Plasmodium protein, unknown function	-4.21	0.0020	0.0055	0.0018	1.0	9381	108	9524	139	-104
228	PFF0860c	histone H2A	-6.11	0.0020	0.0055	0.0018	1.0	9357	356	9802	195	-106
1967	MAL8P1.17	protein disulfide isomerase	-2.85	0.0080	0.0173	0.0046	1.0	9310	469	9597	296	-478
691	PF11_0508	Plasmodium exported protein, unknown function	-4.74	0.0020	0.0055	0.0018	1.1	9270	595	9769	125	-221
329	PF14_0425	fructose-bisphosphate aldolase	-5.67	0.0020	0.0055	0.0018	1.0	9253	363	9668	189	-136
2078	PF10_0203	ADP-ribosylation factor	-2.73	0.0140	0.0277	0.0073	1.0	9114	359	9339	269	-403
1365	PF14_0228	conserved Plasmodium protein, unknown function	-3.59	0.0020	0.0055	0.0018	1.0	8997	304	9244	219	-276
2270	PF14_0110	rhomboid protease ROM8	-2.50	0.0200	0.0376	0.0099	1.0	8921	300	9125	310	-406
1211	PF07_0007	conserved Plasmodium protein, unknown function	-3.82	0.0040	0.0098	0.0026	1.1	8905	833	9631	614	-721
411	MAL7P1.171	Plasmodium exported protein, unknown function	-5.42	0.0020	0.0055	0.0018	1.1	8900	716	9634	280	-263
2302	PFC0835c	conserved protein, unknown function	-2.47	0.0259	0.0469	0.0123	1.0	8880	348	9123	381	-487
1974	PFD1175w	serine/threonine protein kinase, FIKK family	-2.85	0.0140	0.0277	0.0073	1.1	8869	718	9386	651	-854
112	PF11_0062	histone H2B	-6.92	0.0020	0.0055	0.0018	1.1	8857	647	9676	199	-28
1733	PF11_0093	IWS1-like protein, putative	-3.11	0.0100	0.0208	0.0055	1.0	8856	161	8982	145	-180
851	PFF0090w	conserved Plasmodium protein, unknown function	-4.43	0.0020	0.0055	0.0018	1.1	8841	1008	9714	464	-599
2140	PFD0355c	peptidyl-tRNA hydrolase PTH2, putative	-2.65	0.0080	0.0173	0.0046	1.0	8822	234	8995	253	-314
2025	PFI0630w	26S proteasome regulatory subunit, putative	-2.78	0.0140	0.0277	0.0073	1.0	8794	420	9059	306	-461
839	PFD0495c	conserved Plasmodium protein, unknown function	-4.44	0.0020	0.0055	0.0018	1.1	8782	650	9441	479	-470
1635	MAL8P1.142	20S proteasome beta subunit	-3.22	0.0100	0.0208	0.0055	1.0	8779	510	9138	337	-488
1877	PFF1155w	hexokinase	-2.94	0.0120	0.0243	0.0064	1.0	8744	289	8978	304	-359
777	PF11_0106	apicoplast ribosomal protein L36e precursor, putative	-4.57	0.0020	0.0055	0.0018	1.0	8729	253	9075	304	-211
1503	PF10_0143	transcriptional coactivator ADA2	-3.38	0.0020	0.0055	0.0018	1.0	8727	220	8928	225	-244
1274	PFE1600w	Plasmodium exported protein (PHISTb), unknown functio	-3.73	0.0040	0.0098	0.0026	1.1	8619	1306	9609	702	-1017
1005	PFI1281w	conserved Plasmodium protein, unknown function	-4.16	0.0020	0.0055	0.0018	1.1	8547	604	9037	277	-390
947	PFB0080c	Plasmodium exported protein (PHISTb), unknown functio	-4.26	0.0020	0.0055	0.0018	1.1	8516	886	9417	720	-704
486	PF13_0011	gamete antigen 27/25	-5.21	0.0020	0.0055	0.0018	1.1	8466	923	9410	430	-409
2156	PF11_0098	endoplasmic reticulum-resident calcium binding protein	-2.63	0.0200	0.0376	0.0099	1.1	8371	862	8884	623	-973
1294	PFE0050w	Plasmodium exported protein, unknown function	-3.69	0.0020	0.0055	0.0018	1.1	8326	1260	9352	859	-1093
218	PFB0095c	erythrocyte membrane protein 3	-6.17	0.0020	0.0055	0.0018	1.2	8301	1123	9652	512	-284
1009	PFC0925w	conserved Plasmodium protein, unknown function	-4.15	0.0020	0.0055	0.0018	1.1	8285	514	8803	433	-429
2024	PF11_0161	cysteine proteinase falcipain 2b	-2.78	0.0060	0.0136	0.0036	1.0	8226	456	8605	546	-623
1217	PF14_0746	Plasmodium exported protein (PHISTb), unknown functio	-3.82	0.0020	0.0055	0.0018	1.1	8194	946	9162	955	-933
1583	PF11_0412	vacuolar ATP synthase subunit f, putative	-3.29	0.0060	0.0136	0.0036	1.1	8170	577	8629	481	-599
1203	MAL13P1.190	proteasome regulatory subunit, putative	-3.84	0.0040	0.0098	0.0026	1.1	8098	794	8709	404	-587
1067	PFL1890c	HD superfamily phosphohydrolase protein	-4.06	0.0020	0.0055	0.0018	1.1	8053	428	8500	403	-384
131	PFD0315c		-6.78	0.0020	0.0055	0.0018	1.1	8052	750	9175	511	-137
2251	PFB0545c	mitochondrial ribosomal protein L12 precursor, putative	-2.52	0.0080	0.0173	0.0046	1.0	8019	399	8294	416	-540
2008	PFE0915c	proteasome subunit beta type 1, putative	-2.80	0.0160	0.0311	0.0082	1.1	7946	991	8569	701	-1070
1746	PF07_0005	lysophospholipase, putative	-3.10	0.0020	0.0055	0.0018	1.1	7923	640	8605	934	-892
480	PF07_0044	conserved Plasmodium protein, unknown function	-5.22	0.0020	0.0055	0.0018	1.1	7917	318	8397	362	-201
2098	MAL13P1.63	eukaryotic translation initation factor 4 gamma, putative	-2.70	0.0060	0.0136	0.0036	1.1	7888	680	8395	721	-894

1765	PF13_0065	vacuolar ATP synthase subunit a	-3.08	0.0140	0.0277	0.0073	1.1	7880	919	8540	715	-973
222	PF07_0070	drug metabolite transporter, putative	-6.15	0.0020	0.0055	0.0018	1.1	7875	434	8541	385	-153
331	PF13_0208	exoribonuclease, putative	-5.67	0.0020	0.0055	0.0018	1.1	7869	276	8457	452	-140
1172	PFL1445w	conserved Plasmodium protein, unknown function	-3.89	0.0020	0.0055	0.0018	1.1	7845	584	8360	425	-494
530	PFC0725c	formate-nitrite transporter, putative	-5.09	0.0020	0.0055	0.0018	1.1	7804	1070	8898	549	-524
829	MAL8P1.51	secretory complex protein 61 beta subunit	-4.47	0.0020	0.0055	0.0018	1.1	7789	760	8611	636	-574
1209	PF11_0198	tRNA m(1)G methyltransferase, putative	-3.83	0.0060	0.0136	0.0036	1.1	7761	812	8459	575	-690
742	PFC0085c	Plasmodium exported protein, unknown function	-4.65	0.0020	0.0055	0.0018	1.2	7733	1689	9271	782	-933
1953	PFC0730w	HVA22/TB2/DP1 family protein, putative	-2.87	0.0100	0.0208	0.0055	1.1	7728	785	8305	733	-942
2327	PF11_0329	ubiquitin-like protein, putative	-2.45	0.0160	0.0311	0.0082	1.0	7673	301	7960	516	-530
2448	MAL8P1.48	splicing factor, putative	-2.31	0.0120	0.0243	0.0064	1.0	7638	341	8002	717	-694
268	PF13_0272	thioredoxin-related protein, putative	-5.90	0.0020	0.0055	0.0018	1.2	7596	1390	9187	621	-419
182	PFB0075c	Plasmodium exported protein (hyp9), unknown function	-6.40	0.0020	0.0055	0.0018	1.2	7576	1399	9405	762	-332
2092	PF11_0503	Plasmodium exported protein (PHISTc), unknown functio	-2.71	0.0120	0.0243	0.0064	1.2	7538	1728	8683	1479	-2061
1218	PF14_0017	lysophospholipase, putative	-3.82	0.0040	0.0098	0.0026	1.1	7520	877	8595	1168	-969
1459	PFL1185c	cytochrome c heme lyase, putative	-3.44	0.0060	0.0136	0.0036	1.1	7503	763	8179	718	-804
954	PF07_0090a	conserved Plasmodium protein, unknown function	-4.25	0.0020	0.0055	0.0018	1.1	7501	368	7972	446	-343
1476	PF07_0008	Plasmodium exported protein, unknown function	-3.41	0.0060	0.0136	0.0036	1.2	7449	1323	8665	1346	-1453
1801	PF13_0227	vacuolar ATP synthase subunit d, putative	-3.03	0.0140	0.0277	0.0073	1.1	7407	777	7921	515	-778
1782	MAL7P1.174	Plasmodium exported protein (PHISTb), unknown functio	-3.06	0.0060	0.0136	0.0036	1.2	7401	1707	8709	1527	-1926
2267	PFC10_API0041	null	-2.51	0.0140	0.0277	0.0073	1.1	7380	1260	8250	1331	-1721
513	MAL13P1.343	proteasome regulatory subunit, putative	-5.14	0.0020	0.0055	0.0018	1.1	7377	495	8099	548	-321
433	PF11_0321	serpentine receptor, putative	-5.35	0.0020	0.0055	0.0018	1.2	7373	845	8486	730	-463
340	PFE1605w	Plasmodium exported protein (PHISTb), unknown functio	-5.63	0.0020	0.0055	0.0018	1.3	7363	1754	9379	950	-688
1676	PF13_0287	adenylosuccinate synthetase	-3.18	0.0080	0.0173	0.0046	1.1	7335	861	8165	1055	-1086
1229	MAL13P1.335	phosphatidylserine synthase I, putative	-3.80	0.0020	0.0055	0.0018	1.1	7335	646	8291	1106	-796
1313	PFD0785c	conserved Plasmodium protein, unknown function	-3.66	0.0020	0.0055	0.0018	1.1	7301	767	8018	710	-760
1104	PF11_0292	cochaperone prefoldin complex subunit, putative	-4.02	0.0040	0.0098	0.0026	1.1	7241	823	8102	790	-753
1254	PFB0810w	conserved Plasmodium protein, unknown function	-3.76	0.0020	0.0055	0.0018	1.1	7235	432	7708	498	-457
174	PF10_0159	glycophorin binding protein	-6.45	0.0020	0.0055	0.0018	1.2	7209	1061	8893	916	-293
1358	PF13_0073	Plasmodium exported protein (hyp12), unknown functior	-3.60	0.0060	0.0136	0.0036	1.2	7143	2203	8927	1565	-1985
257	MAL13P1.430		-5.99	0.0020	0.0055	0.0018	1.2	7065	479	8307	932	-169
1654	PF13_0098	conserved Plasmodium protein, unknown function	-3.20	0.0060	0.0136	0.0036	1.1	7010	844	7898	1159	-1115
2198	PF10_0026	tryptophan-rich antigen 3	-2.58	0.0160	0.0311	0.0082	1.1	7007	1362	7934	1335	-1770
1047	PF14_0503	conserved Plasmodium protein, unknown function	-4.09	0.0020	0.0055	0.0018	1.1	6997	537	7918	1007	-623
654	PF10_0359	conserved Plasmodium protein, unknown function	-4.81	0.0020	0.0055	0.0018	1.1	6956	581	7852	770	-455
81	PF08_0060	asparagine-rich antigen	-7.20	0.0020	0.0055	0.0018	1.4	6951	1780	9389	715	-58
225	PFF0400w	conserved Plasmodium protein, unknown function	-6.12	0.0020	0.0055	0.0018	1.1	6872	516	7638	433	-184
1557	MAL8P1.32	nucleoside transporter 2	-3.32	0.0020	0.0055	0.0018	1.1	6871	751	7704	1053	-971
1193	PF14_0377	vesicle-associated membrane protein, putative	-3.85	0.0020	0.0055	0.0018	1.1	6854	904	7731	817	-844
1006	PFB0820c	conserved Plasmodium protein, unknown function	-4.16	0.0020	0.0055	0.0018	1.1	6843	403	7345	485	-386
616	MAL7P1.94	prefoldin subunit 3, putative	-4.90	0.0020	0.0055	0.0018	1.2	6836	991	8009	823	-642
1611	PFF0690c	organic anion transporter	-3.25	0.0040	0.0098	0.0026	1.2	6782	1717	8024	1191	-1665

542	PF14_0124	actin II	-5.06	0.0020	0.0055	0.0018	1.2	6748	1312	8371	1117	-807
193	PFL2075c	conserved Plasmodium protein, unknown function	-6.30	0.0020	0.0055	0.0018	1.2	6725	577	7943	813	-172
2057	PFA0580c	TatD-like deoxyribonuclease, putative	-2.75	0.0120	0.0243	0.0064	1.1	6723	538	7121	547	-687
2010	PFI1030c		-2.80	0.0140	0.0277	0.0073	1.1	6719	907	7424	972	-1173
591	PFD0310w	sexual stage-specific protein precursor	-4.97	0.0020	0.0055	0.0018	1.3	6715	1960	8766	1173	-1082
889	PFI1370c	phosphatidylserine decarboxylase	-4.37	0.0020	0.0055	0.0018	1.1	6674	1032	7636	641	-711
210	PFD0080c	Plasmodium exported protein (PHISTb), unknown functio	-6.22	0.0020	0.0055	0.0018	1.2	6674	794	8341	1132	-259
1864	PFL1225c	conserved Plasmodium protein, unknown function	-2.96	0.0060	0.0136	0.0036	1.1	6659	785	7484	1195	-1155
152	PF11_0310	transporter, putative	-6.63	0.0020	0.0055	0.0018	1.3	6645	1484	8787	973	-315
1342	PFF0120w	geranylgeranyltransferase, putative	-3.63	0.0060	0.0136	0.0036	1.1	6632	725	7300	664	-721
1638	PF13_0050	HORMA domain protein, putative	-3.22	0.0020	0.0055	0.0018	1.1	6616	567	7504	1264	-942
485	PFB0930w	Plasmodium exported protein (hyp9), unknown function	-5.22	0.0020	0.0055	0.0018	1.3	6594	1549	8571	1324	-896
1355	PF07_0129	acyl-coA synthetase, PfACS5	-3.61	0.0040	0.0098	0.0026	1.2	6585	1282	7799	1244	-1312
1763	PFC0605w	conserved Plasmodium protein, unknown function	-3.08	0.0040	0.0098	0.0026	1.1	6569	657	7183	808	-850
1404	PF14_0103	conserved Plasmodium protein, unknown function	-3.53	0.0040	0.0098	0.0026	1.1	6556	580	7461	1156	-831
284	PFL1315w	potassium channel protein	-5.82	0.0020	0.0055	0.0018	1.2	6544	560	7706	857	-255
1876	PF11_0351	heat shock protein 70	-2.94	0.0140	0.0277	0.0073	1.2	6542	1285	7734	1663	-1756
1678	MAL13P1.258	conserved Plasmodium protein, unknown function	-3.18	0.0060	0.0136	0.0036	1.1	6536	573	7212	924	-822
170	PF11_0091	transcription factor with AP2 domain(s), putative	-6.48	0.0020	0.0055	0.0018	1.4	6533	2219	9272	896	-377
702	PFE0060w	parasite-infected erythrocyte surface protein	-4.73	0.0020	0.0055	0.0018	1.3	6533	1341	8471	1664	-1067
1702	PFL1745c	clustered-asparagine-rich protein	-3.15	0.0060	0.0136	0.0036	1.2	6505	2184	8105	1683	-2267
2212	PFB0870w	conserved Plasmodium protein, unknown function	-2.56	0.0160	0.0311	0.0082	1.1	6504	928	7213	1109	-1328
1534	PFI0420c	tRNA pseudouridine synthase, putative	-3.35	0.0040	0.0098	0.0026	1.1	6467	512	6960	580	-599
744	PFD0090c	Plasmodium exported protein (PHISTa), unknown functio	-4.64	0.0020	0.0055	0.0018	1.3	6441	906	8109	1587	-825
2162	PFF1180w	anaphase promoting complex subunit, putative	-2.62	0.0060	0.0136	0.0036	1.1	6422	648	7088	1123	-1104
327	PF08_0131	1-cys peroxiredoxin	-5.68	0.0020	0.0055	0.0018	1.2	6403	854	7861	1033	-429
1055	PFI1255w	zinc binding protein (Yippee), putative	-4.08	0.0020	0.0055	0.0018	1.2	6402	727	7539	1221	-811
138	PF10_0323	early transcribed membrane protein 10.2	-6.71	0.0020	0.0055	0.0018	1.4	6380	1961	9059	1053	-335
709	PFA0195w	parasite-infected erythrocyte surface protein	-4.71	0.0020	0.0055	0.0018	1.2	6269	854	7287	779	-614
1896	PFL1395c	conserved Plasmodium protein, unknown function	-2.93	0.0120	0.0243	0.0064	1.1	6241	1393	7154	982	-1462
741	PF14_0014	Plasmodium exported protein, unknown function	-4.65	0.0020	0.0055	0.0018	1.3	6236	1102	7855	1432	-915
553	PFL1775c	s-adenosyl-methyltransferase, putative	-5.05	0.0020	0.0055	0.0018	1.2	6235	929	7313	697	-548
1955	PFE1230c	mitochondrial import receptor subunit tom22, putative	-2.86	0.0080	0.0173	0.0046	1.1	6232	956	7089	1226	-1325
512	PF11_0483	farnesyltransferase beta subunit, putative	-5.15	0.0020	0.0055	0.0018	1.2	6212	792	7635	1180	-549
597	PFC0570c	conserved Plasmodium protein, unknown function	-4.96	0.0020	0.0055	0.0018	1.2	6163	641	7396	1092	-500
273	PFI1505c	elongation factor Tu, putative	-5.87	0.0020	0.0055	0.0018	1.2	6161	858	7363	698	-354
2360	MAL13P1.146	AMP deaminase, putative	-2.41	0.0200	0.0376	0.0099	1.1	6161	456	6578	759	-797
1552	PFI0535w	phosphatidylinositol N-acetylglucosaminyltransferase, pu	-3.33	0.0020	0.0055	0.0018	1.1	6153	759	7049	1153	-1017
1732	PFE0110w	conserved Plasmodium membrane protein, unknown fun	-3.11	0.0080	0.0173	0.0046	1.1	6145	901	6792	682	-936
789	PF10_0350	probable protein, unknown function	-4.55	0.0020	0.0055	0.0018	1.3	6096	1828	8193	1654	-1385
2371	PFE0165w	actin-depolymerizing factor	-2.40	0.0180	0.0344	0.0090	1.2	6080	2427	7593	2323	-3236
562	PFL0700w	conserved Plasmodium protein, unknown function	-5.03	0.0020	0.0055	0.0018	1.2	6056	709	7367	1128	-526
1319	PFD0955w	apical merozoite protein	-3.66	0.0040	0.0098	0.0026	1.2	6039	875	7272	1479	-1121

1471	MAL13P1.64	ubiquitin-like protein nedd8 homologue, putative	-3.42	0.0020	0.0055	0.0018	1.1	6035	663	6772	897	-823
1486	MAL8P1.71	conserved Plasmodium protein, unknown function	-3.40	0.0020	0.0055	0.0018	1.2	6000	979	6909	1017	-1087
1922	PFL0740c	10 kd chaperonin	-2.90	0.0060	0.0136	0.0036	1.2	5986	735	6950	1509	-1280
1946	PFC0015c	erythrocyte membrane protein 1 (PfEMP1), exon2, pseud	-2.87	0.0060	0.0136	0.0036	1.2	5966	1488	7102	1483	-1835
117	PF14_0270	apicoplast ribosomal protein L15 precursor, putative	-6.90	0.0020	0.0055	0.0018	1.2	5965	701	7440	867	-93
460	PF13_0269	glycerol kinase, putative	-5.28	0.0020	0.0055	0.0018	1.3	5959	713	7547	1346	-470
2282	PF07_0072	calcium dependent protein kinase 4	-2.50	0.0080	0.0173	0.0046	1.1	5944	550	6606	1213	-1101
1745	PF14_0790	conserved Plasmodium protein, unknown function	-3.10	0.0120	0.0243	0.0064	1.1	5936	976	6725	938	-1126
657	PFE0115c	conserved Plasmodium protein, unknown function	-4.81	0.0020	0.0055	0.0018	1.3	5927	738	7419	1388	-634
249	MAL7P1.114	P36-like protein homologue, putative	-6.02	0.0020	0.0055	0.0018	1.2	5920	539	6864	619	-216
647	PF14_0741	hypothetical protein	-4.83	0.0020	0.0055	0.0018	1.3	5912	1635	7734	1237	-1051
1491	PFA0435w	conserved Plasmodium protein, unknown function	-3.40	0.0080	0.0173	0.0046	1.2	5886	1260	6891	988	-1244
153	PF13_0180	cochaperonin	-6.62	0.0020	0.0055	0.0018	1.2	5871	603	7154	803	-123
2335	MAL13P1.65	hypothetical protein	-2.44	0.0240	0.0438	0.0115	1.1	5848	749	6605	1383	-1375
121	PF08_0132	glutamate dehydrogenase, putative	-6.87	0.0020	0.0055	0.0018	1.5	5834	2400	9038	1072	-268
178	MAL8P1.62	conserved Plasmodium protein, unknown function	-6.41	0.0020	0.0055	0.0018	1.3	5816	1214	7730	1050	-349
623	MAL7P1.106	conserved Plasmodium protein, unknown function	-4.90	0.0020	0.0055	0.0018	1.2	5806	517	6867	969	-425
1920	PFI1375w		-2.90	0.0140	0.0277	0.0073	1.1	5788	906	6559	1056	-1190
206	PF10_0374	Pf11-1 protein	-6.23	0.0020	0.0055	0.0018	1.3	5772	961	7730	1311	-314
101	PFC0581w	co-chaperone p23	-7.05	0.0020	0.0055	0.0018	1.3	5771	1010	7723	1059	-117
2082	PF10_0029	conserved Plasmodium protein, unknown function	-2.72	0.0160	0.0311	0.0082	1.1	5765	701	6398	975	-1043
2248	PFL1090w	glideosome-associated protein 45	-2.52	0.0120	0.0243	0.0064	1.2	5751	1059	6717	1650	-1744
1107	PF11_0085		-4.01	0.0020	0.0055	0.0018	1.2	5744	1180	7031	1221	-1115
2067	PFL0820c	conserved Plasmodium protein, unknown function	-2.74	0.0160	0.0311	0.0082	1.1	5744	865	6576	1299	-1332
613	PF13_0319	conserved Plasmodium protein, unknown function	-4.92	0.0020	0.0055	0.0018	1.2	5742	1085	7109	1010	-728
386	PFL2215w	actin I	-5.49	0.0020	0.0055	0.0018	1.5	5735	2194	8455	1579	-1053
955	PF10_0070	conserved Plasmodium membrane protein, unknown fun	-4.25	0.0020	0.0055	0.0018	1.3	5689	1516	7483	1628	-1350
1068	PFI1645c	histidyl-tRNA synthetase, putative	-4.06	0.0020	0.0055	0.0018	1.1	5682	581	6485	837	-615
1566	PFF1150w	ribonuclease HII, putative	-3.31	0.0060	0.0136	0.0036	1.2	5659	1138	6636	1088	-1248
885	MAL8P1.82	vacuolar sorting protein VPS9, putative	-4.37	0.0020	0.0055	0.0018	1.1	5655	626	6501	789	-568
144	MAL13P1.405	conserved protein, unknown function	-6.68	0.0020	0.0055	0.0018	1.5	5652	2068	8431	1063	-352
2049	PFI0485c	SET domain protein, putative	-2.75	0.0080	0.0173	0.0046	1.1	5652	735	6432	1242	-1197
1270	PFD0070c		-3.73	0.0020	0.0055	0.0018	1.3	5636	1780	7113	1235	-1538
1145	PF10_0061	conserved Plasmodium protein, unknown function	-3.94	0.0020	0.0055	0.0018	1.3	5629	910	7059	1607	-1088
462	PF14_0731		-5.28	0.0020	0.0055	0.0018	1.6	5626	2926	8872	1741	-1421
1297	PFF1045w	conserved Plasmodium protein, unknown function	-3.68	0.0020	0.0055	0.0018	1.2	5614	593	6769	1453	-891
1314	PFA0170c	zinc-carboxypeptidase, putative	-3.66	0.0020	0.0055	0.0018	1.2	5604	1089	6887	1451	-1258
1975	PFE0905w	RAP protein, putative	-2.85	0.0120	0.0243	0.0064	1.1	5587	764	6363	1171	-1159
762	MAL13P1.161	conserved Plasmodium protein, unknown function	-4.60	0.0020	0.0055	0.0018	1.2	5566	670	6658	1017	-595
2091	PFC0595c	serine/threonine protein phosphatase, putative	-2.71	0.0160	0.0311	0.0082	1.2	5561	1335	6529	1343	-1710
782	PFI1463w	conserved Plasmodium protein, unknown function	-4.57	0.0020	0.0055	0.0018	1.2	5550	789	6897	1283	-726
528	PF14_0426	conserved Plasmodium protein, unknown function	-5.09	0.0020	0.0055	0.0018	1.3	5543	1041	7141	1264	-706
2329	PF11_0287	CRAL/TRIO domain-containing protein, putative	-2.44	0.0200	0.0376	0.0099	1.1	5493	576	6057	1022	-1034

896	PFD0985w	transcription factor with AP2 domain(s), putative	-4.36	0.0020	0.0055	0.0018	1.3	5487	1706	7101	1112	-1204
1660	PF14_0227	serine/threonine protein kinase, putative	-3.20	0.0040	0.0098	0.0026	1.1	5486	634	6295	1118	-943
1842	PFC0360w	activator of Hsp90 ATPase, putative	-2.98	0.0160	0.0311	0.0082	1.2	5479	1456	6664	1515	-1785
314	PF10_0256		-5.72	0.0020	0.0055	0.0018	1.3	5455	983	7277	1325	-486
358	PFA0570w	conserved Plasmodium protein, unknown function	-5.57	0.0020	0.0055	0.0018	1.3	5431	938	6865	986	-490
2119	MAL8P1.110	apicoplast ribosomal protein L33 precursor, putative	-2.67	0.0080	0.0173	0.0046	1.1	5430	823	6195	1222	-1279
939	MAL13P1.51	secretory complex protein 61 alpha,Rab GTPase 5b	-4.28	0.0020	0.0055	0.0018	1.2	5429	607	6552	1180	-664
2379	PF13_0226	conserved Plasmodium protein, unknown function	-2.39	0.0160	0.0311	0.0082	1.1	5394	722	6121	1363	-1357
2053	PFL2370c	conserved Plasmodium protein, unknown function	-2.75	0.0160	0.0311	0.0082	1.1	5393	922	6163	1130	-1282
1993	PFE0340c	rhomboid protease ROM4	-2.82	0.0060	0.0136	0.0036	1.2	5391	912	6466	1704	-1540
599	PF14_0091		-4.95	0.0020	0.0055	0.0018	1.3	5387	1104	7162	1490	-819
1049	PFL2245w	signal recognition particle receptor, beta subunit	-4.09	0.0020	0.0055	0.0018	1.3	5383	1070	6980	1690	-1163
2466	PFC0200w		-2.29	0.0259	0.0469	0.0123	1.2	5382	1406	6250	1428	-1967
1576	PFL1385c	merozoite surface protein 9	-3.30	0.0080	0.0173	0.0046	1.3	5376	1418	6998	2095	-1891
757	PF14_0516	serine/threonine protein kinase, putative	-4.61	0.0020	0.0055	0.0018	1.3	5366	856	6710	1233	-745
140	MAL13P1.295	conserved Plasmodium protein, unknown function	-6.71	0.0020	0.0055	0.0018	1.4	5344	922	7704	1540	-102
1553	PFF1585w	rifin, pseudogene	-3.33	0.0080	0.0173	0.0046	1.3	5337	1568	6694	1505	-1715
2461	PF14_0034	translocation associated membrane protein, putative	-2.30	0.0220	0.0408	0.0107	1.1	5288	947	5936	1129	-1429
1478	PF14_0155	serine C-palmitoyltransferase, putative	-3.41	0.0020	0.0055	0.0018	1.2	5281	568	6241	1295	-903
1441	PF13_0031	conserved Plasmodium protein, unknown function	-3.47	0.0040	0.0098	0.0026	1.2	5248	934	6236	1151	-1098
596	PFL2180w	mitochondrial ribosomal protein L3 precursor, putative	-4.96	0.0020	0.0055	0.0018	1.3	5232	939	6642	1147	-676
1794	PF14_0682	conserved Plasmodium membrane protein, unknown fun	-3.04	0.0040	0.0098	0.0026	1.2	5230	765	6192	1411	-1214
1610	PFF0610c	PP-loop family protein, putative	-3.25	0.0100	0.0208	0.0055	1.1	5216	737	5843	712	-822
669	PF13_0242	isocitrate dehydrogenase (NADP), mitochondrial precurso	-4.78	0.0020	0.0055	0.0018	1.2	5209	906	6385	935	-665
822	PF14_0309	protein-L-isoaspartate O-methyltransferase beta-asparta	-4.48	0.0020	0.0055	0.0018	1.3	5208	710	6587	1385	-717
1153	MAL13P1.284	pyrroline carboxylate reductase	-3.92	0.0040	0.0098	0.0026	1.3	5186	1317	6851	1765	-1417
491	MAL7P1.209	AAA family ATPase, putative	-5.21	0.0020	0.0055	0.0018	1.3	5174	963	6750	1242	-629
1128	PFB0923c	Plasmodium exported protein, unknown function	-3.96	0.0020	0.0055	0.0018	1.4	5171	1617	7343	2315	-1760
1623	PF14_0775	conserved Plasmodium protein, unknown function	-3.24	0.0040	0.0098	0.0026	1.2	5156	1118	6156	1183	-1301
1341	PF11_0277	conserved Plasmodium protein, unknown function	-3.63	0.0020	0.0055	0.0018	1.2	5153	738	6330	1461	-1021
602	PF13_0099	conserved Plasmodium protein, unknown function	-4.95	0.0020	0.0055	0.0018	1.4	5146	1314	7106	1596	-950
302	PF14_0730	Plasmodium exported protein (PHISTb), unknown functio	-5.78	0.0020	0.0055	0.0018	1.7	5143	2878	8696	1798	-1124
1879	PFL2535w	Plasmodium exported protein (PHISTb), unknown functio	-2.94	0.0020	0.0055	0.0018	1.1	5141	935	5864	912	-1123
2350	PFF0495w	mitochondrial ribosomal protein L19 precursor, putative	-2.42	0.0160	0.0311	0.0082	1.1	5134	577	5702	1043	-1052
1812	PFC0640w	CSP and TRAP-related protein	-3.02	0.0080	0.0173	0.0046	1.2	5126	1371	6351	1620	-1766
1837	PFE0595w	prefoldin subunit, putative	-2.99	0.0080	0.0173	0.0046	1.3	5126	1938	6682	1963	-2345
1060	PF08_0122	conserved Plasmodium protein, unknown function	-4.07	0.0020	0.0055	0.0018	1.2	5114	616	6321	1358	-767
937	PF11_0059	metabolite/drug transporter, putative	-4.28	0.0020	0.0055	0.0018	1.2	5101	746	6106	964	-704
2459	PFE1290w	NIMA related kinase 2	-2.30	0.0220	0.0408	0.0107	1.2	5099	1019	6019	1765	-1864
1031	PFL1330c	cyclin	-4.12	0.0020	0.0055	0.0018	1.2	5073	710	6341	1385	-827
1199	PF11_0056	conserved Plasmodium protein, unknown function	-3.84	0.0020	0.0055	0.0018	1.3	5071	771	6389	1550	-1004
1347	PFI1575c	peptide release factor, putative	-3.63	0.0020	0.0055	0.0018	1.2	5066	776	6237	1441	-1046
2593	PFE0875c	conserved Plasmodium protein, unknown function	-2.16	0.0259	0.0469	0.0123	1.1	5063	1116	5659	978	-1497

2565	PFE1540w	CPW-WPC family protein	-2.19	0.0259	0.0469	0.0123	1.2	5059	935	5914	1741	-1821
1442	MAL13P1.58	Plasmodium exported protein (PHISTa-like), unknown fun	-3.47	0.0040	0.0098	0.0026	1.4	5056	2088	6904	1931	-2172
1191	MAL13P1.148	myosin C	-3.86	0.0020	0.0055	0.0018	1.2	5056	937	6155	1152	-990
203	PF11_0413	conserved Plasmodium protein, unknown function	-6.24	0.0020	0.0055	0.0018	1.5	5051	1210	7341	1482	-402
1691	PFF0205w	mitochondrial ribosomal protein L41 precursor, putative	-3.16	0.0060	0.0136	0.0036	1.2	5023	642	5929	1295	-1031
719	PF10_0238	conserved Plasmodium protein, unknown function	-4.69	0.0020	0.0055	0.0018	1.2	5017	564	6052	971	-500
2604	PFE0220w	conserved Plasmodium protein, unknown function	-2.15	0.0220	0.0408	0.0107	1.2	5012	797	5822	1722	-1709
728	PF13_0164	conserved Plasmodium protein, unknown function	-4.68	0.0020	0.0055	0.0018	1.3	5009	803	6269	1133	-677
1394	PFL0285w	targeted glyoxalase II	-3.55	0.0020	0.0055	0.0018	1.2	4987	714	5789	932	-844
1597	PFE0480c	conserved Plasmodium protein, unknown function	-3.27	0.0040	0.0098	0.0026	1.2	4987	467	6134	1673	-993
1361	PFL0960w	D-ribulose-5-phosphate 3-epimerase, putative	-3.60	0.0020	0.0055	0.0018	1.2	4971	939	5994	1148	-1064
2592	PF11_0141	UDP-galactose transporter, putative	-2.16	0.0220	0.0408	0.0107	1.1	4963	962	5674	1399	-1650
758	MAL7P1.163	conserved Plasmodium protein, unknown function	-4.61	0.0020	0.0055	0.0018	1.2	4954	581	6059	1067	-543
929	PFE0520c	topoisomerase I	-4.30	0.0020	0.0055	0.0018	1.4	4953	1637	6808	1603	-1385
237	PFL1495w	conserved Plasmodium protein, unknown function	-6.07	0.0020	0.0055	0.0018	1.3	4950	482	6291	1005	-145
2088	PF10_0080	endonuclease, putative	-2.71	0.0120	0.0243	0.0064	1.2	4943	834	5983	1742	-1537
426	PF14_0681	diacylglycerol kinase, putative	-5.39	0.0020	0.0055	0.0018	1.3	4939	653	6253	1060	-400
765	MAL8P1.130	conserved Plasmodium membrane protein, unknown fun	-4.60	0.0020	0.0055	0.0018	1.3	4937	1179	6371	1162	-907
2599	PF11_0215	conserved Plasmodium protein, unknown function	-2.15	0.0259	0.0469	0.0123	1.1	4925	1018	5503	1002	-1441
2047	PF13_0235	transcription factor with AP2 domain(s), putative	-2.76	0.0180	0.0344	0.0090	1.2	4920	1603	6117	1648	-2054
1415	PF13_0090a	conserved Plasmodium protein, unknown function	-3.51	0.0020	0.0055	0.0018	1.3	4912	767	6153	1609	-1135
733	PF10_0375	Plasmodium exported protein, unknown function	-4.67	0.0020	0.0055	0.0018	1.3	4908	803	6341	1344	-713
2268	PF14_0268	conserved Plasmodium protein, unknown function	-2.50	0.0160	0.0311	0.0082	1.1	4905	608	5567	1190	-1136
791	PFL0095c	conserved protein, unknown function	-4.54	0.0020	0.0055	0.0018	1.2	4882	866	5971	921	-699
598	PFC0845c	ubiquitin-protein ligase, putative	-4.96	0.0020	0.0055	0.0018	1.2	4881	599	5949	926	-458
2104	PFF0240c	conserved Plasmodium protein, unknown function	-2.70	0.0100	0.0208	0.0055	1.2	4880	774	5684	1307	-1278
2210	PFL0235w	conserved Plasmodium protein, unknown function	-2.57	0.0100	0.0208	0.0055	1.1	4870	604	5383	837	-929
1493	MAL13P1.129	conserved Plasmodium protein, unknown function	-3.40	0.0020	0.0055	0.0018	1.2	4869	873	6085	1593	-1250
118	PFC0315c	conserved Plasmodium protein, unknown function	-6.89	0.0020	0.0055	0.0018	1.4	4865	777	6660	1098	-79
1283	PF11_0248	mitochondrial ribosomal protein L37, putative	-3.70	0.0020	0.0055	0.0018	1.2	4862	843	5982	1303	-1026
2481	MAL13P1.42	recombinase, putative	-2.28	0.0220	0.0408	0.0107	1.1	4859	624	5506	1288	-1265
1725	MAL13P1.140	conserved Plasmodium protein, unknown function	-3.12	0.0060	0.0136	0.0036	1.2	4859	684	5696	1184	-1031
2106	PF11_0560	conserved protein, unknown function	-2.69	0.0080	0.0173	0.0046	1.2	4845	1216	5808	1423	-1676
1567	PFC0345w	conserved Plasmodium protein, unknown function	-3.31	0.0020	0.0055	0.0018	1.2	4843	1123	6026	1476	-1416
2145	PF14_0400	GTP binding protein, putative	-2.64	0.0140	0.0277	0.0073	1.2	4836	793	5694	1448	-1383
1704	PFC0325c	conserved Plasmodium protein, unknown function	-3.15	0.0020	0.0055	0.0018	1.2	4834	598	5716	1277	-994
1853	PFL0730w	conserved Plasmodium protein, unknown function	-2.97	0.0060	0.0136	0.0036	1.2	4825	1147	5946	1577	-1603
518	PFD0365c	conserved Plasmodium protein, unknown function	-5.13	0.0020	0.0055	0.0018	1.3	4824	891	6253	1140	-602
787	PFL0270c	conserved Plasmodium protein, unknown function	-4.56	0.0020	0.0055	0.0018	1.2	4820	845	5874	883	-675
2437	PFE1555c	conserved Plasmodium protein, unknown function	-2.32	0.0220	0.0408	0.0107	1.2	4802	1038	5549	1315	-1606
1346	PF10_0376	Plasmodium exported protein, unknown function	-3.63	0.0020	0.0055	0.0018	1.3	4770	914	6108	1636	-1212
1863	PF13_0237	conserved Plasmodium protein, unknown function	-2.96	0.0020	0.0055	0.0018	1.3	4769	921	5969	1827	-1549
1440	PF11_0188	heat shock protein 90, putative	-3.47	0.0020	0.0055	0.0018	1.3	4762	1292	6320	1912	-1645

638	PFL1795c	conserved Plasmodium protein, unknown function	-4.85	0.0020	0.0055	0.0018	1.3	4761	675	5994	1108	-550
547	PF14_0427	conserved Plasmodium protein, unknown function	-5.06	0.0020	0.0055	0.0018	1.2	4748	649	5925	1001	-473
1096	PFI1565w	profilin, putative	-4.03	0.0020	0.0055	0.0018	1.4	4746	1514	6629	1906	-1536
1577	PF13_0189	conserved Plasmodium protein, unknown function	-3.30	0.0040	0.0098	0.0026	1.1	4734	710	5439	861	-867
150	PFC0950c	peptidase, putative	-6.63	0.0020	0.0055	0.0018	1.4	4715	754	6389	1061	-142
1852	MAL13P1.495	rifin	-2.97	0.0080	0.0173	0.0046	1.3	4703	1830	5930	1313	-1917
155	PFL0630w	iron-sulfur subunit of succinate dehydrogenase	-6.59	0.0020	0.0055	0.0018	1.4	4699	883	6362	988	-208
1750	PF11_0079	protein kinase , putative	-3.10	0.0060	0.0136	0.0036	1.1	4680	527	5267	817	-757
2093	PF11_0424	conserved Plasmodium protein, unknown function	-2.71	0.0140	0.0277	0.0073	1.2	4679	635	5402	1195	-1107
1817	PFD0870w	conserved Plasmodium protein, unknown function	-3.01	0.0040	0.0098	0.0026	1.2	4675	633	5434	1112	-987
1149	PF14_0111	conserved Plasmodium protein, unknown function	-3.92	0.0020	0.0055	0.0018	1.2	4657	1033	5682	939	-948
898	PFD1050w	alpha tubulin 2	-4.36	0.0020	0.0055	0.0018	1.3	4651	874	6040	1378	-863
1039	PFE1440c	conserved Plasmodium protein, unknown function	-4.10	0.0020	0.0055	0.0018	1.2	4647	685	5720	1145	-756
2180	PFF0580w	lsm12, putative	-2.61	0.0180	0.0344	0.0090	1.2	4644	995	5390	1125	-1374
921	PF11_0273		-4.32	0.0020	0.0055	0.0018	1.2	4622	659	5539	880	-622
2151	PFL1675c	CPW-WPC family protein	-2.63	0.0120	0.0243	0.0064	1.2	4620	787	5636	1770	-1541
2016	PF14_0273	rRNA (adenosine-2'-O-)-methyltransferase, putative	-2.79	0.0160	0.0311	0.0082	1.3	4611	2006	6023	1815	-2410
88	PF11_0013	stevor, pseudogene	-7.15	0.0020	0.0055	0.0018	1.6	4610	1535	7345	1360	-159
1288	PF11_0506	antigen 332, DBL-like protein	-3.69	0.0020	0.0055	0.0018	1.4	4610	1770	6552	2103	-1931
1117	PFA0465c	N-acetyltransferase, putative	-3.99	0.0020	0.0055	0.0018	1.3	4597	1325	5957	1245	-1210
570	PF08_0012	SET domain protein, putative	-5.01	0.0020	0.0055	0.0018	1.4	4588	1223	6355	1389	-845
800	PFC0185w	membrane skeletal protein IMC1-related	-4.52	0.0020	0.0055	0.0018	1.3	4574	853	6167	1572	-831
515	PF11_0058		-5.14	0.0020	0.0055	0.0018	1.5	4565	1718	6870	1657	-1070
893	PFA0190c	actin-related protein	-4.37	0.0020	0.0055	0.0018	1.3	4545	749	5795	1255	-753
2265	PF11_0060	calcium/calmodulin-dependent protein kinase, putative	-2.51	0.0160	0.0311	0.0082	1.2	4529	968	5468	1645	-1674
2176	PFC1025w	conserved Plasmodium protein, unknown function	-2.61	0.0220	0.0408	0.0107	1.3	4522	1758	5896	2114	-2498
916	PF08_0117	AAA family ATPase, putative	-4.33	0.0020	0.0055	0.0018	1.2	4520	744	5509	929	-684
847	PFL1150c	mitochondrial ribosomal protein L24-2 precursor, putativ	-4.44	0.0020	0.0055	0.0018	1.3	4513	1042	6008	1400	-946
78	PFB0600c	conserved Plasmodium protein, unknown function	-7.21	0.0020	0.0055	0.0018	1.3	4507	615	5912	802	-12
2110	PF14_0523	protein phosphatase, putative	-2.69	0.0140	0.0277	0.0073	1.2	4504	990	5430	1471	-1535
169	MAL13P1.124	conserved Plasmodium protein, unknown function	-6.48	0.0020	0.0055	0.0018	1.4	4493	922	6450	1259	-225
2288	PFI0845w	conserved Plasmodium membrane protein, unknown fun	-2.49	0.0140	0.0277	0.0073	1.2	4493	815	5315	1462	-1455
869	PF08_0134	conserved Plasmodium protein, unknown function	-4.41	0.0020	0.0055	0.0018	1.3	4491	704	5896	1446	-744
1142	PFL0505c	syntaxin, Qa-SNARE family	-3.94	0.0020	0.0055	0.0018	1.2	4487	484	5309	937	-600
128	PF10_0211	conserved Plasmodium membrane protein, unknown fun	-6.81	0.0020	0.0055	0.0018	1.5	4472	1139	6743	1324	-193
230	PFC0955w	ATP-dependent RNA helicase, putative	-6.10	0.0020	0.0055	0.0018	1.5	4449	1049	6465	1357	-391
1064	PFL1295w	conserved Plasmodium protein, unknown function	-4.07	0.0020	0.0055	0.0018	1.3	4439	1351	5685	980	-1086
1664	PFA0295c	conserved Plasmodium protein, unknown function	-3.19	0.0040	0.0098	0.0026	1.2	4437	772	5416	1356	-1149
2325	PF07_0047	AAA family ATPase, CDC48 subfamily	-2.45	0.0220	0.0408	0.0107	1.2	4428	1070	5225	1314	-1587
627	PFF0750w	cdc2-related protein kinase 5	-4.87	0.0020	0.0055	0.0018	1.3	4424	716	5862	1315	-593
2301	PFE0150c	4-diphosphocytidyl-2c-methyl-D-erythritol kinase (CMK),	-2.48	0.0140	0.0277	0.0073	1.1	4414	852	5008	931	-1190
242	PF14_0300	syntaxin, Qa-SNARE family	-6.06	0.0020	0.0055	0.0018	1.3	4385	700	5823	1004	-266
989	PF11_0177	deubiquinating/deneddylating enzyme	-4.18	0.0020	0.0055	0.0018	1.3	4385	972	5755	1381	-984

556	PFI1587c	conserved Plasmodium protein, unknown function	-5.04	0.0020	0.0055	0.0018	1.3	4377	823	5761	1155	-594
1444	PFB0610c	conserved Plasmodium protein, unknown function	-3.47	0.0040	0.0098	0.0026	1.2	4367	783	5131	857	-875
2218	PFF1120c	conserved Apicomplexan protein, unknown function	-2.56	0.0120	0.0243	0.0064	1.1	4354	638	4931	967	-1028
1279	PF14_0029	conserved Plasmodium protein, unknown function	-3.72	0.0020	0.0055	0.0018	1.3	4341	672	5476	1385	-922
59	PF13_0241	rhomboid protease ROM6, putative	-7.54	0.0020	0.0055	0.0018	1.5	4314	1167	6533	1056	-5
1132	PFD1110w	glideosome associated protein with multiple membrane s	-3.95	0.0020	0.0055	0.0018	1.4	4313	1025	6060	1986	-1264
614	PFC0710w		-4.91	0.0020	0.0055	0.0018	1.5	4310	1116	6484	1955	-897
111	PF11_0210	metal ion channel - Mg2 , Co2 and Ni2	-6.94	0.0020	0.0055	0.0018	1.6	4305	1298	6927	1496	-173
2160	PFC0281w	conserved Plasmodium protein, unknown function	-2.62	0.0060	0.0136	0.0036	1.2	4290	714	5100	1391	-1295
183	PFL1140w	integral membrane protein, putative	-6.40	0.0020	0.0055	0.0018	1.4	4285	798	6090	1209	-202
1463	PFI1165c	conserved Plasmodium protein, unknown function	-3.44	0.0020	0.0055	0.0018	1.2	4283	620	5353	1434	-984
1187	PF14_0177b	conserved Plasmodium protein, unknown function	-3.86	0.0020	0.0055	0.0018	1.2	4282	690	5142	924	-754
1356	PFI1555w	conserved Plasmodium protein, unknown function	-3.61	0.0020	0.0055	0.0018	1.3	4278	895	5561	1567	-1180
1909	MAL8P1.78	small heat shock protein, putative	-2.91	0.0040	0.0098	0.0026	1.2	4275	594	5215	1501	-1154
910	PF14_0447	glutaminyl-peptide cyclotransferase, putative	-4.34	0.0040	0.0098	0.0026	1.4	4272	1586	6051	1504	-1312
148	PFE1245w	conserved Plasmodium protein, unknown function	-6.66	0.0020	0.0055	0.0018	1.7	4267	1712	7427	1826	-377
1058	MAL8P1.153	transcription factor with AP2 domain(s), putative	-4.07	0.0020	0.0055	0.0018	1.4	4263	766	5771	1696	-955
1372	MAL7P1.38	regulator of chromosome condensation, putative	-3.58	0.0020	0.0055	0.0018	1.5	4254	2115	6244	2062	-2187
1312	PFI0705w	conserved Plasmodium protein, unknown function	-3.66	0.0020	0.0055	0.0018	1.3	4253	658	5528	1614	-996
97	PFF0815w	malate:quinone oxidoreductase, putative	-7.08	0.0020	0.0055	0.0018	1.4	4231	850	5906	914	-88
2097	PF14_0036	phosphatase, putative	-2.70	0.0120	0.0243	0.0064	1.4	4210	1841	5809	2453	-2696
1045	PFE0315c	conserved Plasmodium protein, unknown function	-4.09	0.0020	0.0055	0.0018	1.4	4196	891	5877	1868	-1079
190	PF07_0100	conserved Plasmodium protein, unknown function	-6.35	0.0020	0.0055	0.0018	1.4	4184	653	5934	1234	-137
1093	PF14_0696	conserved Plasmodium protein, unknown function	-4.03	0.0020	0.0055	0.0018	1.2	4178	752	5132	973	-771
2285	PFC10_API0028	null	-2.50	0.0140	0.0277	0.0073	1.2	4172	1567	5155	1410	-1994
899	PF14_0527	conserved Plasmodium protein, unknown function	-4.36	0.0020	0.0055	0.0018	1.4	4170	1534	5880	1426	-1250
2375	PF10_0362	DNA polymerase zeta catalytic subunit, putative	-2.39	0.0220	0.0408	0.0107	1.2	4168	827	4913	1358	-1440
687	PF10_0317	DER1-like protein, putative	-4.75	0.0020	0.0055	0.0018	1.4	4156	1137	5730	1312	-876
974	MAL13P1.195	CPW-WPC family protein	-4.21	0.0020	0.0055	0.0018	1.4	4150	796	5853	1866	-959
1453	PF13_0246	conserved Plasmodium protein, unknown function	-3.46	0.0020	0.0055	0.0018	1.2	4124	664	4943	1017	-862
1307	MAL8P1.146	filament assembling protein, putative	-3.67	0.0020	0.0055	0.0018	1.3	4118	875	5409	1560	-1144
933	PFL0660w	dynein light chain 1, putative	-4.29	0.0020	0.0055	0.0018	1.3	4114	794	5180	1018	-745
877	PFL0735w	cyclophilin	-4.39	0.0020	0.0055	0.0018	1.3	4102	579	5305	1252	-628
1619	PFI1125c	beta-ketoacyl-acyl carrier protein reductase	-3.25	0.0020	0.0055	0.0018	1.2	4093	851	5088	1319	-1175
2258	PFC0530w	transporter, putative	-2.52	0.0220	0.0408	0.0107	1.1	4088	736	4558	674	-940
2181	PFL1955w	erythrocyte membrane protein 1, PfEMP1	-2.61	0.0100	0.0208	0.0055	1.2	4084	1147	4812	974	-1392
808	PF14_0363	metacaspase-like protein	-4.51	0.0020	0.0055	0.0018	1.4	4072	1050	5533	1319	-908
1400	PFC0580c	conserved Plasmodium protein, unknown function	-3.54	0.0020	0.0055	0.0018	1.3	4045	815	5190	1428	-1098
1924	PF14_0495	rhoptry neck protein 2	-2.90	0.0080	0.0173	0.0046	1.3	4045	922	5176	1751	-1541
2389	PFI1216w	telomeric repeat binding factor 1	-2.37	0.0160	0.0311	0.0082	1.2	4032	681	4970	1848	-1591
978	PF13_0040	DNA-directed RNA polymerase alpha chain, putative	-4.20	0.0020	0.0055	0.0018	1.2	4031	553	5005	1036	-615
655	PF14_0099	alpha/beta hydrolase, putative	-4.81	0.0020	0.0055	0.0018	1.3	4029	623	5181	1050	-521
508	PF11_0046		-5.16	0.0020	0.0055	0.0018	1.5	4025	795	5857	1607	-570

560	PF13_0101	conserved Plasmodium protein, unknown function	-5.04	0.0020	0.0055	0.0018	1.4	4005	1102	5519	1145	-734
1754	PFC0416w	conserved Plasmodium protein, unknown function	-3.08	0.0060	0.0136	0.0036	1.2	3997	699	4719	983	-961
1177	PF14_0148	uracil-DNA glycosylase, putative	-3.88	0.0020	0.0055	0.0018	1.2	3991	760	4956	1037	-833
1640	PFB0890c	pseudouridine synthase, putative	-3.22	0.0040	0.0098	0.0026	1.2	3990	634	4813	1133	-943
446	PF14_0272	CPW-WPC family protein	-5.32	0.0020	0.0055	0.0018	1.5	3988	959	5850	1512	-610
532	PFC0912w	signal peptidase protein	-5.09	0.0020	0.0055	0.0018	1.2	3983	580	4970	815	-409
2182	PFE0240w	conserved Plasmodium protein, unknown function	-2.60	0.0120	0.0243	0.0064	1.2	3979	733	4810	1437	-1339
872	PF11_0338	aquaglyceroporin	-4.40	0.0020	0.0055	0.0018	1.4	3979	979	5714	1748	-991
994	PFF1455c	CPW-WPC family protein	-4.17	0.0020	0.0055	0.0018	1.5	3976	867	5793	2005	-1055
89	MAL8P1.214	stevor, pseudogene	-7.14	0.0020	0.0055	0.0018	1.8	3966	1803	7227	1646	-189
581	PFC0180c	inner membrane complex protein 1a, putative	-4.99	0.0020	0.0055	0.0018	1.4	3928	897	5528	1379	-676
1832	PFL0545w	kinesin-like protein, putative	-3.00	0.0040	0.0098	0.0026	1.3	3923	783	5201	1984	-1489
2004	PFD0910w	conserved Plasmodium protein, unknown function	-2.81	0.0060	0.0136	0.0036	1.4	3913	940	5355	2389	-1888
1938	PFB0570w	secreted protein altered thrombospondin repeat protein	-2.88	0.0040	0.0098	0.0026	1.3	3906	1220	5175	1900	-1852
2168	PFB0575c	conserved Plasmodium protein, unknown function	-2.61	0.0040	0.0098	0.0026	1.2	3903	925	4716	1309	-1422
442	PFL1555w	cytochrome b5, putative	-5.33	0.0020	0.0055	0.0018	1.5	3875	737	5694	1552	-470
323	PF14_0345a	conserved protein, unknown function	-5.69	0.0020	0.0055	0.0018	1.4	3854	797	5277	1026	-400
982	MAL13P1.180	conserved Plasmodium protein, unknown function	-4.20	0.0020	0.0055	0.0018	1.4	3848	1082	5432	1611	-1109
1810	PFE0405c	longevity-assurance (LAG1) domain protein, putative	-3.02	0.0020	0.0055	0.0018	1.3	3821	777	4836	1511	-1272
251	PFC0230c	conserved Plasmodium protein, unknown function	-6.01	0.0020	0.0055	0.0018	1.4	3819	701	5205	963	-279
972	PF14_0266	conserved protein, unknown function	-4.22	0.0020	0.0055	0.0018	1.4	3814	1320	5508	1626	-1252
278	MAL13P1.185	protein kinase 6	-5.84	0.0020	0.0055	0.0018	1.5	3812	959	5573	1239	-436
343	PF08_0001	Plasmodium exported protein, unknown function	-5.62	0.0020	0.0055	0.0018	1.9	3806	2206	7144	2244	-1112
360	PF14_0142	serine/threonine protein phosphatase	-5.56	0.0020	0.0055	0.0018	1.4	3806	763	5161	1006	-415
1015	PF07_0101	conserved Plasmodium protein, unknown function	-4.14	0.0020	0.0055	0.0018	1.3	3801	632	4928	1224	-729
1574	PFI0810c	apicoplast Ufd1 precursor	-3.30	0.0040	0.0098	0.0026	1.2	3781	846	4601	987	-1013
1395	PFI1340w	fumarate hydratase, putative	-3.55	0.0020	0.0055	0.0018	1.3	3780	743	5000	1567	-1089
876	PF07_0115	cation transporting ATPase, putative	-4.39	0.0020	0.0055	0.0018	1.4	3751	1017	5371	1592	-990
127	PFI1365w	cytochrome c oxidase subunit, putative	-6.82	0.0020	0.0055	0.0018	1.6	3749	980	6031	1418	-116
189	PF07_0087	conserved Plasmodium protein, unknown function	-6.36	0.0020	0.0055	0.0018	1.7	3727	1123	6277	1723	-296
1544	PF13_0182	ubiquitin-activating enzyme	-3.34	0.0060	0.0136	0.0036	1.3	3725	995	4890	1494	-1323
434	PFE0270c	DNA repair protein, putative	-5.35	0.0020	0.0055	0.0018	1.4	3696	697	5021	1063	-435
848	PF10_0318	conserved protein, unknown function	-4.44	0.0020	0.0055	0.0018	1.4	3691	1032	5017	1180	-886
559	PFL0495c	ABC transporter, (TAP family), putative	-5.04	0.0020	0.0055	0.0018	1.4	3691	736	5092	1215	-550
1281	PFB0926c	Plasmodium exported protein (hyp2), unknown function	-3.70	0.0020	0.0055	0.0018	1.4	3675	1204	5265	1843	-1457
664	MAL7P1.74	secreted ookinete protein, putative	-4.79	0.0020	0.0055	0.0018	1.5	3660	779	5394	1650	-695
554	PFI0410c	conserved Plasmodium protein, unknown function	-5.04	0.0020	0.0055	0.0018	1.5	3650	801	5315	1469	-606
588	PFF0640w	conserved Plasmodium protein, unknown function	-4.98	0.0020	0.0055	0.0018	1.5	3637	922	5467	1626	-719
506	PF14_0315	conserved Plasmodium membrane protein, unknown fun	-5.16	0.0020	0.0055	0.0018	1.7	3624	2110	6152	1617	-1200
1633	PFE0330w	conserved Plasmodium protein, unknown function	-3.23	0.0060	0.0136	0.0036	1.5	3619	1846	5286	1999	-2177
1980	PFC0485w	protein kinase, putative	-2.84	0.0040	0.0098	0.0026	1.3	3615	963	4664	1619	-1534
2339	PFI0145w	conserved protein, unknown function	-2.43	0.0180	0.0344	0.0090	1.3	3606	1188	4510	1522	-1806
1744	PFL1070c	endoplasmin homolog precursor, putative	-3.10	0.0100	0.0208	0.0055	1.6	3604	2292	5849	2979	-3026

617	PFC1030w	conserved Plasmodium protein, unknown function	-4.90	0.0020	0.0055	0.0018	1.4	3594	1446	5195	1040	-885
1724	PFL1045w	conserved protein, unknown function	-3.12	0.0060	0.0136	0.0036	1.3	3593	939	4518	1220	-1235
465	PF11_0507		-5.27	0.0020	0.0055	0.0018	1.8	3593	1263	6552	2538	-842
429	PF14_0647	conserved Plasmodium protein, unknown function	-5.39	0.0020	0.0055	0.0018	1.6	3584	1521	5651	1380	-833
91	PFF0715c	endonuclease III homologue, putative	-7.11	0.0020	0.0055	0.0018	1.6	3578	947	5641	1179	-63
266	PF14_0222	ankyrin, putative	-5.93	0.0020	0.0055	0.0018	1.5	3577	716	5490	1463	-266
213	PF08_0011	leucine-tRNA ligase	-6.21	0.0020	0.0055	0.0018	1.4	3576	665	4930	911	-221
1234	PF13_0097	transcription factor with AP2 domain(s), putative	-3.79	0.0020	0.0055	0.0018	1.6	3565	1582	5764	2516	-1899
293	PF08_0058	conserved Plasmodium protein, unknown function	-5.79	0.0020	0.0055	0.0018	1.5	3559	702	5384	1427	-302
1371	PFF0185c	conserved Plasmodium protein, unknown function	-3.58	0.0040	0.0098	0.0026	1.4	3553	726	4798	1589	-1070
1116	PFD0660w	phosphoglycerate mutase, putative	-3.99	0.0040	0.0098	0.0026	1.4	3549	926	4864	1416	-1026
593	PFD0065w	stevor, pseudogene	-4.97	0.0020	0.0055	0.0018	1.8	3543	1531	6481	2597	-1191
846	PFL1780w	protein-S-isoprenylcysteine O-methyltransferase, putativ	-4.44	0.0020	0.0055	0.0018	1.5	3540	871	5200	1681	-892
1811	PFC0556c	apicoplast conserved ycf19 protein precursor, unknown f	-3.02	0.0040	0.0098	0.0026	1.2	3537	650	4210	944	-921
2406	PF07_0094	conserved Plasmodium protein, unknown function	-2.36	0.0180	0.0344	0.0090	1.3	3534	943	4472	1781	-1786
381	MAL7P1.110	Ham1-like protein, putative	-5.50	0.0020	0.0055	0.0018	1.5	3509	881	5117	1226	-499
425	PF14_0471	transcription factor with AP2 domain(s), putative	-5.39	0.0020	0.0055	0.0018	1.7	3504	1257	5783	1782	-760
2240	PFL1895w	mitochondrial ribosomal protein L23 precursor, putative	-2.53	0.0200	0.0376	0.0099	1.2	3503	588	4034	903	-959
1083	PFE0145w	organelle ribosomal protein L28 precursor, putative	-4.04	0.0020	0.0055	0.0018	1.4	3500	756	4959	1651	-948
1458	MAL13P1.125	conserved Plasmodium protein, unknown function	-3.45	0.0020	0.0055	0.0018	1.4	3493	1000	4732	1546	-1307
838	PF11_0227	serine/threonine protein kinase, putative	-4.44	0.0020	0.0055	0.0018	1.4	3491	502	4815	1397	-575
2139	MAL8P1.136	conserved Plasmodium protein, unknown function	-2.65	0.0080	0.0173	0.0046	1.3	3491	722	4553	1865	-1525
1813	PFD0935c	conserved Plasmodium protein, unknown function	-3.02	0.0060	0.0136	0.0036	1.3	3491	655	4496	1536	-1186
154	PF14_0280	phosphotyrosyl phosphatase activator, putative	-6.61	0.0020	0.0055	0.0018	1.6	3489	784	5419	1272	-125
1532	PFF1275c	3-oxoacyl-acyl-carrier protein synthase I/II	-3.35	0.0060	0.0136	0.0036	1.3	3488	769	4688	1630	-1200
192	PFL1525c	pre-mRNA splicing factor RNA helicase, putative	-6.32	0.0020	0.0055	0.0018	1.6	3485	1170	5465	1179	-368
1164	PF14_0200	pantothenate kinase, putative	-3.90	0.0020	0.0055	0.0018	1.4	3484	1112	4787	1345	-1154
1330	PFI1440w	conserved Plasmodium protein, unknown function	-3.64	0.0020	0.0055	0.0018	1.4	3457	1004	4682	1415	-1194
2070	PFL2035c	conserved Plasmodium membrane protein, unknown fun	-2.74	0.0140	0.0277	0.0073	1.2	3440	904	4249	1233	-1328
1271	PF11_0527	conserved Plasmodium protein, unknown function	-3.73	0.0020	0.0055	0.0018	1.2	3435	892	4287	830	-871
2331	PFE1610w	Plasmodium exported protein, unknown function	-2.44	0.0200	0.0376	0.0099	1.4	3431	1253	4849	2644	-2478
1617	PF14_0386	adaptor complexes medium subunit family	-3.25	0.0060	0.0136	0.0036	1.6	3431	2203	5615	2724	-2743
1796	PFE0395c	6-cysteine protein	-3.04	0.0020	0.0055	0.0018	1.5	3430	1085	5060	2466	-1921
2167	PF11_0240	dynein heavy chain, putative	-2.61	0.0180	0.0344	0.0090	1.2	3427	987	4262	1329	-1480
1642	PF11_0333	conserved Plasmodium membrane protein, unknown fun	-3.22	0.0020	0.0055	0.0018	1.3	3425	766	4422	1371	-1141
903	PF07_0016	conserved Plasmodium membrane protein, unknown fun	-4.35	0.0020	0.0055	0.0018	1.5	3423	904	5076	1700	-950
236	PF11_0320	RNA-binding protein s1, putative	-6.08	0.0020	0.0055	0.0018	1.4	3415	587	4870	1064	-196
158	PFL1900w	transcription factor with AP2 domain(s), putative	-6.57	0.0020	0.0055	0.0018	1.7	3405	1040	5932	1671	-183
1948	PFL2460w	coronin	-2.87	0.0080	0.0173	0.0046	1.4	3392	1379	4698	1907	-1980
367	PF11_0328	conserved Plasmodium protein, unknown function	-5.55	0.0020	0.0055	0.0018	1.6	3384	891	5515	1725	-486
2453	PF10_0161	Plasmodium exported protein (PHISTc), unknown functio	-2.31	0.0220	0.0408	0.0107	1.3	3381	1101	4326	1783	-1939
636	MAL13P1.167	signal peptidase 21 kDa subunit	-4.86	0.0020	0.0055	0.0018	1.4	3379	617	4764	1298	-529
74	PFL0435w	conserved Plasmodium protein, unknown function	-7.30	0.0020	0.0055	0.0018	1.6	3373	1035	5353	1004	-59

1392	PF13_0230	conserved Plasmodium membrane protein, unknown fun	-3.55	0.0020	0.0055	0.0018	1.3	3370	757	4325	1154	-956
489	PF13_0029	conserved Plasmodium protein, unknown function	-5.21	0.0020	0.0055	0.0018	1.5	3359	855	5100	1471	-586
2382	PFI0785c	sugar transporter, putative	-2.39	0.0100	0.0208	0.0055	1.3	3356	1032	4307	1749	-1830
2247	PFI0675w	conserved Plasmodium protein, unknown function	-2.52	0.0160	0.0311	0.0082	1.4	3352	1799	4730	2207	-2628
472	MAL13P1.356	erythrocyte membrane protein 1, PfEMP1	-5.25	0.0020	0.0055	0.0018	1.4	3345	972	4807	1094	-604
1034	MAL8P1.66	conserved Plasmodium protein, unknown function	-4.12	0.0020	0.0055	0.0018	1.3	3340	672	4307	1003	-708
196	PF11_0485	mitochondrial ATP synthase delta subunit, putative	-6.29	0.0020	0.0055	0.0018	1.5	3339	755	4912	1047	-229
1239	MAL13P1.254	conserved Plasmodium protein, unknown function	-3.78	0.0020	0.0055	0.0018	1.3	3335	630	4307	1142	-800
185	PF14_0606	mitochondrial ribosomal protein S6-2 precursor, putative	-6.38	0.0020	0.0055	0.0018	1.7	3331	1014	5754	1653	-244
1040	PFD0775c	RNA binding protein, putative	-4.10	0.0020	0.0055	0.0018	1.4	3329	1006	4608	1273	-1000
337	PFE0830c	sporozoite surface antigen MB2	-5.66	0.0020	0.0055	0.0018	1.4	3324	809	4745	1026	-414
1495	PFF1175c	conserved Plasmodium protein, unknown function	-3.40	0.0020	0.0055	0.0018	1.3	3320	823	4380	1364	-1127
234	PF11_0327	oxysterol-binding protein-related protein 2	-6.09	0.0020	0.0055	0.0018	1.8	3307	1377	5897	1734	-521
415	PF14_0252	conserved Plasmodium protein, unknown function	-5.41	0.0020	0.0055	0.0018	1.5	3301	1093	5053	1293	-635
1054	PFE0025c	rifin	-4.08	0.0020	0.0055	0.0018	1.7	3283	1912	5480	2093	-1808
311	PFC0506w	conserved Plasmodium protein, unknown function	-5.75	0.0020	0.0055	0.0018	1.6	3274	996	5226	1438	-482
2322	PFL1320w	conserved Plasmodium protein, unknown function	-2.46	0.0160	0.0311	0.0082	1.2	3269	1010	3945	1047	-1381
1207	PFI0690c	conserved Plasmodium protein, unknown function	-3.83	0.0020	0.0055	0.0018	1.5	3257	1135	4729	1621	-1284
141	PFE1345c	minchromosome maintenance (MCM) complex subunit, p	-6.71	0.0020	0.0055	0.0018	1.7	3239	744	5461	1498	-20
1787	PFD0900w	conserved Plasmodium protein, unknown function	-3.05	0.0020	0.0055	0.0018	1.2	3231	711	4030	1136	-1048
1282	PF14_0154	conserved Plasmodium protein, unknown function	-3.70	0.0020	0.0055	0.0018	1.3	3231	938	4173	967	-962
685	PFB0161c	conserved Plasmodium protein, unknown function	-4.75	0.0020	0.0055	0.0018	1.4	3212	702	4641	1350	-622
1670	PFD0595c		-3.19	0.0040	0.0098	0.0026	1.5	3202	1626	4870	2172	-2129
239	PF11_0317	structural maintenance of chromosome protein, putative	-6.07	0.0020	0.0055	0.0018	1.6	3198	746	5076	1382	-249
646	PFL0595c	glutathione peroxidase	-4.83	0.0020	0.0055	0.0018	1.5	3193	1033	4716	1279	-789
1088	PF14_0265	peptide chain release factor 1, putative	-4.03	0.0020	0.0055	0.0018	1.3	3184	708	4128	980	-744
276	PF14_0712	conserved Plasmodium protein, unknown function	-5.85	0.0020	0.0055	0.0018	1.6	3183	875	5103	1430	-384
137	PFC0435w	parasite-infected erythrocyte surface protein	-6.72	0.0020	0.0055	0.0018	1.6	3176	1326	5240	1011	-273
438	PFF0115c	elongation factor G, putative	-5.34	0.0020	0.0055	0.0018	1.4	3176	874	4490	956	-516
1383	PF10_0070a	conserved Plasmodium protein, unknown function	-3.56	0.0020	0.0055	0.0018	1.3	3175	603	4232	1364	-910
1616	PFE0345c	conserved Plasmodium protein, unknown function	-3.25	0.0040	0.0098	0.0026	1.4	3162	1340	4556	1773	-1720
195	PFB0150c	protein kinase, putative	-6.30	0.0020	0.0055	0.0018	1.5	3161	755	4719	1034	-230
277	PF14_0161	conserved protein, unknown function	-5.84	0.0020	0.0055	0.0018	1.5	3160	685	4874	1316	-288
403	PF10_0246	conserved Plasmodium protein, unknown function	-5.46	0.0020	0.0055	0.0018	1.4	3149	780	4426	941	-444
72	PFE0325w		-7.31	0.0020	0.0055	0.0018	1.9	3148	1431	5911	1407	-75
806	MAL13P1.164	elongation factor Tu, putative	-4.51	0.0020	0.0055	0.0018	1.4	3148	770	4305	1078	-690
1885	PF10_0094	tubulin-tyrosine ligase, putative	-2.93	0.0040	0.0098	0.0026	1.4	3144	1244	4429	1873	-1833
304	PF10_0129	conserved Plasmodium protein, unknown function	-5.77	0.0020	0.0055	0.0018	1.7	3132	975	5294	1645	-457
584	MAL13P1.267	conserved Plasmodium protein, unknown function	-4.98	0.0020	0.0055	0.0018	1.4	3115	684	4478	1210	-531
1028	PF14_0470	DNA replication related protein, putative	-4.12	0.0020	0.0055	0.0018	1.4	3111	716	4283	1256	-801
1655	PFI0295c	RNB-like protein, putative	-3.20	0.0060	0.0136	0.0036	1.2	3095	887	3842	866	-1006
73	PFL1920c	hydroxyethylthiazole kinase, putative	-7.30	0.0020	0.0055	0.0018	1.6	3073	825	4878	993	-13
840	PFC0250c	AP endonuclease (DNA-[apurinic or apyrimidinic site] lyas	-4.44	0.0020	0.0055	0.0018	1.6	3072	794	4853	1847	-860

922	PFC0680w	conserved Plasmodium protein, unknown function	-4.32	0.0020	0.0055	0.0018	1.4	3048	689	4372	1385	-751
2214	PF14_0654	aminophospholipid transporter, putative	-2.56	0.0060	0.0136	0.0036	1.3	3033	969	3806	1235	-1431
912	PFI0465c	conserved Plasmodium protein, unknown function	-4.33	0.0020	0.0055	0.0018	1.4	3030	979	4114	909	-803
69	PF10_0343	S-antigen	-7.37	0.0020	0.0055	0.0018	2.1	3027	1635	6436	1804	-30
1990	PFF1285w	conserved Plasmodium protein, unknown function	-2.82	0.0120	0.0243	0.0064	1.3	3021	944	3779	1049	-1235
1119	PFC0495w	plasmepsin VI	-3.98	0.0020	0.0055	0.0018	1.5	3020	776	4654	1901	-1043
1887	PFI1465w	procollagen lysine 5-dioxygenase, putative	-2.93	0.0020	0.0055	0.0018	1.4	3020	954	4353	2076	-1698
1969	PFB0685c	acyl-CoA synthetase, PfACS9	-2.85	0.0100	0.0208	0.0055	1.4	3017	1536	4360	1912	-2106
579	PFI0910w	DNA helicase, putative	-4.99	0.0020	0.0055	0.0018	1.4	3014	805	4289	1055	-584
289	PFL1370w	NIMA related kinase 1	-5.81	0.0020	0.0055	0.0018	1.8	3008	1204	5322	1669	-559
2161	MAL13P1.121	adenosine-diphosphatase, putative	-2.62	0.0120	0.0243	0.0064	1.3	3008	779	3853	1438	-1372
436	PF07_0065	zinc transporter, putative	-5.35	0.0020	0.0055	0.0018	1.6	2996	850	4840	1532	-537
339	PF10_0120	ubiquinol-cytochrome c reductase complex subunit, puta	-5.64	0.0020	0.0055	0.0018	1.6	2989	783	4821	1449	-400
637	PF10_0247		-4.85	0.0020	0.0055	0.0018	1.5	2989	878	4615	1466	-718
1558	PF11_0117	replication factor C subunit 5, putative	-3.32	0.0040	0.0098	0.0026	1.4	2984	867	4188	1618	-1281
496	MAL13P1.332	conserved Plasmodium protein, unknown function	-5.19	0.0020	0.0055	0.0018	1.4	2983	476	4182	1059	-336
1999	MAL13P1.318	mitochondrial ribosomal protein L9 precursor, putative	-2.81	0.0060	0.0136	0.0036	1.3	2980	1025	3789	1117	-1332
660	PF14_0462	ubiquitin-protein ligase, putative	-4.80	0.0020	0.0055	0.0018	1.5	2975	800	4557	1470	-688
500	PF14_0488	conserved Plasmodium protein, unknown function	-5.18	0.0020	0.0055	0.0018	1.3	2971	491	3887	762	-337
2523	PF11_0530	conserved Plasmodium membrane protein, unknown fun	-2.23	0.0259	0.0469	0.0123	1.2	2971	748	3457	861	-1123
1364	MAL13P1.205	Rab GTPase 11b	-3.59	0.0020	0.0055	0.0018	1.4	2957	666	3996	1301	-928
1409	MAL8P1.25a	conserved Plasmodium protein, unknown function	-3.52	0.0060	0.0136	0.0036	1.3	2954	693	3737	920	-831
1753	MAL7P1.155	zinc finger, C3HC4 type, putative	-3.09	0.0060	0.0136	0.0036	1.3	2951	746	3713	1036	-1019
1947	PF08_0057	conserved Plasmodium protein, unknown function	-2.87	0.0060	0.0136	0.0036	1.3	2949	662	3879	1489	-1220
1883	PFI1805w	rifin	-2.94	0.0080	0.0173	0.0046	1.6	2944	2148	4604	2098	-2587
383	PF08_0097	conserved Plasmodium membrane protein, unknown fun	-5.49	0.0020	0.0055	0.0018	1.6	2938	747	4673	1415	-426
1410	PFD0865c	cdc2-related protein kinase 1	-3.52	0.0040	0.0098	0.0026	1.5	2919	1264	4497	1923	-1608
754	PF10_0329	plasmepsin VII	-4.62	0.0020	0.0055	0.0018	1.4	2896	512	4184	1297	-521
1978	PFL0375w	conserved Plasmodium protein, unknown function	-2.84	0.0080	0.0173	0.0046	1.2	2894	522	3505	958	-869
139	PFC10_API0030	null	-6.71	0.0020	0.0055	0.0018	1.7	2894	1102	4988	1217	-225
790	PFI0735c	type II NADH:quinone oxidoreductase	-4.55	0.0020	0.0055	0.0018	1.3	2886	597	3697	715	-501
1954	PF11_0285	conserved Plasmodium protein, unknown function	-2.86	0.0120	0.0243	0.0064	1.3	2882	1088	3818	1315	-1466
1044	PFI1810w	rifin	-4.10	0.0020	0.0055	0.0018	1.5	2881	1120	4297	1408	-1112
1734	PFA0630c	Plasmodium exported protein, unknown function	-3.11	0.0020	0.0055	0.0018	1.7	2867	1302	4808	2857	-2218
2185	PFE0215w	ATP-dependent helicase, putative	-2.60	0.0160	0.0311	0.0082	1.3	2861	743	3665	1379	-1318
443	MAL7P1.13	conserved Plasmodium protein, unknown function	-5.33	0.0020	0.0055	0.0018	1.5	2860	911	4378	1167	-560
256	PF13_0201	sporozoite surface protein 2	-6.00	0.0020	0.0055	0.0018	1.8	2850	1002	5079	1614	-387
1711	PF10_0009	stevor, pseudogene	-3.13	0.0040	0.0098	0.0026	1.4	2848	1519	4065	1413	-1715
951	PF14_0754	hypothetical protein	-4.26	0.0020	0.0055	0.0018	1.5	2845	720	4221	1464	-807
1448	MAL8P1.203	serine/threonine protein kinase, FIKK family	-3.46	0.0020	0.0055	0.0018	1.3	2841	665	3684	1054	-876
229	PF14_0328	mitochondrial import inner membrane translocase subur	-6.11	0.0020	0.0055	0.0018	1.6	2841	851	4544	1165	-312
371	PF13_0148	conserved Plasmodium protein, unknown function	-5.53	0.0020	0.0055	0.0018	1.5	2837	559	4120	1036	-311
780	PF07_0060	conserved Plasmodium protein, unknown function	-4.57	0.0020	0.0055	0.0018	1.5	2835	734	4192	1319	-696

1357	PF11_0076	conserved Plasmodium protein, unknown function	-3.61	0.0020	0.0055	0.0018	1.5	2834	1233	4120	1404	-1350
1264	MAL13P1.440		-3.74	0.0020	0.0055	0.0018	1.5	2830	1031	4300	1720	-1280
388	PFI1750c	Plasmodium exported protein (hyp11), unknown functior	-5.49	0.0020	0.0055	0.0018	1.8	2827	1451	5081	1602	-799
1855	PFE0140c	mitochondrial inner membrane TIM10 associated proteir	-2.97	0.0060	0.0136	0.0036	1.3	2820	948	3609	1029	-1188
986	PF10_0357	probable protein, unknown function	-4.19	0.0020	0.0055	0.0018	1.4	2818	895	3976	1128	-865
162	PF14_0382	stromal-processing peptidase, putative	-6.54	0.0020	0.0055	0.0018	1.5	2814	841	4243	804	-216
1716	MAL13P1.24	conserved Plasmodium protein, unknown function	-3.13	0.0080	0.0173	0.0046	1.4	2813	1155	3840	1284	-1413
2157	PFD0850c		-2.63	0.0080	0.0173	0.0046	1.3	2801	853	3663	1439	-1430
258	PF13_0054	conserved Plasmodium protein, unknown function	-5.98	0.0020	0.0055	0.0018	1.7	2792	846	4808	1490	-319
1333	PF10_0060	conserved Plasmodium protein, unknown function	-3.64	0.0020	0.0055	0.0018	1.3	2788	763	3747	1121	-925
2130	PFD0555c	conserved Plasmodium protein, unknown function	-2.66	0.0120	0.0243	0.0064	1.4	2784	747	3832	1826	-1525
166	PFA0225w	4-hydroxy-3-methylbut-2-enyl diphosphate reductase	-6.50	0.0020	0.0055	0.0018	1.8	2776	934	4963	1450	-197
2340	PF14_0454	conserved Plasmodium protein, unknown function	-2.43	0.0259	0.0469	0.0123	1.3	2770	1013	3533	1280	-1530
223	MAL13P1.141	conserved Plasmodium protein, unknown function	-6.15	0.0020	0.0055	0.0018	1.6	2770	677	4327	1103	-222
119	PF11_0455	conserved Plasmodium protein, unknown function	-6.88	0.0020	0.0055	0.0018	1.8	2760	829	5019	1450	-20
2537	PF14_0329	conserved protein, unknown function	-2.22	0.0259	0.0469	0.0123	1.4	2751	1168	3885	2298	-2333
1580	MAL7P1.100	NIMA related kinase 4	-3.29	0.0020	0.0055	0.0018	1.4	2742	546	3814	1527	-1001
2326	PF08_0082	conserved Plasmodium protein, unknown function	-2.45	0.0120	0.0243	0.0064	1.3	2734	648	3584	1609	-1406
594	PFI1630c	conserved Plasmodium protein, unknown function	-4.96	0.0020	0.0055	0.0018	1.7	2729	943	4594	1662	-739
1056	PF10_0050	conserved Plasmodium protein, unknown function	-4.08	0.0020	0.0055	0.0018	1.4	2729	526	3762	1160	-652
2217	PFL1550w	lipoamide dehydrogenase	-2.56	0.0120	0.0243	0.0064	1.3	2724	813	3411	1124	-1250
949	PF10_0177		-4.26	0.0020	0.0055	0.0018	1.5	2715	994	4176	1457	-990
1770	PFI0975c	conserved Plasmodium protein, unknown function	-3.07	0.0040	0.0098	0.0026	1.5	2711	926	3958	1831	-1509
115	PF11_0075	conserved Plasmodium protein, unknown function	-6.92	0.0020	0.0055	0.0018	2.1	2707	1243	5622	1782	-109
179	PFF0740c	conserved Plasmodium membrane protein, unknown fun	-6.41	0.0020	0.0055	0.0018	1.7	2704	608	4504	1278	-86
441	MAL8P1.27	translation initiation factor IF-3, putative	-5.34	0.0020	0.0055	0.0018	1.5	2700	1010	4081	942	-571
1257	PFB0750w	vacuolar protein-sorting protein VPS45, putative	-3.75	0.0020	0.0055	0.0018	1.4	2697	745	3707	1160	-895
1421	PFC0320w	conserved Plasmodium protein, unknown function	-3.50	0.0020	0.0055	0.0018	1.4	2697	730	3714	1288	-1001
1533	PFF0950w	conserved Plasmodium protein, unknown function	-3.35	0.0020	0.0055	0.0018	1.3	2696	679	3543	1101	-933
1605	PF10_0144	prohibitin, putative	-3.26	0.0020	0.0055	0.0018	1.6	2692	1294	4370	2272	-1889
1781	PF14_0497	aminomethyltransferase, putative	-3.06	0.0020	0.0055	0.0018	1.3	2685	735	3582	1297	-1135
2211	PFI1275w	protein kinase, putative	-2.56	0.0140	0.0277	0.0073	1.3	2680	1034	3426	1129	-1418
564	PFA0700c	Plasmodium exported protein (hyp10), unknown functior	-5.03	0.0020	0.0055	0.0018	2.4	2676	2604	6505	3024	-1799
1473	PFF0770c	conserved Plasmodium protein, unknown function	-3.42	0.0020	0.0055	0.0018	1.5	2671	1368	4047	1604	-1596
1292	PFF0775w	pyridoxine kinase	-3.69	0.0020	0.0055	0.0018	1.5	2666	1027	3915	1417	-1195
384	PFD1035w	steroid dehydrogenase, putative	-5.49	0.0020	0.0055	0.0018	2.0	2658	1479	5389	2095	-843
2170	PFF0260w	serine/threonine protein kinase, putative	-2.61	0.0080	0.0173	0.0046	1.3	2658	726	3391	1232	-1225
2013	PFF0720w	conserved Plasmodium membrane protein, unknown fun	-2.79	0.0140	0.0277	0.0073	1.4	2643	933	3767	1808	-1617
1923	PFF1470c	DNA polymerase epsilon, catalytic subunit a, putative	-2.90	0.0020	0.0055	0.0018	1.3	2634	723	3324	996	-1029
2544	PFA0440w	photosensitized INA-labeled protein 1, PhIL1, putative	-2.21	0.0220	0.0408	0.0107	1.4	2627	1296	3611	1891	-2204
941	MAL13P1.191	conserved Plasmodium protein, unknown function	-4.28	0.0020	0.0055	0.0018	1.5	2621	766	3883	1296	-799
2116	PFE1065w	conserved Plasmodium protein, unknown function	-2.67	0.0100	0.0208	0.0055	1.3	2613	703	3467	1450	-1298
2284	PFL0305c	IMP-specific 5'-nucleotidase, putative,haloacid dehaloger	-2.50	0.0200	0.0376	0.0099	1.3	2612	738	3296	1190	-1244

2272	PF10_0358	conserved Plasmodium protein, unknown function	-2.50	0.0180	0.0344	0.0090	1.2	2608	662	3237	1098	-1131
548	PF14_0031	conserved Plasmodium protein, unknown function	-5.06	0.0020	0.0055	0.0018	1.6	2602	686	4109	1342	-521
1697	PF11_0014	Plasmodium falciparum Maurer's Cleft 2 transmembrane	-3.15	0.0100	0.0208	0.0055	1.8	2599	2198	4584	2474	-2687
1868	PFB0530c	conserved Plasmodium protein, unknown function	-2.95	0.0020	0.0055	0.0018	1.3	2596	913	3366	1020	-1164
766	PF14_0367	conserved Plasmodium protein, unknown function	-4.60	0.0020	0.0055	0.0018	1.7	2592	947	4518	1893	-913
696	PFC0275w	FAD-dependent glycerol-3-phosphate dehydrogenase, pu	-4.74	0.0020	0.0055	0.0018	1.6	2588	918	4221	1503	-787
1582	PF14_0409	conserved Plasmodium protein, unknown function	-3.29	0.0020	0.0055	0.0018	1.5	2586	1230	3827	1531	-1520
501	PF14_0666	conserved Plasmodium protein, unknown function	-5.18	0.0020	0.0055	0.0018	1.5	2585	839	3931	1062	-554
301	PFF0785w	Ndc80 homologue, putative	-5.78	0.0020	0.0055	0.0018	1.5	2578	771	3853	866	-362
2495	MAL7P1.203b	conserved Plasmodium protein, unknown function, fragm	-2.26	0.0259	0.0469	0.0123	1.2	2577	849	3176	1083	-1333
2388	PF14_0658	translation initiation factor EF-1, putative	-2.37	0.0220	0.0408	0.0107	1.3	2576	970	3425	1550	-1671
1105	PFD1085w	conserved Plasmodium protein, unknown function	-4.01	0.0020	0.0055	0.0018	1.7	2563	1086	4275	1883	-1257
232	PFC0210c	circumsporozoite (CS) protein	-6.10	0.0020	0.0055	0.0018	1.9	2560	1152	4762	1481	-431
2172	PFL0840c	conserved Plasmodium protein, unknown function	-2.61	0.0120	0.0243	0.0064	1.2	2558	644	3098	856	-960
2287	PFE0700c	tubulin-tyrosine ligase, putative	-2.49	0.0140	0.0277	0.0073	1.3	2557	525	3212	1208	-1078
1941	MAL13P1.183	conserved Plasmodium protein, unknown function	-2.88	0.0060	0.0136	0.0036	1.3	2555	952	3261	899	-1145
1850	PFL0050c	Plasmodium exported protein (PHISTb), unknown functio	-2.97	0.0040	0.0098	0.0026	1.8	2548	1174	4502	3065	-2284
873	PF13_0070	branched-chain alpha keto-acid dehydrogenase, putative	-4.40	0.0020	0.0055	0.0018	1.6	2542	1257	3962	1178	-1015
566	PF14_0577	conserved Plasmodium protein, unknown function	-5.01	0.0020	0.0055	0.0018	1.7	2540	805	4205	1480	-619
95	PFL2100w	ubiquitin conjugating enzyme E2, putative	-7.08	0.0020	0.0055	0.0018	1.8	2537	951	4481	1082	-89
495	PFI1290w		-5.20	0.0020	0.0055	0.0018	1.8	2536	943	4491	1664	-652
856	PFE0890c	conserved Plasmodium protein, unknown function	-4.42	0.0020	0.0055	0.0018	1.7	2532	895	4378	1900	-950
2428	MAL13P1.102	conserved Plasmodium protein, unknown function	-2.33	0.0160	0.0311	0.0082	1.4	2527	689	3507	1980	-1689
942	PFD1220c	stevor	-4.28	0.0020	0.0055	0.0018	1.8	2516	1546	4594	1996	-1464
2578	PF11_0307	phosphatidylinositol-4-phosphate-5-kinase, putative	-2.17	0.0240	0.0438	0.0115	1.4	2511	743	3414	1939	-1779
464	PF14_0705	conserved Plasmodium protein, unknown function	-5.27	0.0020	0.0055	0.0018	1.8	2507	707	4406	1662	-469
2290	PF08_0022	conserved Plasmodium protein, unknown function	-2.49	0.0080	0.0173	0.0046	1.2	2502	859	3101	931	-1191
1092	PF14_0502	U1 snRNA associated protein, putative	-4.03	0.0020	0.0055	0.0018	1.6	2497	657	3930	1653	-876
406	PFF1165c	conserved Plasmodium protein, unknown function	-5.44	0.0020	0.0055	0.0018	1.6	2497	665	3912	1144	-394
124	MAL7P1.223	stevor	-6.85	0.0020	0.0055	0.0018	2.1	2491	1062	5148	1678	-83
76	PF10_0404	rifin	-7.29	0.0020	0.0055	0.0018	2.4	2486	1762	5883	1738	-103
1384	PFE1170w	DnaJ protein, putative	-3.56	0.0020	0.0055	0.0018	1.4	2474	922	3377	972	-991
1723	PFF0355c	conserved Plasmodium membrane protein, unknown fun	-3.12	0.0020	0.0055	0.0018	1.4	2474	835	3405	1284	-1188
927	PFL2340w	conserved Plasmodium protein, unknown function	-4.31	0.0020	0.0055	0.0018	1.6	2472	1084	3870	1304	-989
926	PFA0390w	DNA repair exonuclease Mre11, putative	-4.31	0.0020	0.0055	0.0018	1.5	2469	865	3780	1300	-855
2473	PF13_0283	conserved Plasmodium protein, unknown function	-2.29	0.0120	0.0243	0.0064	1.3	2469	621	3099	1243	-1234
2150	PF11_0387		-2.63	0.0120	0.0243	0.0064	1.5	2467	1086	3757	2219	-2015
1758	PFB0185w	conserved Plasmodium protein, unknown function	-3.08	0.0020	0.0055	0.0018	1.4	2464	945	3389	1238	-1258
1960	PFL0655w	conserved Plasmodium membrane protein, unknown fun	-2.86	0.0060	0.0136	0.0036	1.4	2460	561	3409	1558	-1169
342	PFA0725w	surface-associated interspersed protein 1.3 (SURFIN 1.3)	-5.62	0.0020	0.0055	0.0018	1.8	2448	1068	4416	1462	-562
1747	PFB0795w	ATP synthase F1, alpha subunit, putative	-3.10	0.0020	0.0055	0.0018	1.5	2447	1042	3678	1737	-1548
474	PF14_0693	conserved Plasmodium protein, unknown function	-5.24	0.0020	0.0055	0.0018	1.7	2442	883	4078	1336	-584
2444	PF13_0266	conserved Plasmodium protein, unknown function	-2.31	0.0160	0.0311	0.0082	1.4	2441	616	3300	1743	-1500

633	PFF0175c	conserved Plasmodium protein, unknown function	-4.87	0.0020	0.0055	0.0018	1.8	2433	1096	4377	1727	-879
746	PFA0365c	hypothetical protein conserved in P. falciparum	-4.64	0.0020	0.0055	0.0018	1.6	2429	742	3770	1272	-673
821	PF14_0468a	conserved Plasmodium protein, unknown function	-4.48	0.0020	0.0055	0.0018	1.8	2427	1434	4372	1752	-1241
936	PFL0480w	porphobilinogen deaminase	-4.29	0.0020	0.0055	0.0018	1.4	2417	672	3385	953	-656
291	PF11_0477	CCAAT-box DNA binding protein subunit B	-5.80	0.0020	0.0055	0.0018	1.7	2414	964	4108	1180	-450
1848	PF13_0361	conserved Plasmodium protein, unknown function	-2.98	0.0060	0.0136	0.0036	1.4	2412	754	3306	1327	-1188
493	MAL8P1.1	surface-associated interspersed gene 8.1, (SURFIN8.1)	-5.20	0.0020	0.0055	0.0018	1.8	2411	780	4250	1605	-545
2081	PF11_0094	conserved Plasmodium protein, unknown function	-2.72	0.0080	0.0173	0.0046	1.3	2407	1040	3121	940	-1266
1479	PFF0465c	mitochondrial cardiolipin synthase, PfCLS	-3.41	0.0040	0.0098	0.0026	1.5	2391	1140	3499	1271	-1304
437	PFA0745w	rifin	-5.34	0.0020	0.0055	0.0018	2.0	2390	1265	4794	1933	-793
2274	PFE0695w	conserved Plasmodium protein, unknown function	-2.50	0.0120	0.0243	0.0064	1.3	2389	623	3094	1278	-1195
1146	PFL1865w	conserved Plasmodium protein, unknown function	-3.94	0.0020	0.0055	0.0018	1.7	2363	983	4002	1865	-1209
1223	PF14_0053	ribonucleotide reductase small subunit	-3.81	0.0020	0.0055	0.0018	1.6	2350	663	3817	1802	-999
372	PFB0345c	serine repeat antigen 4	-5.52	0.0020	0.0055	0.0018	2.0	2350	1306	4780	1857	-732
557	PF14_0020	choline kinase	-5.04	0.0020	0.0055	0.0018	2.1	2348	1640	4968	2144	-1164
1793	PFL0865w	conserved protein, unknown function	-3.04	0.0100	0.0208	0.0055	1.6	2344	1607	3708	1733	-1976
1943	PFF0085w	Plasmodium exported protein (PHISTa), unknown functio	-2.87	0.0100	0.0208	0.0055	1.7	2331	1686	4063	2594	-2547
126	PF14_0233	conserved Plasmodium protein, unknown function	-6.82	0.0020	0.0055	0.0018	1.6	2330	548	3807	956	-26
2103	MAL13P1.301	guanylyl cyclase beta	-2.70	0.0140	0.0277	0.0073	1.3	2327	788	2930	872	-1057
2308	MAL7P1.29	conserved Plasmodium membrane protein, unknown fun	-2.47	0.0160	0.0311	0.0082	1.3	2325	766	3028	1238	-1301
2226	PFL0390c	conserved Plasmodium protein, unknown function	-2.55	0.0080	0.0173	0.0046	1.4	2313	1142	3337	1716	-1834
2411	PF14_0310	conserved Plasmodium protein, unknown function	-2.35	0.0200	0.0376	0.0099	1.4	2311	1072	3324	1913	-1970
2262	PFC0165w	spindle pole body protein, putative	-2.51	0.0100	0.0208	0.0055	1.2	2297	694	2712	562	-841
1296	PFF0425w	conserved Plasmodium protein, unknown function	-3.68	0.0020	0.0055	0.0018	1.6	2296	710	3705	1777	-1078
1208	PF10_0407	dihydrolipoamide acyltransferase component E2	-3.83	0.0020	0.0055	0.0018	1.4	2291	845	3202	923	-858
1976	PFL0610w	conserved Plasmodium protein, unknown function	-2.84	0.0040	0.0098	0.0026	1.3	2287	694	2936	954	-999
221	PFI0380c	peptidyl deformylase	-6.15	0.0020	0.0055	0.0018	1.9	2285	948	4267	1362	-328
1420	PF13_0095	minchromosome maintenance (MCM) complex subunit, p	-3.50	0.0020	0.0055	0.0018	1.6	2283	763	3583	1704	-1167
1430	PF11_0440	conserved Plasmodium protein, unknown function	-3.48	0.0020	0.0055	0.0018	1.4	2283	915	3121	894	-971
107	PFA0345w		-7.02	0.0020	0.0055	0.0018	1.8	2282	781	4143	1121	-41
328	PFD0705c	conserved protein, unknown function	-5.67	0.0020	0.0055	0.0018	1.5	2279	608	3517	939	-309
1490	PF11_0473	conserved Plasmodium protein, unknown function	-3.40	0.0020	0.0055	0.0018	1.4	2277	882	3169	1050	-1041
1374	PF14_0586	conserved Plasmodium protein, unknown function	-3.58	0.0020	0.0055	0.0018	1.7	2248	749	3757	1969	-1208
1589	PF08_0100	ruvB-like DNA helicase, putative	-3.28	0.0020	0.0055	0.0018	1.4	2245	445	3151	1300	-839
409	PF11_0195	conserved Plasmodium protein, unknown function	-5.43	0.0020	0.0055	0.0018	1.6	2233	600	3564	1091	-360
191	PF14_0747	surface -associated intersprsed gene 14.1 (SURFIN 14.1)	-6.34	0.0020	0.0055	0.0018	2.3	2229	1210	5059	1937	-318
1893	MAL13P1.68	peptidyl-prolyl cis-trans isomerase, putative	-2.93	0.0060	0.0136	0.0036	1.3	2221	804	2944	1003	-1084
603	PFF0040c	rifin, pseudogene	-4.95	0.0020	0.0055	0.0018	2.0	2209	1732	4485	1721	-1177
971	PFL0910c		-4.22	0.0020	0.0055	0.0018	1.6	2205	866	3480	1291	-881
1816	PFI0770c	conserved Plasmodium protein, unknown function	-3.01	0.0020	0.0055	0.0018	1.3	2204	737	2895	938	-984
1795	MAL8P1.74	conserved Plasmodium protein, unknown function	-3.04	0.0040	0.0098	0.0026	1.2	2196	434	2661	655	-624
1927	PFL0215c		-2.89	0.0060	0.0136	0.0036	1.6	2196	837	3522	2137	-1648
325	PFA0280w	asparagine-rich antigen Pfa35-2	-5.69	0.0020	0.0055	0.0018	1.7	2191	803	3643	1053	-404

1570	MAL7P1.11	conserved Plasmodium protein, unknown function	-3.30	0.0040	0.0098	0.0026	1.3	2186	459	2842	893	-696
1460	PF11_0222	WD domain, G-beta repeat-containing protein	-3.44	0.0020	0.0055	0.0018	1.5	2178	636	3168	1303	-950
964	PFD0805w	prohibitin-like protein, putative	-4.23	0.0020	0.0055	0.0018	1.5	2170	582	3248	1149	-653
2298	MAL13P1.139	conserved Plasmodium protein, unknown function	-2.48	0.0100	0.0208	0.0055	1.3	2162	616	2768	1080	-1090
1542	PF10_0347	merozoite surface protein	-3.34	0.0040	0.0098	0.0026	1.4	2162	602	3044	1191	-912
361	PF11_0253	conserved Plasmodium protein, unknown function	-5.56	0.0020	0.0055	0.0018	1.7	2149	906	3672	1105	-488
1590	PF14_0325	conserved Plasmodium membrane protein, unknown fun	-3.28	0.0020	0.0055	0.0018	1.6	2124	1019	3456	1794	-1481
413	PF07_0038	Cg7 protein	-5.41	0.0020	0.0055	0.0018	1.6	2124	700	3342	933	-415
662	MAL13P1.327	mitochondrial ribosomal protein S17 precursor, putative	-4.79	0.0020	0.0055	0.0018	1.7	2122	979	3505	1150	-746
116	PF07_0119	conserved Plasmodium protein, unknown function	-6.91	0.0020	0.0055	0.0018	1.9	2120	689	4031	1225	-4
2109	PF10_0342	probable protein, unknown function	-2.69	0.0020	0.0055	0.0018	1.4	2119	768	3029	1528	-1386
1452	PF14_0544	conserved Plasmodium protein, unknown function	-3.46	0.0040	0.0098	0.0026	2.1	2117	1909	4410	2825	-2441
2424	PF10_0314a	conserved Plasmodium protein, unknown function	-2.33	0.0240	0.0438	0.0115	1.3	2117	739	2776	1238	-1317
1933	PFI1210w	conserved Plasmodium protein, unknown function	-2.88	0.0060	0.0136	0.0036	1.6	2103	650	3266	1900	-1387
546	PF10_0047	RNA binding protein, putative	-5.06	0.0020	0.0055	0.0018	2.4	2102	1555	5097	2591	-1151
1730	PFA0510w	bromodomain protein, putative	-3.11	0.0080	0.0173	0.0046	1.4	2096	854	2982	1196	-1164
2555	PF14_0345		-2.20	0.0259	0.0469	0.0123	1.4	2088	1340	2992	1669	-2105
416	PFB0615c	conserved Plasmodium protein, unknown function	-5.41	0.0020	0.0055	0.0018	1.6	2081	546	3319	1024	-332
149	PFL0465c	zinc finger transcription factor (krox1)	-6.64	0.0020	0.0055	0.0018	1.8	2080	818	3824	1087	-161
498	PFC1095w	rifin (3D7-rifT3-5)	-5.18	0.0020	0.0055	0.0018	2.1	2077	1548	4306	1658	-977
2577	PF13_0168	CPW-WPC family protein	-2.17	0.0140	0.0277	0.0073	1.5	2077	625	3053	2140	-1788
1090	PFE1510c	triose phosphate transporter	-4.03	0.0020	0.0055	0.0018	1.6	2063	702	3234	1294	-826
2426	PF11_0258	co-chaperone GrpE, putative	-2.33	0.0220	0.0408	0.0107	1.4	2063	1031	2891	1508	-1711
585	PF14_0597	cytochrome c1 precursor, putative	-4.98	0.0020	0.0055	0.0018	1.8	2058	772	3672	1448	-607
2324	PFI0940c	PPPDE peptidase, putative	-2.45	0.0180	0.0344	0.0090	1.4	2051	1176	2943	1482	-1766
1106	PF14_0396	conserved Plasmodium protein, unknown function	-4.01	0.0020	0.0055	0.0018	1.6	2049	575	3264	1406	-765
577	PFI1265w	conserved Plasmodium protein, unknown function	-4.99	0.0020	0.0055	0.0018	1.7	2047	951	3517	1200	-681
2318	PF14_0130	conserved Plasmodium protein, unknown function	-2.46	0.0160	0.0311	0.0082	1.2	2041	567	2512	809	-905
102	PF13_0221	conserved Plasmodium protein, unknown function	-7.05	0.0020	0.0055	0.0018	1.9	2040	844	3857	1045	-73
1977	PF11_0264	DNA-dependent RNA polymerase	-2.84	0.0100	0.0208	0.0055	1.4	2037	912	2757	976	-1168
1598	MAL8P1.76	meiotic recombination protein dmc1-like protein	-3.27	0.0020	0.0055	0.0018	1.9	2033	1189	3951	2693	-1965
1581	PFA0535c	kinesin, putative	-3.29	0.0020	0.0055	0.0018	1.4	2021	774	2928	1182	-1050
322	PFD0645w	rifin	-5.69	0.0020	0.0055	0.0018	1.7	2012	1061	3468	880	-486
1545	PF14_0412	conserved Plasmodium protein, unknown function	-3.33	0.0040	0.0098	0.0026	1.6	2010	1105	3241	1552	-1425
423	PF13_0234	phosphoenolpyruvate carboxykinase	-5.39	0.0020	0.0055	0.0018	1.7	2003	526	3329	1121	-321
319	PF08_0014	apicoplast ribosomal protein L21 precursor, putative	-5.70	0.0020	0.0055	0.0018	1.7	2002	589	3350	1047	-288
527	PF14_0548	ATPase Vps4, putative	-5.10	0.0020	0.0055	0.0018	1.7	2000	454	3309	1199	-344
401	PF14_0538	conserved Plasmodium protein, unknown function	-5.46	0.0020	0.0055	0.0018	1.8	1992	681	3611	1335	-397
650	PFL1785c	conserved Plasmodium protein, unknown function	-4.82	0.0020	0.0055	0.0018	1.9	1985	826	3698	1596	-709
2480	PFI0185w	LCCL domain-containing protein	-2.28	0.0200	0.0376	0.0099	1.7	1959	734	3370	2972	-2295
2184	PFD0295c	apical sushi protein	-2.60	0.0080	0.0173	0.0046	1.5	1949	860	2872	1581	-1518
788	PF14_0007	stevor	-4.55	0.0020	0.0055	0.0018	1.9	1946	1526	3709	1399	-1161
1846	PFL0015c	rifin	-2.98	0.0060	0.0136	0.0036	1.5	1941	1437	2997	1249	-1630

533	PF11_0074	exonuclease, putative	-5.08	0.0020	0.0055	0.0018	1.9	1936	669	3637	1540	-508
923	PF11_0186	conserved Plasmodium protein, unknown function	-4.32	0.0020	0.0055	0.0018	1.6	1930	901	3154	1164	-841
378	PFI1355w	conserved Plasmodium protein, unknown function	-5.50	0.0020	0.0055	0.0018	1.7	1921	630	3289	1097	-359
467	MAL13P1.20	conserved Plasmodium protein, unknown function	-5.27	0.0020	0.0055	0.0018	1.8	1918	568	3511	1403	-378
1337	MAL8P1.106		-3.64	0.0020	0.0055	0.0018	1.8	1916	1020	3404	1812	-1344
799	PF14_0376	leucine carboxyl methyltransferase, putative	-4.52	0.0020	0.0055	0.0018	1.6	1913	688	2971	991	-621
238	PFE0320w	conserved Plasmodium protein, unknown function	-6.07	0.0020	0.0055	0.0018	2.1	1906	1115	4032	1439	-427
448	PFC0030c	rifin	-5.32	0.0020	0.0055	0.0018	2.2	1902	1474	4190	1704	-890
531	PF10_0397	rifin	-5.09	0.0020	0.0055	0.0018	2.2	1899	1347	4175	1875	-946
2200	PF10_0253	conserved Plasmodium protein, unknown function	-2.58	0.0180	0.0344	0.0090	1.4	1888	829	2568	1087	-1236
430	PFF1170w	conserved Plasmodium protein, unknown function	-5.38	0.0020	0.0055	0.0018	2.2	1881	493	4116	1994	-252
2663	MAL8P1.15	conserved Plasmodium protein, unknown function	-2.07	0.0240	0.0438	0.0115	1.3	1880	557	2414	1171	-1194
348	PFL1460c	conserved Plasmodium protein, unknown function	-5.60	0.0020	0.0055	0.0018	1.9	1880	668	3484	1287	-351
2502	PFF0570c	conserved Plasmodium protein, unknown function	-2.26	0.0240	0.0438	0.0115	1.6	1879	1003	3020	2329	-2191
168	PF10_0191	tRNA methyltransferase, putative	-6.49	0.0020	0.0055	0.0018	2.1	1870	761	3884	1380	-127
1304	MAL7P1.77	conserved Plasmodium protein, unknown function	-3.67	0.0040	0.0098	0.0026	2.2	1868	1204	4020	2685	-1737
265	PF11_0213	conserved Plasmodium protein, unknown function	-5.93	0.0020	0.0055	0.0018	1.9	1859	614	3601	1344	-216
1027	PFA0285c	conserved Plasmodium protein, unknown function	-4.12	0.0020	0.0055	0.0018	1.7	1854	787	3098	1321	-865
86	PFD0470c	replication protein A large subunit	-7.17	0.0020	0.0055	0.0018	2.4	1837	1032	4353	1484	0
2194	PFI1380c	conserved Plasmodium protein, unknown function	-2.59	0.0080	0.0173	0.0046	1.6	1834	871	2906	1895	-1694
1991	PF11_0135	conserved Plasmodium protein, unknown function	-2.82	0.0060	0.0136	0.0036	1.4	1833	760	2600	1170	-1162
1396	PF14_0399	ADP-ribosylation factor, putative	-3.55	0.0040	0.0098	0.0026	1.7	1832	1127	3109	1489	-1339
2015	PF13_0185	histone H3 variant, putative	-2.79	0.0080	0.0173	0.0046	1.3	1831	647	2331	687	-835
793	PFE0970w	cytochrome c oxidase assembly protein (heme A: farnesy	-4.54	0.0020	0.0055	0.0018	1.8	1812	548	3176	1398	-582
1727	PFI0725c	GINS complex subunit Psf3, putative	-3.11	0.0040	0.0098	0.0026	1.4	1806	687	2498	923	-918
306	MAL8P1.330		-5.76	0.0020	0.0055	0.0018	2.1	1803	1021	3701	1367	-490
2503	PFL2070w	syntaxin, Qa-SNARE family	-2.25	0.0240	0.0438	0.0115	1.2	1803	480	2202	767	-849
2294	MAL7P1.123	conserved Plasmodium protein, unknown function	-2.49	0.0100	0.0208	0.0055	1.4	1802	480	2570	1459	-1171
1596	PF10_0051	ADP/ATP carrier protein, putative	-3.27	0.0020	0.0055	0.0018	1.5	1797	999	2690	1040	-1147
1572	PFE0825w	metabolite/drug transporter, putative	-3.30	0.0020	0.0055	0.0018	2.0	1797	871	3540	2480	-1608
2514	MAL8P1.77	conserved Plasmodium protein, unknown function	-2.25	0.0259	0.0469	0.0123	1.4	1792	875	2420	1156	-1403
252	PF14_0063	ATP-dependent Clp protease, putative	-6.01	0.0020	0.0055	0.0018	1.9	1787	514	3328	1179	-152
656	PFA0360c	hypothetical protein conserved in P. falciparum	-4.81	0.0020	0.0055	0.0018	1.7	1772	558	2996	1156	-490
1431	PF10_0257	conserved Plasmodium protein, unknown function	-3.48	0.0020	0.0055	0.0018	2.2	1767	2012	3961	2582	-2401
1921	PF08_0038	mitochondrial ribosomal protein L4 precursor, putative	-2.90	0.0060	0.0136	0.0036	1.5	1767	789	2587	1215	-1185
487	PFL1750c	conserved Plasmodium protein, unknown function	-5.21	0.0020	0.0055	0.0018	2.3	1762	1400	4091	1845	-917
2204	PF14_0248	ubiquinol-cytochrome c reductase hinge protein, putative	-2.58	0.0100	0.0208	0.0055	1.4	1750	986	2505	1174	-1405
717	PFL1526c	conserved Plasmodium protein, unknown function	-4.69	0.0020	0.0055	0.0018	1.6	1747	663	2737	870	-544
2133	PFA0100c	Plasmodium exported protein (PHISTa), unknown functio	-2.66	0.0160	0.0311	0.0082	1.5	1744	1053	2553	1203	-1446
730	PF10_0052	conserved Plasmodium protein, unknown function	-4.68	0.0020	0.0055	0.0018	1.7	1742	504	2899	1134	-482
1911	PF14_0726	conserved Plasmodium protein, unknown function	-2.91	0.0060	0.0136	0.0036	1.4	1710	745	2453	1083	-1085
1190	MAL13P1.328	DNA topoisomerase VI, b subunit, putative	-3.86	0.0020	0.0055	0.0018	1.8	1708	768	3070	1605	-1010
2118	PFI1455c	conserved Plasmodium protein, unknown function	-2.67	0.0040	0.0098	0.0026	1.6	1704	600	2701	1758	-1360

488	PFB0220w	ubiE/COQ5 methyltransferase, putative	-5.21	0.0020	0.0055	0.0018	1.5	1698	319	2581	786	-222
295	MAL13P1.40	conserved Plasmodium protein, unknown function	-5.79	0.0020	0.0055	0.0018	1.7	1695	583	2898	892	-272
2527	PF14_0356	conserved Plasmodium protein, unknown function	-2.23	0.0220	0.0408	0.0107	1.2	1694	554	2113	795	-930
1964	PF11_0216	heat shock factor binding protein 1, putative	-2.85	0.0040	0.0098	0.0026	1.5	1692	681	2468	1202	-1108
270	PFC0910w	conserved Plasmodium protein, unknown function	-5.87	0.0020	0.0055	0.0018	2.6	1650	1047	4258	1989	-429
1202	PFE1615c	Plasmodium exported protein, unknown function	-3.84	0.0020	0.0055	0.0018	2.6	1649	1393	4310	3188	-1920
160	PF14_0090	DNA-damage inducible protein, putative	-6.55	0.0020	0.0055	0.0018	2.7	1644	976	4382	1876	-114
1140	PFL2605w	rifin	-3.94	0.0040	0.0098	0.0026	1.8	1643	1351	2971	1198	-1221
1451	MAL13P1.131	conserved Plasmodium membrane protein, unknown fun	-3.46	0.0040	0.0098	0.0026	1.4	1642	548	2311	829	-708
979	PF14_0792	conserved Plasmodium protein, unknown function	-4.20	0.0020	0.0055	0.0018	2.1	1638	573	3441	2046	-816
1255	PF14_0455	multidrug resistance protein 2 (heavy metal transport far	-3.76	0.0020	0.0055	0.0018	2.4	1629	1290	3934	2800	-1786
2001	PF10_0190	conserved Plasmodium protein, unknown function	-2.81	0.0020	0.0055	0.0018	1.4	1618	606	2197	872	-898
816	MAL8P1.25	conserved Plasmodium protein, unknown function	-4.49	0.0020	0.0055	0.0018	2.2	1615	718	3608	2090	-814
354	PF11_0525		-5.58	0.0020	0.0055	0.0018	1.8	1613	507	2902	1048	-266
1289	PF14_0217	conserved Plasmodium protein, unknown function	-3.69	0.0040	0.0098	0.0026	1.6	1602	1036	2611	1019	-1045
2455	PF10_0394	rifin	-2.30	0.0100	0.0208	0.0055	1.8	1598	1933	2804	1988	-2714
2497	PF08_0002	surface-associated interspersed gene 8.2 (SURFIN8.2)	-2.26	0.0240	0.0438	0.0115	1.6	1596	1088	2579	1924	-2029
2468	PFD0285c	lysine decarboxylase, putative	-2.29	0.0180	0.0344	0.0090	1.4	1595	752	2227	1192	-1312
1026	PFF0730c	enoyl-acyl carrier reductase	-4.12	0.0020	0.0055	0.0018	1.8	1585	597	2814	1372	-739
1632	MAL8P1.37	lipoate-protein ligase, putative	-3.23	0.0060	0.0136	0.0036	1.5	1585	755	2399	1058	-1000
172	PFF0510w	histone H3	-6.48	0.0020	0.0055	0.0018	2.7	1581	824	4315	1949	-39
345	PFI0155c	Rab GTPase 7	-5.62	0.0020	0.0055	0.0018	2.6	1573	908	4041	2013	-453
338	PF13_0220	conserved Plasmodium protein, unknown function	-5.64	0.0020	0.0055	0.0018	2.0	1572	533	3147	1293	-251
2528	PF14_0698	conserved Plasmodium protein, unknown function	-2.23	0.0220	0.0408	0.0107	1.3	1560	689	2019	826	-1056
1512	PF10_0075	transcription factor with AP2 domain(s), putative	-3.37	0.0020	0.0055	0.0018	1.8	1556	662	2840	1782	-1160
2511	PF13_0161	conserved Plasmodium protein, unknown function	-2.25	0.0200	0.0376	0.0099	1.6	1554	966	2493	1878	-1906
1861	PF14_0740	Plasmodium exported protein (hyp17), unknown function	-2.96	0.0080	0.0173	0.0046	1.9	1553	1237	2875	1921	-1836
2328	PFF0847w	rifin, pseudogene	-2.44	0.0160	0.0311	0.0082	1.6	1550	1605	2535	1440	-2060
708	PFD0435c	conserved Plasmodium protein, unknown function	-4.71	0.0020	0.0055	0.0018	1.9	1546	592	2906	1324	-555
938	PF10_0212a	conserved Plasmodium protein, unknown function	-4.28	0.0020	0.0055	0.0018	1.9	1546	824	2910	1399	-859
2037	PFI1345c	conserved Plasmodium protein, unknown function	-2.77	0.0040	0.0098	0.0026	1.6	1531	876	2426	1398	-1379
2223	PF07_0125	tRNA pseudouridine synthase D, putative	-2.55	0.0120	0.0243	0.0064	1.5	1525	753	2218	1169	-1230
2203	MAL8P1.161	Plasmodium exported protein (hyp7), unknown function	-2.58	0.0140	0.0277	0.0073	1.4	1511	915	2154	951	-1223
635	PFF0375c	conserved Plasmodium protein, unknown function	-4.86	0.0020	0.0055	0.0018	1.7	1504	500	2613	1035	-427
333	PF10_0165	DNA polymerase delta catalytic subunit	-5.67	0.0020	0.0055	0.0018	2.1	1487	591	3082	1286	-282
571	PFF1010c	DnaJ protein, putative	-5.01	0.0020	0.0055	0.0018	2.5	1480	581	3672	2087	-476
1833	PFE1565w	conserved Plasmodium protein, unknown function	-3.00	0.0020	0.0055	0.0018	1.4	1472	514	2052	844	-779
619	PFD0595w	conserved Apicomplexan protein, unknown function	-4.90	0.0020	0.0055	0.0018	2.1	1469	587	3084	1538	-510
1762	PFL2005w	replication factor C subunit 4	-3.08	0.0020	0.0055	0.0018	1.5	1451	509	2248	1195	-907
164	PF11_0035	Plasmodium exported protein, unknown function	-6.51	0.0020	0.0055	0.0018	3.6	1447	1579	5152	2450	-324
1214	PF13_0314	conserved Plasmodium protein, unknown function	-3.82	0.0020	0.0055	0.0018	2.4	1446	465	3413	2492	-991
1122	PFC0381c	conserved Plasmodium protein, unknown function	-3.98	0.0020	0.0055	0.0018	1.6	1443	661	2357	980	-727
103	PFB1035w	rifin	-7.04	0.0020	0.0055	0.0018	3.5	1433	1534	4987	2113	-92

895	PFE1640w	erythrocyte membrane protein 1 (PfEMP1), truncated	-4.36	0.0020	0.0055	0.0018	2.2	1423	1281	3113	1565	-1156
697	PF08_0063	ClpB protein, putative	-4.73	0.0020	0.0055	0.0018	2.7	1421	889	3797	2343	-857
2396	PF10_0261	WD-repeat protein, putative	-2.37	0.0160	0.0311	0.0082	1.4	1416	464	1985	1110	-1005
1148	MAL7P1.56	erythrocyte membrane protein 1, PfEMP1	-3.93	0.0020	0.0055	0.0018	2.1	1402	1085	2883	1606	-1210
109	PF07_0136	rifin	-7.02	0.0020	0.0055	0.0018	3.0	1395	1102	4221	1742	-19
987	MAL8P1.6	early transcribed membrane protein 8	-4.18	0.0020	0.0055	0.0018	1.8	1381	515	2547	1294	-643
861	PF14_0304	conserved Plasmodium protein, unknown function	-4.42	0.0020	0.0055	0.0018	2.0	1380	815	2805	1425	-815
1449	MAL8P1.206	Plasmodium exported protein, unknown function	-3.46	0.0020	0.0055	0.0018	3.1	1380	1202	4215	3885	-2252
151	PF07_0130	stevor	-6.63	0.0020	0.0055	0.0018	3.1	1374	986	4318	2010	-52
1340	PFE0955w	conserved Plasmodium protein, unknown function	-3.63	0.0020	0.0055	0.0018	1.6	1373	534	2212	1038	-733
680	PF08_0006	prohibitin, putative	-4.76	0.0020	0.0055	0.0018	2.3	1354	780	3142	1719	-710
205	PFF1565c	rifin	-6.24	0.0020	0.0055	0.0018	2.4	1352	911	3304	1329	-288
2498	MAL7P1.43	rifin, pseudogene	-2.26	0.0140	0.0277	0.0073	1.6	1348	906	2197	1673	-1731
454	PF14_0243b	MORN repeat protein, putative	-5.31	0.0020	0.0055	0.0018	2.4	1347	1081	3224	1474	-677
1874	PFL0925w	formin 2, putative	-2.95	0.0080	0.0173	0.0046	2.3	1345	1666	3120	2597	-2488
1595	PF11_0294	6-phosphofructokinase	-3.27	0.0020	0.0055	0.0018	1.6	1313	510	2138	1157	-842
1514	PFL0510c	conserved Plasmodium protein, unknown function	-3.37	0.0020	0.0055	0.0018	2.0	1302	568	2555	1758	-1074
1260	PFD0735c	conserved Plasmodium protein, unknown function	-3.75	0.0040	0.0098	0.0026	1.6	1300	641	2055	826	-713
271	PFL1720w	serine hydroxymethyltransferase	-5.87	0.0020	0.0055	0.0018	2.3	1266	586	2934	1301	-220
567	MAL13P1.314		-5.01	0.0020	0.0055	0.0018	2.3	1264	689	2895	1484	-543
1959	MAL13P1.67	methionyl-tRNA formyltransferase, putative	-2.86	0.0100	0.0208	0.0055	1.8	1253	1099	2286	1511	-1577
247	MAL13P1.202	conserved Plasmodium protein, unknown function	-6.03	0.0020	0.0055	0.0018	2.3	1251	666	2854	1177	-240
1939	PFB0790c	conserved Plasmodium membrane protein, unknown fun	-2.88	0.0060	0.0136	0.0036	1.5	1240	522	1871	981	-872
404	PFA0065w	Pfmc-2TM Maurer's cleft two transmembrane protein	-5.46	0.0020	0.0055	0.0018	3.4	1240	1386	4265	2455	-815
1869	PFF0385c	conserved Plasmodium protein, unknown function	-2.95	0.0020	0.0055	0.0018	1.6	1234	587	2025	1216	-1012
2075	PFE0265c	conserved Plasmodium protein, unknown function	-2.73	0.0060	0.0136	0.0036	1.7	1232	569	2115	1512	-1198
1755	PFI1615w	conserved Plasmodium protein, unknown function	-3.08	0.0040	0.0098	0.0026	1.8	1231	805	2167	1323	-1193
721	PF10_0282	conserved Plasmodium protein, unknown function	-4.69	0.0020	0.0055	0.0018	2.0	1230	558	2486	1225	-527
1351	PFI0795w	conserved Plasmodium protein, unknown function	-3.62	0.0020	0.0055	0.0018	2.6	1228	993	3141	2450	-1530
1643	MAL13P1.255	N6-adenine-specific methylase, putative	-3.22	0.0020	0.0055	0.0018	1.7	1225	442	2055	1206	-819
2166	PFB0770c	conserved Plasmodium membrane protein, unknown fun	-2.61	0.0180	0.0344	0.0090	1.5	1209	374	1765	993	-811
774	MAL8P1.147		-4.58	0.0020	0.0055	0.0018	3.3	1208	1254	3952	2737	-1247
770	PFL1545c	chaperonin, cpn60	-4.59	0.0020	0.0055	0.0018	2.0	1206	573	2363	1137	-553
165	PFI1815c	rifin	-6.51	0.0020	0.0055	0.0018	3.3	1193	1140	3960	1850	-222
451	PFF1495w	conserved Plasmodium protein, unknown function	-5.32	0.0020	0.0055	0.0018	3.2	1189	1022	3812	2260	-659
2101	PFI0775w	glycolipid transfer protein, putative	-2.70	0.0020	0.0055	0.0018	2.5	1187	1235	3018	3159	-2562
1612	PFD0675w	apicoplast ribosomal protein L10 precursor, putative	-3.25	0.0020	0.0055	0.0018	1.5	1182	418	1824	900	-676
963	PF11_0128	coq4 homolog, putative	-4.23	0.0020	0.0055	0.0018	1.6	1181	524	1877	676	-504
2221	PF14_0706	conserved Plasmodium protein, unknown function	-2.56	0.0080	0.0173	0.0046	2.1	1179	1468	2451	2103	-2299
2315	PF08_0080	conserved Plasmodium protein, unknown function	-2.46	0.0140	0.0277	0.0073	1.5	1178	739	1745	943	-1115
420	PFC1105w	stevor (3D7-stevorT3-2)	-5.40	0.0020	0.0055	0.0018	3.0	1169	1217	3513	1865	-738
1332	PF11_0015	hypothetical protein, pseudogene	-3.64	0.0020	0.0055	0.0018	2.4	1169	1144	2770	1928	-1470
1646	PFD0615c	erythrocyte membrane protein 1, PfEMP1	-3.22	0.0100	0.0208	0.0055	1.7	1165	950	1958	907	-1064

1683	PFF1595c	erythrocyte membrane protein 1, PfEMP1	-3.17	0.0080	0.0173	0.0046	1.7	1163	802	2023	1144	-1086
1818	MAL13P1.59	Plasmodium exported protein (PHISTa), unknown functio	-3.01	0.0060	0.0136	0.0036	2.0	1138	1035	2285	1652	-1539
321	PFC0701w	mitochondrial ribosomal protein L27 precursor, putative	-5.70	0.0020	0.0055	0.0018	2.2	1137	535	2468	1051	-256
2510	MAL7P1.52	rifin, pseudogene	-2.25	0.0160	0.0311	0.0082	1.8	1134	1172	2043	1720	-1983
1637	PF11_0077	DEAD/DEAH box helicase, putative	-3.22	0.0020	0.0055	0.0018	1.9	1131	959	2196	1404	-1297
884	PFL2640c	rifin	-4.37	0.0020	0.0055	0.0018	2.3	1123	1030	2610	1423	-966
395	PFA0680c	Pfmc-2TM Maurer's cleft two transmembrane protein	-5.47	0.0020	0.0055	0.0018	2.8	1119	898	3156	1660	-522
1523	MAL13P1.312	conserved Plasmodium protein, unknown function	-3.36	0.0020	0.0055	0.0018	2.9	1115	1103	3216	2923	-1925
835	MAL7P1.207	conserved Plasmodium protein, unknown function	-4.45	0.0020	0.0055	0.0018	1.9	1113	543	2150	1046	-553
298	PF13_0251	DNA topoisomerase III, putative	-5.79	0.0020	0.0055	0.0018	2.0	1105	335	2201	884	-123
274	PF11_0339	glycine cleavage H protein	-5.86	0.0020	0.0055	0.0018	2.5	1104	894	2804	1205	-400
456	PF13_0021	small heat shock protein, putative	-5.31	0.0020	0.0055	0.0018	3.0	1104	647	3362	2012	-401
2083	PF08_0129	serine/threonine protein phosphatase, putative	-2.72	0.0020	0.0055	0.0018	1.6	1103	443	1713	1035	-867
2243	MAL7P1.18	serine/threonine protein kinase, putative	-2.53	0.0020	0.0055	0.0018	1.5	1102	417	1655	1013	-877
407	PFF1545w	rifin	-5.44	0.0020	0.0055	0.0018	3.0	1093	1317	3313	1665	-762
1707	PFC10_API0017	null	-3.15	0.0020	0.0055	0.0018	1.9	1087	765	2112	1461	-1201
300	PFL0010c	rifin	-5.78	0.0020	0.0055	0.0018	3.1	1084	882	3352	1774	-388
1035	PFC10_API0060	null	-4.11	0.0020	0.0055	0.0018	2.0	1083	491	2186	1247	-636
470	PF10_0006	rifin	-5.26	0.0020	0.0055	0.0018	2.3	1077	883	2503	1103	-560
2464	PF11_0214	conserved Plasmodium protein, unknown function	-2.30	0.0080	0.0173	0.0046	2.2	1063	555	2312	2626	-1931
279	PF07_0064	drug/metabolite exporter, drug/metabolite transporter	-5.84	0.0020	0.0055	0.0018	3.3	1056	647	3505	1982	-179
399	PFI0025c	rifin	-5.46	0.0020	0.0055	0.0018	2.3	1055	919	2425	955	-505
701	PFL0470w	conserved Plasmodium protein, unknown function	-4.73	0.0020	0.0055	0.0018	2.2	1054	501	2361	1288	-482
643	PF10_0336	conserved Plasmodium protein, unknown function	-4.83	0.0020	0.0055	0.0018	2.0	1052	674	2098	896	-524
833	PF14_0522	conserved Plasmodium protein, unknown function	-4.46	0.0020	0.0055	0.0018	2.7	1049	614	2851	1912	-724
756	PFE0500c	conserved Plasmodium protein, unknown function	-4.61	0.0020	0.0055	0.0018	2.7	1040	525	2770	1783	-578
1614	PF07_0134	rifin	-3.25	0.0020	0.0055	0.0018	2.2	1032	1310	2259	1485	-1568
1648	MAL8P1.18	conserved Plasmodium protein, unknown function	-3.21	0.0020	0.0055	0.0018	1.7	1027	427	1754	1048	-749
1981	PF11_0057	conserved Plasmodium protein, unknown function	-2.84	0.0120	0.0243	0.0064	1.7	1026	468	1744	1174	-925
874	MAL13P1.251	conserved Plasmodium protein, unknown function	-4.39	0.0020	0.0055	0.0018	2.0	1012	413	2030	1081	-476
1910	PF07_0023	minchromosome maintenance (MCM) complex subunit, p	-2.91	0.0040	0.0098	0.0026	2.0	1008	721	2065	1673	-1336
2569	PF10_0303	25 kDa ookinete surface antigen precursor	-2.19	0.0200	0.0376	0.0099	2.5	1006	1252	2563	3325	-3020
644	PFL1030w	membrane skeletal protein, putative	-4.83	0.0020	0.0055	0.0018	3.3	970	434	3231	2264	-437
1700	PF14_0672	phosphodiesterase delta, putative	-3.15	0.0020	0.0055	0.0018	2.3	970	392	2232	1935	-1065
794	PF11_0009	rifin	-4.53	0.0020	0.0055	0.0018	2.3	966	655	2185	1197	-633
803	PFI0050c	rifin	-4.52	0.0020	0.0055	0.0018	2.4	965	879	2333	1287	-798
2143	PF13_0338	cysteine-rich surface protein	-2.64	0.0060	0.0136	0.0036	1.8	961	693	1684	1206	-1176
2146	PFD0711c		-2.64	0.0080	0.0173	0.0046	2.1	939	954	2011	1821	-1703
476	PF10_0093	protein phosphatase, putative	-5.24	0.0020	0.0055	0.0018	2.4	919	319	2206	1174	-206
792	PFF0655c	adapter-related protein, putative	-4.54	0.0020	0.0055	0.0018	2.0	915	550	1830	873	-507
2012	PFB0815w	calcium dependent protein kinase 1	-2.80	0.0040	0.0098	0.0026	2.5	897	1174	2271	2197	-1996
2451	PFL1380w	RNA pseudouridylate synthase, putative	-2.31	0.0220	0.0408	0.0107	1.5	896	381	1368	951	-859
2283	PFI0600w		-2.50	0.0120	0.0243	0.0064	1.6	891	691	1468	973	-1086

1501	PFE0020c	rifin	-3.39	0.0020	0.0055	0.0018	2.0	887	937	1813	1084	-1095
475	PF11_0340	conserved Plasmodium protein, unknown function	-5.24	0.0020	0.0055	0.0018	3.3	886	521	2922	1852	-338
1298	PFI1360c	serine/threonine protein phosphatase, putative	-3.68	0.0040	0.0098	0.0026	1.7	878	408	1507	764	-543
574	PFE0795c	protein phosphatase, putative	-5.01	0.0020	0.0055	0.0018	2.5	868	588	2143	1146	-458
2469	PF14_0674	conserved Plasmodium protein, unknown function	-2.29	0.0200	0.0376	0.0099	1.8	858	934	1547	1244	-1489
281	PFF1590w	rifin	-5.83	0.0020	0.0055	0.0018	3.5	857	650	3015	1728	-220
1884	PFI0670w	conserved Plasmodium protein, unknown function	-2.93	0.0040	0.0098	0.0026	1.7	854	661	1474	874	-915
1554	PF11_0442	transcription factor with AP2 domain(s), putative	-3.33	0.0020	0.0055	0.0018	2.5	831	480	2048	1747	-1009
537	PF14_0675	conserved Plasmodium protein, unknown function	-5.07	0.0020	0.0055	0.0018	2.2	827	390	1834	916	-299
1809	PFB0330c	serine repeat antigen 7	-3.02	0.0020	0.0055	0.0018	1.7	810	320	1359	848	-620
587	PF11_0529	rifin	-4.98	0.0020	0.0055	0.0018	3.9	810	887	3183	2213	-726
1461	PFL1910c	conserved Plasmodium protein, unknown function	-3.44	0.0020	0.0055	0.0018	3.1	806	357	2476	2359	-1046
1163	PF14_0770	rifin	-3.90	0.0020	0.0055	0.0018	2.2	799	718	1767	1057	-806
1075	PF11_0425	conserved Plasmodium protein, unknown function	-4.05	0.0020	0.0055	0.0018	2.5	796	359	1975	1395	-574
1945	PFD0420c	flap endonuclease 1	-2.87	0.0060	0.0136	0.0036	1.5	794	331	1201	635	-560
2467	PFC0575w	conserved Plasmodium protein, unknown function	-2.29	0.0120	0.0243	0.0064	1.7	793	510	1354	1120	-1068
355	PFI1825w	rifin	-5.58	0.0020	0.0055	0.0018	3.1	766	651	2375	1304	-345
2367	PFA0015c	var-like erythrocyte membrane protein 1	-2.40	0.0180	0.0344	0.0090	2.0	755	1071	1513	1255	-1569
1184	MAL13P1.86	cholinephosphate cytidylyltransferase	-3.87	0.0020	0.0055	0.0018	2.0	755	856	1491	592	-712
2383	PFF0060w	Pfmc-2TM Maurer's cleft two transmembrane protein	-2.38	0.0100	0.0208	0.0055	2.0	749	1256	1514	1165	-1656
668	PF10_0401	rifin	-4.78	0.0020	0.0055	0.0018	3.5	742	1073	2598	1674	-892
2100	PF14_0384	allantoicase, putative	-2.70	0.0060	0.0136	0.0036	2.4	741	868	1761	1702	-1550
1914	PFC0371w	conserved protein, unknown function	-2.91	0.0060	0.0136	0.0036	2.1	739	811	1522	1130	-1158
2064	PF10_0092	metallopeptidase, putative	-2.74	0.0060	0.0136	0.0036	2.6	735	898	1924	1985	-1694
886	PFB0840w	replication factor C, subunit 2	-4.37	0.0020	0.0055	0.0018	3.0	734	542	2190	1566	-653
245	MAL13P1.535	rifin	-6.04	0.0020	0.0055	0.0018	4.0	732	660	2942	1706	-156
1379	PF10_0013	Plasmodium exported protein (hyp12), unknown function	-3.57	0.0020	0.0055	0.0018	3.8	731	882	2787	2723	-1548
393	PF14_0768	rifin	-5.47	0.0020	0.0055	0.0018	3.7	729	924	2682	1566	-538
324	MAL7P1.227	stevor	-5.69	0.0020	0.0055	0.0018	3.3	723	497	2391	1374	-203
2366	PF14_0092	conserved Plasmodium membrane protein, unknown fun	-2.40	0.0100	0.0208	0.0055	2.4	723	641	1733	1987	-1619
639	PFE0800w	RAP protein, putative	-4.85	0.0020	0.0055	0.0018	2.7	719	630	1968	1146	-526
1175	PFB1070w		-3.88	0.0020	0.0055	0.0018	4.7	715	1335	3346	3124	-1828
1951	PF14_0578	conserved Plasmodium protein, unknown function	-2.87	0.0040	0.0098	0.0026	1.9	699	450	1322	994	-821
287	MAL8P1.212	RESA-like pseudogene	-5.82	0.0020	0.0055	0.0018	5.2	694	661	3577	2362	-140
1287	PFI1000w	conserved Plasmodium protein, unknown function	-3.69	0.0020	0.0055	0.0018	3.2	685	518	2173	1924	-955
1908	PFL0080c	NIMA related kinase 3	-2.91	0.0020	0.0055	0.0018	3.8	682	737	2599	3162	-1983
375	PFB0025c	stevor	-5.52	0.0020	0.0055	0.0018	3.7	680	753	2486	1473	-420
2207	PFL1155w	GTP cyclohydrolase I	-2.57	0.0080	0.0173	0.0046	3.6	678	1398	2417	3097	-2756
659	PF07_0113	conserved Plasmodium protein, unknown function	-4.80	0.0020	0.0055	0.0018	2.4	675	321	1629	936	-303
2291	PFI0995w	conserved Plasmodium protein, unknown function	-2.49	0.0060	0.0136	0.0036	2.7	661	443	1815	2239	-1529
380	PFC0035w	rifin	-5.50	0.0020	0.0055	0.0018	4.0	661	772	2646	1644	-431
1913	PFD1105w	asparagine-rich protein	-2.91	0.0040	0.0098	0.0026	3.0	660	722	1951	2089	-1519
1526	PFA0590w	ABC transporter, (CT family), putative	-3.35	0.0040	0.0098	0.0026	2.9	640	482	1837	1701	-986

2553	PF10_0355	merozoite surface protein	-2.20	0.0020	0.0055	0.0018	2.1	640	353	1362	1580	-1211
551	PF14_0064	phosphatase, putative	-5.05	0.0020	0.0055	0.0018	2.7	635	655	1685	858	-463
379	MAL8P1.219	rifin	-5.50	0.0020	0.0055	0.0018	3.9	628	604	2480	1568	-321
1019	PF11_0511	Plasmodium exported protein, unknown function	-4.14	0.0020	0.0055	0.0018	5.1	625	891	3175	2924	-1265
1230	PFA0255c	conserved Plasmodium protein, unknown function	-3.79	0.0020	0.0055	0.0018	3.5	623	838	2158	1853	-1156
1286	PF10_0398	rifin	-3.69	0.0020	0.0055	0.0018	3.0	617	568	1834	1542	-893
1017	PF14_0732	Plasmodium exported protein (PHISTb), unknown functio	-4.14	0.0020	0.0055	0.0018	2.7	616	510	1679	1183	-630
243	PF11_0021	rifin	-6.05	0.0020	0.0055	0.0018	4.4	613	565	2712	1633	-99
457	PFB0015c	rifin	-5.31	0.0020	0.0055	0.0018	3.9	612	492	2410	1607	-302
2027	PF11_0464	serine/threonine protein kinase, putative	-2.78	0.0020	0.0055	0.0018	3.3	611	782	2034	2417	-1777
628	PFB1015w	rifin	-4.87	0.0020	0.0055	0.0018	3.8	608	798	2307	1572	-671
2343	PFI0030c	rifin	-2.42	0.0100	0.0208	0.0055	1.7	606	654	1055	722	-927
703	PF11_0020	rifin	-4.72	0.0020	0.0055	0.0018	3.9	604	549	2327	1726	-553
1613	PFE0440w	conserved Plasmodium protein, unknown function	-3.25	0.0020	0.0055	0.0018	2.5	585	339	1442	1258	-741
583	MAL13P1.515	rifin	-4.98	0.0020	0.0055	0.0018	3.6	583	544	2110	1429	-446
2583	PF11_0357	zinc finger protein, putative	-2.17	0.0200	0.0376	0.0099	2.6	573	586	1518	2080	-1721
2108	PF14_0151	RNA-binding protein Nova-1, putative	-2.69	0.0020	0.0055	0.0018	2.1	564	273	1184	1106	-759
2017	PFI0960w	dolichyl-diphosphooligosaccharide-protein glycosyltransf	-2.79	0.0020	0.0055	0.0018	2.7	564	298	1543	1701	-1019
2443	PFD0045c	rifin	-2.31	0.0180	0.0344	0.0090	2.1	562	1063	1173	936	-1388
1561	PF08_0118	conserved Plasmodium protein, unknown function	-3.32	0.0020	0.0055	0.0018	2.4	559	270	1319	1098	-608
1513	PF14_0737	lysophospholipase, putative	-3.37	0.0020	0.0055	0.0018	3.7	544	655	2013	2064	-1250
260	PFC0025c	stevor	-5.98	0.0020	0.0055	0.0018	4.5	540	615	2416	1448	-187
1511	MAL8P1.84	conserved Plasmodium protein, unknown function	-3.37	0.0020	0.0055	0.0018	2.1	534	365	1117	790	-571
2005	PFI0980w	long chain fatty acid elongation enzyme, putative	-2.81	0.0020	0.0055	0.0018	3.3	529	362	1739	2092	-1244
2124	PFL1010c	conserved Plasmodium protein, unknown function	-2.67	0.0080	0.0173	0.0046	1.9	526	703	1009	664	-883
1668	PFL0365c	conserved Plasmodium protein, unknown function	-3.19	0.0020	0.0055	0.0018	4.0	519	248	2091	2407	-1083
1470	MAL13P1.7	stevor	-3.42	0.0020	0.0055	0.0018	3.5	518	1013	1835	1683	-1378
292	PFF1550w	stevor	-5.80	0.0020	0.0055	0.0018	5.1	516	422	2654	1772	-56
1369	PF07_0061	conserved Plasmodium protein, unknown function	-3.59	0.0020	0.0055	0.0018	4.5	504	475	2281	2394	-1092
2551	PFD1230c	rifin	-2.20	0.0220	0.0408	0.0107	2.6	501	851	1286	1594	-1659
396	PFC0915w	ATP-dependent RNA helicase, putative	-5.47	0.0020	0.0055	0.0018	4.6	497	298	2296	1592	-90
1263	PF10_0363	pyruvate kinase 2, putative	-3.74	0.0020	0.0055	0.0018	3.7	486	765	1802	1598	-1047
2060	PFI0080w	stevor	-2.75	0.0040	0.0098	0.0026	2.7	480	840	1278	1236	-1278
740	PF10_0400	rifin	-4.65	0.0020	0.0055	0.0018	3.9	469	554	1841	1368	-550
2447	PFD1100c	conserved Plasmodium protein, unknown function	-2.31	0.0120	0.0243	0.0064	3.0	460	506	1392	1928	-1503
405	PF11_0049	NOT family protein, putative	-5.44	0.0020	0.0055	0.0018	3.8	456	280	1735	1127	-128
2586	PFL0795c	male development gene 1	-2.16	0.0140	0.0277	0.0073	2.6	454	549	1190	1602	-1415
953	PFB0055c	rifin	-4.26	0.0020	0.0055	0.0018	3.6	451	558	1613	1253	-649
1757	MAL7P1.310	stevor, pseudogene	-3.08	0.0060	0.0136	0.0036	2.6	450	643	1159	987	-922
516	PFB0050c	stevor, pseudogene	-5.14	0.0020	0.0055	0.0018	4.7	440	433	2089	1530	-314
1989	PF10_0236	conserved Plasmodium protein, unknown function	-2.82	0.0020	0.0055	0.0018	2.7	439	288	1183	1268	-812
1197	PF14_0443	centrin-2	-3.84	0.0020	0.0055	0.0018	3.2	419	298	1330	1134	-521
2509	PFL0420w	amino acid transporter, putative	-2.25	0.0040	0.0098	0.0026	2.3	413	162	955	1173	-793

2115	PF10_0016	acyl-CoA binding protein, isoform 2, ACBP2	-2.68	0.0060	0.0136	0.0036	4.0	413	661	1651	2198	-1620
1290	PF10_0140	conserved protein, unknown function	-3.69	0.0020	0.0055	0.0018	2.5	409	582	1026	658	-623
1889	PFI0070w	rifin	-2.93	0.0040	0.0098	0.0026	2.5	408	482	1006	913	-797
1645	PFE1545c	formin 1, putative	-3.22	0.0020	0.0055	0.0018	2.4	401	144	952	830	-423
2186	PFD0800c	conserved Plasmodium protein, unknown function	-2.60	0.0040	0.0098	0.0026	2.5	394	352	1001	1104	-849
1081	PF14_0004	rifin	-4.04	0.0020	0.0055	0.0018	3.1	388	584	1201	854	-625
2734	PF11_0540	conserved Plasmodium protein, unknown function	-2.00	0.0160	0.0311	0.0082	3.0	382	230	1144	1856	-1324
2496	PF14_0294	mitogen-activated protein kinase 1	-2.26	0.0060	0.0136	0.0036	2.8	379	344	1062	1452	-1112
1843	PFL1925w	cell division protein FtsH, putative	-2.98	0.0020	0.0055	0.0018	3.1	365	256	1132	1243	-731
2236	PF08_0017	conserved Plasmodium protein, unknown function	-2.54	0.0020	0.0055	0.0018	2.5	362	175	913	1054	-678
2704	PFL1075w	transcription factor with AP2 domain(s), putative	-2.03	0.0040	0.0098	0.0026	2.6	361	166	944	1399	-982
2020	MAL7P1.225		-2.79	0.0020	0.0055	0.0018	3.5	353	320	1229	1517	-961
2431	PF14_0759	conserved Plasmodium protein, unknown function, pseu	-2.32	0.0080	0.0173	0.0046	2.6	353	244	902	1139	-834
2456	PFE0945c		-2.30	0.0040	0.0098	0.0026	4.3	347	316	1505	2451	-1609
1965	MAL13P1.500	rifin	-2.85	0.0020	0.0055	0.0018	3.2	334	441	1063	1196	-908
2369	PF11_0443	DnaJ protein, putative	-2.40	0.0020	0.0055	0.0018	2.5	324	217	820	994	-715
2494	PFE0680w	secreted ookinete protein, putative	-2.26	0.0020	0.0055	0.0018	2.5	320	204	803	1033	-753
2289	PFF1200w	conserved Plasmodium protein, unknown function	-2.49	0.0020	0.0055	0.0018	3.2	305	253	988	1327	-897
2039	PFF0380w	conserved Plasmodium protein, unknown function	-2.77	0.0020	0.0055	0.0018	5.7	299	216	1714	2497	-1297
2352	MAL7P1.184	rifin	-2.42	0.0120	0.0243	0.0064	2.4	296	610	704	646	-849
2235	PFL1965w	null	-2.54	0.0020	0.0055	0.0018	3.1	279	259	872	1123	-790
2711	PF14_0644	conserved Plasmodium protein, unknown function	-2.03	0.0180	0.0344	0.0090	4.5	270	418	1207	2239	-1720
2355	PF14_0815	conserved Plasmodium protein, unknown function	-2.41	0.0020	0.0055	0.0018	3.1	241	116	743	1013	-628
1520	PF10_0189	conserved Plasmodium protein, unknown function	-3.37	0.0020	0.0055	0.0018	3.5	226	133	794	819	-385
2681	PF08_0024	conserved Plasmodium protein, unknown function	-2.05	0.0040	0.0098	0.0026	3.6	206	87	739	1270	-824
1174	PF11_0517	rifin	-3.89	0.0020	0.0055	0.0018	5.4	205	149	1113	1138	-379
2338	PF14_0683	conserved Plasmodium protein, unknown function	-2.43	0.0020	0.0055	0.0018	6.9	203	206	1403	2408	-1414
2415	PFL1095c	conserved Plasmodium protein, unknown function	-2.35	0.0060	0.0136	0.0036	6.1	200	172	1212	2109	-1269
2445	PF13_0071	probable protein, unknown function	-2.31	0.0020	0.0055	0.0018	3.2	191	124	603	866	-579
2201	PF11_0431	inner membrane complex protein 1b, putative	-2.58	0.0020	0.0055	0.0018	6.0	171	83	1031	1633	-856
2006	PF13_0005	rifin	-2.80	0.0020	0.0055	0.0018	6.1	161	173	979	1422	-777
2674	PF11_0460	conserved Plasmodium protein, unknown function	-2.06	0.0060	0.0136	0.0036	5.6	125	130	697	1356	-913
1468	PF14_0402	conserved Plasmodium protein, unknown function	-3.43	0.0020	0.0055	0.0018	12.9	114	128	1473	1940	-709
2649	PFB0730w	DEAD/DEAH box helicase, putative	-2.10	0.0040	0.0098	0.0026	9.8	106	69	1047	2199	-1327
2068	PFE0040c	mature parasite-infected erythrocyte surface antigen,ery	-2.74	0.0020	0.0055	0.0018	12.0	98	231	1176	1918	-1071
3101	MAL13P1.475	Plasmodium exported protein (PHISTb), unknown functio	-1.58	0.0040	0.0098	0.0026	6.6	69	49	453	1193	-857
2269	PF10_0307	conserved Plasmodium protein, unknown function	-2.50	0.0020	0.0055	0.0018	13.2	47	35	624	1128	-586
2645	PF13_0191	MSP7-like protein	-2.10	0.0020	0.0055	0.0018	17.5	30	27	519	1140	-678
2859	PF13_0123	conserved Plasmodium protein, unknown function	-1.86	0.0259	0.0469	0.0123	20.9	30	40	619	1553	-1003
2725	PF14_0633a	conserved Plasmodium protein, unknown function	-2.01	0.0060	0.0136	0.0036	23.8	26	39	629	1466	-902
Genes highly induced in Cluster B relative to Cluster A												
27	PF10_0095	conserved Plasmodium membrane protein, unknown fun	8.36	0.0020	0.0055	0.0018	1.0	9273	101	9008	130	34
132	PF14_0563	DEAD box helicase, putative	6.78	0.0020	0.0055	0.0018	1.1	8823	242	8079	498	5

224	PF13_0340	exosome complex exonuclease, putative	6.12	0.0020	0.0055	0.0018	1.2	7505	299	6134	1068	4
201	PFB0196c	dynein light chain, putative	6.26	0.0020	0.0055	0.0018	1.2	7409	322	5974	1090	23
35	PFL1760w	conserved Plasmodium protein, unknown function	8.06	0.0020	0.0055	0.0018	1.3	7401	529	5751	900	222
16	PFE0655w	conserved Plasmodium protein, unknown function	8.64	0.0020	0.0055	0.0018	1.2	7352	376	6022	685	270
133	PFE0390w	conserved Plasmodium protein, unknown function	6.74	0.0020	0.0055	0.0018	1.4	7069	475	4974	1468	151
75	PFF1355w	conserved Plasmodium protein, unknown function	7.30	0.0020	0.0055	0.0018	1.3	7019	444	5352	1055	167
134	PFE1430c	cyclophilin, putative	6.74	0.0020	0.0055	0.0018	1.2	7010	441	5618	941	10
62	PFL1680w	splicing factor 3b, subunit 3, 130kD, putative	7.52	0.0020	0.0055	0.0018	1.3	6983	406	5480	917	179
15	PFI0415c	ribosomal RNA methyltransferase, putative	8.67	0.0020	0.0055	0.0018	1.4	6759	594	4892	928	344
92	PFE1200w	conserved Plasmodium protein, unknown function	7.10	0.0020	0.0055	0.0018	1.5	6682	898	4365	1411	10
136	MAL7P1.16	conserved Plasmodium membrane protein, unknown fun	6.73	0.0020	0.0055	0.0018	1.6	6630	758	4267	1600	6
79	PFL1935c	vesicle fusion and protein sorting subunit 16, putative	7.21	0.0020	0.0055	0.0018	1.6	6555	834	4225	1420	77
70	PFL1405w	conserved Plasmodium membrane protein, unknown fun	7.36	0.0020	0.0055	0.0018	1.2	6334	547	5084	694	9
37	PF11_0397	beta-catenin-like protein 1, putative	7.94	0.0020	0.0055	0.0018	2.0	6221	940	3168	1710	403
98	MAL13P1.153	ribonuclease P protein subunit rpr, putative	7.08	0.0020	0.0055	0.0018	1.6	6146	875	3735	1499	37
55	PF14_0511	glucose-6-phosphate dehydrogenase-6-phosphogluconol	7.60	0.0020	0.0055	0.0018	1.5	6139	604	4215	1132	188
56	MAL13P1.317	conserved Plasmodium protein, unknown function	7.57	0.0020	0.0055	0.0018	1.8	6138	798	3353	1674	313
19	PF14_0355	conserved Plasmodium membrane protein, unknown fun	8.52	0.0020	0.0055	0.0018	1.5	6101	644	3966	1102	388
82	PFF1250w	conserved Plasmodium protein, unknown function	7.20	0.0020	0.0055	0.0018	1.4	5972	441	4285	1086	160
57	MAL8P1.141	conserved Plasmodium protein, unknown function	7.55	0.0020	0.0055	0.0018	1.8	5712	1150	3121	1375	66
17	PFB0745w	conserved Plasmodium protein, unknown function	8.58	0.0020	0.0055	0.0018	1.7	5665	956	3328	1066	315
77	PF10_0082	conserved Plasmodium membrane protein, unknown fun	7.22	0.0020	0.0055	0.0018	1.8	5584	502	3184	1573	325
40	MAL13P1.292	riboflavin kinase / FAD synthase family protein, putative	7.86	0.0020	0.0055	0.0018	2.1	5412	1071	2595	1507	238
26	PFE1085w	DEAD/DEAH box ATP-dependent RNA helicase, putative	8.38	0.0020	0.0055	0.0018	2.0	5357	749	2696	1422	489
13	PF14_0136	diphthamide synthesis protein, putative	8.75	0.0020	0.0055	0.0018	2.3	4761	866	2076	1316	503
3	PFI0815c	methyltransferase, putative	9.97	0.0020	0.0055	0.0018	3.1	4745	1318	1528	1128	771
47	PFL2078w	conserved Plasmodium protein, unknown function	7.74	0.0020	0.0055	0.0018	1.8	4576	883	2571	1030	93
39	PFC0695w	conserved Plasmodium protein, unknown function	7.87	0.0020	0.0055	0.0018	2.4	4448	1441	1823	1099	85
34	PFD1065c	conserved Plasmodium protein, unknown function	8.09	0.0020	0.0055	0.0018	2.0	4000	965	2030	875	129
49	MAL8P1.55	conserved Plasmodium protein, unknown function	7.73	0.0020	0.0055	0.0018	2.3	3809	1052	1640	1053	64
43	PF14_0305	leucine-rich repeat protein	7.79	0.0020	0.0055	0.0018	1.7	3666	1028	2114	454	70
38	PFB0501c	conserved Plasmodium protein, unknown function	7.91	0.0020	0.0055	0.0018	2.7	3107	1041	1162	828	75
23	PFE1090w	nucleotide binding protein, putative	8.48	0.0020	0.0055	0.0018	5.3	1578	848	296	205	230
Genes induced in Cluster B relative to Cluster A												
2205	PF11_0040	early transcribed membrane protein 11.2	2.57	0.0040	0.0098	0.0026	1.0	9997	2	9993	7	-5
2034	PFB0120w	early transcribed membrane protein 2	2.78	0.0020	0.0055	0.0018	1.0	9995	3	9982	22	-13
1983	PF10_0019	early transcribed membrane protein 10.1	2.84	0.0040	0.0098	0.0026	1.0	9991	3	9988	5	-4
1426	PFE0065w	skeleton-binding protein 1	3.49	0.0020	0.0055	0.0018	1.0	9988	3	9967	28	-11
2526	PF11_0039	early transcribed membrane protein 11.1	2.23	0.0240	0.0438	0.0115	1.0	9984	6	9977	16	-14
1439	PFI1755c	ring-exported protein 3	3.47	0.0020	0.0055	0.0018	1.0	9969	19	9904	90	-44
1062	PFI1740c		4.07	0.0020	0.0055	0.0018	1.0	9954	19	9896	67	-29
2147	MAL7P1.170	Plasmodium exported protein, unknown function	2.64	0.0060	0.0136	0.0036	1.0	9952	25	9916	64	-52
684	MAL13P1.237	conserved Plasmodium protein, unknown function	4.76	0.0020	0.0055	0.0018	1.0	9949	22	9904	43	-19

1377	MAL7P1.172	Plasmodium exported protein (PHISTc), unknown functio	3.57	0.0020	0.0055	0.0018	1.0	9941	35	9875	85	-54
1238	PF08_0074	DNA/RNA-binding protein Alba, putative	3.78	0.0020	0.0055	0.0018	1.0	9934	34	9894	43	-37
1012	MAL7P1.76	conserved Plasmodium protein, unknown function	4.15	0.0020	0.0055	0.0018	1.0	9923	31	9863	66	-37
2238	PFC0400w	60S acidic ribosomal protein P2, putative	2.53	0.0120	0.0243	0.0064	1.0	9922	25	9907	21	-31
2376	PF10_0115	QF122 antigen	2.39	0.0240	0.0438	0.0115	1.0	9920	35	9898	36	-49
1749	PFE0070w	interspersed repeat antigen	3.10	0.0020	0.0055	0.0018	1.0	9911	89	9732	274	-183
2225	PF14_0075	plasmepsin IV	2.55	0.0060	0.0136	0.0036	1.0	9895	43	9851	76	-75
1799	PFL1170w	polyadenylate-binding protein, putative	3.03	0.0100	0.0208	0.0055	1.0	9876	64	9820	72	-81
1830	PFE0660c	purine nucleoside phosphorylase	3.00	0.0040	0.0098	0.0026	1.0	9864	124	9738	176	-175
2155	PF10_0100	conserved Plasmodium protein, unknown function	2.63	0.0040	0.0098	0.0026	1.0	9859	184	9600	458	-383
400	PFB0445c	DEAD box helicase	5.46	0.0020	0.0055	0.0018	1.0	9858	45	9803	32	-22
879	PF13_0346	60S ribosomal protein L40/UBI, putative	4.39	0.0020	0.0055	0.0018	1.0	9855	39	9795	59	-37
1594	PFL1420w	macrophage migration inhibitory factor	3.28	0.0020	0.0055	0.0018	1.0	9855	96	9741	149	-132
752	PFC0975c	peptidyl-prolyl cis-trans isomerase	4.63	0.0020	0.0055	0.0018	1.0	9854	62	9760	85	-53
1077	PFI0580c	falstatin	4.05	0.0020	0.0055	0.0018	1.0	9827	71	9727	106	-77
2589	PF13_0276	membrane-associated histidine rich protein 2, (MARHP2)	2.16	0.0080	0.0173	0.0046	1.0	9826	118	9636	419	-347
2095	PF13_0045	40S ribosomal protein S27, putative	2.70	0.0080	0.0173	0.0046	1.0	9822	66	9762	93	-100
2177	PFL0060w	Plasmodium exported protein, unknown function	2.61	0.0020	0.0055	0.0018	1.1	9820	246	9228	1092	-746
2644	PF07_0126	transcription factor with AP2 domain(s), putative	2.10	0.0200	0.0376	0.0099	1.0	9818	89	9711	239	-221
1182	PF13_0214	elongation factor 1-gamma, putative	3.87	0.0020	0.0055	0.0018	1.0	9815	68	9733	88	-73
1424	PFL2095w	translation initiation factor SUI1, putative	3.49	0.0060	0.0136	0.0036	1.0	9809	64	9733	91	-80
1429	PF14_0122	nuclear transport factor 2, putative	3.49	0.0020	0.0055	0.0018	1.0	9800	105	9638	210	-153
764	PFE0865c	splicing factor, putative	4.60	0.0020	0.0055	0.0018	1.0	9799	93	9615	180	-89
1502	PFA0420w	conserved Plasmodium protein, unknown function	3.39	0.0040	0.0098	0.0026	1.0	9790	184	9538	330	-262
2377	PFL2565w	Plasmodium exported protein (PHISTa), unknown functio	2.39	0.0060	0.0136	0.0036	1.0	9782	214	9576	381	-390
1656	MAL8P1.69	14-3-3 protein, putative	3.20	0.0020	0.0055	0.0018	1.0	9772	122	9658	140	-149
1684	PF13_0116	rhoptry protein 2, putative	3.17	0.0060	0.0136	0.0036	1.0	9770	46	9722	63	-61
2252	PFA0375c	lipid/sterol:H symporter	2.52	0.0160	0.0311	0.0082	1.0	9759	44	9708	92	-85
768	PF11_0183	GTP-binding nuclear protein ran/tc4	4.59	0.0020	0.0055	0.0018	1.0	9752	47	9698	41	-35
1875	PF14_0585	40S ribosomal protein S28e, putative	2.95	0.0060	0.0136	0.0036	1.0	9750	57	9693	82	-82
731	PFE0885w	eukaryotic translation initiation factor, putative	4.68	0.0020	0.0055	0.0018	1.0	9744	63	9673	54	-45
2189	PF14_0013	DnaJ protein, putative	2.60	0.0080	0.0173	0.0046	1.1	9741	360	9217	942	-778
2086	PFI1720w	gametocytogenesis-implicated protein	2.72	0.0040	0.0098	0.0026	1.1	9741	344	9115	1091	-809
1695	PF13_0194	probable protein, unknown function	3.16	0.0020	0.0055	0.0018	1.0	9740	156	9465	406	-287
1303	PFI0720w	transporter, putative	3.68	0.0020	0.0055	0.0018	1.0	9737	79	9605	163	-110
2373	PFI1435w	RNA binding protein, putative	2.40	0.0220	0.0408	0.0107	1.0	9713	67	9659	96	-108
2321	PFC0300c	60S ribosomal protein L7, putative	2.46	0.0100	0.0208	0.0055	1.0	9711	78	9636	134	-137
961	PF11_0250	splicing factor, putative	4.24	0.0020	0.0055	0.0018	1.0	9708	65	9614	93	-65
2183	PF14_0784	ribosome biogenesis protein, NOP10-like	2.60	0.0080	0.0173	0.0046	1.0	9704	74	9650	79	-100
1604	MAL13P1.233	DNA/RNA-binding protein Alba, putative	3.26	0.0080	0.0173	0.0046	1.0	9702	73	9603	136	-109
2363	PFB0105c	Plasmodium exported protein (PHISTc), unknown functio	2.41	0.0120	0.0243	0.0064	1.0	9689	415	9306	696	-729
1023	PFL1410c	ABC transporter, (CT family)	4.13	0.0020	0.0055	0.0018	1.0	9682	70	9514	190	-93
1277	PF14_0068	fibrillarin, putative	3.72	0.0040	0.0098	0.0026	1.0	9682	55	9625	59	-57

2639	PF10_0268	merozoite capping protein 1	2.10	0.0200	0.0376	0.0099	1.0	9679	212	9506	361	-400
1952	PFA0635c	Plasmodium exported protein (hyp1), unknown function	2.87	0.0020	0.0055	0.0018	1.1	9678	223	9074	1015	-635
1336	PF11_0445	DNA-directed RNA polymerase I, putative	3.64	0.0020	0.0055	0.0018	1.0	9675	63	9607	75	-70
1422	PF10_0063	DNA/RNA-binding protein Alba, putative	3.49	0.0040	0.0098	0.0026	1.0	9664	94	9553	134	-118
1926	PF10_0160	serine/threonine protein kinase, FIKK family	2.89	0.0060	0.0136	0.0036	1.0	9661	69	9577	130	-115
1878	PF13_0268	60S ribosomal protein L17, putative	2.94	0.0020	0.0055	0.0018	1.0	9658	102	9536	185	-164
2620	MAL13P1.214	phosphoethanolamine N-methyltransferase	2.12	0.0200	0.0376	0.0099	1.0	9656	108	9537	260	-248
1564	PFI0930c	nucleosome assembly protein	3.31	0.0020	0.0055	0.0018	1.0	9648	130	9454	265	-201
2216	PFB0915w	liver stage antigen 3	2.56	0.0060	0.0136	0.0036	1.0	9648	167	9366	520	-406
1306	PFC0920w	histone H2A variant, putative	3.67	0.0020	0.0055	0.0018	1.0	9639	95	9468	214	-138
2084	PF08_0056	zinc finger protein, putative	2.72	0.0120	0.0243	0.0064	1.0	9633	108	9514	195	-183
2429	PFE1490c	RING zinc finger protein, putative	2.33	0.0200	0.0376	0.0099	1.0	9631	93	9566	111	-139
1992	PF13_0257	glutamate--tRNA ligase, putative	2.82	0.0040	0.0098	0.0026	1.0	9624	102	9490	217	-185
915	PF14_0434	conserved Plasmodium protein, unknown function	4.33	0.0020	0.0055	0.0018	1.0	9621	58	9525	98	-60
911	PF13_0163	conserved Plasmodium protein, unknown function	4.33	0.0020	0.0055	0.0018	1.0	9619	131	9419	198	-128
2191	PF14_0368	thioredoxin peroxidase 1	2.59	0.0020	0.0055	0.0018	1.0	9615	133	9329	530	-377
2299	PFF0220w	conserved Plasmodium protein, unknown function	2.48	0.0200	0.0376	0.0099	1.0	9607	105	9517	156	-170
1280	PF14_0487	conserved Plasmodium protein, unknown function	3.71	0.0020	0.0055	0.0018	1.0	9606	185	9326	334	-240
2540	PF11_0455a	conserved Plasmodium protein, unknown function	2.21	0.0120	0.0243	0.0064	1.0	9605	207	9429	349	-379
2297	PFI1735c	ring-exported protein 1	2.48	0.0180	0.0344	0.0090	1.0	9599	169	9473	206	-248
1278	PF07_0054	histone H2B variant, putative	3.72	0.0020	0.0055	0.0018	1.0	9597	126	9391	249	-170
2486	PF08_0076	40S ribosomal protein S16, putative	2.27	0.0160	0.0311	0.0082	1.0	9580	62	9508	147	-136
1247	PF14_0655	helicase 45	3.77	0.0020	0.0055	0.0018	1.0	9579	132	9408	194	-154
1575	PF14_0076	plasmepsin I	3.30	0.0020	0.0055	0.0018	1.1	9579	260	9054	748	-483
2178	PF13_0143	phosphoribosylpyrophosphate synthetase	2.61	0.0080	0.0173	0.0046	1.0	9568	116	9453	193	-194
973	MAL13P1.163		4.21	0.0020	0.0055	0.0018	1.0	9564	77	9299	302	-113
2559	PF11_0377		2.20	0.0240	0.0438	0.0115	1.0	9561	89	9499	117	-144
908	PF11_0245	translation elongation factor EF-1, subunit alpha, putative	4.34	0.0020	0.0055	0.0018	1.0	9559	61	9389	185	-75
1362	PF11_0260	60S ribosomal protein L35, putative	3.60	0.0020	0.0055	0.0018	1.0	9553	132	9405	170	-153
2090	MAL8P1.88	conserved Plasmodium protein, unknown function	2.71	0.0040	0.0098	0.0026	1.0	9550	271	9139	708	-567
1354	PFD0950w	ran binding protein 1, putative	3.62	0.0020	0.0055	0.0018	1.0	9536	106	9391	175	-136
1827	PFL0145c	high mobility group protein	3.00	0.0060	0.0136	0.0036	1.0	9534	151	9324	318	-260
1615	PFL0035c	acyl-CoA synthetase, PfACS7	3.25	0.0020	0.0055	0.0018	1.1	9525	359	8905	884	-623
1870	PF11_0191	box C/D snoRNP rRNA 2'-O-methylation factor, putative	2.95	0.0080	0.0173	0.0046	1.0	9522	97	9428	132	-136
1681	PF14_0437	DEAD/DEAH box ATP-dependent RNA helicase, putative	3.17	0.0080	0.0173	0.0046	1.0	9508	86	9414	126	-118
1881	PF10_0340	methionine-tRNA ligase, putative	2.94	0.0020	0.0055	0.0018	1.0	9504	323	9274	272	-364
1259	MAL8P1.40	RNA binding protein, putative	3.75	0.0040	0.0098	0.0026	1.0	9502	99	9378	139	-114
1438	PFL1490w	atypical protein kinase, RIO family, putative	3.47	0.0020	0.0055	0.0018	1.0	9500	161	9302	243	-207
558	PFI1696c	conserved protein, unknown function	5.04	0.0020	0.0055	0.0018	1.0	9498	76	9361	117	-56
1499	PF14_0753	Plasmodium exported protein (hyp13), unknown function	3.39	0.0020	0.0055	0.0018	1.1	9496	299	8669	1170	-641
866	PFI1175c	RNA binding protein, putative	4.41	0.0020	0.0055	0.0018	1.1	9475	160	9004	506	-195
918	PF13_0138	MSF1-like protein, putative	4.32	0.0020	0.0055	0.0018	1.0	9471	111	9289	185	-113
2197	PF13_0262	lysine-tRNA ligase, putative	2.58	0.0100	0.0208	0.0055	1.0	9464	135	9345	194	-211

2230	PF14_0423	eukaryotic initiation factor 2alpha kinase 1	2.55	0.0020	0.0055	0.0018	1.0	9462	211	9173	527	-448
2264	MAL13P1.77	conserved Plasmodium protein, unknown function	2.51	0.0140	0.0277	0.0073	1.0	9460	101	9348	201	-190
1766	PF10_0028		3.07	0.0060	0.0136	0.0036	1.0	9451	184	9211	350	-294
2076	PF14_0359	HSP40, subfamily A, putative	2.73	0.0040	0.0098	0.0026	1.0	9441	81	9339	170	-149
2767	PF10_0180	conserved Plasmodium protein, unknown function	1.96	0.0200	0.0376	0.0099	1.0	9418	176	9223	463	-445
2281	PF11_0291	conserved Plasmodium protein, unknown function	2.50	0.0180	0.0344	0.0090	1.0	9415	101	9313	182	-181
2253	PFB0875c	chromatin-binding protein, putative	2.52	0.0200	0.0376	0.0099	1.0	9414	164	9281	218	-250
2491	PF11_0347	conserved Plasmodium protein, unknown function	2.26	0.0100	0.0208	0.0055	1.0	9408	209	9130	576	-507
1788	PF10_0085	small subunit rRNA processing protein, putative	3.05	0.0020	0.0055	0.0018	1.0	9406	116	9180	350	-240
1016	PFD0455w	40S ribosomal processing protein, putative	4.14	0.0020	0.0055	0.0018	1.0	9400	172	9214	166	-152
2957	PF11_0086	MIF4G domain containing protein	1.74	0.0220	0.0408	0.0107	1.0	9394	82	9286	296	-270
2030	PF11_0251	endoplasmic reticulum oxidoreductin, putative	2.78	0.0060	0.0136	0.0036	1.0	9392	135	9219	283	-244
1719	MAL7P1.4	Plasmodium exported protein (hyp4), unknown function	3.12	0.0040	0.0098	0.0026	1.0	9392	394	8957	597	-556
1773	PFA0670c	Plasmodium exported protein (hyp8), unknown function	3.07	0.0040	0.0098	0.0026	1.0	9385	169	9167	317	-268
2746	PFC1020c	40S ribosomal protein S3A, putative	1.98	0.0200	0.0376	0.0099	1.0	9384	178	9199	434	-426
977	PF13_0315	rRNA associated RNA binding protein, putative	4.21	0.0020	0.0055	0.0018	1.0	9370	102	9230	139	-101
1940	PF14_0579	60S ribosomal protein L27, putative	2.88	0.0020	0.0055	0.0018	1.0	9366	142	9097	443	-316
275	PF10_0123	GMP synthetase	5.85	0.0020	0.0055	0.0018	1.0	9365	135	8936	341	-46
810	PFB0295w	adenylosuccinate lyase	4.51	0.0020	0.0055	0.0018	1.0	9361	197	9081	255	-172
2278	PFI1640c	conserved Plasmodium protein, unknown function	2.50	0.0040	0.0098	0.0026	1.0	9359	229	9092	486	-448
819	PF10_0285	conserved Plasmodium protein, unknown function	4.49	0.0020	0.0055	0.0018	1.0	9355	153	9043	315	-156
1858	PFL1975c	conserved Plasmodium protein, unknown function	2.97	0.0040	0.0098	0.0026	1.0	9354	156	9177	261	-240
1433	PF11_0512	ring-infected erythrocyte surface antigen 2	3.48	0.0020	0.0055	0.0018	1.1	9348	200	8787	771	-411
1671	MAL7P1.109		3.18	0.0020	0.0055	0.0018	1.0	9342	88	9130	318	-194
2699	PFE1035c	BIS(5'-nucleosyl)-tetraphosphatase (diadenosine tetraphо	2.03	0.0060	0.0136	0.0036	1.1	9337	535	8716	1427	-1341
3000	PF11_0331	TCP-1/cpn60 chaperonin family, putative	1.70	0.0200	0.0376	0.0099	1.0	9335	135	9123	604	-527
1624	PFD0450c	pre-mRNA splicing factor, putative	3.24	0.0020	0.0055	0.0018	1.0	9322	242	9113	241	-275
796	PFI1085w	ubiquitin-like protein, putative	4.53	0.0020	0.0055	0.0018	1.0	9303	126	9061	240	-124
991	PF14_0336	conserved Plasmodium protein, unknown function	4.18	0.0020	0.0055	0.0018	1.0	9299	138	9073	238	-151
120	PFC0965w	conserved Plasmodium protein, unknown function	6.88	0.0020	0.0055	0.0018	1.1	9292	174	8833	293	-7
836	PFL1235c	conserved Plasmodium protein, unknown function	4.45	0.0020	0.0055	0.0018	1.0	9290	72	9097	204	-83
2782	PFF1315w	ankyrin-repeat protein, putative	1.95	0.0240	0.0438	0.0115	1.0	9290	189	9034	623	-556
1802	PF11_0166	conserved Plasmodium protein, unknown function	3.03	0.0040	0.0098	0.0026	1.1	9288	420	8697	889	-718
2362	PF11_0199	conserved Plasmodium protein, unknown function	2.41	0.0120	0.0243	0.0064	1.0	9286	125	9180	187	-206
1160	PFE1350c	ubiquitin conjugating enzyme 13, putative	3.90	0.0020	0.0055	0.0018	1.0	9285	177	8945	401	-237
2605	PFC0255c	ubiquitin conjugating enzyme E2, putative	2.15	0.0259	0.0469	0.0123	1.0	9285	178	9157	251	-301
2487	PFL0405w	conserved Plasmodium protein, unknown function	2.27	0.0120	0.0243	0.0064	1.0	9284	111	9173	221	-221
1385	PFI1480w	conserved Plasmodium protein, unknown function	3.56	0.0020	0.0055	0.0018	1.0	9284	140	9129	177	-163
820	PF11_0189	insulinase, putative	4.48	0.0020	0.0055	0.0018	1.0	9268	141	8914	367	-155
2871	PF11_0368a	conserved Plasmodium protein, unknown function	1.85	0.0020	0.0055	0.0018	1.0	9267	135	8961	802	-632
1759	PF14_0603	conserved Plasmodium protein, unknown function	3.08	0.0040	0.0098	0.0026	1.0	9258	93	9118	208	-161
1786	PFB0085c	DnaJ protein, putative	3.05	0.0020	0.0055	0.0018	1.1	9257	254	8511	1179	-687
2199	PF07_0080	40S ribosomal protein S10, putative	2.58	0.0040	0.0098	0.0026	1.0	9256	231	9013	418	-406

2398	PFC0090w	Plasmodium exported protein, unknown function	2.37	0.0200	0.0376	0.0099	1.1	9250	1307	8174	1938	-2168
1156	PFL2475w	DEAD/DEAH box ATP-dependent RNA helicase, putative	3.91	0.0020	0.0055	0.0018	1.0	9245	150	9034	233	-171
900	PF14_0117	conserved Plasmodium protein, unknown function	4.36	0.0020	0.0055	0.0018	1.1	9241	284	8372	948	-362
1550	PFB0490c	conserved Plasmodium protein, unknown function	3.33	0.0040	0.0098	0.0026	1.0	9239	190	9035	253	-240
1857	PF10_0021	Plasmodium exported protein (PHISTc), unknown functio	2.97	0.0020	0.0055	0.0018	1.1	9234	209	8777	733	-485
1393	PF14_0174	pseudouridine synthase, putative	3.55	0.0020	0.0055	0.0018	1.0	9226	123	9038	238	-173
2320	MAL7P1.15	conserved Plasmodium membrane protein, unknown fun	2.46	0.0180	0.0344	0.0090	1.0	9220	126	9097	222	-224
1819	PFL2400w	rRNA processing protein, putative	3.01	0.0020	0.0055	0.0018	1.0	9213	149	9045	243	-224
1405	PFI1020c	inosine-5'-monophosphate dehydrogenase	3.53	0.0020	0.0055	0.0018	1.1	9202	228	8728	629	-383
958	PF10_0150	methionine aminopeptidase 1b, putative	4.25	0.0020	0.0055	0.0018	1.0	9195	204	8818	400	-227
1373	PFI0345w	GTPase activator, putative	3.58	0.0020	0.0055	0.0018	1.0	9193	183	8790	530	-309
2353	MAL8P1.4	Plasmodium exported protein (PHISTc), unknown functio	2.42	0.0240	0.0438	0.0115	1.0	9185	337	8887	533	-572
2818	PF11_0437	60S ribosomal protein L28, putative	1.90	0.0140	0.0277	0.0073	1.0	9181	192	8835	875	-721
1608	PFC0486c	conserved Plasmodium protein, unknown function	3.26	0.0020	0.0055	0.0018	1.1	9176	418	8628	744	-614
1627	PFB0245c	DNA-directed RNA polymerase II 16 kDa subunit, putative	3.24	0.0020	0.0055	0.0018	1.0	9173	179	8830	496	-332
2245	PFI0820c	RNA-binding protein musashi, putative	2.53	0.0080	0.0173	0.0046	1.0	9168	317	8754	758	-661
1228	PF14_0277	coatamer beta subunit, putative	3.80	0.0020	0.0055	0.0018	1.0	9159	108	8894	330	-173
824	PF11_0200	U2 snRNP auxiliary factor, small subunit, putative	4.47	0.0020	0.0055	0.0018	1.0	9157	222	8742	415	-221
2422	PFC0870w	elongation factor 1 (EF-1), putative	2.34	0.0140	0.0277	0.0073	1.0	9155	263	8946	378	-432
952	PFI1695c	small nuclear ribonucleoprotein (snRNP), putative	4.26	0.0020	0.0055	0.0018	1.0	9147	269	8811	314	-247
2470	PF14_0229	conserved Plasmodium protein, unknown function	2.29	0.0120	0.0243	0.0064	1.0	9146	150	9011	258	-274
2334	PFB0106c	Plasmodium exported protein, unknown function	2.44	0.0060	0.0136	0.0036	1.1	9145	386	8702	829	-772
1412	MAL13P1.348	glycosylphosphatidylinositol anchor attachment 1 protein	3.51	0.0020	0.0055	0.0018	1.1	9141	441	8101	1401	-803
387	PFE1320w	conserved Plasmodium protein, unknown function	5.49	0.0020	0.0055	0.0018	1.0	9136	134	8764	312	-75
2018	PFD0180c	CGI-201 protein, short form	2.79	0.0020	0.0055	0.0018	1.0	9135	146	8959	283	-253
2617	PF14_0752	Plasmodium exported protein (PHISTa), unknown functio	2.13	0.0200	0.0376	0.0099	1.1	9124	757	8121	2222	-1975
1905	PF13_0106	conserved protein, unknown function	2.92	0.0020	0.0055	0.0018	1.1	9122	374	8317	1315	-884
705	PF14_0127	N-myristoyltransferase	4.72	0.0020	0.0055	0.0018	1.1	9121	304	8177	946	-307
2244	PFL1945c	early transcribed membrane protein 12	2.53	0.0180	0.0344	0.0090	1.1	9114	407	8672	787	-751
2336	PFF0430w	chaperone, putative	2.44	0.0040	0.0098	0.0026	1.0	9114	218	8699	814	-616
1784	PFL0310c	eukaryotic translation initiation factor 3 subunit 8, putati	3.05	0.0020	0.0055	0.0018	1.0	9111	107	8824	452	-272
2547	PF14_0690	histone deacetylase, putative	2.21	0.0240	0.0438	0.0115	1.0	9108	284	8746	769	-690
2003	PF07_0067	large ribosomal subunit assembling factor, putative	2.81	0.0060	0.0136	0.0036	1.0	9103	147	8904	325	-273
1455	PFE0425w	conserved Plasmodium protein, unknown function	3.45	0.0020	0.0055	0.0018	1.0	9103	279	8797	364	-338
750	PFC0970w	conserved Plasmodium membrane protein, unknown fun	4.63	0.0020	0.0055	0.0018	1.1	9103	225	8641	451	-214
1880	PF10_0018	alpha/beta hydrolase, putative	2.94	0.0020	0.0055	0.0018	1.1	9096	232	8462	1037	-635
2393	PFE1150w	multidrug resistance protein	2.37	0.0140	0.0277	0.0073	1.0	9091	394	8690	760	-753
2113	PF11_0096	casein kinase 2, alpha subunit	2.68	0.0020	0.0055	0.0018	1.0	9085	210	8764	559	-449
1111	PFI0740c	ubiquitin conjugating enzyme, putative	4.00	0.0020	0.0055	0.0018	1.0	9078	193	8826	263	-204
1085	PFF0500c	step II splicing factor, putative	4.04	0.0020	0.0055	0.0018	1.0	9075	174	8855	222	-177
1079	MAL13P1.122	SET domain protein, putative	4.05	0.0020	0.0055	0.0018	1.1	9074	400	8479	637	-442
653	PFE1115c	S-adenosylmethionine-dependent methyltransferase, put	4.81	0.0020	0.0055	0.0018	1.0	9069	249	8675	343	-197
830	PF11_0458	conserved Plasmodium protein, unknown function	4.47	0.0020	0.0055	0.0018	1.0	9065	158	8795	265	-153

2002	PF10_0200	ribosomal processing ATPase, putative	2.81	0.0020	0.0055	0.0018	1.0	9064	139	8931	201	-206
514	PF14_0469		5.14	0.0020	0.0055	0.0018	1.1	9060	339	8385	577	-241
2128	PF10_0103	eukaryotic translation initiation factor 2 beta subunit, put	2.66	0.0060	0.0136	0.0036	1.0	9055	251	8762	496	-454
2817	PFF0450c	Zn2 or Fe2 permease	1.91	0.0020	0.0055	0.0018	1.1	9048	184	8529	1323	-989
2385	PFL0460w	lsm7 homologue, putative	2.38	0.0180	0.0344	0.0090	1.0	9046	238	8870	302	-364
1411	PF13_0253	ethanolamine-phosphate cytidylyltransferase, putative	3.52	0.0020	0.0055	0.0018	1.1	9042	356	8098	1280	-693
1556	MAL13P1.70	conserved Plasmodium membrane protein, unknown fun	3.33	0.0040	0.0098	0.0026	1.0	9031	229	8721	414	-333
1689	PF07_0122	BRIX protein, putative	3.16	0.0020	0.0055	0.0018	1.0	9029	152	8813	310	-245
2450	MAL8P1.23	ubiquitin-protein ligase 1, putative	2.31	0.0160	0.0311	0.0082	1.0	9021	293	8649	749	-670
1539	PFL1340c	conserved Plasmodium protein, unknown function	3.34	0.0020	0.0055	0.0018	1.1	9014	483	8516	607	-592
422	PF10_0327	Myb2 protein	5.40	0.0020	0.0055	0.0018	1.0	9005	154	8677	268	-95
2187	PF08_0135	conserved Plasmodium protein, unknown function	2.60	0.0140	0.0277	0.0073	1.0	8993	142	8868	203	-220
1311	PFI0475w	small nuclear ribonucleoprotein (snRNP), putative	3.66	0.0020	0.0055	0.0018	1.0	8993	102	8813	225	-147
1300	PF13_0013	deoxyhypusine hydroxylase	3.68	0.0020	0.0055	0.0018	1.0	8989	182	8746	286	-224
1949	MAL13P1.288	conserved Plasmodium protein, unknown function	2.87	0.0060	0.0136	0.0036	1.1	8985	343	8551	682	-592
1224	PF14_0282	acid phosphatase, putative	3.80	0.0020	0.0055	0.0018	1.1	8984	561	8176	928	-680
1007	PF14_0546	conserved Plasmodium protein, unknown function	4.16	0.0020	0.0055	0.0018	1.1	8964	301	8420	589	-346
2330	PFE1365w	conserved Plasmodium protein, unknown function	2.44	0.0240	0.0438	0.0115	1.0	8960	330	8665	522	-557
1141	PF07_0040	lysophospholipase, putative	3.94	0.0020	0.0055	0.0018	1.1	8953	309	8388	653	-397
1739	PFE1370w	hsp70 interacting protein, putative	3.10	0.0020	0.0055	0.0018	1.0	8950	290	8563	560	-463
1706	PFF1145c	protein kinase, putative	3.15	0.0020	0.0055	0.0018	1.1	8945	307	8473	688	-523
1069	PF14_0150	DNA-directed RNA polymerase I/III subunit, putative	4.06	0.0020	0.0055	0.0018	1.0	8934	174	8642	321	-203
1996	PFL1835w	conserved Plasmodium protein, unknown function	2.82	0.0100	0.0208	0.0055	1.1	8924	594	8361	842	-873
2132	PF13_0209	conserved Plasmodium protein, unknown function	2.66	0.0020	0.0055	0.0018	1.1	8923	239	8495	763	-574
1390	PF07_0091	cell cycle control protein cwf15, putative	3.55	0.0040	0.0098	0.0026	1.0	8914	205	8697	244	-233
2164	PFB0695c	acyl-CoA synthetase, PfACS8	2.62	0.0040	0.0098	0.0026	1.0	8913	180	8615	537	-419
1099	PFC0395w	asparagine synthetase, putative	4.02	0.0020	0.0055	0.0018	1.1	8907	537	7934	1095	-659
2439	PF14_0450	conserved Plasmodium protein, unknown function	2.32	0.0100	0.0208	0.0055	1.0	8906	183	8669	477	-423
2361	PF14_0728	conserved Plasmodium protein, unknown function	2.41	0.0060	0.0136	0.0036	1.0	8899	312	8500	768	-682
540	PFC0825c	cleavage and polyadenylation specifity factor, putative	5.07	0.0020	0.0055	0.0018	1.0	8899	149	8482	383	-116
2169	PFL2235w	conserved Plasmodium protein, unknown function	2.61	0.0120	0.0243	0.0064	1.0	8893	331	8612	448	-498
1299	PFL2295w	nucleolar rRNA processing protein, putative	3.68	0.0020	0.0055	0.0018	1.0	8892	162	8687	236	-194
1071	PFA0525w	transcription initiation factor TFIIB, putative	4.05	0.0020	0.0055	0.0018	1.1	8891	226	8417	540	-293
483	PFC0375c	U2 snRNP spliceosome subunit, putative	5.22	0.0020	0.0055	0.0018	1.0	8887	151	8521	319	-104
2085	PF10_0068	RNA binding protein, putative	2.72	0.0040	0.0098	0.0026	1.0	8866	312	8528	549	-523
2195	PFF0530w	transketolase	2.58	0.0080	0.0173	0.0046	1.0	8859	331	8585	439	-496
1651	PFF1430c	amino acid transporter, putative	3.21	0.0020	0.0055	0.0018	1.1	8856	305	8328	766	-543
2354	PFF1500c	DEAD/DEAH box ATP-dependent RNA helicase	2.42	0.0240	0.0438	0.0115	1.0	8855	199	8628	427	-400
412	PFD0505c	protein phosphatase, putative	5.42	0.0020	0.0055	0.0018	1.1	8850	298	7953	771	-173
2144	PFE1050w	adenosylhomocysteinase,S-adenosyl-L-homocysteine hyc	2.64	0.0100	0.0208	0.0055	1.0	8846	272	8499	601	-526
2122	PF14_0204	conserved Plasmodium protein, unknown function	2.67	0.0140	0.0277	0.0073	1.1	8845	1199	7749	1741	-1844
565	PF13_0354	alanine--tRNA ligase, putative	5.01	0.0020	0.0055	0.0018	1.1	8841	258	8391	382	-190
1401	PF14_0555	conserved Plasmodium protein, unknown function	3.54	0.0020	0.0055	0.0018	1.1	8841	310	7873	1314	-657

2019	PFL1220w	conserved Plasmodium protein, unknown function	2.79	0.0060	0.0136	0.0036	1.0	8831	320	8494	528	-511
2488	PFI0895c	eukaryotic translation initiation factor 3 subunit 5, putati	2.27	0.0140	0.0277	0.0073	1.0	8829	282	8530	600	-583
1985	PFF0830w	alpha adaptin-like protein, putative	2.83	0.0080	0.0173	0.0046	1.0	8827	238	8566	405	-382
2126	PFA0515w	phosphatidylinositol-4-phosphate-5-kinase	2.66	0.0140	0.0277	0.0073	1.0	8826	181	8673	237	-265
2175	MAL13P1.43	conserved Plasmodium protein, unknown function	2.61	0.0020	0.0055	0.0018	1.0	8821	272	8509	539	-499
1891	PF13_0102	secretory complex protein 63	2.93	0.0120	0.0243	0.0064	1.0	8809	293	8558	339	-382
969	PFL0620c	glycerol-3-phosphate acyltransferase	4.22	0.0020	0.0055	0.0018	1.1	8804	321	8197	650	-365
1548	PF14_0079	transcription factor with AP2 domain(s), putative	3.33	0.0020	0.0055	0.0018	1.0	8804	374	8395	512	-477
1600	PFA0310c	calcium-transporting ATPase	3.27	0.0020	0.0055	0.0018	1.0	8788	256	8511	354	-334
552	MAL13P1.257	conserved Plasmodium protein, unknown function	5.05	0.0020	0.0055	0.0018	1.2	8785	418	7566	1130	-329
1248	MAL13P1.179	conserved Plasmodium protein, unknown function	3.76	0.0020	0.0055	0.0018	1.1	8778	277	8112	834	-446
2581	PF11_0505	probable protein, unknown function	2.17	0.0180	0.0344	0.0090	1.1	8775	644	8106	1414	-1388
1154	PF08_0003	tryptophan/threonine-rich antigen	3.92	0.0020	0.0055	0.0018	1.2	8774	757	7224	1832	-1039
2029	PF11_0362	protein phosphatase, putative	2.78	0.0020	0.0055	0.0018	1.0	8767	279	8400	603	-514
2682	PFL1345c	histone S-adenosyl methyltransferase, putative	2.05	0.0259	0.0469	0.0123	1.0	8765	176	8640	260	-311
1748	PFF1050w	nascent polypeptide associated complex alpha chain, put	3.10	0.0020	0.0055	0.0018	1.1	8762	307	8196	858	-598
2457	PF13_0324	Sec24 subunit a	2.30	0.0240	0.0438	0.0115	1.1	8759	553	8279	910	-983
1349	PFF0325c	conserved Plasmodium protein, unknown function	3.63	0.0020	0.0055	0.0018	1.1	8754	433	8097	809	-586
871	PF13_0341	DNA-directed RNA polymerase 2, putative	4.40	0.0020	0.0055	0.0018	1.1	8746	191	8313	454	-212
1173	PFL0665c	RNA polymerase subunit 8c, putative	3.89	0.0020	0.0055	0.0018	1.0	8730	193	8424	349	-237
1258	PFC0850c	endonuclease/exonuclease/phosphatase family protein,	3.75	0.0020	0.0055	0.0018	1.0	8730	297	8357	418	-342
2307	PF14_0429	RNA helicase, putative	2.47	0.0120	0.0243	0.0064	1.0	8722	325	8379	626	-606
683	MAL13P1.213	transcription activator, putative	4.76	0.0020	0.0055	0.0018	1.0	8713	216	8302	382	-187
2052	PF08_0079	translation initiation factor SUI1, putative	2.75	0.0060	0.0136	0.0036	1.1	8712	196	8289	736	-508
1650	PFI1280c	protein kinase, putative	3.21	0.0020	0.0055	0.0018	1.0	8709	327	8316	534	-468
316	PFL2430c	eukaryotic translation initiation factor 2b, subunit 2, puta	5.71	0.0020	0.0055	0.0018	1.2	8708	472	7366	1081	-210
1841	PFI0370c	subunit of proteaseome activator complex, putative	2.98	0.0060	0.0136	0.0036	1.1	8707	466	8210	715	-685
1806	PF14_0164	NADP-specific glutamate dehydrogenase	3.02	0.0060	0.0136	0.0036	1.1	8700	935	7812	1206	-1253
1828	PF11_0447	translation initiation factor eIF-1A, putative	3.00	0.0040	0.0098	0.0026	1.0	8698	232	8367	505	-406
1743	PFE0120c	merozoite surface protein 8,ring-stage membrane protei	3.10	0.0040	0.0098	0.0026	1.1	8696	334	8221	696	-555
2647	PF13_0091	conserved Plasmodium protein, unknown function	2.10	0.0140	0.0277	0.0073	1.0	8670	198	8430	535	-492
786	PFE1215c	cytosolic preribosomal GTP-binding protein, putative	4.56	0.0020	0.0055	0.0018	1.1	8666	258	7961	726	-279
365	MAL7P1.150	cysteine desulfurase, putative	5.56	0.0020	0.0055	0.0018	1.1	8661	371	8030	461	-201
1593	PFL0860c	zinc finger protein, putative	3.28	0.0020	0.0055	0.0018	1.1	8659	330	8225	586	-481
798	MAL13P1.135	SNARE protein, putative	4.52	0.0020	0.0055	0.0018	1.1	8656	375	7989	650	-358
351	PF13_0178	translation initiation factor 6, putative	5.59	0.0020	0.0055	0.0018	1.1	8654	258	8140	395	-139
1472	MAL8P1.122	ubiquitin regulatory protein, putative	3.42	0.0020	0.0055	0.0018	1.1	8651	374	7911	1012	-646
1698	PF11_0052	syntaxin, Qa-SNARE family	3.15	0.0060	0.0136	0.0036	1.0	8649	339	8243	565	-497
1808	PF14_0109		3.02	0.0080	0.0173	0.0046	1.0	8645	304	8315	471	-445
2094	PFE1240w	tRNA-YW synthesizing protein, putative	2.70	0.0040	0.0098	0.0026	1.0	8644	289	8308	559	-512
1979	PFD0265w	pre-mRNA splicing factor, putative	2.84	0.0020	0.0055	0.0018	1.0	8642	161	8331	520	-370
968	PF10_0087	diphthine synthase	4.22	0.0020	0.0055	0.0018	1.1	8641	236	8046	661	-302
753	PF10_0293	transcription factor, putative	4.62	0.0020	0.0055	0.0018	1.1	8638	332	8130	461	-284

2531	PFB0265c	DNA repair endonuclease, putative	2.22	0.0200	0.0376	0.0099	1.0	8635	386	8332	583	-666
1907	PF10_0328	bromodomain protein, putative	2.92	0.0020	0.0055	0.0018	1.0	8634	164	8437	302	-268
2610	PFL0930w	clathrin heavy chain, putative	2.14	0.0020	0.0055	0.0018	1.0	8629	185	8248	860	-665
1549	PFF1095w	leucyl tRNA synthase	3.33	0.0020	0.0055	0.0018	1.1	8621	303	8032	828	-541
887	MAL13P1.341	ribosome biogenesis protein MRT4, putative	4.37	0.0020	0.0055	0.0018	1.0	8621	163	8272	366	-180
1036	PFF1295w	conserved Plasmodium protein, unknown function	4.11	0.0020	0.0055	0.0018	1.2	8621	587	7381	1393	-740
1560	PF13_0296	splicing factor 3b subunit, putative	3.32	0.0020	0.0055	0.0018	1.1	8615	273	8186	590	-434
1272	PF14_0407	guanine nucleotide exchange factor, putative	3.73	0.0020	0.0055	0.0018	1.1	8609	465	7489	1418	-763
2096	PFI1700c	sec-1 family protein	2.70	0.0080	0.0173	0.0046	1.0	8605	274	8358	383	-411
1360	PF10_0149	cysteinyl-tRNA synthetase, putative	3.60	0.0020	0.0055	0.0018	1.1	8603	305	7599	1343	-644
1328	PFE0055c	heat shock protein 40, type II	3.64	0.0020	0.0055	0.0018	1.2	8600	863	7316	1567	-1146
1571	PF14_0689	Yip1 protein, putative	3.30	0.0040	0.0098	0.0026	1.1	8595	412	8105	640	-562
1048	PF07_0117	eukaryotic translation initiation factor 2 alpha subunit, pu	4.09	0.0020	0.0055	0.0018	1.1	8594	290	7803	916	-415
1210	PFB0865w	small nuclear ribonucleoprotein, putative	3.83	0.0020	0.0055	0.0018	1.1	8583	306	8170	461	-355
2358	MAL7P1.122	GTP binding protein, putative	2.41	0.0020	0.0055	0.0018	1.1	8583	220	8103	957	-698
377	MAL13P1.36	PNAS-3 related protein, putative	5.51	0.0020	0.0055	0.0018	1.1	8576	240	7974	497	-134
1693	PFI0915w	conserved Plasmodium protein, unknown function	3.16	0.0020	0.0055	0.0018	1.2	8573	906	7244	1913	-1491
2412	PF14_0070	pre-mRNA splicing factor, putative	2.35	0.0180	0.0344	0.0090	1.0	8568	272	8311	485	-500
2392	PF10_0079	conserved Plasmodium protein, unknown function	2.37	0.0180	0.0344	0.0090	1.0	8562	223	8263	588	-512
2259	PFI1075w	conserved Plasmodium protein, unknown function	2.51	0.0160	0.0311	0.0082	1.0	8559	365	8257	503	-566
1380	PF11_0108	U5 snRNP-associated protein, putative	3.57	0.0020	0.0055	0.0018	1.1	8557	287	8127	538	-395
1721	MAL13P1.80		3.12	0.0020	0.0055	0.0018	1.1	8554	281	7991	851	-570
842	PFE0485w	phosphatidylinositol 4-kinase	4.44	0.0020	0.0055	0.0018	1.1	8553	217	8102	463	-229
1403	PF13_0119	Rab GTPase 11a	3.53	0.0020	0.0055	0.0018	1.1	8546	351	8079	576	-460
2036	PFA0330w	small ribosomal subunit assembling AARP2 protein	2.78	0.0060	0.0136	0.0036	1.0	8539	207	8224	530	-421
2501	MAL8P1.127	conserved Plasmodium protein, unknown function	2.26	0.0220	0.0408	0.0107	1.0	8538	145	8403	267	-277
1302	PFE1425c	conserved Plasmodium protein, unknown function	3.68	0.0020	0.0055	0.0018	1.1	8537	254	8062	597	-376
1652	MAL13P1.352	conserved Plasmodium protein, unknown function	3.20	0.0020	0.0055	0.0018	1.0	8532	298	8196	448	-410
2522	PF14_0494	small subunit rRNA processing factor, putative	2.23	0.0080	0.0173	0.0046	1.0	8526	203	8241	601	-519
1772	PFD0745c	nonclathrin coat protein zeta2-cop-related protein, putat	3.07	0.0020	0.0055	0.0018	1.1	8524	283	7891	982	-632
2038	PF14_0059	conserved Plasmodium protein, unknown function	2.77	0.0040	0.0098	0.0026	1.0	8523	249	8244	446	-416
1216	PF13_0343	conserved Plasmodium protein, unknown function	3.82	0.0020	0.0055	0.0018	1.1	8520	425	7998	566	-470
907	MAL13P1.302	SUMO ligase, putative	4.35	0.0020	0.0055	0.0018	1.1	8502	430	7927	538	-393
998	PF11_0341	conserved membrane protein, unknown function	4.17	0.0020	0.0055	0.0018	1.0	8498	332	8120	346	-300
1736	PFD0085c	acyl-CoA synthetase, PfACS6	3.11	0.0040	0.0098	0.0026	1.2	8497	703	7373	1671	-1250
1944	PF10_0179a	PHF5-like protein, putative	2.87	0.0020	0.0055	0.0018	1.1	8495	337	7957	874	-673
1220	PF08_0041	ribosome biogenesis protein nep1 homologue, putative	3.81	0.0020	0.0055	0.0018	1.1	8486	359	7886	710	-469
1466	PFF1000w	cleavage stimulation factor subunit 1-like protein, putativ	3.43	0.0020	0.0055	0.0018	1.1	8485	292	7804	941	-552
2072	MAL7P1.24	large ribosomal subunit processing protein, putative	2.73	0.0040	0.0098	0.0026	1.1	8477	264	8049	735	-570
1378	PFD1070w	eukaryotic initiation factor, putative	3.57	0.0020	0.0055	0.0018	1.1	8463	327	7761	922	-548
1528	PFE1445c	conserved Plasmodium protein, unknown function	3.35	0.0040	0.0098	0.0026	1.0	8462	239	8071	535	-383
855	PFB0860c	DEAD/DEAH box ATP-dependent RNA helicase, putative	4.42	0.0020	0.0055	0.0018	1.1	8461	245	7955	520	-260
1915	PF10_0025	PF70 protein	2.91	0.0040	0.0098	0.0026	1.2	8459	748	7355	1750	-1395

645	PFI1060w	tetratricopeptide repeat family protein, putative	4.83	0.0020	0.0055	0.0018	1.1	8455	136	7842	611	-135
1658	PFI1725w	Plasmodium exported protein, unknown function	3.20	0.0040	0.0098	0.0026	1.3	8453	1213	6721	2448	-1929
1087	PF13_0019	sodium/hydrogen exchanger, Na , H antiporter	4.03	0.0020	0.0055	0.0018	1.1	8452	316	7668	914	-446
2234	PF11_0471	nucleolar preribosomal assembly protein, putative	2.54	0.0080	0.0173	0.0046	1.1	8447	276	8035	760	-624
2584	PF10_0325	haloacid dehalogenase-like hydrolase, putative	2.16	0.0100	0.0208	0.0055	1.1	8440	261	7863	1288	-972
1957	PF10_0225	orotidine monophosphate decarboxylase	2.86	0.0020	0.0055	0.0018	1.1	8438	403	7539	1502	-1006
2046	PFB0255w		2.76	0.0080	0.0173	0.0046	1.1	8437	387	7999	708	-657
1261	PFI0885w	cytochrome b5-like heme/steroid binding protein, putativ	3.75	0.0020	0.0055	0.0018	1.2	8437	1099	6884	1808	-1355
841	PFI1235w	rRNA processing and telomere maintaining methyltransfe	4.44	0.0020	0.0055	0.0018	1.1	8426	341	7948	443	-306
2359	PFF1135w	transcription or splicing factor-like protein, putative	2.41	0.0240	0.0438	0.0115	1.0	8420	367	8178	383	-508
843	PF14_0214	conserved Plasmodium protein, unknown function	4.44	0.0020	0.0055	0.0018	1.0	8417	247	8049	348	-228
1022	PFC0441c		4.14	0.0020	0.0055	0.0018	1.0	8416	299	8063	335	-281
2154	PF14_0621	conserved Plasmodium protein, unknown function	2.63	0.0060	0.0136	0.0036	1.1	8415	584	7669	1301	-1138
1950	PFE1360c	methionine aminopeptidase 1a, putative	2.87	0.0120	0.0243	0.0064	1.0	8414	385	8117	389	-478
2368	PF13_0044	carbamoyl phosphate synthetase	2.40	0.0020	0.0055	0.0018	1.1	8413	245	7989	840	-661
1626	PFB0635w	T-complex protein 1, putative	3.24	0.0020	0.0055	0.0018	1.1	8411	291	7721	1015	-615
2346	PFC1011c	conserved Plasmodium membrane protein, unknown fun	2.42	0.0020	0.0055	0.0018	1.1	8409	278	7686	1443	-998
286	PF14_0170	NOT family protein, putative	5.82	0.0020	0.0055	0.0018	1.1	8407	436	7498	671	-199
2232	PF07_0020	methyltransferase, putative	2.55	0.0100	0.0208	0.0055	1.0	8406	308	8127	471	-500
1115	PFL0530c	conserved Plasmodium protein, unknown function	3.99	0.0020	0.0055	0.0018	1.2	8403	707	7268	1261	-833
1084	PFD0245c	ATP-dependent RNA helicase, putative	4.04	0.0020	0.0055	0.0018	1.1	8400	276	7449	1129	-455
2293	PFI1800w	lysophospholipase, putative	2.49	0.0100	0.0208	0.0055	1.1	8384	1060	7413	1693	-1782
1866	PFD0095c	Plasmodium exported protein (PHISTb), unknown functio	2.96	0.0020	0.0055	0.0018	1.2	8383	645	7084	2085	-1430
2021	PFL0290w	conserved Plasmodium protein, unknown function	2.78	0.0020	0.0055	0.0018	1.1	8382	513	7586	1333	-1050
677	MAL13P1.304	conserved Plasmodium protein, unknown function	4.77	0.0020	0.0055	0.0018	1.1	8382	175	7752	631	-175
2607	PFF0590c	homologue of human HSPC025	2.14	0.0259	0.0469	0.0123	1.0	8379	223	8152	486	-481
1450	MAL13P1.79	conserved Plasmodium protein, unknown function	3.46	0.0020	0.0055	0.0018	1.1	8377	457	7691	893	-663
985	PF08_0020	ubiquitination-mediated degradation component, putativ	4.19	0.0020	0.0055	0.0018	1.1	8372	287	7858	550	-323
1602	PFI0120c	serine/threonine protein kinase, FIKK family	3.26	0.0020	0.0055	0.0018	1.2	8367	876	6869	2124	-1502
186	PF08_0065	nucleolar preribosomal assembly protein, putative	6.38	0.0020	0.0055	0.0018	1.1	8366	206	7603	560	-2
1266	PFB0805c	clathrin coat assembly protein, putative	3.74	0.0020	0.0055	0.0018	1.2	8365	353	7145	1571	-704
1018	MAL8P1.83	eukaryotic translation initiation factor, putative	4.14	0.0020	0.0055	0.0018	1.1	8361	304	7819	589	-351
2163	PFL1425w	T-complex protein 1, gamma subunit, putative	2.62	0.0020	0.0055	0.0018	1.1	8356	338	7488	1600	-1070
2357	PF13_0245	conserved Plasmodium protein, unknown function	2.41	0.0120	0.0243	0.0064	1.0	8351	230	8087	501	-467
1599	PF11_0375	conserved Plasmodium protein, unknown function	3.27	0.0020	0.0055	0.0018	1.0	8350	322	7967	507	-445
2193	PF14_0094	conserved Plasmodium protein, unknown function	2.59	0.0120	0.0243	0.0064	1.1	8339	496	7782	969	-908
1767	MAL7P1.113	DEAD/DEAH box ATP-dependent RNA helicase, putative	3.07	0.0020	0.0055	0.0018	1.1	8334	254	7860	724	-504
1584	PF14_0518	nifU protein, putative	3.29	0.0020	0.0055	0.0018	1.1	8331	451	7713	839	-672
2600	PFC0805w	DNA-directed RNA polymerase II, putative	2.15	0.0220	0.0408	0.0107	1.1	8330	332	7910	916	-828
504	MAL13P1.268	conserved Plasmodium protein, unknown function	5.17	0.0020	0.0055	0.0018	1.5	8330	1473	5641	2228	-1012
1011	PFL0040c	serine/threonine protein kinase, FIKK family	4.15	0.0020	0.0055	0.0018	1.1	8319	587	7397	970	-635
695	PFL1620w	asparagine and aspartate rich protein 1	4.74	0.0020	0.0055	0.0018	1.2	8315	466	6944	1363	-457
2206	PFA0660w	heat shock protein 40, type II	2.57	0.0160	0.0311	0.0082	1.0	8312	309	7993	548	-538

1741	PF10_0163	Plasmodium exported protein (PHISTc), unknown functio	3.10	0.0040	0.0098	0.0026	1.2	8306	1051	6913	2013	-1672
1849	PFC0760c	conserved Plasmodium protein, unknown function	2.97	0.0040	0.0098	0.0026	1.1	8305	325	7814	761	-595
1966	PFF0755c	conserved Plasmodium protein, unknown function	2.85	0.0020	0.0055	0.0018	1.1	8303	333	7505	1342	-877
1183	PFF0525w	conserved Plasmodium protein, unknown function	3.87	0.0020	0.0055	0.0018	1.1	8294	298	7597	846	-447
678	PF14_0567	conserved Plasmodium protein, unknown function	4.77	0.0020	0.0055	0.0018	1.1	8294	473	7249	998	-426
1871	PFB0640c	sec31p	2.95	0.0080	0.0173	0.0046	1.1	8291	344	7872	631	-557
2079	PFC0155c	DNA-directed RNA polymerase subunit I, putative	2.73	0.0040	0.0098	0.0026	1.1	8288	298	7885	680	-574
1609	PFL1175w	conserved Plasmodium protein, unknown function	3.26	0.0020	0.0055	0.0018	1.0	8286	264	7904	531	-413
1865	PF13_0023	DNA-directed RNA polymerase 2, putative	2.96	0.0040	0.0098	0.0026	1.1	8277	300	7861	641	-525
2196	MAL7P1.147	ubiquitin carboxyl-terminal hydrolase, putative	2.58	0.0120	0.0243	0.0064	1.0	8271	282	7969	522	-502
296	PF14_0084	conserved Plasmodium protein, unknown function	5.79	0.0020	0.0055	0.0018	1.2	8258	289	6704	1292	-27
1847	PFI1425w	conserved Plasmodium protein, unknown function	2.98	0.0080	0.0173	0.0046	1.0	8253	336	7913	484	-479
1253	PF11_0192	histone acetyltransferase, putative	3.76	0.0020	0.0055	0.0018	1.1	8253	472	7823	397	-438
801	PF10_0215	conserved Plasmodium membrane protein, unknown fun	4.52	0.0020	0.0055	0.0018	1.1	8252	325	7673	566	-311
1666	PFB0932w	Plasmodium exported protein (hyp9), unknown function	3.19	0.0060	0.0136	0.0036	1.1	8239	551	7673	737	-721
795	PF14_0198	glycine-tRNA ligase, putative	4.53	0.0020	0.0055	0.0018	1.1	8239	261	7780	445	-247
948	PF14_0365	conserved Plasmodium protein, unknown function	4.26	0.0020	0.0055	0.0018	1.2	8239	347	6951	1452	-511
1375	PFC1050w	survival of motor neuron (SMN)-like protein	3.58	0.0020	0.0055	0.0018	1.1	8234	303	7679	717	-464
1046	PFL0130c	conserved protein, unknown function	4.09	0.0020	0.0055	0.0018	1.1	8228	293	7701	582	-347
2131	PFC0875w	ABC transporter, putative	2.66	0.0040	0.0098	0.0026	1.1	8228	426	7691	922	-811
2058	PF14_0478	DNA polymerase delta interacting protein, putative	2.75	0.0080	0.0173	0.0046	1.1	8225	471	7810	624	-681
1070	PFB0555c	conserved Plasmodium membrane protein, unknown fun	4.06	0.0020	0.0055	0.0018	1.2	8222	324	7143	1274	-520
2629	PFI0320w	arginase, putative	2.11	0.0240	0.0438	0.0115	1.1	8220	803	7697	1007	-1287
1447	PF11_0465	dynamin-like protein	3.46	0.0020	0.0055	0.0018	1.0	8211	279	7847	458	-373
2477	PF10_0158	conserved Plasmodium protein, unknown function	2.28	0.0060	0.0136	0.0036	1.1	8200	378	7539	1383	-1099
1677	PF14_0185	ATP-dependent RNA helicase, putative	3.18	0.0040	0.0098	0.0026	1.1	8198	342	7569	926	-639
1097	PF10_0278	nucleolar preribosomal assembly protein, putative	4.03	0.0020	0.0055	0.0018	1.1	8190	511	7286	1013	-620
1397	MAL7P1.107	conserved Plasmodium protein, unknown function	3.54	0.0020	0.0055	0.0018	1.2	8189	594	7055	1486	-947
827	PF13_0147	conserved Plasmodium protein, unknown function	4.47	0.0020	0.0055	0.0018	1.2	8185	435	7089	1145	-483
1592	PF14_0187	glutathione S-transferase	3.28	0.0020	0.0055	0.0018	1.1	8185	652	7281	1232	-981
2141	PFF0560c	SWI/SNF-related matrix-associated actin-dependent regu	2.65	0.0120	0.0243	0.0064	1.0	8181	324	7898	447	-488
852	PF08_0036	Pfsec23	4.43	0.0020	0.0055	0.0018	1.1	8171	604	7274	853	-560
732	PF13_0297	conserved protein, unknown function	4.67	0.0020	0.0055	0.0018	1.1	8161	495	7101	1030	-465
1200	PFE1570c	conserved Plasmodium protein, unknown function	3.84	0.0020	0.0055	0.0018	1.1	8158	333	7369	966	-511
2022	MAL13P1.283	TCP-1/cpn60 chaperonin family, putative	2.78	0.0020	0.0055	0.0018	1.1	8156	269	7408	1298	-819
1756	PF11_0170	cyclophilin, putative	3.08	0.0020	0.0055	0.0018	1.1	8151	534	7515	905	-804
1262	PFD0485w	conserved Plasmodium protein, unknown function	3.75	0.0020	0.0055	0.0018	1.1	8145	304	7290	1088	-538
572	PFE1530c	XAP-5 DNA binding protein, putative	5.01	0.0020	0.0055	0.0018	1.1	8142	330	7408	662	-258
1050	PFL1940w	3-hydroxyisobutyryl-coenzyme A hydrolase, putative	4.09	0.0020	0.0055	0.0018	1.1	8127	640	7072	1145	-730
2504	PF10_0041	U5 small nuclear ribonuclear protein, putative	2.25	0.0100	0.0208	0.0055	1.1	8104	616	7586	999	-1098
1407	PF07_0107	Plasmodium exported protein, unknown function	3.53	0.0020	0.0055	0.0018	1.2	8103	1052	7032	1198	-1179
2387	PFF1350c	acetyl-CoA synthetase	2.38	0.0240	0.0438	0.0115	1.0	8100	342	7839	455	-536
1151	PFI1110w	glutamine synthetase, putative	3.92	0.0020	0.0055	0.0018	1.1	8099	494	7123	1148	-666

625	PFL1080c	ATP-binding protein, putative	4.89	0.0020	0.0055	0.0018	1.1	8098	358	7278	765	-303
1840	PFD1180w	Plasmodium exported protein (PHISTb), unknown functio	2.98	0.0040	0.0098	0.0026	1.3	8089	1514	6319	2615	-2357
497	PFL1815c	conserved Plasmodium protein, unknown function	5.19	0.0020	0.0055	0.0018	1.1	8089	382	7307	666	-265
2043	MAL13P1.299	conserved protein, unknown function	2.76	0.0040	0.0098	0.0026	1.1	8087	362	7476	1039	-790
2111	PF13_0352	conserved Plasmodium protein, unknown function	2.68	0.0100	0.0208	0.0055	1.1	8082	444	7643	711	-715
1872	PFC0900w	T-complex protein 1 epsilon subunit, putative	2.95	0.0020	0.0055	0.0018	1.2	8082	446	7015	1730	-1110
1506	PFL1800w	conserved Plasmodium protein, unknown function	3.38	0.0020	0.0055	0.0018	1.1	8079	421	7415	896	-652
960	PF13_0125	conserved Plasmodium protein, unknown function	4.25	0.0020	0.0055	0.0018	1.1	8076	549	7399	631	-503
1543	PFA0480w	phenylalanyl-tRNA synthetase, putative	3.34	0.0020	0.0055	0.0018	1.2	8060	574	6982	1507	-1003
735	PF11_0156	serine/threonine protein kinase	4.66	0.0020	0.0055	0.0018	1.1	8053	234	7463	587	-231
1235	PFL0880c	conserved Plasmodium protein, unknown function	3.79	0.0020	0.0055	0.0018	1.2	8048	543	6730	1644	-868
1873	PF11_0399		2.95	0.0020	0.0055	0.0018	1.0	8047	199	7765	438	-355
984	PF14_0247	conserved Plasmodium protein, unknown function	4.19	0.0020	0.0055	0.0018	1.1	8044	395	7037	1130	-517
1065	MAL7P1.28	rRNA/tRNA ribonuclease MRP/P subunit, putative	4.07	0.0020	0.0055	0.0018	1.1	8039	423	7463	596	-443
1376	PF10_0179	conserved Plasmodium protein, unknown function	3.58	0.0020	0.0055	0.0018	1.1	8028	476	7086	1227	-761
726	PF11_0400	conserved Plasmodium protein, unknown function	4.68	0.0020	0.0055	0.0018	1.1	8021	277	7582	396	-234
1591	PFL1560c	conserved protein, unknown function	3.28	0.0040	0.0098	0.0026	1.1	8011	384	7582	552	-508
1662	PFC0425w	conserved Plasmodium protein, unknown function	3.20	0.0060	0.0136	0.0036	1.1	8008	690	6966	1488	-1137
1982	PF10_0015	acyl-CoA binding protein, isoform 1, ACBP1	2.84	0.0080	0.0173	0.0046	1.3	8007	1990	5962	3113	-3057
1003	PFE0430w	ATP-dependent RNA helicase, putative	4.16	0.0020	0.0055	0.0018	1.1	8007	413	7273	791	-470
1309	PF14_0677	RNA 3'-terminal phosphate cyclase-like protein, putative	3.67	0.0020	0.0055	0.0018	1.1	8006	277	7162	1104	-537
1825	PFD1135c	probable protein, unknown function	3.00	0.0040	0.0098	0.0026	1.1	8004	788	7132	1259	-1175
1284	MAL8P1.114	conserved Plasmodium protein, unknown function	3.70	0.0020	0.0055	0.0018	1.1	7999	382	7260	925	-568
2273	MAL7P1.139	mago nashi protein homolog, putative	2.50	0.0100	0.0208	0.0055	1.1	7998	423	7546	810	-782
1133	PF11_0211	alpha/beta hydrolase fold domain containing protein, put	3.95	0.0020	0.0055	0.0018	1.2	7997	744	6847	1281	-876
2499	MAL13P1.56	M1-family alanyl aminopeptidase	2.26	0.0100	0.0208	0.0055	1.1	7994	435	7391	1255	-1087
582	PFC0545c	conserved Plasmodium protein, unknown function	4.99	0.0020	0.0055	0.0018	1.2	7992	407	6892	1025	-332
2114	PF10_0067	conserved Plasmodium protein, unknown function	2.68	0.0080	0.0173	0.0046	1.1	7979	453	7511	767	-751
2254	PFI1040c	conserved Plasmodium membrane protein, unknown fun	2.52	0.0080	0.0173	0.0046	1.1	7971	269	7372	1143	-812
2381	MAL8P1.58	phosphatidylglycerophosphate synthase	2.39	0.0259	0.0469	0.0123	1.0	7969	354	7637	613	-635
1971	MAL8P1.19	DEAD/DEAH box ATP-dependent RNA helicase, putative	2.85	0.0040	0.0098	0.0026	1.1	7968	329	7089	1485	-935
2492	PFA0230c	conserved Plasmodium protein, unknown function	2.26	0.0140	0.0277	0.0073	1.1	7966	928	7126	1641	-1729
1308	PFL0900c	arginyl-tRNA synthetase, putative	3.67	0.0020	0.0055	0.0018	1.1	7965	470	7188	960	-653
612	PFB0415c	3'exoribonuclease, putative	4.92	0.0020	0.0055	0.0018	1.1	7961	389	7075	819	-321
1114	PFC0240c	conserved Plasmodium membrane protein, unknown fun	3.99	0.0020	0.0055	0.0018	1.1	7957	662	7052	961	-719
1888	PF14_0218	actin-related protein homolog, arp4 homolog	2.93	0.0020	0.0055	0.0018	1.1	7956	450	7251	1115	-860
2044	PFE0625w	Rab GTPase 1b	2.76	0.0060	0.0136	0.0036	1.1	7946	355	7460	809	-678
349	PF11_0231	conserved Plasmodium protein, unknown function	5.59	0.0020	0.0055	0.0018	1.3	7937	452	6218	1457	-190
1936	PFD0740w	cdc2-related protein kinase 3	2.88	0.0040	0.0098	0.0026	1.0	7937	391	7582	506	-542
521	PFL2530w	lysophospholipase, putative	5.11	0.0020	0.0055	0.0018	1.2	7932	629	6351	1421	-469
1488	MAL8P1.64	conserved Plasmodium protein, unknown function	3.40	0.0020	0.0055	0.0018	1.1	7926	558	7101	1091	-825
1057	PF14_0401	tRNA binding protein, putative	4.08	0.0020	0.0055	0.0018	1.1	7918	617	6983	998	-679
2127	PFB0285c	conserved Plasmodium protein, unknown function	2.66	0.0120	0.0243	0.0064	1.1	7901	808	6974	1563	-1445

1630	PFB0194w	conserved Plasmodium protein, unknown function	3.23	0.0020	0.0055	0.0018	1.1	7892	481	7036	1234	-858
1916	PFF0920c		2.91	0.0020	0.0055	0.0018	1.1	7885	489	7207	1066	-877
1344	PFE1020w	U6 snRNA-associated sm-like protein lsm2, putative	3.63	0.0020	0.0055	0.0018	1.2	7880	363	6839	1372	-693
2391	PF14_0574	conserved Plasmodium protein, unknown function	2.37	0.0180	0.0344	0.0090	1.1	7878	664	7245	1183	-1213
2460	PFC0282w	conserved Plasmodium protein, unknown function	2.30	0.0200	0.0376	0.0099	1.1	7876	437	7367	1023	-950
1038	PFL2355w	conserved Plasmodium protein, unknown function	4.11	0.0020	0.0055	0.0018	1.2	7871	415	6592	1486	-622
1703	PF07_0073	seryl-tRNA synthetase, putative	3.15	0.0020	0.0055	0.0018	1.1	7870	461	7235	909	-735
2032	PFF1070c	radical SAM protein, putative	2.78	0.0040	0.0098	0.0026	1.1	7865	376	6938	1602	-1052
1622	MAL7P1.104	3'-5' exoribonuclease Csl4 homolog, putative	3.24	0.0080	0.0173	0.0046	1.1	7863	472	7468	446	-523
421	PF11_0201	ubiquitin-protein ligase, putative	5.40	0.0020	0.0055	0.0018	1.1	7862	252	7032	723	-145
1113	PFL0075w	XPA binding protein 1, putative	3.99	0.0020	0.0055	0.0018	1.1	7861	418	6840	1202	-599
1158	PFL0520c	conserved Plasmodium protein, unknown function	3.90	0.0020	0.0055	0.0018	1.1	7861	525	7115	827	-605
1803	PFL0670c	bifunctional aminoacyl-tRNA synthetase, putative	3.03	0.0020	0.0055	0.0018	1.1	7858	340	7340	789	-611
2654	PF08_0048	Snf2-related CBP activator, putative	2.08	0.0240	0.0438	0.0115	1.0	7854	391	7534	678	-749
1783	PF13_0309	U3 snoRNA-associated small subunit rRNA processing prc	3.05	0.0060	0.0136	0.0036	1.1	7852	330	7402	666	-546
479	PF10_0098	conserved Plasmodium protein, unknown function	5.22	0.0020	0.0055	0.0018	1.3	7851	761	6171	1440	-521
1252	PF13_0259	cytidine and deoxycytidylate deaminase, putative	3.76	0.0020	0.0055	0.0018	1.1	7850	644	6976	1001	-771
1508	PFC0865w	RNA binding protein, putative	3.38	0.0020	0.0055	0.0018	1.1	7849	363	6956	1259	-729
1201	MAL13P1.172	conserved protein, unknown function	3.84	0.0020	0.0055	0.0018	1.2	7844	719	6621	1439	-934
2055	PF10_0296	conserved Plasmodium protein, unknown function	2.75	0.0080	0.0173	0.0046	1.2	7843	1028	6667	1908	-1760
299	PF13_0318	RNA binding protein, putative	5.78	0.0020	0.0055	0.0018	1.1	7842	423	6902	713	-196
1368	PF13_0150	DNA-directed RNA polymerase 3 largest subunit	3.59	0.0020	0.0055	0.0018	1.1	7839	381	6988	1117	-647
478	MAL13P1.116	conserved Plasmodium membrane protein, unknown fun	5.23	0.0020	0.0055	0.0018	1.1	7836	329	6827	905	-224
875	PFI1130c	DNA-directed RNA polymerase II, putative	4.39	0.0020	0.0055	0.0018	1.1	7836	222	7380	473	-239
2142	PF11_0433	conserved Plasmodium protein, unknown function	2.65	0.0060	0.0136	0.0036	1.2	7826	659	6789	1838	-1460
738	PFI0635c	conserved Plasmodium protein, unknown function	4.66	0.0020	0.0055	0.0018	1.2	7825	328	6789	1053	-346
283	PF14_0372	conserved Plasmodium protein, unknown function	5.83	0.0020	0.0055	0.0018	1.1	7817	288	6965	674	-111
881	PF11_0252	neutral-sphingomyelinase activation factor protein, putat	4.38	0.0020	0.0055	0.0018	1.2	7815	328	6670	1249	-433
1367	PF13_0261	nucleolar preribosomal associated cytoplasmic ATPase, p	3.59	0.0020	0.0055	0.0018	1.1	7800	415	7254	659	-528
2011	PFD0750w	nuclear cap-binding protein, putative	2.80	0.0040	0.0098	0.0026	1.1	7799	364	7306	809	-679
1134	PFE1220w	conserved Plasmodium protein, unknown function	3.95	0.0020	0.0055	0.0018	1.1	7788	332	7335	488	-367
751	PF13_0063	26S proteasome regulatory subunit 7, putative	4.63	0.0020	0.0055	0.0018	1.2	7769	344	6580	1226	-380
390	PFL0175c	conserved Plasmodium protein, unknown function	5.48	0.0020	0.0055	0.0018	1.3	7762	440	5929	1598	-204
1178	PF13_0157	ribose-phosphate pyrophosphokinase, putative	3.88	0.0020	0.0055	0.0018	1.1	7752	348	7091	781	-469
267	PFL2455w	conserved Plasmodium protein, unknown function	5.91	0.0020	0.0055	0.0018	1.3	7748	357	6076	1354	-39
244	PFE1270c	WD domain, G-beta repeat-containing protein	6.04	0.0020	0.0055	0.0018	1.1	7743	303	7006	540	-106
956	PFE0730c	ribose 5-phosphate epimerase, putative	4.25	0.0020	0.0055	0.0018	1.1	7740	547	6800	983	-588
1484	PFC0350c	TCP-1/cpn60 chaperonin family, putative	3.40	0.0020	0.0055	0.0018	1.1	7736	439	6882	1172	-758
1398	PFF0970w	splicing factor 3a subunit, putative	3.54	0.0020	0.0055	0.0018	1.1	7730	364	7230	620	-484
850	PFL1520w	DIM1 protein homolog, putative	4.43	0.0020	0.0055	0.0018	1.1	7726	346	6974	778	-373
1515	PF11_0439	conserved protein, unknown function	3.37	0.0020	0.0055	0.0018	1.1	7725	452	6986	1005	-718
1962	PF13_0174	sedlin, putative	2.86	0.0060	0.0136	0.0036	1.1	7722	647	7244	614	-783
2241	MAL8P1.202	apicoplast phosphatidic acid phosphatase, putative	2.53	0.0140	0.0277	0.0073	1.1	7722	462	7147	1044	-931

814	PFL0155c	conserved Plasmodium protein, unknown function	4.49	0.0020	0.0055	0.0018	1.3	7722	533	6070	1745	-626
1821	PFL0450c	conserved Plasmodium protein, unknown function	3.01	0.0060	0.0136	0.0036	1.1	7719	830	6740	1436	-1286
601	PF14_0364	cleavage and polyadenylation specifity factor, putative	4.95	0.0020	0.0055	0.0018	1.1	7717	249	6950	730	-211
924	PFF0515c	conserved protein, unknown function	4.32	0.0020	0.0055	0.0018	1.2	7712	948	6393	1267	-896
880	PFE0160c	Ser/Arg-rich splicing factor, putative	4.39	0.0020	0.0055	0.0018	1.2	7708	478	6415	1387	-572
1606	MAL13P1.93	small ribosomal subunit nuclear export protein, putative	3.26	0.0020	0.0055	0.0018	1.1	7707	355	7045	949	-642
1024	PF14_0464	SNARE protein, putative	4.13	0.0020	0.0055	0.0018	1.1	7699	288	6754	1095	-439
447	PF11_0048	casein kinase II beta chain	5.32	0.0020	0.0055	0.0018	1.2	7695	583	6425	1061	-374
870	PF10_0326	WD-repeat protein, putative	4.40	0.0020	0.0055	0.0018	1.3	7694	324	6154	1691	-475
686	PF10_0023	Plasmodium exported protein (hyp16), unknown functior	4.75	0.0020	0.0055	0.0018	1.3	7690	605	5823	1856	-595
1167	PF14_0042	U3 small nucleolar ribonucleoprotein, U3 snoRNP, putati	3.89	0.0020	0.0055	0.0018	1.1	7688	319	6861	1005	-497
1546	PFL0135w	conserved Plasmodium protein, unknown function	3.33	0.0020	0.0055	0.0018	1.1	7684	303	6793	1285	-696
832	PF14_0528	hemolysin, putative	4.46	0.0020	0.0055	0.0018	1.2	7666	539	6661	1006	-540
1641	MAL7P1.178	alpha/beta hydrolase, putative	3.22	0.0020	0.0055	0.0018	1.3	7665	1629	5861	2378	-2202
2121	PFD0875c	conserved Plasmodium protein, unknown function	2.67	0.0080	0.0173	0.0046	1.1	7665	949	7044	813	-1142
1932	PF11_0071	RuvB DNA helicase, putative	2.88	0.0020	0.0055	0.0018	1.1	7664	521	6984	1069	-911
1621	PF08_0081	conserved Plasmodium protein, unknown function	3.24	0.0020	0.0055	0.0018	1.1	7660	648	6662	1405	-1055
966	PF10_0197	methyltransferase, putative	4.22	0.0020	0.0055	0.0018	1.1	7656	354	7118	549	-365
859	PFD0250c	Sec24 subunit b	4.42	0.0020	0.0055	0.0018	1.1	7652	293	7087	576	-303
673	PF11_0115	conserved Plasmodium protein, unknown function	4.77	0.0020	0.0055	0.0018	1.2	7650	425	6357	1277	-410
615	PFE0305w	transcription initiation factor TFiid, TATA-binding protein	4.91	0.0020	0.0055	0.0018	1.3	7647	755	5787	1745	-640
631	PFB0270w	iron-sulfur assembly protein, putative	4.87	0.0020	0.0055	0.0018	1.2	7643	489	6590	978	-413
1789	PF14_0760	Plasmodium exported protein, unknown function	3.05	0.0040	0.0098	0.0026	1.2	7641	1042	6466	1674	-1541
944	PF10_0078	histone deacetylase, putative	4.27	0.0020	0.0055	0.0018	1.2	7633	271	6178	1655	-471
785	PF14_0604	conserved Plasmodium protein, unknown function	4.56	0.0020	0.0055	0.0018	1.3	7632	749	5663	2020	-799
586	PF10_0074	conserved Plasmodium protein, unknown function	4.98	0.0020	0.0055	0.0018	1.3	7631	379	6050	1521	-320
1162	PF11_0436	coproporphyrinogen III oxidase	3.90	0.0020	0.0055	0.0018	1.1	7627	352	6986	749	-460
410	PF11_0144	Rpr2, RNAse P, putative	5.42	0.0020	0.0055	0.0018	1.1	7626	337	6934	557	-202
1492	MAL13P1.249	conserved Plasmodium protein, unknown function	3.40	0.0020	0.0055	0.0018	1.1	7625	518	6913	931	-736
737	PF10_0245	glucosamine-fructose-6-phosphate aminotransferase, pu	4.66	0.0020	0.0055	0.0018	1.4	7622	815	5564	2052	-809
1494	PF14_0576	ubiquitin carboxyl-terminal hydrolase isozyme L3	3.40	0.0040	0.0098	0.0026	1.1	7616	635	7039	638	-696
1021	PF14_0137	DnaJ protein, putative	4.14	0.0020	0.0055	0.0018	1.1	7609	248	7190	450	-279
970	PFF0490w	conserved Plasmodium protein, unknown function	4.22	0.0020	0.0055	0.0018	1.2	7605	761	6594	983	-734
1925	PF10_0089	small subunit rRNA synthesis-associated protein, putative	2.89	0.0100	0.0208	0.0055	1.1	7603	665	6979	897	-938
1251	PF10_0311	protein phosphatase inhibitor, putative	3.76	0.0020	0.0055	0.0018	1.1	7596	672	6958	610	-644
608	PF13_0177	DEAD/DEAH box ATP-dependent RNA helicase, putative	4.94	0.0020	0.0055	0.0018	1.1	7591	361	6774	751	-296
1628	PF07_0097	conserved Plasmodium protein, unknown function	3.24	0.0040	0.0098	0.0026	1.1	7589	493	6840	1054	-798
2611	PF11_0293	multiprotein bridging factor type 1, putative	2.13	0.0220	0.0408	0.0107	1.1	7584	481	7000	1277	-1174
1509	PF10_0162	Plasmodium exported protein (PHISTc), unknown functio	3.38	0.0020	0.0055	0.0018	1.2	7574	845	6417	1521	-1209
2277	PF10_0209	RNA helicase, putative	2.50	0.0120	0.0243	0.0064	1.1	7563	384	7186	664	-671
811	MAL8P1.204	DnaJ protein, putative	4.50	0.0020	0.0055	0.0018	1.2	7562	565	6069	1554	-625
1886	PF14_0107	conserved Plasmodium protein, unknown function	2.93	0.0040	0.0098	0.0026	1.1	7561	357	7034	827	-657
1994	PF11_0068	conserved protein, unknown function	2.82	0.0040	0.0098	0.0026	1.1	7557	544	6950	951	-888

2380	PFB0375w	conserved Plasmodium protein, unknown function	2.39	0.0100	0.0208	0.0055	1.1	7547	297	7117	846	-713
1699	PF10_0022	Plasmodium exported protein (PHISTc), unknown functio	3.15	0.0060	0.0136	0.0036	1.4	7546	1418	5448	3038	-2358
1103	PF14_0316	DNA topoisomerase II, putative	4.02	0.0020	0.0055	0.0018	1.3	7533	864	5632	2200	-1163
1137	PF08_0121	peptidyl-prolyl cis-trans isomerase precursor	3.95	0.0020	0.0055	0.0018	1.3	7526	695	5760	2114	-1043
1536	PFL2365w	protein phosphatase, putative	3.34	0.0020	0.0055	0.0018	1.1	7513	675	6871	750	-783
1973	PFD0730w	conserved Plasmodium protein, unknown function	2.85	0.0020	0.0055	0.0018	1.1	7508	524	6764	1201	-981
2237	PFC0235w	conserved Plasmodium protein, unknown function	2.54	0.0020	0.0055	0.0018	1.1	7502	290	7068	803	-659
1136	PFI0970c	TLD domain containing protein	3.95	0.0020	0.0055	0.0018	1.1	7489	781	6527	998	-817
1127	PF10_0024	Plasmodium exported protein (hyp2), unknown function	3.97	0.0020	0.0055	0.0018	1.4	7486	1154	5347	2457	-1472
130	PF08_0092	histone-arginine methyltransferase, putative	6.79	0.0020	0.0055	0.0018	1.3	7481	628	5662	1203	-12
1161	PF13_0175	conserved protein, unknown function	3.90	0.0020	0.0055	0.0018	1.2	7475	684	6257	1417	-883
2031	PF14_0559	conserved Plasmodium protein, unknown function	2.78	0.0060	0.0136	0.0036	1.1	7468	384	7073	617	-606
1406	PF14_0256	exosome complex exonuclease rrp41, putative	3.53	0.0020	0.0055	0.0018	1.1	7464	588	6784	805	-713
167	MAL8P1.151	inositol phosphatase, putative	6.50	0.0020	0.0055	0.0018	1.2	7459	603	6193	809	-146
713	PF14_0520	6-phosphogluconate dehydrogenase, decarboxylating, pu	4.70	0.0020	0.0055	0.0018	1.3	7458	700	5670	1767	-679
2536	PFB0710c	conserved Plasmodium protein, unknown function	2.22	0.0200	0.0376	0.0099	1.1	7456	328	6960	1058	-890
1331	PF13_0162	conserved Plasmodium protein, unknown function	3.64	0.0020	0.0055	0.0018	1.2	7455	806	6372	1289	-1012
2372	PF11_0163	transcription factor with AP2 domain(s), putative	2.40	0.0220	0.0408	0.0107	1.1	7450	1031	6650	1385	-1616
1329	PFE0300c	60S ribosomal subunit protein L24-2, putative	3.64	0.0020	0.0055	0.0018	1.2	7448	646	6294	1453	-946
1291	PF11_0184	DNA mismatch repair protein MLH1, putative	3.69	0.0020	0.0055	0.0018	1.1	7431	554	6715	830	-667
1912	PFE0295w	conserved Plasmodium protein, unknown function	2.91	0.0040	0.0098	0.0026	1.1	7426	516	6907	759	-756
576	PFI0695c	phospholipid or glycerol acyltransferase, putative	5.00	0.0020	0.0055	0.0018	1.2	7418	608	6378	883	-451
722	PF11_0359	coatomer delta subunit, putative	4.69	0.0020	0.0055	0.0018	1.1	7418	524	6717	585	-408
2419	PFA0245w	transporter, putative	2.34	0.0259	0.0469	0.0123	1.1	7416	746	6755	1234	-1319
1226	PF10_0033	conserved Plasmodium protein, unknown function	3.80	0.0020	0.0055	0.0018	1.3	7405	883	5729	2027	-1235
347	MAL13P1.216	DNA helicase, putative	5.60	0.0020	0.0055	0.0018	1.4	7403	1024	5347	1578	-547
1483	MAL8P1.108	protein phosphatase, putative	3.40	0.0020	0.0055	0.0018	1.1	7401	461	6803	768	-631
818	PF13_0051	small nucleolar ribonucleoprotein, putative	4.49	0.0020	0.0055	0.0018	1.2	7395	348	6097	1386	-436
568	PF08_0044	protein kinase 1	5.01	0.0020	0.0055	0.0018	1.4	7373	805	5456	1746	-635
209	PFE1400c	beta adaptin protein, putative	6.22	0.0020	0.0055	0.0018	1.2	7368	396	6323	753	-104
1713	PFE1390w	RNA helicase 1	3.13	0.0020	0.0055	0.0018	1.1	7368	490	6759	859	-740
1391	PFL0705c	adrenodoxin-type ferredoxin, putative	3.55	0.0020	0.0055	0.0018	1.2	7363	727	6216	1458	-1039
1195	PF08_0083	conserved Plasmodium protein, unknown function	3.85	0.0020	0.0055	0.0018	1.2	7356	714	6309	1189	-857
1659	PFL1855w	cell cycle control protein, putative	3.20	0.0020	0.0055	0.0018	1.1	7351	399	6862	669	-579
1350	PF14_0473	Rrp6 homologue, putative	3.62	0.0020	0.0055	0.0018	1.1	7344	527	6616	879	-679
767	PF14_0620	tRNA 3???-trailer sequence RNase, putative	4.60	0.0020	0.0055	0.0018	1.2	7342	396	6235	1131	-421
667	PFC0365w	PRP19-like protein, putative	4.78	0.0020	0.0055	0.0018	1.2	7329	536	6052	1229	-487
535	PF14_0589	valine-tRNA ligase, putative	5.08	0.0020	0.0055	0.0018	1.2	7324	458	5884	1335	-354
1918	PF14_0246	phosphoenolpyruvate carboxylase, putative	2.91	0.0060	0.0136	0.0036	1.1	7323	360	6785	854	-677
1168	PF10_0244	formin 2, putative	3.89	0.0020	0.0055	0.0018	1.2	7320	749	6140	1344	-913
1647	PF14_0540	conserved Plasmodium protein, unknown function	3.21	0.0020	0.0055	0.0018	1.2	7320	589	6153	1708	-1131
2516	PFF0420c	proteasome subunit alpha type 2, putative	2.24	0.0180	0.0344	0.0090	1.1	7319	859	6667	1224	-1433
418	PF10_0161a	Plasmodium exported protein, unknown function	5.40	0.0020	0.0055	0.0018	1.3	7317	550	5736	1357	-325

2677	PFF0340c	glutaredoxin-like protein, putative	2.06	0.0160	0.0311	0.0082	1.1	7314	431	6616	1620	-1354		
2332	PF13_0020	conserved Plasmodium protein, unknown function	2.44	0.0180	0.0344	0.0090	1.1	7311	409	6820	923	-842		
2224	PFL2145w	conserved Plasmodium protein, unknown function	2.55	0.0200	0.0376	0.0099	1.1	7311	560	6787	888	-925		
1928	PFI0105c	serine/threonine protein kinase, FIKK family	2.89	0.0140	0.0277	0.0073	1.1	7310	763	6466	1280	-1199		
1123	PFL1270w	cof-like hydrolase, had-superfamily, subfamily iib	3.97	0.0020	0.0055	0.0018	1.3	7308	1219	5493	1991	-1394		
739	PFL1735c	RNA-processing protein, putative	4.66	0.0020	0.0055	0.0018	1.3	7306	539	5710	1617	-560		
747	MAL8P1.43	U5 snrnp-specific protein, putative	4.64	0.0020	0.0055	0.0018	1.2	7304	336	6092	1250	-373		
1408	PFE0090w	chromosome assembly factor 1	3.52	0.0020	0.0055	0.0018	1.2	7300	1079	5967	1617	-1362		
2120	PF14_0686	conserved Plasmodium protein, unknown function	2.67	0.0100	0.0208	0.0055	1.1	7293	818	6360	1566	-1452		
1273	PFC0100c	golgi organization and biogenesis factor, putative	3.73	0.0020	0.0055	0.0018	1.2	7275	634	6049	1521	-929		
2295	MAL7P1.68	zinc finger protein, putative	2.48	0.0060	0.0136	0.0036	1.1	7272	539	6748	927	-943		
1317	PF10_0132	phosphoinositide specific phospholipase c, putative	3.66	0.0020	0.0055	0.0018	1.1	7271	538	6635	722	-623		
1540	PF08_0086	RNA binding protein, putative	3.34	0.0020	0.0055	0.0018	1.2	7267	574	6037	1738	-1081		
1176	PF10_0054	conserved protein, unknown function	3.88	0.0020	0.0055	0.0018	1.1	7265	412	6595	771	-513		
1565	PF14_0197	zinc finger protein, putative	3.31	0.0020	0.0055	0.0018	1.1	7264	411	6467	1127	-741		
2242	PFI1775w	lysophospholipase, putative	2.53	0.0160	0.0311	0.0082	1.1	7257	461	6733	940	-877		
2099	PF11_0151		2.70	0.0140	0.0277	0.0073	1.1	7254	735	6530	1161	-1171		
2089	PFD1185w	Plasmodium exported protein (PHISTa), unknown functio	2.71	0.0140	0.0277	0.0073	1.1	7252	1063	6307	1456	-1574		
1760	PFE0335w	conserved Plasmodium protein, unknown function	3.08	0.0060	0.0136	0.0036	1.1	7250	365	6838	579	-532		
1462	MAL7P1.146	conserved Plasmodium membrane protein, unknown fun	3.44	0.0040	0.0098	0.0026	1.1	7246	403	6533	957	-648		
690	PF14_0552	conserved Plasmodium protein, unknown function	4.75	0.0020	0.0055	0.0018	1.2	7242	336	6227	1009	-330		
2136	PF10_0319	conserved Plasmodium protein, unknown function	2.65	0.0080	0.0173	0.0046	1.1	7238	283	6600	1153	-798		
1275	PFB0715w	DNA-directed RNA polymerase II second largest subunit,	3.73	0.0020	0.0055	0.0018	1.1	7234	321	6698	652	-437		
2213	PFI0920c	dihydrouridine synthase, putative	2.56	0.0160	0.0311	0.0082	1.1	7228	413	6878	574	-636		
1634	MAL7P1.22	conserved Plasmodium protein, unknown function	3.23	0.0060	0.0136	0.0036	1.3	7222	1606	5445	2338	-2166		
698	MAL8P1.41	RWD domain-containing protein, putative	4.73	0.0020	0.0055	0.0018	1.1	7213	528	6444	663	-421		
392	PFF1240w	poly(A) polymerase PAP, putative	5.48	0.0020	0.0055	0.0018	1.2	7212	594	5773	1187	-342		
200	PFC0690c	conserved Plasmodium protein, unknown function	6.27	0.0020	0.0055	0.0018	1.2	7211	538	5876	940	-143		
854	PF13_0186	conserved Plasmodium protein, unknown function	4.43	0.0020	0.0055	0.0018	1.2	7207	514	5767	1534	-608		
1192	PF10_0294	RNA helicase, putative	3.86	0.0020	0.0055	0.0018	1.2	7205	482	5789	1753	-819		
1157	MAL13P1.337	Skp1 family protein, putative	3.90	0.0020	0.0055	0.0018	1.2	7195	759	6094	1227	-884		
2483	MAL13P1.270	proteasome subunit, putative	2.27	0.0240	0.0438	0.0115	1.1	7195	926	6365	1611	-1707		
652	PF10_0136	initiation factor 2 subunit family, putative	4.81	0.0020	0.0055	0.0018	1.5	7189	968	4872	2213	-864		
2539	PF14_0734	serine/threonine protein kinase, FIKK family	2.22	0.0180	0.0344	0.0090	1.2	7189	2397	5840	2193	-3241		
957	PF14_0476	serine/threonine protein kinase, putative	4.25	0.0040	0.0098	0.0026	1.1	7186	560	6437	723	-535		
718	PF11_0463	coatamer gamma subunit, putative	4.69	0.0020	0.0055	0.0018	1.2	7182	375	5939	1260	-391		
1126	MAL13P1.26	conserved Plasmodium protein, unknown function	3.97	0.0020	0.0055	0.0018	1.2	7173	384	5972	1447	-630		
1798	PF14_0145	ubiquitin carboxyl-terminal hydrolase, putative	3.03	0.0040	0.0098	0.0026	1.2	7166	569	6088	1675	-1165		
867	PFC0410w	YT521-B-like family protein, putative	4.41	0.0020	0.0055	0.0018	1.1	7162	408	6564	568	-379		
1675	MAL8P1.160	Plasmodium exported protein (hyp7), unknown function	3.18	0.0020	0.0055	0.0018	1.3	7161	1784	5357	2342	-2322		
891	PF14_0221	GTPase, putative	4.37	0.0020	0.0055	0.0018	1.1	7155	361	6427	757	-391		
335	PFF0790c	conserved Plasmodium protein, unknown function	5.66	0.0020	0.0055	0.0018	1.3	7146	566	5628	1224	-273		
1851	PF10_0077	eukaryotic translation initiation factor 3 subunit 7, putati	2.97	0.0020	0.0055	0.0018	1.2	7145	481	6129	1625	-1090		

711	PF13_0158	nonsense-mediated mRNA decay protein Upf3, putative	4.71	0.0020	0.0055	0.0018	1.2	7140	404	5730	1429	-422
461	PF10_0269	DNA-directed RNA polymerase II, putative	5.28	0.0020	0.0055	0.0018	1.3	7138	595	5599	1337	-393
1089	MAL13P1.174	MSP7-like protein	4.03	0.0020	0.0055	0.0018	1.2	7138	758	6020	1200	-839
666	PF07_0034	Cg3 protein	4.78	0.0020	0.0055	0.0018	1.3	7134	443	5390	1747	-445
511	PFI0825w	conserved Plasmodium protein, unknown function	5.15	0.0020	0.0055	0.0018	1.3	7132	621	5327	1638	-453
502	MAL13P1.322	splicing factor, putative	5.18	0.0020	0.0055	0.0018	1.2	7128	392	5879	1136	-278
1366	PFL0160w	signal recognition particle SRP14	3.59	0.0020	0.0055	0.0018	1.1	7127	623	6273	1041	-810
759	PF14_0636	conserved Plasmodium protein, unknown function	4.61	0.0020	0.0055	0.0018	1.3	7127	419	5694	1482	-468
1338	PFD0525w	conserved Plasmodium protein, unknown function	3.64	0.0020	0.0055	0.0018	1.3	7127	743	5664	1870	-1149
1243	MAL13P1.225	thioredoxin 2	3.78	0.0020	0.0055	0.0018	1.2	7116	767	5882	1466	-999
671	PF10_0128	U3 snoRNA-associated small subunit rRNA processing prc	4.78	0.0020	0.0055	0.0018	1.2	7112	500	5689	1397	-475
1139	PF13_0348	rhoptry protein	3.94	0.0020	0.0055	0.0018	1.2	7106	476	6142	1129	-641
272	PF08_0130	rRNA processing WD-repeat protein, putative	5.87	0.0020	0.0055	0.0018	1.2	7101	249	6083	823	-55
1779	MAL7P1.7	Plasmodium exported protein (PHISTb), unknown functio	3.06	0.0020	0.0055	0.0018	1.5	7097	2498	4851	2920	-3172
694	PFC0635c	translation initiation factor 4E	4.74	0.0020	0.0055	0.0018	1.3	7096	739	5353	1691	-687
2227	PFC0780w	CPSF (cleavage and polyadenylation specific factor), subu	2.55	0.0080	0.0173	0.0046	1.1	7091	431	6622	825	-787
865	PF08_0120	GTPase activator, putative	4.41	0.0020	0.0055	0.0018	1.1	7088	450	6299	791	-452
468	MAL7P1.112	conserved Plasmodium protein, unknown function	5.26	0.0020	0.0055	0.0018	1.2	7086	457	5890	1045	-306
1906	PFF0485c	zinc finger protein, putative	2.92	0.0100	0.0208	0.0055	1.2	7078	791	6106	1491	-1311
1265	PFI1025w	RNA binding protein, putative	3.74	0.0020	0.0055	0.0018	1.2	7076	642	5987	1321	-874
2316	PFL1610c	conserved Plasmodium membrane protein, unknown fun	2.46	0.0080	0.0173	0.0046	1.1	7073	560	6466	1114	-1067
1241	PFD0207c	conserved Plasmodium protein, unknown function	3.78	0.0020	0.0055	0.0018	1.1	7073	553	6330	844	-654
2246	PF14_0738	lysophospholipase, putative	2.52	0.0220	0.0408	0.0107	1.2	7073	1334	5896	1990	-2148
263	PF13_0142	lsm6 homologue, putative	5.95	0.0020	0.0055	0.0018	1.4	7070	679	5002	1604	-215
2065	PF10_0091	zinc finger protein, putative	2.74	0.0140	0.0277	0.0073	1.1	7065	415	6682	591	-622
414	MAL13P1.294	GTP binding protein, putative	5.41	0.0020	0.0055	0.0018	1.3	7065	467	5537	1327	-265
2228	PFL1195w	conserved Plasmodium protein, unknown function	2.55	0.0180	0.0344	0.0090	1.1	7055	1004	6215	1375	-1540
1682	PFL1830w	ubiquitin-like protein, putative	3.17	0.0040	0.0098	0.0026	1.2	7054	372	6093	1451	-862
1100	PF11_0363	conserved Plasmodium membrane protein, unknown fun	4.02	0.0020	0.0055	0.0018	1.2	7052	554	6006	1186	-693
1188	PF07_0045	conserved Plasmodium protein, unknown function	3.86	0.0020	0.0055	0.0018	1.2	7049	529	5932	1345	-758
2306	PF13_0136	conserved Plasmodium protein, unknown function	2.47	0.0020	0.0055	0.0018	1.1	7047	424	6358	1321	-1056
2374	PFA0215w		2.39	0.0259	0.0469	0.0123	1.1	7042	956	6394	1055	-1363
458	PF10_0299	glycoprotease, putative	5.30	0.0020	0.0055	0.0018	1.3	7030	720	5476	1302	-468
997	PFI1115c	pre-mRNA splicing factor, putative	4.17	0.0020	0.0055	0.0018	1.1	7028	462	6169	932	-535
1256	PFB0410c	phospholipase A2, putative	3.76	0.0020	0.0055	0.0018	1.1	7022	601	6267	844	-690
1562	MAL13P1.14	DEAD/DEAH box ATP-dependent RNA helicase, putative	3.32	0.0040	0.0098	0.0026	1.1	7013	581	6343	860	-771
959	PF07_0046	mitochondrial ribosomal protein L1 precursor, putative	4.25	0.0020	0.0055	0.0018	1.2	7010	527	5737	1400	-653
676	PF11_0212	tRNA nucleotidyltransferase, putative	4.77	0.0020	0.0055	0.0018	1.2	7007	571	5912	1016	-492
1051	MAL13P1.222	conserved Plasmodium protein, unknown function	4.08	0.0020	0.0055	0.0018	1.1	6999	387	6113	1012	-513
845	PFC0600w	conserved Plasmodium protein, unknown function	4.44	0.0020	0.0055	0.0018	1.2	6997	490	5661	1416	-570
1988	PF08_0127	conserved Plasmodium protein, unknown function	2.82	0.0100	0.0208	0.0055	1.1	6996	645	6354	974	-977
285	PFD0725c	arsenical pump-driving ATPase, putative	5.82	0.0020	0.0055	0.0018	1.2	6993	376	5902	861	-147
1780	MAL8P1.105	conserved protein, unknown function	3.06	0.0020	0.0055	0.0018	1.1	6985	363	6270	1103	-751

1956	PFL1905w	conserved Plasmodium protein, unknown function	2.86	0.0100	0.0208	0.0055	1.1	6982	503	6382	934	-837
904	PFB0732c	conserved Plasmodium protein, unknown function	4.35	0.0020	0.0055	0.0018	1.2	6970	1144	5728	1016	-918
1587	PF14_0060	glycerophodiester phosphodiesterase, putative	3.29	0.0040	0.0098	0.0026	1.1	6958	570	6239	960	-810
1505	PFC0060c	serine/threonine protein kinase, FIKK family	3.38	0.0060	0.0136	0.0036	1.2	6956	980	5792	1472	-1288
1672	PF08_0124	conserved Plasmodium protein, unknown function	3.18	0.0020	0.0055	0.0018	1.1	6955	275	6559	564	-443
920	PFA0475c	conserved protein, unknown function	4.32	0.0020	0.0055	0.0018	1.2	6954	512	5941	1066	-564
2023	PF11_0232	conserved Plasmodium protein, unknown function	2.78	0.0020	0.0055	0.0018	1.1	6936	393	6431	825	-713
1310	PFD0905w	conserved Plasmodium protein, unknown function	3.66	0.0020	0.0055	0.0018	1.2	6933	724	5889	1256	-936
522	PFF1040w	conserved Plasmodium protein, unknown function	5.11	0.0020	0.0055	0.0018	1.3	6932	701	5201	1553	-523
864	PF14_0159	root hair defective 3 GTP-binding protein (RHD3) homolo	4.41	0.0020	0.0055	0.0018	1.1	6932	487	6050	890	-496
1072	MAL7P1.133		4.05	0.0020	0.0055	0.0018	1.1	6932	463	6048	995	-574
523	PF11_0116	tRNA m5C-methyltransferase, putative	5.10	0.0020	0.0055	0.0018	1.3	6931	596	5147	1638	-449
1563	PF07_0071	queuine tRNA-ribosyltransferase, putative	3.32	0.0020	0.0055	0.0018	1.1	6907	497	6253	870	-714
1250	PFI0590c	conserved Plasmodium protein, unknown function	3.76	0.0020	0.0055	0.0018	1.1	6886	566	6041	992	-713
2390	PFI0685w	pseudouridylate synthase, putative	2.37	0.0080	0.0173	0.0046	1.1	6883	561	6185	1361	-1224
1701	PF14_0561	conserved Plasmodium protein, unknown function	3.15	0.0020	0.0055	0.0018	1.1	6879	331	6053	1255	-759
1904	PFE0380c	nuclear pore associated protein (NPL4), putative	2.92	0.0060	0.0136	0.0036	1.1	6879	569	6136	1153	-979
1327	MAL13P1.52	conserved Plasmodium protein, unknown function	3.65	0.0020	0.0055	0.0018	1.1	6875	571	6018	1046	-760
965	PFA0585w	conserved Plasmodium protein, unknown function	4.22	0.0020	0.0055	0.0018	1.4	6867	836	5038	2001	-1008
1942	PFL0855c	conserved Plasmodium protein, unknown function	2.87	0.0060	0.0136	0.0036	1.1	6858	773	6171	976	-1061
995	PF08_0096	RNA helicase, putative	4.17	0.0020	0.0055	0.0018	1.3	6857	893	5248	1733	-1017
444	PF14_0640	conserved Plasmodium protein, unknown function	5.33	0.0020	0.0055	0.0018	1.3	6854	716	5116	1480	-459
1205	MAL7P1.34	conserved Plasmodium membrane protein, unknown fun	3.84	0.0020	0.0055	0.0018	1.2	6849	767	5617	1435	-970
925	PFD0270c	conserved Plasmodium protein, unknown function	4.32	0.0020	0.0055	0.0018	1.3	6844	550	5101	1922	-729
1086	PFD0945c	conserved Plasmodium membrane protein, unknown fun	4.04	0.0020	0.0055	0.0018	1.2	6835	512	5692	1318	-688
1010	PF11_0243	leucine-rich repeat protein	4.15	0.0020	0.0055	0.0018	1.3	6834	956	5248	1692	-1061
1487	MAL8P1.65	DNA helicase, putative	3.40	0.0020	0.0055	0.0018	1.1	6832	485	5958	1190	-802
1213	PF11_0255	ribonucleoprotein, putative	3.82	0.0020	0.0055	0.0018	1.1	6830	395	6081	902	-547
658	PF07_0066	conserved Plasmodium protein, unknown function	4.80	0.0020	0.0055	0.0018	1.4	6822	805	5035	1693	-711
1159	PFC0890w	SNARE protein	3.90	0.0020	0.0055	0.0018	1.1	6821	457	6030	916	-582
573	PF14_0456	U3 snoRNA-associated small subunit rRNA processing prc	5.01	0.0020	0.0055	0.0018	1.2	6820	521	5763	936	-401
2370	MAL13P1.88	conserved Plasmodium protein, unknown function	2.40	0.0060	0.0136	0.0036	1.1	6816	442	6060	1498	-1184
216	PF10_0096	conserved Plasmodium protein, unknown function	6.18	0.0020	0.0055	0.0018	1.4	6814	515	4771	1559	-32
1579	PF11_0053	chromatin remodeling protein	3.29	0.0020	0.0055	0.0018	1.2	6803	612	5862	1302	-973
1792	PFD0665c	26S proteasome AAA-ATPase subunit RPT3, putative	3.04	0.0020	0.0055	0.0018	1.2	6794	642	5849	1422	-1120
749	PFL0695c	conserved protein, unknown function	4.63	0.0020	0.0055	0.0018	1.1	6791	469	6022	711	-412
469	MAL13P1.274	serine/threonine protein phosphatase	5.26	0.0020	0.0055	0.0018	1.3	6790	546	5067	1539	-361
1052	PFL2260w	conserved Plasmodium protein, unknown function	4.08	0.0020	0.0055	0.0018	1.3	6789	689	5389	1577	-866
913	PFI0470w	FHA domain protein, putative	4.33	0.0020	0.0055	0.0018	1.3	6789	1018	5134	1664	-1028
1629	PFI0780w	conserved Plasmodium protein, unknown function	3.24	0.0020	0.0055	0.0018	1.1	6786	640	5959	1131	-944
1489	PF14_0661	small subunit rRNA processing KH domain protein, putati	3.40	0.0020	0.0055	0.0018	1.2	6776	758	5649	1494	-1125
1728	PF08_0028	conserved Plasmodium membrane protein, unknown fun	3.11	0.0020	0.0055	0.0018	1.1	6773	603	5915	1251	-997
1897	PF14_0513	RNA binding protein, putative	2.93	0.0060	0.0136	0.0036	1.1	6767	808	6106	873	-1021

1249	PF08_0034	histone acetyltransferase GCN5	3.76	0.0020	0.0055	0.0018	1.1	6765	485	5943	988	-652
1118	PF10_0361	conserved Plasmodium protein, unknown function	3.99	0.0020	0.0055	0.0018	1.2	6761	1156	5470	1254	-1119
1834	PF11_0187	clathrin assembly protein AP19, putative	2.99	0.0100	0.0208	0.0055	1.1	6761	581	6088	987	-895
308	PFI1215w	splicing factor 3A	5.76	0.0020	0.0055	0.0018	1.3	6760	507	5367	1106	-220
1625	PF13_0292	RAP protein, putative	3.24	0.0040	0.0098	0.0026	1.1	6756	640	5913	1154	-952
490	MAL13P1.13	conserved Plasmodium protein, unknown function	5.21	0.0020	0.0055	0.0018	1.5	6756	662	4609	1941	-456
1601	PF13_0347	conserved Plasmodium protein, unknown function	3.27	0.0040	0.0098	0.0026	1.1	6751	482	5901	1209	-841
917	PF14_0086	tRNA-dihydrouridine synthase, putative	4.32	0.0020	0.0055	0.0018	1.2	6743	669	5596	1173	-694
440	PFD1090c	clathrin assembly protein, putative	5.34	0.0020	0.0055	0.0018	1.5	6742	732	4427	2034	-452
320	PF13_0273	P-loop containing nucleoside triphospahte hydrolase, put	5.70	0.0020	0.0055	0.0018	1.3	6736	329	5105	1374	-72
914	MAL8P1.111	JmjC domain containing protein	4.33	0.0020	0.0055	0.0018	1.3	6718	824	5157	1624	-888
715	PF14_0593	conserved Plasmodium protein, unknown function	4.70	0.0020	0.0055	0.0018	1.3	6714	851	5141	1477	-755
761	PF11_0336	pre-mRNA splicing factor, putative	4.61	0.0020	0.0055	0.0018	1.3	6712	562	5179	1560	-590
391	PF14_0688	pre-mRNA-splicing factor ISY1 homolog, putative	5.48	0.0020	0.0055	0.0018	1.2	6708	561	5386	1084	-324
241	PFB0200c	aspartate aminotransferase	6.06	0.0020	0.0055	0.0018	1.3	6696	529	5284	1052	-168
1807	PFD0880w	golgi organization and biogenesis factor, putative	3.02	0.0020	0.0055	0.0018	1.2	6695	851	5597	1629	-1382
1443	PFI0610w	conserved Plasmodium protein, unknown function	3.47	0.0020	0.0055	0.0018	1.2	6688	496	5491	1639	-938
2631	PFC0130c	conserved Plasmodium protein, unknown function	2.11	0.0220	0.0408	0.0107	1.1	6666	725	5897	1677	-1632
1204	PFB0920w	DnaJ protein, putative	3.84	0.0040	0.0098	0.0026	1.3	6665	1436	5066	1648	-1484
781	PF13_0064	conserved Plasmodium protein, unknown function	4.57	0.0020	0.0055	0.0018	1.3	6661	595	5089	1611	-633
505	PFE1363c		5.16	0.0020	0.0055	0.0018	1.5	6648	714	4444	2003	-513
1334	PF10_0130	conserved Plasmodium protein, unknown function	3.64	0.0080	0.0173	0.0046	1.1	6643	441	5820	1044	-662
600	MAL7P1.78	conserved Plasmodium protein, unknown function	4.95	0.0020	0.0055	0.0018	1.3	6642	685	5190	1316	-549
1500	PF14_0671	conserved protein, unknown function	3.39	0.0060	0.0136	0.0036	1.1	6637	510	6126	602	-601
1345	PF11_0241	conserved Plasmodium protein, unknown function	3.63	0.0020	0.0055	0.0018	1.2	6632	676	5499	1420	-963
156	PFB0700c	conserved Plasmodium protein, unknown function	6.59	0.0020	0.0055	0.0018	1.2	6626	599	5456	709	-138
1102	PFL0980w	conserved Plasmodium protein, unknown function	4.02	0.0020	0.0055	0.0018	1.2	6624	558	5318	1521	-772
362	PF14_0125	deoxyhypusine synthase	5.56	0.0020	0.0055	0.0018	1.5	6623	632	4508	1787	-303
2292	PFI0090c	probable protein, unknown function	2.49	0.0180	0.0344	0.0090	1.2	6621	1031	5634	1740	-1784
1775	PF07_0099	conserved Plasmodium protein, unknown function	3.07	0.0060	0.0136	0.0036	1.2	6618	1016	5430	1696	-1524
575	PF11_0205	alternative splicing factor ASF-1, putative	5.00	0.0020	0.0055	0.0018	1.2	6618	505	5375	1141	-404
980	PF10_0112	conserved Plasmodium membrane protein, unknown fun	4.20	0.0020	0.0055	0.0018	1.2	6614	563	5509	1198	-656
1568	PFF1290c	conserved Plasmodium protein, unknown function	3.31	0.0020	0.0055	0.0018	1.2	6608	747	5714	1165	-1019
1636	PF13_0342	conserved Plasmodium protein, unknown function	3.22	0.0020	0.0055	0.0018	1.2	6606	655	5519	1557	-1125
1541	PF10_0097	mitochondrial ribosomal protein L22/L43, putative	3.34	0.0020	0.0055	0.0018	1.2	6604	1050	5480	1394	-1319
1464	PFL2315c	conserved Plasmodium membrane protein, unknown fun	3.44	0.0020	0.0055	0.0018	1.2	6602	780	5553	1345	-1076
439	PFC0450w	conserved Plasmodium protein, unknown function	5.34	0.0020	0.0055	0.0018	1.4	6594	551	4835	1546	-338
630	PF07_0109a	conserved Plasmodium protein, unknown function	4.87	0.0020	0.0055	0.0018	1.4	6594	761	4747	1745	-659
2276	MAL7P1.120	ubiquitin carboxyl-terminal hydrolase, putative	2.50	0.0080	0.0173	0.0046	1.1	6592	869	5810	1347	-1434
369	MAL7P1.158	signal recognition particle SRP9	5.54	0.0020	0.0055	0.0018	1.4	6592	731	4555	1693	-386
689	PFC1060c	conserved Plasmodium protein, unknown function	4.75	0.0020	0.0055	0.0018	1.2	6590	685	5619	821	-535
988	PF14_0146	ribonucleoprotein, putative	4.18	0.0020	0.0055	0.0018	1.2	6588	433	5710	962	-516
1386	PF11_0464a	conserved Plasmodium protein, unknown function	3.56	0.0020	0.0055	0.0018	1.2	6586	863	5308	1603	-1188

975	PFA0610c	Plasmodium exported protein (hyp11), unknown functior	4.21	0.0020	0.0055	0.0018	1.5	6577	2068	4293	2013	-1796
930	PF08_0085	ubiquitin conjugating enzyme, putative	4.30	0.0020	0.0055	0.0018	1.2	6576	842	5376	1169	-811
1517	PFE0375w	cell differentiation protein rcd1, putative	3.37	0.0020	0.0055	0.0018	1.1	6573	766	5748	1014	-954
1225	MAL7P1.206	DNA mismatch repair protein, putative	3.80	0.0020	0.0055	0.0018	1.2	6572	998	5344	1342	-1111
2080	PF07_0057	transcription elongation factor s-II, putative	2.73	0.0020	0.0055	0.0018	1.1	6564	559	5821	1250	-1066
2000	PF14_0128		2.81	0.0040	0.0098	0.0026	1.1	6561	678	5913	974	-1004
2192	PF13_0356	conserved Plasmodium protein, unknown function	2.59	0.0080	0.0173	0.0046	1.1	6555	1123	5725	1252	-1545
1215	PFL0990w	conserved Plasmodium protein, unknown function	3.82	0.0020	0.0055	0.0018	1.1	6547	474	5788	889	-604
802	PFE1435c	nucleolar preribosomal GTPase, putative	4.52	0.0020	0.0055	0.0018	1.3	6544	761	5069	1467	-752
188	PF08_0110	Rab GTPase 18	6.36	0.0020	0.0055	0.0018	1.4	6541	470	4832	1255	-16
1805	PF14_0415	dephospho-CoA kinase, putative	3.02	0.0040	0.0098	0.0026	1.2	6537	657	5629	1364	-1112
2333	PFL2395c	apicoplast dimethyladenosine synthase, putative	2.44	0.0160	0.0311	0.0082	1.1	6532	540	6040	877	-925
946	PFD1115c	conserved Plasmodium protein, unknown function	4.27	0.0020	0.0055	0.0018	1.2	6525	635	5265	1346	-721
1797	MAL13P1.132	micro-fibrillar-associated protein, putative	3.04	0.0040	0.0098	0.0026	1.1	6523	613	5682	1255	-1027
868	PF14_0087	conserved Plasmodium protein, unknown function	4.41	0.0020	0.0055	0.0018	1.2	6517	492	5539	1006	-519
688	PFL2270w	conserved protein, unknown function	4.75	0.0020	0.0055	0.0018	1.1	6514	514	5867	509	-376
350	PFF1440w	SET domain protein, putative	5.59	0.0020	0.0055	0.0018	1.4	6509	572	4629	1575	-267
724	PF08_0042	ATP-dependent RNA helicase prh1, putative	4.69	0.0020	0.0055	0.0018	1.2	6499	437	5427	1059	-423
163	PFC0140c	N-ethylmaleimide sensitive fusion protein, putative	6.53	0.0020	0.0055	0.0018	1.3	6496	443	4983	1072	-1
499	PFL1820w	rRNA processing WD-repeat protein, putative	5.18	0.0020	0.0055	0.0018	1.3	6487	653	5176	1112	-454
226	PF11_0393	ubiquitin related modifier homologue	6.11	0.0020	0.0055	0.0018	1.4	6485	700	4752	1258	-225
1037	PFC0765c	conserved Plasmodium protein, unknown function	4.11	0.0020	0.0055	0.0018	1.2	6484	737	5315	1248	-816
640	PFI0760w	conserved Plasmodium protein, unknown function	4.85	0.0020	0.0055	0.0018	1.4	6475	781	4730	1638	-674
1631	PFL2590w	Plasmodium exported protein (PHISTa), unknown functio	3.23	0.0020	0.0055	0.0018	1.4	6471	2319	4532	2200	-2580
2102	MAL13P1.39	conserved Plasmodium protein, unknown function	2.70	0.0120	0.0243	0.0064	1.1	6468	689	5801	1065	-1087
1240	PFL2015w	Tetratricopeptide repeat protein, putative	3.78	0.0020	0.0055	0.0018	1.2	6456	685	5290	1399	-918
105	PFI0250c	conserved Plasmodium membrane protein, unknown fun	7.03	0.0020	0.0055	0.0018	1.5	6456	830	4329	1307	-10
805	PF11_0415	conserved Plasmodium protein, unknown function	4.51	0.0020	0.0055	0.0018	1.4	6455	960	4677	1753	-936
2159	PF13_0151	conserved Plasmodium protein, unknown function	2.62	0.0100	0.0208	0.0055	1.1	6454	970	5646	1272	-1434
1112	PF10_0277	nucleolar preribosomal assembly protein, putative	3.99	0.0020	0.0055	0.0018	1.2	6440	883	5284	1209	-935
2219	PF07_0106	conserved Plasmodium protein, unknown function	2.56	0.0100	0.0208	0.0055	1.1	6436	742	5725	1212	-1242
526	PF13_0285	inositol-polyphosphate 5-phosphatase, putative	5.10	0.0020	0.0055	0.0018	1.3	6435	716	5145	1083	-510
1414	PFE1055c	conserved Plasmodium protein, unknown function	3.51	0.0040	0.0098	0.0026	1.3	6434	821	5034	1828	-1249
672	PF10_0116	conserved Plasmodium protein, unknown function	4.78	0.0020	0.0055	0.0018	1.2	6419	546	5381	960	-469
1169	MAL8P1.143		3.89	0.0020	0.0055	0.0018	1.2	6412	490	5546	1009	-633
1822	PFF0445w	conserved Plasmodium protein, unknown function	3.01	0.0060	0.0136	0.0036	1.2	6407	651	5269	1773	-1285
775	PFE1235c	ubiquitin fusion degradation protein UFD1, putative	4.58	0.0020	0.0055	0.0018	1.3	6402	606	5047	1356	-607
682	MAL8P1.57	C-13 antigen	4.76	0.0020	0.0055	0.0018	1.2	6401	483	5125	1249	-456
290	PFB0675w	conserved Plasmodium membrane protein, unknown fun	5.80	0.0020	0.0055	0.0018	1.3	6400	604	5022	1048	-274
2520	MAL13P1.34	RED-like protein, putative	2.24	0.0220	0.0408	0.0107	1.1	6398	420	5911	1006	-939
217	PFE1185w	transporter, putative	6.17	0.0020	0.0055	0.0018	1.6	6396	757	3900	1877	-138
1961	PF11_0394	conserved Plasmodium membrane protein, unknown fun	2.86	0.0120	0.0243	0.0064	1.2	6391	830	5432	1490	-1361
1143	PF10_0314	dcp1 homologue, putative	3.94	0.0020	0.0055	0.0018	1.3	6388	1161	4854	1641	-1268

1537	PF14_0605	cyclin homologue	3.34	0.0020	0.0055	0.0018	1.2	6385	721	5173	1669	-1178
1236	PF07_0084	conserved Plasmodium protein, unknown function	3.78	0.0020	0.0055	0.0018	1.3	6383	837	5084	1528	-1066
999	PFI0115c	serine/threonine protein kinase, FIKK family	4.17	0.0020	0.0055	0.0018	1.3	6381	1031	4820	1619	-1089
2123	PFE1420w	f-actin capping protein alpha subunit, putative	2.67	0.0120	0.0243	0.0064	1.1	6375	720	5743	991	-1079
207	PFB0560w	conserved Plasmodium protein, unknown function	6.23	0.0020	0.0055	0.0018	1.6	6373	785	3912	1820	-144
901	PF11_0452	conserved Plasmodium protein, unknown function	4.36	0.0020	0.0055	0.0018	1.3	6370	764	4947	1465	-807
837	PF14_0584	mitochondrial ribosomal protein S4/S9 precursor, putativ	4.45	0.0020	0.0055	0.0018	1.2	6364	737	5102	1245	-719
2304	PF08_0071	Fe-superoxide dismutase	2.47	0.0180	0.0344	0.0090	1.2	6359	1377	5287	1784	-2089
1639	PFF0620c	6-cysteine protein	3.22	0.0040	0.0098	0.0026	1.2	6351	622	5285	1533	-1090
2173	PFI0945w	thioredoxin, putative	2.61	0.0240	0.0438	0.0115	1.2	6343	895	5425	1548	-1525
2209	PFI1490c		2.57	0.0080	0.0173	0.0046	1.1	6341	573	5616	1297	-1144
466	PFE1140c	G10 protein, putative	5.27	0.0020	0.0055	0.0018	1.3	6324	623	4722	1395	-416
255	PFB0910w	Plasmodium exported protein, unknown function	6.00	0.0020	0.0055	0.0018	1.4	6320	770	4471	1364	-285
1295	PF10_0382	hypothetical protein	3.69	0.0040	0.0098	0.0026	1.4	6319	1164	4377	2389	-1611
1742	PFI1295c	monocarboxylate transporter, putative	3.10	0.0080	0.0173	0.0046	1.3	6318	547	5015	2005	-1249
723	PFC0582c		4.69	0.0020	0.0055	0.0018	1.3	6315	781	4689	1567	-722
2554	PFL1530w	asparagine-rich protein, putative	2.20	0.0259	0.0469	0.0123	1.2	6310	1425	5343	1789	-2248
353	PF14_0370	DEAD/DEAH box helicase, putative	5.58	0.0020	0.0055	0.0018	1.5	6296	738	4320	1619	-381
996	PF13_0362	U1 small nuclear ribonucleoprotein a, putative	4.17	0.0020	0.0055	0.0018	1.2	6292	449	5314	1085	-556
2220	MAL8P1.150	adenylyl cyclase beta, putative	2.56	0.0140	0.0277	0.0073	1.2	6264	1167	5341	1472	-1716
919	PF14_0699	GTPase activator, putative	4.32	0.0020	0.0055	0.0018	1.2	6261	734	5087	1180	-739
1578	MAL13P1.289	mitotic control protein dis3 homologue, putative	3.30	0.0020	0.0055	0.0018	1.2	6259	556	5261	1409	-966
424	PF11_0218	conserved Plasmodium protein, unknown function	5.39	0.0020	0.0055	0.0018	1.6	6259	1307	4008	1727	-782
364	PFI0365w	translation initiation factor SUI1, putative	5.56	0.0020	0.0055	0.0018	1.4	6254	585	4380	1576	-288
398	PF14_0274	diphthamide synthesis protein, putative	5.46	0.0020	0.0055	0.0018	1.4	6251	910	4423	1450	-532
402	PFI0100c	serine/threonine protein kinase, FIKK family	5.46	0.0020	0.0055	0.0018	1.5	6248	1006	4035	1797	-590
932	PF14_0529	gamma-adaptin, putative	4.30	0.0020	0.0055	0.0018	1.1	6242	598	5499	683	-537
459	PFF0125c	conserved Plasmodium protein, unknown function	5.29	0.0020	0.0055	0.0018	1.3	6241	489	4696	1371	-315
1352	MAL8P1.200	acetyltransferase, putative	3.62	0.0020	0.0055	0.0018	1.1	6237	471	5482	943	-658
368	PF14_0724	bromodomain protein, putative	5.54	0.0020	0.0055	0.0018	1.5	6224	817	4093	1754	-440
862	PFE0515w	conserved Plasmodium protein, unknown function	4.41	0.0020	0.0055	0.0018	1.3	6222	657	4806	1472	-712
1958	PF13_0172	conserved Plasmodium membrane protein, unknown fun	2.86	0.0100	0.0208	0.0055	1.2	6211	1068	5171	1541	-1568
1521	PF14_0496	leucine-rich repeat protein	3.36	0.0040	0.0098	0.0026	1.1	6206	611	5459	961	-824
303	PF14_0507	conserved Plasmodium protein, unknown function	5.77	0.0020	0.0055	0.0018	1.6	6198	512	3898	1904	-117
1181	PFE1535w	conserved Plasmodium protein, unknown function	3.87	0.0020	0.0055	0.0018	1.3	6194	700	4859	1583	-948
945	PF14_0156	small subunit rRNA dimethylase, putative	4.27	0.0020	0.0055	0.0018	1.4	6192	1233	4497	1649	-1187
1892	PFE0510c	conserved Plasmodium protein, unknown function	2.93	0.0060	0.0136	0.0036	1.2	6174	823	5242	1397	-1288
1777	PF11_0401		3.06	0.0020	0.0055	0.0018	1.1	6170	487	5676	676	-669
1457	PFC0265c	conserved Plasmodium protein, unknown function	3.45	0.0020	0.0055	0.0018	1.2	6165	617	5326	1073	-851
2449	PFF1377w	conserved Plasmodium membrane protein, unknown fun	2.31	0.0240	0.0438	0.0115	1.1	6159	691	5423	1450	-1405
220	PF11_0349	conserved Plasmodium protein, unknown function	6.16	0.0020	0.0055	0.0018	1.5	6157	785	4238	1376	-241
2061	PFD0835c	LETM1-like protein, putative	2.75	0.0020	0.0055	0.0018	1.2	6151	654	5322	1374	-1199
1776	MAL7P1.204	conserved Plasmodium protein, unknown function	3.06	0.0060	0.0136	0.0036	1.1	6150	758	5379	1054	-1042

2042	PFE0670w	mitochondrial matrix protein, putative	2.76	0.0100	0.0208	0.0055	1.2	6147	739	5214	1534	-1340
1482	PFD0930w	CGI-141 protein homolog, putative	3.41	0.0040	0.0098	0.0026	1.2	6141	914	5224	1073	-1070
648	PFC0790w	conserved Plasmodium protein, unknown function	4.83	0.0020	0.0055	0.0018	1.2	6141	673	4987	1026	-545
215	MAL7P1.108	phosphoinositide-binding protein, putative	6.19	0.0020	0.0055	0.0018	1.6	6135	675	3908	1668	-116
1823	PF14_0350	N-acetyltransferase, putative	3.01	0.0020	0.0055	0.0018	1.2	6123	651	5256	1304	-1087
2040	PF14_0515	conserved Plasmodium protein, unknown function	2.77	0.0060	0.0136	0.0036	1.2	6123	1167	4999	1729	-1772
261	PF14_0778		5.97	0.0020	0.0055	0.0018	1.3	6122	566	4674	1090	-207
823	PFE0880c	f-actin capping protein beta subunit, putative	4.48	0.0020	0.0055	0.0018	1.2	6115	677	5088	968	-619
538	PF11_0274	large ribosomal subunit processing factor, putative	5.07	0.0020	0.0055	0.0018	1.3	6114	699	4580	1361	-525
2014	MAL8P1.49	conserved Plasmodium membrane protein, unknown fun	2.79	0.0120	0.0243	0.0064	1.2	6106	818	5262	1310	-1284
280	PF11_0145	glyoxalase I	5.84	0.0020	0.0055	0.0018	1.4	6104	712	4276	1412	-296
555	PFD0600c	mitochondrial ribosomal protein S12 precursor, putative	5.04	0.0020	0.0055	0.0018	1.3	6100	1075	4534	1225	-734
2007	MAL8P1.92	ATPase, putative	2.80	0.0060	0.0136	0.0036	1.2	6087	709	5278	1281	-1182
181	PF11_0526	conserved Plasmodium protein, unknown function	6.40	0.0020	0.0055	0.0018	1.7	6082	708	3638	1774	-38
1276	PFF1480w	microtubule-associated protein ytm1 homologue, putativ	3.72	0.0020	0.0055	0.0018	1.3	6080	640	4641	1816	-1016
736	PFE0100w	conserved Plasmodium protein, unknown function	4.66	0.0020	0.0055	0.0018	1.3	6074	557	4603	1473	-558
1061	PF14_0178	ubiquitin fusion degradation protein UFD1, putative	4.07	0.0020	0.0055	0.0018	1.2	6060	508	5193	951	-593
1607	PF10_0324	conserved Plasmodium protein, unknown function	3.26	0.0020	0.0055	0.0018	1.1	6057	498	5381	925	-748
1000	PF11_0244	conserved Plasmodium protein, unknown function	4.16	0.0020	0.0055	0.0018	1.2	6034	342	5038	1137	-482
539	PF13_0212	conserved Plasmodium protein, unknown function	5.07	0.0020	0.0055	0.0018	1.3	6034	730	4646	1193	-535
376	PFI1180w	patatin-like phospholipase, putative	5.51	0.0020	0.0055	0.0018	1.4	6019	627	4316	1420	-344
1569	PFI0865w	XPA binding protein 1, putative	3.30	0.0020	0.0055	0.0018	1.1	6017	487	5339	919	-727
262	PFI0290c	beta subunit of coatomer complex, putative	5.96	0.0020	0.0055	0.0018	1.5	6004	650	4042	1518	-206
983	PF11_0479a	conserved Plasmodium membrane protein, unknown fun	4.19	0.0020	0.0055	0.0018	1.3	5996	641	4751	1351	-747
194	MAL13P1.324	aldo-keto reductase, putative	6.30	0.0020	0.0055	0.0018	1.6	5993	1093	3830	1410	-340
385	PF11_0398	conserved Plasmodium protein, unknown function	5.49	0.0020	0.0055	0.0018	1.5	5978	1215	3919	1527	-683
1196	PF07_0059	4-nitrophenylphosphatase	3.84	0.0020	0.0055	0.0018	1.3	5967	1169	4601	1438	-1241
641	PF07_0123	mRNA (N6-adenosine)-methyltransferase, putative	4.85	0.0020	0.0055	0.0018	1.3	5960	642	4650	1209	-541
382	PFD1060w	u5 small nuclear ribonucleoprotein-specific protein, puta	5.50	0.0020	0.0055	0.0018	1.4	5957	687	4203	1452	-385
219	MAL13P1.35	U1 small nuclear ribonucleoprotein a, putative	6.17	0.0020	0.0055	0.0018	1.4	5951	628	4399	1114	-190
849	PF13_0293	histone-lysine N-methyltransferase, putative	4.44	0.0020	0.0055	0.0018	1.2	5944	555	5124	776	-511
175	PFC0810c	conserved Plasmodium protein, unknown function	6.45	0.0020	0.0055	0.0018	1.5	5932	774	3991	1323	-156
1525	MAL8P1.126	serine protease, putative	3.35	0.0040	0.0098	0.0026	1.3	5928	1109	4706	1521	-1409
2490	PF11_0337	mitochondrial ribosomal protein L2 precursor	2.27	0.0240	0.0438	0.0115	1.1	5917	613	5479	793	-968
1335	PFF0270c	cyclin dependent kinase binding protein, putative	3.64	0.0020	0.0055	0.0018	1.3	5915	914	4465	1795	-1260
844	PF13_0313	zinc finger protein, putative	4.44	0.0020	0.0055	0.0018	1.2	5906	783	4912	879	-667
519	PFF0170w	cation/H antiporter	5.13	0.0020	0.0055	0.0018	1.5	5902	1107	4031	1527	-762
307	PF07_0024	inositol phosphatase, putative	5.76	0.0020	0.0055	0.0018	1.6	5891	1055	3668	1670	-503
1859	PFI1650w	DNA excision-repair helicase, putative	2.97	0.0060	0.0136	0.0036	1.2	5889	728	4897	1521	-1256
550	MAL13P1.293	conserved Plasmodium protein, unknown function	5.05	0.0020	0.0055	0.0018	1.4	5875	922	4225	1401	-673
1510	PF08_0040	clp1-related protein, putative	3.37	0.0020	0.0055	0.0018	1.2	5872	635	4778	1497	-1038
2062	PF11_0038	Plasmodium exported protein, unknown function	2.75	0.0100	0.0208	0.0055	1.2	5863	977	4740	1829	-1682
1436	PF14_0697	dihydroorotase, putative	3.48	0.0020	0.0055	0.0018	1.2	5860	659	4823	1351	-974

2239	PF13_0263	conserved Plasmodium protein, unknown function	2.53	0.0160	0.0311	0.0082	1.1	5846	1036	5099	1152	-1441
1686	PF10_0309	DEAD/DEAH box helicase, putative	3.17	0.0040	0.0098	0.0026	1.1	5835	800	5138	844	-947
1305	PFE0895c	zinc finger protein, putative	3.67	0.0020	0.0055	0.0018	1.2	5831	675	4811	1237	-891
2515	MAL13P1.244	TBC domain protein, putative	2.25	0.0259	0.0469	0.0123	1.2	5822	1092	5011	1514	-1794
725	PFF0105w	MYND finger protein, putative	4.69	0.0020	0.0055	0.0018	1.5	5811	844	3801	1977	-812
2280	PFD0940w	conserved Plasmodium protein, unknown function	2.50	0.0220	0.0408	0.0107	1.2	5810	849	4960	1507	-1505
2311	PFI0715w	zinc binding protein, putative	2.47	0.0040	0.0098	0.0026	1.2	5808	886	4996	1432	-1505
700	PF14_0635	large ribosomal subunit assembling protein, putative	4.73	0.0020	0.0055	0.0018	1.5	5806	710	3834	1955	-692
1898	PFC0465c	pre-mRNA splicing factor, putative	2.93	0.0020	0.0055	0.0018	1.2	5804	771	4858	1446	-1271
1150	PF13_0336	DNase I-like protein, putative	3.92	0.0020	0.0055	0.0018	1.3	5784	842	4458	1496	-1013
315	MAL7P1.35	conserved Plasmodium protein, unknown function	5.72	0.0020	0.0055	0.0018	1.4	5783	739	4213	1194	-362
363	PF13_0323	arginine methyltransferase 5, putative	5.56	0.0020	0.0055	0.0018	1.3	5775	564	4521	999	-309
1692	MAL13P1.261	conserved Plasmodium protein, unknown function	3.16	0.0040	0.0098	0.0026	1.3	5768	881	4568	1705	-1386
712	PF14_0630	protein serine/threonine phosphatase	4.70	0.0020	0.0055	0.0018	1.5	5747	1187	3764	1809	-1013
129	PF10_0099	DNA2/NAM7 helicase. putative	6.80	0.0020	0.0055	0.0018	1.5	5746	763	3790	1255	-62
778	PF14_0713	conserved Plasmodium protein, unknown function	4.57	0.0020	0.0055	0.0018	1.3	5741	750	4393	1300	-703
176	PFE1040c	conserved Plasmodium protein, unknown function	6.44	0.0020	0.0055	0.0018	1.3	5740	771	4278	905	-214
618	PFL0125c	conserved Plasmodium protein, unknown function	4.90	0.0020	0.0055	0.0018	1.3	5729	706	4450	1131	-559
651	PF11_0386	apicoplast ribosomal protein S14p/S29e precursor, putat	4.82	0.0020	0.0055	0.0018	1.4	5728	860	4182	1395	-710
1419	PFB0960c	Pfmc-2TM family pseudogene	3.50	0.0020	0.0055	0.0018	1.6	5726	1818	3579	2587	-2258
2476	MAL8P1.10	conserved protein, unknown function	2.28	0.0160	0.0311	0.0082	1.1	5723	699	5106	1186	-1268
2594	PFE1470w	cell cycle regulator protein, putative	2.16	0.0180	0.0344	0.0090	1.1	5720	807	5168	1055	-1310
197	PF14_0196	tetratricopeptide repeat family protein, putative	6.29	0.0020	0.0055	0.0018	1.4	5709	778	4144	1028	-241
1014	PF07_0041	conserved Plasmodium protein, unknown function	4.15	0.0020	0.0055	0.0018	1.3	5701	1073	4488	1116	-975
727	PF10_0341	U2 snRNA/tRNA pseudouridine synthase, putative	4.68	0.0020	0.0055	0.0018	1.2	5697	825	4677	812	-617
1764	PFL1400c	conserved Plasmodium protein, unknown function	3.08	0.0060	0.0136	0.0036	1.2	5693	993	4661	1416	-1377
2231	PF07_0037	Cg2 protein	2.55	0.0180	0.0344	0.0090	1.2	5686	1188	4728	1548	-1779
2074	PFL0970w	pre-mRNA splicing factor, putative	2.73	0.0120	0.0243	0.0064	1.2	5678	740	4896	1259	-1217
1694	PF14_0157	conserved Plasmodium protein, unknown function	3.16	0.0040	0.0098	0.0026	1.2	5663	789	4697	1342	-1166
1231	PFF1055c	conserved Plasmodium protein, unknown function	3.79	0.0040	0.0098	0.0026	1.2	5663	731	4849	853	-770
1522	PF14_0361	secretory complex protein 62	3.36	0.0020	0.0055	0.0018	1.3	5659	1134	4284	1765	-1524
649	PF10_0370	enhancer of rudimentary homolog, putative	4.82	0.0020	0.0055	0.0018	1.6	5650	1036	3516	1985	-888
1519	PFF0890c	conserved Plasmodium membrane protein, unknown fun	3.37	0.0040	0.0098	0.0026	1.2	5643	882	4524	1449	-1213
1516	PF14_0594	conserved Plasmodium protein, unknown function	3.37	0.0020	0.0055	0.0018	1.3	5631	1224	4499	1287	-1378
783	PFI0480w	helicase with Zn-finger motif, putative	4.56	0.0020	0.0055	0.0018	1.4	5623	1072	3923	1587	-959
1194	PFC0831w	triosephophate isomerase, putative	3.85	0.0020	0.0055	0.0018	1.4	5618	965	4078	1783	-1207
590	PFI0515w	SNARE protein, putative	4.97	0.0020	0.0055	0.0018	1.3	5607	694	4358	1083	-528
831	PFE0745w	conserved Plasmodium protein, unknown function	4.46	0.0020	0.0055	0.0018	1.4	5606	820	4054	1558	-826
240	PFF1450w	sec14-like cytosolic factor or phosphatidylinositol/phospl	6.06	0.0020	0.0055	0.0018	1.6	5603	815	3546	1514	-272
2519	PF14_0393	structure specific recognition protein, putative	2.24	0.0259	0.0469	0.0123	1.2	5578	1256	4486	2143	-2307
771	PF14_0374	CCAAT-binding transcription factor, putative	4.59	0.0020	0.0055	0.0018	2.0	5572	2031	2776	2451	-1686
1481	PFC0166w	plasmoredoxin	3.41	0.0020	0.0055	0.0018	1.2	5569	958	4537	1247	-1174
828	PF14_0369	copper transporter putative	4.47	0.0020	0.0055	0.0018	1.3	5568	866	4346	1125	-768

1059	PF11_0047	actin-like protein, putative	4.07	0.0020	0.0055	0.0018	1.2	5562	516	4644	1016	-614
1129	MAL7P1.134	conserved Plasmodium protein, unknown function	3.96	0.0020	0.0055	0.0018	1.2	5558	605	4637	1018	-702
1930	PFI0800c	conserved Plasmodium protein, unknown function	2.88	0.0100	0.0208	0.0055	1.2	5558	1054	4638	1286	-1421
2071	PFD0970c	zinc finger protein, putative	2.73	0.0100	0.0208	0.0055	1.2	5557	1096	4678	1279	-1495
2125	MAL13P1.107	conserved Plasmodium protein, unknown function	2.66	0.0259	0.0469	0.0123	1.2	5554	874	4752	1280	-1351
1860	PFL1115w	conserved Plasmodium protein, unknown function	2.96	0.0060	0.0136	0.0036	1.1	5549	590	4893	964	-898
1665	PFB0895c	replication factor C subunit 1, putative	3.19	0.0040	0.0098	0.0026	1.3	5545	1038	4433	1467	-1394
1320	PFC1015c	conserved Plasmodium protein, unknown function	3.66	0.0020	0.0055	0.0018	1.3	5539	911	4139	1713	-1224
2135	PFF0320c	polypyrimidine tract binding protein, putative	2.65	0.0100	0.0208	0.0055	1.2	5523	746	4787	1205	-1215
1389	PFF1385c	conserved Plasmodium protein, unknown function	3.55	0.0040	0.0098	0.0026	1.3	5512	722	4324	1522	-1055
1008	MAL13P1.28	conserved Plasmodium protein, unknown function	4.16	0.0020	0.0055	0.0018	1.4	5509	1005	4054	1493	-1043
860	PF14_0501	conserved Plasmodium protein, unknown function	4.42	0.0020	0.0055	0.0018	1.2	5509	597	4576	905	-569
135	PF14_0101	conserved Plasmodium protein, unknown function	6.73	0.0020	0.0055	0.0018	1.5	5506	710	3661	1205	-69
1935	PF13_0264	ubiquitin-activating enzyme e1, putative	2.88	0.0060	0.0136	0.0036	1.2	5506	811	4751	1088	-1144
297	PFL0170w	transporter, putative	5.79	0.0020	0.0055	0.0018	1.5	5505	1189	3568	1299	-551
1091	PFF1085a	conserved Plasmodium protein, unknown function	4.03	0.0020	0.0055	0.0018	1.3	5498	932	4076	1540	-1050
1708	PFF0365c	G-protein associated signal transduction protein, putative	3.15	0.0060	0.0136	0.0036	1.2	5496	721	4722	1042	-989
309	PFC0980c	RNA triphosphatase	5.75	0.0020	0.0055	0.0018	1.6	5488	742	3540	1537	-331
1890	PF14_0245	conserved Plasmodium protein, unknown function	2.93	0.0040	0.0098	0.0026	1.3	5486	1025	4158	2048	-1744
2153	PFD0240c	6-cysteine protein	2.63	0.0100	0.0208	0.0055	1.2	5480	1185	4701	1055	-1461
863	PF13_0037	DEAD box helicase, putative	4.41	0.0020	0.0055	0.0018	1.4	5474	1085	3919	1467	-997
269	PF14_0171	conserved Plasmodium protein, unknown function	5.88	0.0020	0.0055	0.0018	1.4	5474	784	3914	1120	-344
1761	PFL0895c	conserved Plasmodium protein, unknown function	3.08	0.0080	0.0173	0.0046	1.3	5474	1477	4333	1324	-1660
1497	PFL2540w	Plasmodium exported protein (PHISTb), unknown functio	3.39	0.0020	0.0055	0.0018	1.3	5466	1018	4248	1537	-1337
544	PF10_0219	kelch protein, putative	5.06	0.0020	0.0055	0.0018	1.6	5445	1229	3492	1583	-859
1696	PF10_0174	26s proteasome regulatory subunit p55, putative	3.15	0.0020	0.0055	0.0018	1.2	5442	1046	4457	1253	-1315
1206	PF14_0600	conserved Plasmodium protein, unknown function	3.84	0.0020	0.0055	0.0018	1.4	5440	1085	3997	1603	-1244
204	PF11_0297	NOT family protein, putative	6.24	0.0020	0.0055	0.0018	1.7	5425	1046	3169	1537	-327
710	PF14_0398	transcription factor TFIIH complex subunit Tfb5, putative	4.71	0.0020	0.0055	0.0018	1.5	5424	1443	3596	1466	-1080
254	PF14_0422	conserved Plasmodium protein, unknown function	6.01	0.0020	0.0055	0.0018	1.4	5424	601	3898	1138	-213
417	PF14_0342	conserved Plasmodium membrane protein, unknown fun	5.41	0.0020	0.0055	0.0018	1.3	5419	683	4149	998	-410
1171	PF11_0311	N-acetyl glucosamine phosphate mutase, putative	3.89	0.0020	0.0055	0.0018	1.2	5402	836	4374	1086	-895
1729	PF14_0108		3.11	0.0060	0.0136	0.0036	1.3	5396	1019	4243	1601	-1466
1002	PFB0210c	hexose transporter	4.16	0.0020	0.0055	0.0018	1.3	5392	503	4273	1247	-631
250	PFB0540w	conserved Plasmodium protein, unknown function	6.02	0.0020	0.0055	0.0018	1.7	5390	1010	3144	1619	-384
580	PF14_0052	conserved Plasmodium protein, unknown function	4.99	0.0020	0.0055	0.0018	1.3	5386	731	4045	1165	-556
1353	PF14_0704	conserved Plasmodium protein, unknown function	3.62	0.0040	0.0098	0.0026	1.2	5383	715	4503	1030	-865
1998	PFB0380c	conserved Plasmodium protein, unknown function	2.81	0.0060	0.0136	0.0036	1.3	5371	1100	4234	1750	-1713
2560	PFA0185w	conserved Plasmodium protein, unknown function	2.20	0.0220	0.0408	0.0107	1.1	5360	696	4725	1289	-1351
1416	PFI0170w	conserved Plasmodium protein, unknown function	3.51	0.0020	0.0055	0.0018	1.4	5342	1246	3892	1735	-1531
665	PF14_0512	conserved Plasmodium protein, unknown function	4.78	0.0020	0.0055	0.0018	1.3	5341	897	4013	1133	-701
326	PFL1470c	WD-repeat protein, putative	5.69	0.0020	0.0055	0.0018	1.4	5335	541	3794	1247	-247
1649	PF14_0358	41-2 protein antigen precursor,transport protein particle	3.21	0.0060	0.0136	0.0036	1.2	5333	607	4599	997	-870

2344	PF14_0330	conserved Plasmodium protein, unknown function	2.42	0.0200	0.0376	0.0099	1.2	5312	1088	4498	1369	-1642
1882	PFL2545c	Plasmodium exported protein, unknown function	2.94	0.0100	0.0208	0.0055	1.6	5311	2359	3271	2765	-3083
408	MAL8P1.112	conserved Plasmodium protein, unknown function	5.43	0.0020	0.0055	0.0018	1.4	5307	1102	3706	1109	-610
1124	PFL0560c	minichromosome maintenance protein, putative	3.97	0.0060	0.0136	0.0036	1.2	5307	664	4298	1113	-768
259	PF11_0123	conserved Plasmodium protein, unknown function	5.98	0.0020	0.0055	0.0018	1.7	5302	857	3109	1645	-309
1845	PF13_0089	inositol polyphosphate kinase, putative	2.98	0.0060	0.0136	0.0036	1.3	5300	1113	4178	1591	-1582
1814	PF10_0185	conserved Plasmodium protein, unknown function	3.02	0.0060	0.0136	0.0036	1.2	5300	872	4255	1529	-1357
605	PF14_0071	conserved Plasmodium protein, unknown function	4.94	0.0020	0.0055	0.0018	1.5	5298	805	3485	1664	-656
813	PFB0440c	conserved Plasmodium protein, unknown function	4.49	0.0020	0.0055	0.0018	1.4	5298	1165	3689	1455	-1012
1585	PF14_0749	acyl-CoA binding protein	3.29	0.0060	0.0136	0.0036	1.5	5297	1837	3464	2253	-2256
1222	PFF1060w	conserved Plasmodium protein, unknown function	3.81	0.0020	0.0055	0.0018	1.3	5287	941	3978	1484	-1117
2073	PFI0625c	tRNA 1-methyladenosine methyltransferase subunit, puta	2.73	0.0100	0.0208	0.0055	1.2	5270	1186	4256	1518	-1691
1867	PF13_0042	fork head domain protein, putative	2.95	0.0040	0.0098	0.0026	1.3	5268	948	4144	1685	-1510
1465	PF08_0125	tubulin gamma chain	3.43	0.0040	0.0098	0.0026	1.2	5265	933	4403	950	-1020
909	PFL0915c	conserved Plasmodium protein, unknown function	4.34	0.0020	0.0055	0.0018	1.4	5255	944	3806	1432	-927
177	PF10_0053	methionine-tRNA ligase, putative	6.42	0.0020	0.0055	0.0018	1.9	5254	1061	2727	1710	-243
1095	PF10_0181	conserved protein, unknown function	4.03	0.0020	0.0055	0.0018	1.3	5252	1155	4029	1127	-1060
171	MAL7P1.157a	RNA binding protein, putative	6.48	0.0020	0.0055	0.0018	1.4	5251	701	3795	930	-175
2028	PFB0020c	erythrocyte membrane protein 1 (PfEMP1), exon2	2.78	0.0080	0.0173	0.0046	1.3	5249	1325	4133	1620	-1829
1445	PF13_0154	conserved Plasmodium protein, unknown function	3.46	0.0060	0.0136	0.0036	1.3	5245	1004	3991	1559	-1309
1227	PFB0240w	conserved Plasmodium protein, unknown function	3.80	0.0020	0.0055	0.0018	1.4	5239	1518	3689	1537	-1505
622	MAL13P1.144	translation initiation factor EIF-2B gamma subunit, putati	4.90	0.0020	0.0055	0.0018	1.2	5233	713	4205	835	-521
161	PF14_0596	conserved Plasmodium membrane protein, unknown fun	6.55	0.0020	0.0055	0.0018	1.9	5233	994	2695	1704	-161
1937	MAL13P1.344	RNAse L inhibitor protein, putative	2.88	0.0060	0.0136	0.0036	1.2	5232	1060	4277	1359	-1465
1135	PFA0500w	human hepatopoietin-like protein, putative	3.95	0.0020	0.0055	0.0018	1.3	5232	1050	4003	1245	-1066
743	PF14_0449	conserved Plasmodium protein, unknown function	4.65	0.0020	0.0055	0.0018	1.6	5218	1133	3202	1900	-1017
663	PF14_0474	conserved Plasmodium protein, unknown function	4.79	0.0020	0.0055	0.0018	1.4	5193	601	3607	1542	-558
2558	PF08_0037	RNA polymerase II mediator complex protein MED7, puta	2.20	0.0240	0.0438	0.0115	1.2	5179	908	4345	1695	-1770
2482	PFD0367w	mitochondrial carrier protein, putative	2.27	0.0200	0.0376	0.0099	1.2	5177	771	4472	1375	-1441
1735	PFL2505c	conserved Plasmodium protein, unknown function	3.11	0.0080	0.0173	0.0046	1.4	5174	1256	3583	2276	-1940
1661	PFB0257c	conserved Plasmodium protein with pleckstrin homology	3.20	0.0040	0.0098	0.0026	1.3	5173	806	4085	1522	-1240
890	PF08_0027	conserved Plasmodium protein, unknown function	4.37	0.0020	0.0055	0.0018	1.5	5172	844	3541	1686	-899
967	PFL1130c	conserved Plasmodium protein, unknown function	4.22	0.0020	0.0055	0.0018	1.2	5170	593	4245	951	-618
541	PF10_0298	26S proteasome regulatory subunit, putative	5.07	0.0020	0.0055	0.0018	1.6	5170	937	3322	1605	-693
334	PF14_0389	conserved Plasmodium protein, unknown function	5.66	0.0020	0.0055	0.0018	1.5	5163	935	3540	1165	-476
481	PF11_0230	conserved Plasmodium protein, unknown function	5.22	0.0020	0.0055	0.0018	1.7	5162	795	2954	1961	-548
1131	PFD0895c	Bet3 transport protein, putative	3.96	0.0020	0.0055	0.0018	1.4	5161	1241	3618	1602	-1300
288	MAL8P1.46	dynein light chain 1	5.82	0.0020	0.0055	0.0018	1.4	5158	1133	3644	848	-468
1895	PFA0150c	conserved Plasmodium protein, unknown function	2.93	0.0140	0.0277	0.0073	1.2	5146	1037	4291	1136	-1317
2312	PF11_0266	small nuclear ribonucleoprotein D1, putative	2.46	0.0160	0.0311	0.0082	1.2	5142	892	4189	1740	-1679
2149	PF11_0066	centrin-4	2.63	0.0060	0.0136	0.0036	1.2	5131	966	4240	1445	-1520
561	PFI0350c	conserved Plasmodium protein, unknown function	5.04	0.0020	0.0055	0.0018	1.7	5128	1100	3090	1754	-816
1363	PF14_0475	conserved Plasmodium protein, unknown function	3.60	0.0020	0.0055	0.0018	1.3	5127	1004	4040	1217	-1134

1844	PF14_0791	dfg10 like protein, putative	2.98	0.0040	0.0098	0.0026	1.2	5127	788	4242	1295	-1199
772	PFF1445c	cullin-like protein, putative	4.59	0.0020	0.0055	0.0018	1.6	5115	1047	3298	1730	-961
529	PFB0195c	conserved Plasmodium protein, unknown function	5.09	0.0020	0.0055	0.0018	1.8	5113	1163	2910	1881	-840
366	PFL2435w	conserved Plasmodium protein, unknown function	5.55	0.0020	0.0055	0.0018	1.3	5103	889	3967	667	-421
294	PFL0140w	conserved Plasmodium protein, unknown function	5.79	0.0020	0.0055	0.0018	1.7	5086	1187	2943	1513	-557
93	PF14_0291	conserved Plasmodium protein, unknown function	7.10	0.0020	0.0055	0.0018	2.0	5085	1133	2497	1512	-57
1029	PF11_0092	mechanosensitive ion channel protein	4.12	0.0020	0.0055	0.0018	1.4	5078	937	3656	1496	-1011
2063	PFI0990c	CS-domain containing protein, conserved in Apicomplexa	2.74	0.0200	0.0376	0.0099	1.1	5075	732	4444	945	-1045
1268	PF13_0028	membrane integral peptidase, M50 family, putative	3.73	0.0040	0.0098	0.0026	1.4	5066	1206	3663	1537	-1340
1324	PFB0954c		3.65	0.0040	0.0098	0.0026	1.3	5065	877	4036	1167	-1015
611	PFD0155c	conserved Plasmodium protein, unknown function	4.93	0.0020	0.0055	0.0018	1.5	5061	932	3349	1510	-730
473	PF11_0364	conserved Plasmodium protein, unknown function	5.24	0.0020	0.0055	0.0018	1.9	5055	1351	2651	1938	-884
760	MAL8P1.93	conserved Plasmodium protein, unknown function	4.61	0.0040	0.0098	0.0026	1.3	5041	961	3915	883	-718
63	PFE1500c	conserved Plasmodium protein, unknown function	7.49	0.0020	0.0055	0.0018	3.3	5031	1770	1527	1742	-8
1997	PFF0683c	conserved Plasmodium protein, unknown function	2.82	0.0080	0.0173	0.0046	1.3	5027	1477	3974	1348	-1772
450	PF14_0664	biotin carboxylase subunit of acetyl CoA carboxylase, put	5.32	0.0020	0.0055	0.0018	1.5	5022	1029	3358	1266	-631
1437	PFI1050c	Fe-S-cluster redox enzyme, putative	3.48	0.0040	0.0098	0.0026	1.3	5018	991	3838	1439	-1250
661	PF14_0670	conserved Plasmodium protein, unknown function	4.80	0.0020	0.0055	0.0018	1.5	5017	1226	3396	1296	-902
1219	PFL0525w	conserved Plasmodium protein, unknown function	3.81	0.0020	0.0055	0.0018	1.3	5013	915	3984	1075	-961
310	PFE0610c	CDK-activating kinase assembly factor	5.75	0.0020	0.0055	0.0018	1.5	5000	717	3405	1218	-340
2305	PFI0305c	conserved Plasmodium protein, unknown function	2.47	0.0180	0.0344	0.0090	1.1	4996	772	4378	1042	-1195
1731	PF14_0223	cyclophilin, putative	3.11	0.0080	0.0173	0.0046	1.4	4986	1135	3652	1872	-1673
1269	MAL7P1.75	mitochondrial ATP synthase F1, epsilon subunit, putative	3.73	0.0020	0.0055	0.0018	1.3	4985	1001	3837	1250	-1103
902	PF14_0193	conserved protein, unknown function	4.35	0.0020	0.0055	0.0018	1.4	4982	997	3653	1240	-908
1835	PF13_0329	conserved Plasmodium protein, unknown function	2.99	0.0080	0.0173	0.0046	1.2	4980	965	4089	1212	-1286
517	PF07_0058	conserved Plasmodium protein, unknown function	5.13	0.0020	0.0055	0.0018	1.4	4975	785	3564	1173	-548
1326	PF10_0353		3.65	0.0020	0.0055	0.0018	1.4	4969	1578	3460	1531	-1600
675	PF11_0295	farnesyl pyrophosphate synthase, putative	4.77	0.0020	0.0055	0.0018	1.4	4965	1010	3584	1135	-764
1829	PFF0780w	conserved Plasmodium protein, unknown function	3.00	0.0020	0.0055	0.0018	1.3	4956	873	3959	1456	-1331
1066	PF10_0287	conserved Plasmodium protein, unknown function	4.06	0.0020	0.0055	0.0018	1.3	4948	738	3845	1176	-812
84	PFA0165c	conserved Plasmodium protein, unknown function	7.19	0.0020	0.0055	0.0018	1.5	4928	738	3346	881	-37
2048	PF14_0283	conserved Plasmodium protein, unknown function	2.75	0.0160	0.0311	0.0082	1.3	4917	1263	3782	1718	-1846
2378	PFI0530c	DNA primase large subunit, putative	2.39	0.0240	0.0438	0.0115	1.2	4910	1242	4115	1252	-1699
1527	MAL13P1.279	protein kinase 5	3.35	0.0040	0.0098	0.0026	1.3	4910	972	3809	1385	-1257
543	PFC0261c	conserved Plasmodium protein, unknown function	5.06	0.0020	0.0055	0.0018	1.7	4877	1173	2912	1626	-834
549	PF11_0041	Plasmodium exported protein (hyp11), unknown functior	5.05	0.0020	0.0055	0.0018	2.1	4873	1908	2349	1848	-1233
492	MAL8P1.380	conserved Plasmodium protein, unknown function	5.20	0.0020	0.0055	0.0018	2.0	4857	1339	2484	1932	-898
332	PF08_0043	conserved Plasmodium protein, unknown function	5.67	0.0020	0.0055	0.0018	1.8	4857	1140	2642	1656	-582
253	PFI0925w	gamma-glutamylcysteine synthetase	6.01	0.0020	0.0055	0.0018	1.7	4839	1026	2781	1439	-406
202	MAL7P1.151	DNA (cytosine-5)-methyltransferase, putative;with=InterI	6.24	0.0020	0.0055	0.0018	1.6	4838	696	3092	1239	-188
477	PF10_0333	conserved Plasmodium protein, unknown function	5.23	0.0020	0.0055	0.0018	1.8	4837	1237	2646	1771	-817
1423	MAL7P1.177	Plasmodium exported protein (hyp9), unknown function	3.49	0.0020	0.0055	0.0018	1.3	4836	728	3772	1360	-1025
624	PF13_0122	RRM containing cyclophilin, putative	4.89	0.0020	0.0055	0.0018	1.4	4834	696	3515	1185	-562

233	PFD0160w	conserved Plasmodium protein, unknown function	6.09	0.0020	0.0055	0.0018	1.9	4833	1521	2538	1331	-557
1551	PF10_0147	FAD synthetase, putative	3.33	0.0100	0.0208	0.0055	1.3	4822	1038	3766	1286	-1268
449	PF14_0115	conserved Plasmodium protein, unknown function	5.32	0.0020	0.0055	0.0018	1.6	4814	1209	3091	1219	-705
878	MAL8P1.121	coatomer epsilon subunit, putative	4.39	0.0020	0.0055	0.0018	1.5	4812	1160	3241	1458	-1047
706	PF10_0108	conserved Plasmodium protein, unknown function	4.72	0.0020	0.0055	0.0018	1.3	4812	719	3653	1041	-601
146	MAL7P1.10	centrin, putative	6.66	0.0020	0.0055	0.0018	2.2	4805	982	2200	1728	-105
1232	PF13_0282	proteasome subunit, putative	3.79	0.0020	0.0055	0.0018	1.6	4798	1390	2909	2141	-1643
313	PFE0835w	ubiquitin carboxyl-terminal hydrolase 2, putative	5.72	0.0020	0.0055	0.0018	1.4	4786	936	3378	914	-442
211	PF14_0292	large ribosomal subunit associated GTPase, putative	6.22	0.0020	0.0055	0.0018	1.7	4780	1114	2740	1307	-381
704	PFL1240c	conserved Plasmodium protein, unknown function	4.72	0.0020	0.0055	0.0018	1.6	4774	861	2994	1700	-781
336	PF08_0123	U2 snRNA/tRNA pseudouridine synthase, putative	5.66	0.0020	0.0055	0.0018	1.4	4774	876	3388	948	-438
159	PF11_0142	ubiquitin domain containing protein	6.56	0.0020	0.0055	0.0018	1.7	4760	1098	2859	1077	-275
1680	PFI0955w	sugar transporter, putative	3.17	0.0040	0.0098	0.0026	1.5	4757	1538	3264	1908	-1953
2394	PFF1115w	ferredoxin NADP reductase	2.37	0.0259	0.0469	0.0123	1.2	4737	1085	3877	1526	-1750
1144	PF14_0733	serine/threonine protein kinase, FIKK family	3.94	0.0020	0.0055	0.0018	2.0	4730	2349	2311	2275	-2204
507	PF11_0169	SNO glutamine amidotransferase family protein	5.16	0.0020	0.0055	0.0018	1.4	4726	700	3357	1159	-491
536	PF11_0469	conserved Plasmodium protein, unknown function	5.07	0.0020	0.0055	0.0018	1.5	4722	978	3046	1393	-696
626	PFI0700c	met-10 like protein, putative	4.87	0.0020	0.0055	0.0018	1.5	4713	1021	3048	1437	-793
2054	MAL13P1.241	GTPase, putative	2.75	0.0140	0.0277	0.0073	1.2	4709	796	3975	1123	-1185
145	PF13_0333	conserved Plasmodium protein, unknown function	6.68	0.0020	0.0055	0.0018	1.4	4704	611	3296	896	-100
745	PFF0925w	conserved Plasmodium protein, unknown function	4.64	0.0020	0.0055	0.0018	1.3	4687	801	3606	921	-642
1987	PF14_0098	tetratricopeptide repeat family protein, putative	2.83	0.0120	0.0243	0.0064	1.2	4661	906	3832	1220	-1297
1618	PFF1130c	superoxide dismutase	3.25	0.0020	0.0055	0.0018	1.2	4656	598	3878	1060	-880
1717	PF14_0616	ATP-dependent protease la, putative	3.12	0.0040	0.0098	0.0026	1.2	4655	960	3741	1184	-1230
1709	PFL2520w	reticulocyte binding protein homologue 3, pseudogene	3.13	0.0020	0.0055	0.0018	1.3	4653	860	3705	1294	-1206
563	PFD0925w	conserved Plasmodium protein, unknown function	5.03	0.0020	0.0055	0.0018	1.6	4630	1163	2910	1363	-805
397	PF08_0053	BRIX domain, putative	5.47	0.0020	0.0055	0.0018	1.7	4622	1194	2660	1443	-676
1903	PFB0325c	serine repeat antigen 8	2.92	0.0140	0.0277	0.0073	1.3	4615	1066	3498	1648	-1597
1507	PFE0665c	GTP binding protein, putative	3.38	0.0020	0.0055	0.0018	1.2	4612	789	3804	967	-948
935	PF14_0610	zinc finger protein, putative	4.29	0.0020	0.0055	0.0018	1.4	4604	1262	3262	1106	-1025
595	PF10_0122	phosphoglucomutase, putative	4.96	0.0020	0.0055	0.0018	1.7	4598	1079	2692	1648	-822
2404	PF11_0225	ABC transporter GCN20	2.36	0.0140	0.0277	0.0073	1.3	4596	1209	3596	1810	-2020
1919	MAL13P1.150	conserved Plasmodium protein, unknown function	2.90	0.0040	0.0098	0.0026	1.3	4591	1166	3504	1552	-1631
2148	PF10_0066	thioredoxin, putative	2.64	0.0120	0.0243	0.0064	1.3	4590	1131	3652	1460	-1652
1080	PFA0685c	Plasmodium exported protein (hyp4), unknown function	4.05	0.0020	0.0055	0.0018	1.4	4585	1268	3181	1325	-1189
214	PFD0345c	conserved Plasmodium protein, unknown function	6.21	0.0020	0.0055	0.0018	2.0	4584	1419	2336	1315	-486
1538	MAL13P1.126	DHHC-type zinc finger protein, putative	3.34	0.0040	0.0098	0.0026	1.4	4579	1087	3278	1673	-1459
2395	PF14_0056	RNA binding protein, putative	2.37	0.0200	0.0376	0.0099	1.3	4550	1477	3599	1526	-2051
1559	PF10_0109	conserved Plasmodium protein, unknown function	3.32	0.0060	0.0136	0.0036	1.4	4528	939	3348	1550	-1309
104	PFI1385c	conserved Plasmodium protein, unknown function	7.04	0.0020	0.0055	0.0018	1.7	4510	843	2721	1025	-79
1496	PFB0835c	conserved Plasmodium protein, unknown function	3.39	0.0040	0.0098	0.0026	1.4	4503	1097	3226	1598	-1417
826	PFF0505c	conserved Plasmodium protein, unknown function	4.47	0.0020	0.0055	0.0018	1.5	4497	1000	2913	1519	-935
2257	PFL1875w	conserved Plasmodium protein, unknown function	2.52	0.0180	0.0344	0.0090	1.2	4497	1025	3674	1351	-1553

2190	PF10_0239		2.60	0.0180	0.0344	0.0090	1.3	4485	1418	3467	1505	-1906
1082	PFD0460c	conserved Plasmodium protein, unknown function	4.04	0.0020	0.0055	0.0018	1.4	4476	1123	3123	1341	-1111
1518	PFF0955c		3.37	0.0080	0.0173	0.0046	1.4	4465	1130	3232	1522	-1419
1043	PFE0225w	3-methyl-2-oxobutanoate dehydrogenase (lipoamide), pu	4.10	0.0020	0.0055	0.0018	1.4	4464	970	3125	1379	-1010
1901	PFE0260w	UDP-N-acetyl glucosamine:UMP antiporter	2.92	0.0020	0.0055	0.0018	1.4	4463	1126	3224	1851	-1739
1934	PFB0660w	conserved Plasmodium protein, unknown function	2.88	0.0040	0.0098	0.0026	1.3	4459	1106	3398	1547	-1592
246	PF07_0006	sporozoite threonine and asparagine-rich protein	6.04	0.0020	0.0055	0.0018	2.7	4455	1924	1671	1579	-719
892	PFD1210w	Plasmodium exported protein (PHISTa), unknown functio	4.37	0.0020	0.0055	0.0018	1.5	4451	1374	2969	1197	-1089
142	PF10_0142	conserved Plasmodium protein, unknown function	6.70	0.0020	0.0055	0.0018	1.5	4450	950	2940	762	-202
773	PF07_0083	small subunit rRNA processing protein, putative	4.58	0.0020	0.0055	0.0018	1.4	4444	822	3159	1187	-724
817	PF14_0183b	DEAD/DEAH box ATP-dependent RNA helicase, putative	4.49	0.0020	0.0055	0.0018	1.6	4439	1394	2755	1415	-1126
2026	PFF0250w	RNA binding protein, putative	2.78	0.0080	0.0173	0.0046	1.3	4429	1098	3417	1524	-1610
1339	MAL13P1.217	conserved Plasmodium protein, unknown function	3.63	0.0020	0.0055	0.0018	1.5	4427	1350	2981	1584	-1489
1109	MAL13P1.323	conserved Plasmodium protein, unknown function	4.00	0.0020	0.0055	0.0018	1.5	4415	1086	2946	1549	-1166
1316	MAL13P1.197	probable protein, unknown function	3.66	0.0020	0.0055	0.0018	1.5	4404	1114	2943	1715	-1369
1138	MAL13P1.50	conserved Plasmodium protein, unknown function	3.94	0.0020	0.0055	0.0018	1.5	4403	994	2941	1613	-1145
1413	PF14_0322	queuine tRNA-ribosyltransferase, putative	3.51	0.0020	0.0055	0.0018	1.6	4393	1369	2744	1994	-1714
2271	PFE1082c	conserved Plasmodium protein, unknown function	2.50	0.0140	0.0277	0.0073	1.3	4386	1079	3436	1623	-1751
1836	PFE0280c	conserved Plasmodium membrane protein, unknown fun	2.99	0.0060	0.0136	0.0036	1.2	4373	835	3560	1132	-1154
147	PFI0890c		6.66	0.0020	0.0055	0.0018	1.7	4371	1157	2541	931	-257
1101	PFF0965c	conserved Plasmodium membrane protein, unknown fun	4.02	0.0020	0.0055	0.0018	1.4	4365	931	3052	1395	-1012
455	PFC0610c	zinc finger protein, putative	5.31	0.0020	0.0055	0.0018	1.7	4360	1409	2574	1146	-768
2310	PF13_0202	conserved Plasmodium protein, unknown function	2.47	0.0240	0.0438	0.0115	1.2	4357	985	3592	1275	-1495
1402	PF07_0022	conserved Plasmodium protein, unknown function	3.54	0.0040	0.0098	0.0026	1.3	4357	748	3238	1418	-1047
604	MAL13P1.385	RNA binding protein, putative	4.94	0.0020	0.0055	0.0018	1.4	4350	596	3151	1077	-475
2033	MAL13P1.390	aminomethyltransferase, putative	2.78	0.0100	0.0208	0.0055	1.2	4331	875	3497	1273	-1315
1456	PFL2200w	conserved Plasmodium protein, unknown function	3.45	0.0020	0.0055	0.0018	1.3	4331	1013	3393	1024	-1099
1588	PFE1110w	conserved Plasmodium protein, unknown function	3.29	0.0020	0.0055	0.0018	1.3	4310	914	3211	1447	-1262
1838	PF14_0669	conserved Plasmodium protein, unknown function	2.99	0.0040	0.0098	0.0026	1.3	4306	1014	3271	1469	-1447
1221	MAL13P1.272	60S ribosomal protein L7-2, putative	3.81	0.0020	0.0055	0.0018	1.3	4291	897	3223	1148	-976
1242	PF08_0045	2-oxoglutarate dehydrogenase E1 component	3.78	0.0040	0.0098	0.0026	1.4	4282	1104	3057	1290	-1169
2260	PF14_0209	conserved Plasmodium protein, unknown function	2.51	0.0160	0.0311	0.0082	1.3	4275	1068	3283	1713	-1789
282	PFF1485w	conserved Plasmodium protein, unknown function	5.83	0.0020	0.0055	0.0018	1.6	4275	1055	2721	960	-461
1098	PF11_0484	conserved Plasmodium protein, unknown function	4.03	0.0040	0.0098	0.0026	1.4	4275	1003	3066	1205	-1000
1387	PFB0905c	Plasmodium exported protein (PHISTc), unknown functio	3.56	0.0020	0.0055	0.0018	1.5	4274	1391	2805	1652	-1574
1504	PF10_0177b	conserved Plasmodium protein, unknown function	3.38	0.0080	0.0173	0.0046	1.2	4268	1167	3446	675	-1020
1323	PF13_0339	conserved Plasmodium protein, unknown function	3.65	0.0040	0.0098	0.0026	1.4	4247	1385	3078	1050	-1266
2384	PF11_0315	conserved Plasmodium protein, unknown function	2.38	0.0240	0.0438	0.0115	1.2	4242	907	3436	1469	-1571
634	PFF0145w	conserved Plasmodium protein, unknown function	4.87	0.0020	0.0055	0.0018	1.6	4241	998	2657	1358	-771
1322	PFB0645c	mitochondrial ribosomal protein L13 precursor, putative	3.65	0.0040	0.0098	0.0026	1.6	4235	1480	2689	1658	-1593
212	PFE1025c	conserved Plasmodium protein, unknown function	6.21	0.0020	0.0055	0.0018	1.8	4235	1045	2416	1134	-360
1674	PF10_0378	RESA-like protein with PHIST and DnaJ domains	3.18	0.0060	0.0136	0.0036	1.4	4233	1153	3027	1586	-1533
1130	PFL0550w	HSP20-like chaperone	3.96	0.0020	0.0055	0.0018	1.4	4230	1291	2934	1181	-1176

1899	PFD0605c	conserved Plasmodium protein, unknown function	2.92	0.0060	0.0136	0.0036	1.2	4228	856	3520	943	-1092
471	PF07_0018	conserved Plasmodium membrane protein, unknown fun	5.26	0.0020	0.0055	0.0018	1.5	4224	541	2873	1174	-364
2152	PF14_0113	conserved Plasmodium protein, unknown function	2.63	0.0120	0.0243	0.0064	1.3	4220	1151	3342	1316	-1590
198	PF07_0095	tRNAHis guanylyltransferase, putative	6.29	0.0020	0.0055	0.0018	1.7	4216	939	2503	1076	-302
769	PF11_0381	subtilisin-like protease 2	4.59	0.0020	0.0055	0.0018	1.7	4214	1309	2547	1399	-1040
2300	PFE1485w	conserved Plasmodium protein, unknown function	2.48	0.0240	0.0438	0.0115	1.3	4207	1135	3302	1513	-1744
356	PFB0877c	probable protein, unknown function	5.58	0.0020	0.0055	0.0018	1.5	4204	810	2809	1020	-435
389	MAL13P1.178	conserved Plasmodium protein, unknown function	5.48	0.0020	0.0055	0.0018	1.8	4194	862	2324	1507	-500
578	PF14_0312	conserved Plasmodium membrane protein, unknown fun	4.99	0.0020	0.0055	0.0018	1.4	4193	827	2992	953	-579
431	MAL13P1.53	conserved Plasmodium protein, unknown function	5.37	0.0020	0.0055	0.0018	1.9	4162	823	2222	1631	-514
1053	PF14_0188	conserved Plasmodium membrane protein, unknown fun	4.08	0.0020	0.0055	0.0018	1.4	4155	958	3076	1014	-894
990	PFF1090c	conserved Plasmodium membrane protein, unknown fun	4.18	0.0020	0.0055	0.0018	1.5	4152	1106	2694	1434	-1082
2112	PF13_0249	conserved Plasmodium protein, unknown function	2.68	0.0080	0.0173	0.0046	1.3	4147	840	3239	1499	-1431
748	PFC0160w	palmitoyl transferase	4.63	0.0020	0.0055	0.0018	1.4	4138	743	3023	999	-628
1370	PFI0540w	conserved Plasmodium protein, unknown function	3.58	0.0040	0.0098	0.0026	1.4	4137	1000	2885	1491	-1239
1820	MAL13P1.340	conserved Plasmodium protein, unknown function	3.01	0.0080	0.0173	0.0046	1.3	4096	987	3222	1158	-1271
1529	MAL13P1.286	conserved Plasmodium protein, unknown function	3.35	0.0020	0.0055	0.0018	1.3	4090	871	3234	1016	-1030
1032	MAL8P1.8	prolyl 4-hydroxylase alpha subunit 1, putative	4.12	0.0020	0.0055	0.0018	1.4	4089	857	2858	1277	-902
525	PF11_0378	conserved Plasmodium protein, unknown function	5.10	0.0020	0.0055	0.0018	1.9	4084	1320	2197	1436	-868
1189	PF13_0256	conserved Plasmodium protein, unknown function	3.86	0.0020	0.0055	0.0018	1.5	4069	1105	2762	1374	-1172
1165	PFB0826c	conserved Plasmodium protein, unknown function	3.90	0.0020	0.0055	0.0018	1.3	4059	724	3064	1093	-822
509	PFI0310w	Maf-like protein, putative	5.15	0.0020	0.0055	0.0018	1.5	4057	834	2662	1125	-564
1432	PFA0235w	conserved Plasmodium protein, unknown function	3.48	0.0040	0.0098	0.0026	1.2	4051	528	3318	932	-727
2129	PF11_0281	protein phosphatase, putative	2.66	0.0200	0.0376	0.0099	1.3	4050	1236	3192	1191	-1568
1074	PFL1810w	conserved Plasmodium protein, unknown function	4.05	0.0020	0.0055	0.0018	1.7	4049	1617	2376	1500	-1444
629	PF13_0096	ubiquitin carboxyl-terminal hydrolase, putative	4.87	0.0020	0.0055	0.0018	1.4	4048	848	2862	957	-619
231	PFL0790w	conserved Plasmodium membrane protein, unknown fun	6.10	0.0020	0.0055	0.0018	1.9	4037	1228	2147	1113	-451
1705	PF14_0262	probable protein, unknown function	3.15	0.0040	0.0098	0.0026	1.2	4012	938	3272	840	-1039
670	PF13_0135	vacuolar protein sorting 52 homologue	4.78	0.0020	0.0055	0.0018	1.4	3988	631	2761	1141	-545
510	PFF1210w	phosphatidic acid phosphatase	5.15	0.0020	0.0055	0.0018	1.7	3983	990	2389	1269	-664
1477	PFI0135c	serine repeat antigen 9	3.41	0.0040	0.0098	0.0026	1.6	3981	1510	2557	1604	-1690
2286	PFC0305w	EB1 homolog, putative	2.49	0.0200	0.0376	0.0099	1.2	3979	803	3264	1231	-1319
1831	PFB0510w	GAF domain-related protein, putative	3.00	0.0040	0.0098	0.0026	1.4	3978	1138	2913	1455	-1528
1839	PFL0115w	dynein heavy chain, putative	2.99	0.0100	0.0208	0.0055	1.2	3977	810	3283	912	-1029
1321	PF14_0114	GTP binding protein, putative	3.65	0.0020	0.0055	0.0018	1.3	3971	733	3067	1044	-873
2263	PF14_0612	conserved Plasmodium protein, unknown function	2.51	0.0140	0.0277	0.0073	1.4	3962	1012	2899	1891	-1841
776	PF10_0071	rhoGAP GTPase, putative	4.58	0.0020	0.0055	0.0018	1.6	3941	1177	2478	1211	-926
1485	PFL2265c	conserved Plasmodium protein, unknown function	3.40	0.0060	0.0136	0.0036	1.3	3938	923	3038	1036	-1060
312	PF13_0211	calcium dependent protein kinase 5	5.74	0.0020	0.0055	0.0018	2.0	3935	1208	1934	1373	-580
812	PFL2300w	conserved Plasmodium protein, unknown function	4.49	0.0020	0.0055	0.0018	1.5	3932	1276	2590	996	-930
1033	PFA0270c	conserved Plasmodium protein, unknown function	4.12	0.0020	0.0055	0.0018	1.6	3919	1297	2413	1423	-1214
1970	PFD0830w	bifunctional dihydrofolate reductase-thymidylate synthas	2.85	0.0060	0.0136	0.0036	1.3	3906	982	2960	1402	-1437
609	MAL7P1.203	conserved Plasmodium protein, unknown function	4.94	0.0020	0.0055	0.0018	1.8	3889	1042	2164	1470	-788

2134	PFE0415w	transcription factor IIb, putative	2.66	0.0240	0.0438	0.0115	1.3	3877	998	2904	1585	-1611
1986	PFD1006w	conserved Plasmodium protein, unknown function	2.83	0.0140	0.0277	0.0073	1.4	3867	829	2834	1648	-1445
993	PF11_0095a	pterin-4a-carbinolamine dehydratase	4.18	0.0020	0.0055	0.0018	1.5	3862	1175	2518	1230	-1061
2009	PF14_0608	YL1 nuclear protein, putative	2.80	0.0100	0.0208	0.0055	1.2	3857	1097	3087	983	-1309
1894	PF14_0638	conserved Plasmodium membrane protein, unknown fun	2.93	0.0080	0.0173	0.0046	1.2	3856	859	3115	1008	-1126
318	MAL8P1.132	kinesin-like protein, putative	5.71	0.0020	0.0055	0.0018	2.3	3846	1302	1682	1500	-639
114	PFE1000c	conserved Plasmodium protein, unknown function	6.92	0.0020	0.0055	0.0018	2.0	3833	1013	1925	1049	-154
1417	PFC0075c	Plasmodium exported protein, unknown function	3.50	0.0040	0.0098	0.0026	1.6	3813	1904	2457	1020	-1568
2056	PF11_0459	apicoplast import protein Tic20, putative	2.75	0.0180	0.0344	0.0090	1.3	3798	1307	2824	1344	-1678
1679	PFE0445c	SNAP protein (soluble N-ethylmaleimide-sensitive factor	3.18	0.0040	0.0098	0.0026	1.5	3786	1068	2595	1603	-1481
427	MAL8P1.35	exonuclease, putative	5.39	0.0020	0.0055	0.0018	2.0	3781	1212	1932	1338	-701
1774	PFE0175c	myosin B	3.07	0.0080	0.0173	0.0046	1.4	3780	700	2774	1494	-1189
435	PF13_0270	conserved Plasmodium protein, unknown function	5.35	0.0020	0.0055	0.0018	2.0	3771	1065	1910	1450	-654
1428	PF14_0691	conserved Plasmodium membrane protein, unknown fun	3.49	0.0040	0.0098	0.0026	1.3	3768	918	2835	1061	-1046
1555	PF11_0284	methyltransferase, putative	3.33	0.0020	0.0055	0.0018	1.6	3759	1457	2354	1668	-1720
1042	PFL0850w	anaphase promoting complex subunit 10, putative	4.10	0.0040	0.0098	0.0026	1.5	3750	1151	2488	1158	-1046
2275	PF14_0667a	conserved Plasmodium protein, unknown function	2.50	0.0200	0.0376	0.0099	1.3	3743	950	2955	1319	-1482
1715	PF14_0694	protein disulfide isomerase	3.13	0.0060	0.0136	0.0036	1.4	3743	1134	2726	1277	-1395
482	PFD0360w	transcription factor, putative	5.22	0.0020	0.0055	0.0018	1.7	3728	1038	2230	1102	-642
2107	PF14_0582	conserved Plasmodium protein, unknown function	2.69	0.0160	0.0311	0.0082	1.3	3720	957	2923	1209	-1370
1769	PFD0695w	conserved Plasmodium protein, unknown function	3.07	0.0020	0.0055	0.0018	1.4	3689	1160	2544	1543	-1559
1480	MAL8P1.45	conserved Plasmodium protein, unknown function	3.41	0.0020	0.0055	0.0018	1.3	3668	764	2853	980	-929
1862	PFC0830w	trophozoite stage antigen	2.96	0.0120	0.0243	0.0064	1.4	3663	1119	2609	1468	-1532
1073	PFE0715w	aspartyl-tRNA synthetase, putative	4.05	0.0020	0.0055	0.0018	1.6	3658	987	2232	1512	-1073
330	PF14_0119	p1/s1 nuclease, putative	5.67	0.0020	0.0055	0.0018	2.1	3650	1264	1718	1287	-619
1931	PF11_0449	conserved Plasmodium protein, unknown function	2.88	0.0080	0.0173	0.0046	1.2	3649	661	2997	958	-967
1025	MAL8P1.47	conserved Plasmodium protein, unknown function	4.12	0.0020	0.0055	0.0018	1.6	3647	1314	2219	1288	-1173
1186	PFL1350w	RNA pseudouridylate synthase, putative	3.87	0.0020	0.0055	0.0018	1.4	3645	936	2669	954	-914
1620	PFE1395c	conserved Plasmodium protein, unknown function	3.25	0.0040	0.0098	0.0026	1.6	3633	1154	2276	1803	-1600
317	MAL13P1.186	1-deoxy-D-xylulose 5-phosphate synthase	5.71	0.0020	0.0055	0.0018	1.4	3594	823	2486	652	-367
934	PF13_0351	conserved Plasmodium protein, unknown function	4.29	0.0020	0.0055	0.0018	1.5	3578	1153	2463	825	-863
2066	PF10_0131	conserved Plasmodium protein, unknown function	2.74	0.0080	0.0173	0.0046	1.2	3571	858	3002	717	-1006
359	PF11_0545	conserved Plasmodium protein, unknown function	5.57	0.0020	0.0055	0.0018	1.8	3546	1419	1976	699	-548
1293	PFL1135c	conserved Plasmodium protein, unknown function	3.69	0.0040	0.0098	0.0026	1.4	3545	723	2555	1166	-899
1710	PFL1305c	conserved Plasmodium protein, unknown function	3.13	0.0040	0.0098	0.0026	1.4	3519	1076	2437	1431	-1424
2041	PF10_0056	conserved Plasmodium protein, unknown function	2.77	0.0120	0.0243	0.0064	1.4	3503	1860	2424	1097	-1878
432	PFE1525w	conserved Plasmodium membrane protein, unknown fun	5.36	0.0020	0.0055	0.0018	1.6	3477	959	2186	861	-529
592	PF08_0008	GPI-anchored micronemal antigen	4.97	0.0020	0.0055	0.0018	1.8	3474	1158	1912	1195	-791
1399	PF10_0106	conserved Plasmodium protein, unknown function	3.54	0.0020	0.0055	0.0018	1.6	3471	1055	2194	1528	-1306
882	PFC0750w	conserved Plasmodium protein, unknown function	4.38	0.0020	0.0055	0.0018	1.5	3462	1094	2271	963	-866
2323	PF13_0056	conserved Plasmodium protein, unknown function	2.46	0.0120	0.0243	0.0064	1.3	3453	841	2729	1259	-1376
1170	PF14_0601	replication factor C3	3.89	0.0020	0.0055	0.0018	1.4	3448	985	2458	931	-927
2341	PF14_0477a	conserved Plasmodium protein, unknown function	2.43	0.0160	0.0311	0.0082	1.4	3447	1506	2442	1580	-2082

716	PF13_0258	serine/threonine protein kinase	4.70	0.0020	0.0055	0.0018	1.5	3443	859	2225	1046	-687
763	MAL8P1.30	conserved Plasmodium membrane protein, unknown fun	4.60	0.0020	0.0055	0.0018	1.7	3426	1023	2012	1235	-844
1078	MAL13P1.297	ADP-ribosylation factor, putative	4.05	0.0020	0.0055	0.0018	1.7	3409	1326	2032	1241	-1189
569	PF14_0498	DER1-like protein	5.01	0.0020	0.0055	0.0018	2.1	3405	1589	1593	1163	-940
1179	PFD0415c	conserved Plasmodium protein, unknown function	3.88	0.0020	0.0055	0.0018	1.6	3397	1122	2095	1346	-1167
1318	PF10_0157	conserved Plasmodium protein, unknown function	3.66	0.0020	0.0055	0.0018	1.6	3386	1137	2074	1474	-1299
2035	PF14_0366	small subunit DNA primase	2.78	0.0220	0.0408	0.0107	1.3	3368	799	2657	1060	-1148
1121	PFL0380c	tRNA delta(2)-isopentenylpyrophosphate transferase, pu	3.98	0.0020	0.0055	0.0018	1.5	3357	983	2175	1200	-1000
184	PF14_0572	conserved Plasmodium membrane protein, unknown fun	6.39	0.0020	0.0055	0.0018	1.9	3352	1005	1771	869	-293
1348	PFE1505w	SNARE protein, putative	3.63	0.0040	0.0098	0.0026	1.5	3314	1127	2226	1123	-1163
1233	PFC0565w	GTP-binding protein EngA, putative	3.79	0.0020	0.0055	0.0018	1.5	3312	1018	2241	1088	-1036
607	PF14_0451	mitochondrial ribosomal protein S14 precursor, putative	4.94	0.0020	0.0055	0.0018	1.6	3309	1030	2073	867	-662
1752	PFD1130w	conserved Plasmodium protein, unknown function	3.09	0.0060	0.0136	0.0036	1.5	3303	1360	2185	1357	-1598
1301	PFL1725w	ATP synthase beta chain, mitochondrial precursor, putati	3.68	0.0020	0.0055	0.0018	1.5	3302	1327	2197	963	-1184
729	PF14_0562	conserved protein, unknown function	4.68	0.0020	0.0055	0.0018	1.6	3295	776	2121	1042	-644
950	PFL1190c	conserved Plasmodium protein, unknown function	4.26	0.0020	0.0055	0.0018	1.7	3284	1632	1989	580	-917
2436	MAL13P1.11	Plasmodium exported protein (PHISTa), unknown functio	2.32	0.0180	0.0344	0.0090	1.8	3284	2627	1852	2067	-3262
67	PFC0905c	oocyst capsule protein	7.46	0.0020	0.0055	0.0018	3.5	3263	1382	937	992	-49
1791	PF08_0112	conserved Plasmodium protein, unknown function	3.04	0.0060	0.0136	0.0036	1.4	3235	959	2382	1111	-1217
173	PF14_0215	ubiquitin ligase, putative	6.45	0.0020	0.0055	0.0018	1.9	3225	1047	1712	738	-272
227	PFL1275c	conserved Plasmodium protein, unknown function	6.11	0.0020	0.0055	0.0018	1.8	3221	740	1820	935	-274
707	PF13_0181	conserved Plasmodium protein, unknown function	4.71	0.0020	0.0055	0.0018	1.5	3202	804	2181	817	-601
1902	PFE0365c	conserved Plasmodium protein, unknown function	2.92	0.0060	0.0136	0.0036	1.5	3199	1283	2080	1539	-1703
857	MAL13P1.33	conserved Plasmodium protein, unknown function	4.42	0.0020	0.0055	0.0018	1.4	3171	664	2214	901	-608
906	PFB1025w	erythrocyte membrane protein 1 (PfEMP1), truncated, de	4.35	0.0020	0.0055	0.0018	1.8	3158	1353	1783	1054	-1031
1722	MAL13P1.182	conserved Plasmodium protein, unknown function	3.12	0.0060	0.0136	0.0036	1.4	3148	1136	2278	978	-1244
2188	PF10_0017	Plasmodium exported protein (PHISTa), unknown functio	2.60	0.0299	0.0530	0.0139	1.6	2292	1290	1460	1135	-1592
714	PFC0405c	ribosomal protein l7ae, putative	4.70	0.0020	0.0055	0.0018	1.9	3140	1170	1638	1220	-888
2174	PFI0285w	conserved Plasmodium protein, unknown function	2.61	0.0180	0.0344	0.0090	1.3	3137	1138	2392	1021	-1413
1108	PF07_0098	dynactin 4, putative	4.01	0.0020	0.0055	0.0018	1.7	3119	1107	1871	1210	-1069
1063	PF10_0148	conserved protein, unknown function	4.07	0.0020	0.0055	0.0018	1.8	3094	1509	1690	1117	-1223
804	PF08_0069	importin beta, putative	4.52	0.0020	0.0055	0.0018	2.2	3087	1587	1410	1237	-1148
208	PFF1005w	conserved Plasmodium protein, unknown function	6.23	0.0020	0.0055	0.0018	2.6	3085	1439	1210	847	-410
428	PF11_0350		5.39	0.0020	0.0055	0.0018	2.0	3077	1268	1524	927	-642
1778	PFB0670c	conserved Plasmodium protein, unknown function	3.06	0.0060	0.0136	0.0036	1.5	3074	1004	2090	1331	-1350
370	PFI1655c	conserved Plasmodium protein, unknown function	5.53	0.0020	0.0055	0.0018	2.0	3053	959	1503	1113	-521
2087	MAL13P1.134	DEAD box helicase, putative	2.71	0.0120	0.0243	0.0064	1.3	3043	1004	2322	992	-1275
1800	PF14_0460		3.03	0.0100	0.0208	0.0055	1.3	3039	842	2327	906	-1037
2077	PFI0860c	ATP-dependent RNA helicase, putative	2.73	0.0140	0.0277	0.0073	1.4	3035	1204	2247	991	-1406
981	PFL2115c	glucose inhibited division protein a homologue, putative	4.20	0.0020	0.0055	0.0018	1.5	3029	914	1962	982	-828
883	PF13_0079	conserved Plasmodium protein, unknown function	4.38	0.0020	0.0055	0.0018	1.7	3024	910	1735	1223	-844
453	PFL1870c	sphingomyelin phosphodiesterase, putative	5.31	0.0020	0.0055	0.0018	2.0	3018	893	1522	1157	-554
1237	PF13_0041	conserved Plasmodium protein, unknown function	3.78	0.0020	0.0055	0.0018	1.7	3014	1173	1819	1193	-1171

2319	PF10_0230	conserved Plasmodium protein, unknown function	2.46	0.0240	0.0438	0.0115	1.3	3010	930	2253	1292	-1464
1963	PF10_0044	hypothetical protein	2.85	0.0140	0.0277	0.0073	1.3	2997	1163	2252	824	-1242
858	PFI0710c	conserved Plasmodium protein, unknown function	4.42	0.0020	0.0055	0.0018	1.6	2980	687	1920	1023	-650
524	PF11_0419	conserved Plasmodium protein, unknown function	5.10	0.0020	0.0055	0.0018	1.8	2974	1075	1695	831	-627
1004	PFE1480c	conserved Plasmodium protein, unknown function	4.16	0.0020	0.0055	0.0018	1.7	2944	1309	1729	916	-1010
1120	PF11_0118	conserved Plasmodium protein, unknown function	3.98	0.0020	0.0055	0.0018	1.7	2942	987	1693	1295	-1033
940	PF13_0055	conserved Plasmodium protein, unknown function	4.28	0.0020	0.0055	0.0018	1.7	2935	971	1755	1077	-868
1152	PFF1420w	phosphatidylcholine-sterol acyltransferase precursor, put	3.92	0.0020	0.0055	0.0018	2.0	2895	1446	1464	1313	-1329
1446	PF11_0119	SNARE protein, putative	3.46	0.0020	0.0055	0.0018	1.5	2884	973	1981	981	-1052
2233	PF13_0198	reticulocyte binding protein 2 homologue a	2.54	0.0379	0.0651	0.0171	1.7	2073	1044	1231	1363	-1566
248	PFI1045w	conserved Plasmodium protein, unknown function	6.02	0.0020	0.0055	0.0018	1.9	2883	941	1485	816	-360
674	PFL1590c	elongation factor G, putative	4.77	0.0020	0.0055	0.0018	1.6	2876	1184	1744	600	-652
1030	PF14_0311	SF-assemblin, putative	4.12	0.0020	0.0055	0.0018	1.6	2856	1039	1802	900	-885
1667	PF10_0027	conserved Plasmodium protein, unknown function	3.19	0.0040	0.0098	0.0026	1.3	2855	580	2235	818	-778
1245	PFD1080w	conserved Plasmodium protein, unknown function	3.77	0.0020	0.0055	0.0018	1.4	2833	805	2074	716	-762
1535	PFE0045c	serine/threonine protein kinase, FIKK family	3.34	0.0120	0.0243	0.0064	1.4	2827	1048	1958	919	-1098
1180	PFL2650w	Plasmodium exported protein, unknown function, fragme	3.87	0.0020	0.0055	0.0018	2.3	2819	1674	1222	1448	-1525
1771	PFB0467w	50S ribosomal protein L33, putative	3.07	0.0020	0.0055	0.0018	1.4	2811	770	1980	1157	-1096
1185	PFL2125c	transcription factor Tfb2, putative	3.87	0.0020	0.0055	0.0018	1.7	2787	1022	1633	1183	-1051
305	PF11_0510	serine/threonine protein kinase, FIKK family	5.77	0.0020	0.0055	0.0018	1.8	2786	835	1575	753	-377
394	MAL13P1.281	glutamate--tRNA ligase, putative	5.47	0.0020	0.0055	0.0018	2.0	2783	986	1366	961	-530
1212	MAL13P1.365	syntaxin, Qa-SNARE family	3.82	0.0020	0.0055	0.0018	1.6	2778	1151	1752	891	-1017
1690	PFE1125w	mitochondrial ribosomal protein L17 precursor, putative	3.16	0.0100	0.0208	0.0055	1.5	2778	1144	1849	1070	-1285
807	PF11_0125	conserved Plasmodium protein, unknown function	4.51	0.0020	0.0055	0.0018	1.6	2745	949	1691	821	-716
2059	PFD0790c	DNA replication licensing factor, putative	2.75	0.0120	0.0243	0.0064	1.2	2744	778	2212	688	-934
1388	PF07_0019	conserved Plasmodium protein, unknown function	3.56	0.0020	0.0055	0.0018	1.5	2736	829	1861	983	-937
1856	PFF0795w	conserved Plasmodium protein, unknown function	2.97	0.0080	0.0173	0.0046	1.3	2734	840	2079	818	-1003
2261	PFI0900w	conserved Plasmodium protein, unknown function	2.51	0.0359	0.0621	0.0163	1.2	2343	695	1889	665	-906
1001	PFF0820w	conserved Plasmodium protein, unknown function	4.16	0.0020	0.0055	0.0018	1.9	2723	1116	1449	1170	-1012
888	PFL0370w	conserved Plasmodium protein, unknown function	4.37	0.0020	0.0055	0.0018	1.6	2705	916	1714	802	-727
1824	PF11_0368	conserved Plasmodium protein, unknown function	3.01	0.0040	0.0098	0.0026	1.3	2678	594	2033	925	-874
2364	PF11_0299		2.40	0.0259	0.0469	0.0123	1.2	2669	599	2151	928	-1009
1972	PF14_0430	mitochondrial ribosomal protein S29 precursor, putative	2.85	0.0100	0.0208	0.0055	1.3	2636	761	1998	891	-1014
632	PFL2560c	Plasmodium exported protein, unknown function	4.87	0.0020	0.0055	0.0018	2.8	2627	1718	949	879	-918
693	PF11_0140	conserved Plasmodium protein, unknown function	4.74	0.0020	0.0055	0.0018	2.0	2591	1143	1327	886	-764
755	PF07_0116	conserved Plasmodium protein, unknown function	4.62	0.0020	0.0055	0.0018	2.0	2584	1048	1284	1061	-809
1768	MAL13P1.169	syntaxin, Qa-SNARE family	3.07	0.0040	0.0098	0.0026	1.4	2578	883	1789	1016	-1110
734	PF14_0354	pantothenate kinase, putative	4.66	0.0020	0.0055	0.0018	1.9	2575	769	1384	1071	-649
1804	PF11_0095	D-tyrosyl-tRNA(Tyr) deacylase, putative	3.02	0.0080	0.0173	0.0046	1.5	2568	1044	1712	1073	-1261
2179	MAL13P1.165	GPI transamidase subunit PIG-U, putative	2.61	0.0160	0.0311	0.0082	1.3	2568	824	2007	794	-1057
1738	PF11_0335		3.11	0.0100	0.0208	0.0055	1.4	2561	607	1854	993	-892
187	MAL7P1.23	RAP protein, putative	6.38	0.0020	0.0055	0.0018	2.4	2554	939	1080	811	-276
1244	MAL13P1.342a	conserved Plasmodium protein, unknown function	3.77	0.0020	0.0055	0.0018	2.2	2538	1400	1168	1333	-1364

2296	PFD1200c	Plasmodium exported protein (hyp6), unknown function	2.48	0.0299	0.0530	0.0139	2.9	1361	1831	471	845	-1787
357	MAL13P1.46	conserved Plasmodium protein, unknown function	5.57	0.0020	0.0055	0.0018	2.0	2531	1055	1294	630	-448
797	PFA0305c	conserved Plasmodium protein, unknown function	4.53	0.0020	0.0055	0.0018	2.1	2517	918	1215	1179	-796
2309	PF14_0687		2.47	0.0180	0.0344	0.0090	1.3	2504	1131	1860	855	-1342
2208	MAL13P1.177	conserved Plasmodium protein, unknown function	2.57	0.0080	0.0173	0.0046	1.3	2500	532	1951	946	-929
341	PFD0325w	conserved Plasmodium protein, unknown function	5.62	0.0020	0.0055	0.0018	2.8	2496	1524	906	527	-461
1382	PF07_0011	conserved Plasmodium protein, unknown function	3.56	0.0040	0.0098	0.0026	1.6	2490	1073	1523	977	-1084
931	MAL8P1.91	phospholipase DDHD1, putative	4.30	0.0020	0.0055	0.0018	2.5	2466	1480	976	1156	-1146
962	PFC0445w	sybindin-like protein, putative	4.23	0.0020	0.0055	0.0018	1.6	2441	702	1530	874	-665
720	PF10_0331	Sec1 family protein, putative	4.69	0.0020	0.0055	0.0018	2.2	2437	1014	1108	1097	-781
1076	PF13_0012	early transcribed membrane protein 13	4.05	0.0020	0.0055	0.0018	1.6	2437	917	1523	794	-796
344	PFD0400w	conserved Plasmodium protein, unknown function	5.62	0.0020	0.0055	0.0018	2.0	2406	989	1181	672	-435
853	PF14_0255a	conserved Plasmodium protein, unknown function	4.43	0.0020	0.0055	0.0018	2.3	2383	1196	1042	1092	-947
2313	PFE0210c	conserved Plasmodium protein, unknown function	2.46	0.0339	0.0590	0.0155	1.5	766	375	497	432	-538
1427	PF13_0039	conserved Plasmodium protein, unknown function	3.49	0.0040	0.0098	0.0026	1.7	2377	927	1400	1131	-1081
943	PFI1155w	conserved Plasmodium protein, unknown function	4.27	0.0020	0.0055	0.0018	1.4	2367	624	1729	511	-497
1644	PF14_0212	mitochondrial ribosomal protein L21 precursor, putative	3.22	0.0020	0.0055	0.0018	1.4	2347	875	1662	738	-929
1435	PFL2205w	conserved Plasmodium protein, unknown function	3.48	0.0040	0.0098	0.0026	1.6	2346	1277	1437	700	-1067
264	PFE0805w	cation-transporting ATPase 1	5.94	0.0020	0.0055	0.0018	1.9	2334	695	1213	717	-290
1425	PFL1285c	proliferating cell nuclear antigen 2	3.49	0.0040	0.0098	0.0026	1.8	2319	1173	1292	1054	-1200
1657	PFE0035c	rifin, pseudogene	3.20	0.0060	0.0136	0.0036	1.9	2289	1293	1208	1247	-1460
779	MAL13P1.100	conserved Plasmodium protein, unknown function	4.57	0.0020	0.0055	0.0018	1.6	2285	843	1386	655	-598
1359	PF14_0656	U2 snRNP auxiliary factor, putative	3.60	0.0020	0.0055	0.0018	1.9	2261	1060	1179	1173	-1151
1530	PFF0680c	thiamin-phosphate pyrophosphorylase, putative	3.35	0.0020	0.0055	0.0018	1.6	2243	1234	1367	754	-1111
1498	PFI1675w	conserved Plasmodium protein, unknown function	3.39	0.0020	0.0055	0.0018	1.3	2240	666	1764	400	-590
452	PF10_0124	protein phosphatase, putative	5.31	0.0020	0.0055	0.0018	2.2	2225	1055	1013	682	-524
157	PF11_0405		6.59	0.0020	0.0055	0.0018	3.1	2205	1140	712	562	-209
2050	PFE0710w	conserved Plasmodium protein, unknown function	2.75	0.0100	0.0208	0.0055	1.6	2179	1289	1341	1026	-1477
1712	PF14_0043		3.13	0.0040	0.0098	0.0026	1.5	2165	974	1458	747	-1013
2345	PF10_0076	conserved Plasmodium protein, unknown function	2.42	0.0419	0.0710	0.0187	1.5	1569	805	1069	751	-1056
699	PF14_0025	proteosome subunit, putative	4.73	0.0020	0.0055	0.0018	1.9	2152	894	1150	718	-609
2347	PF11_0175a	conserved Plasmodium protein, unknown function	2.42	0.0359	0.0621	0.0163	1.3	1662	642	1251	632	-864
2348	PFL1265c	conserved Plasmodium protein, unknown function	2.42	0.0339	0.0590	0.0155	1.4	3833	2011	2758	1369	-2305
2349	PFL2155w	conserved Plasmodium protein, unknown function	2.42	0.0339	0.0590	0.0155	1.9	848	795	458	420	-825
1246	PF14_0483	conserved Plasmodium protein, unknown function	3.77	0.0020	0.0055	0.0018	1.8	2145	1098	1197	817	-966
2418	MAL13P1.184	endopeptidase, putative	2.34	0.0240	0.0438	0.0115	1.4	2144	943	1517	1045	-1361
1673	PFC0625w	conserved Plasmodium membrane protein, unknown fun	3.18	0.0060	0.0136	0.0036	1.6	2141	1007	1299	983	-1148
1653	PFI0790w	thioredoxin reductase 3	3.20	0.0040	0.0098	0.0026	1.4	2129	718	1517	716	-822
2314	PFB0315w	conserved Plasmodium protein, unknown function	2.46	0.0220	0.0408	0.0107	1.4	2103	933	1544	788	-1163
1815	PF13_0327	cytochrome c oxidase subunit 2, putative	3.01	0.0060	0.0136	0.0036	1.3	2076	680	1552	633	-789
445	PF10_0297	conserved Plasmodium protein, unknown function	5.32	0.0020	0.0055	0.0018	1.9	2066	668	1083	710	-394
2303	PFF0210w	conserved Plasmodium protein, unknown function	2.47	0.0180	0.0344	0.0090	1.3	2064	764	1555	777	-1032
2105	PFB0190c	conserved Plasmodium protein, unknown function	2.70	0.0259	0.0469	0.0123	1.8	2047	953	1155	1410	-1471

809	MAL13P1.194	conserved Plasmodium protein, unknown function	4.51	0.0020	0.0055	0.0018	2.5	2038	1475	815	477	-730
825	PF14_0121	conserved Plasmodium protein, unknown function	4.47	0.0020	0.0055	0.0018	2.1	2020	1289	948	454	-671
2117	MAL13P1.331	conserved Plasmodium protein, unknown function	2.67	0.0259	0.0469	0.0123	1.4	2005	794	1442	787	-1019
235	PF10_0224	dynein heavy chain, putative	6.08	0.0020	0.0055	0.0018	2.8	1998	1067	724	500	-293
463	PFL0685w	phosphatidylinositol-glycan biosynthesis class O protein,	5.27	0.0020	0.0055	0.0018	2.0	1990	622	987	772	-391
2158	PF08_0046	conserved Plasmodium protein, unknown function	2.62	0.0180	0.0344	0.0090	1.4	1978	692	1445	807	-967
2407	PFL1947c	erythrocyte membrane protein 1 (PfEMP1), pseudogene	2.36	0.0220	0.0408	0.0107	1.4	1975	1175	1389	713	-1302
784	PF14_0435	conserved Plasmodium membrane protein, unknown fun	4.56	0.0020	0.0055	0.0018	1.6	1930	625	1204	575	-475
642	PF11_0219	conserved Plasmodium protein, unknown function	4.84	0.0020	0.0055	0.0018	1.5	1926	654	1245	417	-390
620	PFB0965c	Plasmodium exported protein, unknown function, pseudo	4.90	0.0020	0.0055	0.0018	2.1	1904	936	895	633	-559
1547	PF10_0042	conserved Plasmodium protein, unknown function	3.33	0.0020	0.0055	0.0018	1.5	1895	838	1240	658	-840
1524	PF08_0090	conserved Plasmodium protein, unknown function	3.36	0.0060	0.0136	0.0036	1.5	1892	724	1232	747	-811
2452	PF07_0108	conserved Plasmodium protein, unknown function	2.31	0.0180	0.0344	0.0090	1.3	1854	691	1428	694	-959
2386	PFF0800w	TRAP-like protein	2.38	0.0399	0.0680	0.0179	1.3	3616	1114	2827	1329	-1653
894	MAL8P1.137	conserved Plasmodium protein, unknown function	4.36	0.0020	0.0055	0.0018	1.9	1849	889	991	607	-639
1343	PFI0336w	conserved Plasmodium protein, unknown function	3.63	0.0040	0.0098	0.0026	1.9	1841	1053	966	780	-959
679	PFE1280w	conserved Plasmodium protein, unknown function	4.77	0.0020	0.0055	0.0018	2.1	1813	900	876	597	-560
1434	PFC0990c	conserved Plasmodium protein, unknown function	3.48	0.0040	0.0098	0.0026	1.7	1794	740	1072	804	-822
1687	PFB0360c	serine repeat antigen 1	3.17	0.0020	0.0055	0.0018	1.6	1767	976	1110	602	-921
897	PF13_0306	dynein light chain, putative	4.36	0.0020	0.0055	0.0018	2.9	1765	991	615	989	-830
1663	MAL13P1.239	conserved Plasmodium protein, unknown function	3.19	0.0080	0.0173	0.0046	1.5	1761	600	1209	680	-728
2397	PF11_0371	conserved Plasmodium protein, unknown function	2.37	0.0279	0.0501	0.0132	1.2	4507	1009	3622	1623	-1746
2256	PF14_0120	conserved Plasmodium protein, unknown function	2.52	0.0220	0.0408	0.0107	1.9	1750	1629	936	797	-1612
2400	PFC0490w	conserved Plasmodium protein, unknown function	2.36	0.0319	0.0561	0.0147	1.2	5513	1168	4605	1607	-1867
180	PFA0120c	lysophospholipase, putative	6.40	0.0020	0.0055	0.0018	2.8	1744	884	631	417	-188
1826	PF14_0275	conserved Plasmodium protein, unknown function	3.00	0.0040	0.0098	0.0026	1.5	1742	871	1146	641	-917
2138	PFI1730w	cytoadherence linked asexual protein 9	2.65	0.0100	0.0208	0.0055	1.4	1719	597	1198	820	-897
2408	PFF0140c	conserved Plasmodium protein, unknown function	2.36	0.0319	0.0561	0.0147	1.6	1701	1242	1061	829	-1430
1720	PF10_0011		3.12	0.0100	0.0208	0.0055	2.4	1707	1448	712	980	-1432
2413	PFC0571c		2.35	0.0399	0.0680	0.0179	1.3	1956	720	1501	732	-997
2414	PFF0300w	RNA binding protein, putative	2.35	0.0279	0.0501	0.0132	1.0	9158	113	9023	266	-243
2416	MAL13P1.276	conserved Plasmodium protein, unknown function	2.34	0.0499	0.0823	0.0216	1.3	2486	1167	1860	865	-1406
815	PFL0250w	conserved Plasmodium protein, unknown function	4.49	0.0020	0.0055	0.0018	1.8	1680	706	931	561	-519
1285	PF11_0152	GTPase activator, putative	3.70	0.0020	0.0055	0.0018	3.0	1667	1430	549	867	-1178
2420	PF07_0092	nucleolar rRNA processing protein, putative	2.34	0.0319	0.0561	0.0147	1.0	8980	224	8798	330	-372
2421	PFD1020c	rifin	2.34	0.0319	0.0561	0.0147	1.6	1618	1134	1038	757	-1311
534	PFA0350w		5.08	0.0020	0.0055	0.0018	1.9	1657	665	877	504	-389
2423	PF14_0452a	conserved Plasmodium protein, unknown function	2.33	0.0319	0.0561	0.0147	1.2	6992	1057	6031	1812	-1908
2425	PF10_0265	conserved Plasmodium protein, unknown function	2.33	0.0339	0.0590	0.0155	1.4	1873	1026	1350	680	-1183
1586	MAL7P1.65	conserved Plasmodium protein, unknown function	3.29	0.0060	0.0136	0.0036	1.9	1650	1221	866	558	-995
2432	PF08_0108	plasmepsin X	2.32	0.0299	0.0530	0.0139	1.1	5549	752	4841	1353	-1397
1325	PFL1535w	conserved Plasmodium protein, unknown function	3.65	0.0040	0.0098	0.0026	1.7	1647	734	988	632	-708
905	PFL0955c	raf kinase inhibitor	4.35	0.0020	0.0055	0.0018	1.9	1636	779	869	564	-576

2440	MAL13P1.111	ATP-dependent Clp protease adaptor protein ClpS, putati	2.32	0.0379	0.0651	0.0171	1.3	2404	822	1857	926	-1202
1041	PF10_0040	conserved Plasmodium protein, unknown function	4.10	0.0040	0.0098	0.0026	1.7	1606	698	941	537	-569
1418	PF14_0788		3.50	0.0040	0.0098	0.0026	1.8	1595	1029	897	453	-785
2401	PFB0690w	conserved Plasmodium protein, unknown function	2.36	0.0220	0.0408	0.0107	1.2	1589	484	1295	454	-644
1751	PF14_0467	conserved Plasmodium protein, unknown function	3.09	0.0060	0.0136	0.0036	1.7	1586	887	939	703	-943
2454	PF11_0139	protein tyrosine phosphatase	2.30	0.0299	0.0530	0.0139	1.0	9709	50	9673	65	-79
1968	PF10_0152	conserved Plasmodium protein, unknown function	2.85	0.0100	0.0208	0.0055	1.7	1565	897	909	837	-1078
2137	PFA0495c	selenocysteine-specific elongation factor selB homologue	2.65	0.0080	0.0173	0.0046	1.7	1562	1080	932	728	-1178
2463	PF13_0061	ATP synthase gamma chain, mitochondrial precursor, put	2.30	0.0399	0.0680	0.0179	1.2	3089	774	2556	933	-1174
1166	PFE1070c	conserved Plasmodium protein, unknown function	3.90	0.0020	0.0055	0.0018	2.1	1558	727	759	798	-726
2471	PFD1150c	reticulocyte binding protein homologue 4	2.29	0.0299	0.0530	0.0139	1.3	1172	576	869	430	-704
2472	PF11_0523	erythrocyte membrane protein 1 (PfEMP1), truncated, ps	2.29	0.0319	0.0561	0.0147	1.6	1852	1435	1152	889	-1624
520	PF13_0302	phosphatase 2A regulatory subunit-related protein, putat	5.11	0.0020	0.0055	0.0018	2.1	1549	733	738	473	-396
1714	PFI0655c	conserved Plasmodium protein, unknown function	3.13	0.0080	0.0173	0.0046	1.8	1526	1121	870	413	-878
1381	PF14_0703	conserved Plasmodium protein, unknown function	3.57	0.0020	0.0055	0.0018	1.9	1522	849	798	695	-819
1740	PF10_0415	conserved Plasmodium protein, unknown function	3.10	0.0060	0.0136	0.0036	1.9	1518	836	797	896	-1010
1929	PFF0840w	conserved Plasmodium protein, unknown function	2.89	0.0140	0.0277	0.0073	1.9	1491	846	798	937	-1090
1688	MAL7P1.25	cytoskeleton associated protein, putative	3.17	0.0020	0.0055	0.0018	1.7	1430	976	833	423	-803
1785	PFL0765w	conserved Plasmodium membrane protein, unknown fun	3.05	0.0080	0.0173	0.0046	1.6	1374	594	858	663	-740
2489	PFI1240c	prolyl-t-RNA synthase, putative	2.27	0.0439	0.0739	0.0194	1.2	3453	717	2787	1307	-1358
419	PF14_0371	conserved Plasmodium protein, unknown function	5.40	0.0020	0.0055	0.0018	2.0	1350	481	688	444	-263
1155	MAL13P1.16	SNARE protein, putative	3.91	0.0040	0.0098	0.0026	1.5	1339	531	871	381	-443
1917	PF14_0729	early transcribed membrane protein 14.2	2.91	0.0120	0.0243	0.0064	1.5	1338	680	886	504	-732
1900	MAL8P1.149	conserved Plasmodium protein, unknown function	2.92	0.0080	0.0173	0.0046	1.6	1336	724	834	582	-804
992	MAL7P1.164	adapter-related protein, putative	4.18	0.0020	0.0055	0.0018	2.7	1331	1038	489	462	-658
681	MAL7P1.73	calcium/calmodulin-dependent protein kinase, putative	4.76	0.0020	0.0055	0.0018	1.7	1310	564	759	312	-324
2505	PFL0785c	signal recognition particle SRP19	2.25	0.0479	0.0797	0.0209	1.1	6927	595	6527	711	-906
2506	PF11_0097	succinyl-CoA synthetase alpha subunit, putative	2.25	0.0279	0.0501	0.0132	1.1	5532	789	4878	1260	-1395
2507	PFA0425c	conserved Plasmodium protein, unknown function	2.25	0.0279	0.0501	0.0132	1.2	3762	822	3167	1095	-1322
2508	PFI1560c	conserved Plasmodium membrane protein, unknown fun	2.25	0.0299	0.0530	0.0139	1.1	6984	754	6298	1354	-1421
2512	PFL0185c	nucleosome assembly protein	2.25	0.0279	0.0501	0.0132	1.0	9808	85	9750	105	-132
1531	PFB0110w	Plasmodium exported protein (hyp11), unknown function	3.35	0.0020	0.0055	0.0018	3.7	1303	1634	350	233	-915
2069	PFB0925w	DnaJ protein, putative	2.74	0.0100	0.0208	0.0055	1.8	1241	881	694	640	-974
2517	PF10_0237	conserved Plasmodium protein, unknown function	2.24	0.0319	0.0561	0.0147	1.2	4511	827	3905	1127	-1348
373	PF14_0179	liver specific protein 1, putative	5.52	0.0020	0.0055	0.0018	3.5	1241	839	350	360	-307
1685	PFI0520w	actin-like protein, putative	3.17	0.0100	0.0208	0.0055	1.5	1225	573	819	402	-569
484	PF10_0216	zinc transporter, putative	5.22	0.0020	0.0055	0.0018	2.2	1215	581	540	404	-310
2524	PFB0423c	conserved protein, unknown function	2.23	0.0299	0.0530	0.0139	1.1	7610	689	6962	1300	-1340
2525	PFF1110c	coronin binding protein, putative	2.23	0.0379	0.0651	0.0171	1.0	8593	219	8454	245	-325
1995	PFD0440w	peptidase, M22 family, putative	2.82	0.0100	0.0208	0.0055	1.7	1203	817	712	508	-834
2529	PF13_0093	conserved Plasmodium protein, unknown function	2.23	0.0339	0.0590	0.0155	1.1	6769	821	6077	1356	-1486
545	PFL2080c	conserved Plasmodium protein, unknown function	5.06	0.0020	0.0055	0.0018	2.0	1175	495	577	403	-300
2532	PFI0460w	conserved Plasmodium protein, unknown function	2.22	0.0319	0.0561	0.0147	1.2	5595	991	4811	1513	-1720

1790	PF11_0298	GPI8p transamidase	3.04	0.0100	0.0208	0.0055	1.6	1165	634	717	484	-671
2538	PFD0350w	conserved Plasmodium protein, unknown function	2.22	0.0299	0.0530	0.0139	1.1	7450	962	6879	967	-1357
1454	PF11_0007	erythrocyte membrane protein 1, PfEMP1	3.46	0.0020	0.0055	0.0018	38.4	1154	1894	30	59	-829
2342	PF14_0073	conserved Plasmodium protein, unknown function	2.43	0.0100	0.0208	0.0055	1.6	1122	909	689	423	-899
2542	PFC0205c	1-cys-glutaredoxin-like protein-1	2.21	0.0519	0.0851	0.0223	1.4	1305	489	947	680	-810
2545	PFB0935w	cytoadherence linked asexual protein 2	2.21	0.0539	0.0879	0.0231	1.5	1862	1113	1219	1079	-1548
2446	MAL8P1.97	hypothetical protein	2.31	0.0240	0.0438	0.0115	1.8	1093	956	611	628	-1103
2548	MAL7P1.187	erythrocyte membrane protein 1, PfEMP1	2.20	0.0579	0.0935	0.0245	1.5	2700	1874	1750	1403	-2328
2549	MAL13P1.83	exportin 1-like protein, putative	2.20	0.0419	0.0710	0.0187	1.0	9404	112	9340	106	-154
2550	PFA0140c	conserved Plasmodium protein, unknown function	2.20	0.0459	0.0767	0.0201	1.3	1366	727	1012	496	-869
2552	PFI0595c	conserved Plasmodium membrane protein, unknown fun	2.20	0.0379	0.0651	0.0171	1.2	5810	742	4979	1742	-1653
503	PF13_0081	conserved Plasmodium protein, unknown function	5.17	0.0020	0.0055	0.0018	3.1	1086	752	346	303	-315
2556	PFL0600w	conserved Plasmodium protein, unknown function	2.20	0.0459	0.0767	0.0201	1.1	6156	720	5602	1075	-1241
2557	MAL13P1.76	TFIIH basal transcription factor subunit	2.20	0.0299	0.0530	0.0139	1.1	6753	779	5947	1673	-1646
1467	MAL7P1.82	conserved Plasmodium membrane protein, unknown fun	3.43	0.0060	0.0136	0.0036	2.0	1083	606	548	571	-641
1718	PF07_0109	conserved Plasmodium protein, unknown function	3.12	0.0040	0.0098	0.0026	1.6	1065	673	647	330	-586
1125	PF14_0118		3.97	0.0020	0.0055	0.0018	7.9	1052	1269	134	384	-735
2568	PFF0285c	DNA repair-like protein, putative	2.19	0.0439	0.0739	0.0194	1.2	4410	1660	3565	1276	-2092
2570	PF11_0361		2.19	0.0339	0.0590	0.0155	1.2	3062	668	2589	898	-1093
2571	PFB0250w	conserved Plasmodium protein, unknown function	2.18	0.0439	0.0739	0.0194	1.3	1892	617	1483	756	-964
2572	MAL7P1.105	conserved Plasmodium protein, unknown function	2.18	0.0439	0.0739	0.0194	1.2	4947	1205	4042	1761	-2061
2574	PF11_0509	ring-infected erythrocyte surface antigen	2.18	0.0439	0.0739	0.0194	1.2	7286	1777	5848	2873	-3212
2575	PFC0135c	exportin 1, putative	2.17	0.0379	0.0651	0.0171	1.0	8295	354	8052	459	-570
2579	PF14_0436		2.17	0.0279	0.0501	0.0132	1.0	8911	189	8756	313	-346
1475	PFB0974c	erythrocyte membrane protein 1 (PfEMP1), truncated, de	3.41	0.0020	0.0055	0.0018	2.9	1050	1096	358	369	-774
2582	PFF1370w	protein kinase PK4	2.17	0.0279	0.0501	0.0132	1.0	8516	201	8294	474	-452
1020	PF10_0369	DNA repair helicase rad25, putative	4.14	0.0020	0.0055	0.0018	2.7	1044	769	383	440	-549
2585	PFA0105w	stevor, pseudogene	2.16	0.0599	0.0963	0.0252	1.5	2142	1622	1423	892	-1795
199	PFL1930w	conserved Plasmodium protein, unknown function	6.29	0.0020	0.0055	0.0018	2.5	1041	477	417	276	-128
2590	PFL2345c	tat-binding protein homolog	2.16	0.0339	0.0590	0.0155	1.1	7171	617	6504	1422	-1372
374	PFE0565w	conserved Plasmodium protein, unknown function	5.52	0.0020	0.0055	0.0018	2.6	1026	595	389	265	-223
2595	PFL0260c	conserved Plasmodium protein, unknown function	2.16	0.0519	0.0851	0.0223	1.3	1867	625	1441	812	-1012
2596	PF11_0266a	conserved Plasmodium protein, unknown function	2.15	0.0499	0.0823	0.0216	1.4	1278	589	935	606	-851
2597	PFD0515w	exosome rRNA processing protein, putative	2.15	0.0279	0.0501	0.0132	1.0	7942	262	7623	692	-635
2598	PF14_0057	RNA binding protein, putative	2.15	0.0359	0.0621	0.0163	1.2	4499	1237	3622	1706	-2066
834	PF07_0118	conserved Plasmodium membrane protein, unknown fun	4.46	0.0020	0.0055	0.0018	2.1	1021	628	484	264	-356
2601	PFD0135c	rifin, pseudogene	2.15	0.0279	0.0501	0.0132	1.3	3474	1316	2654	1505	-2002
2602	PF08_0088	conserved Plasmodium protein, unknown function	2.15	0.0319	0.0561	0.0147	1.2	5001	938	4322	1331	-1590
2603	PF11_0289	conserved Plasmodium protein, unknown function	2.15	0.0459	0.0767	0.0201	1.7	1216	1010	733	701	-1228
1573	PFL0030c	erythrocyte membrane protein 1, PfEMP1	3.30	0.0040	0.0098	0.0026	2.1	982	747	473	420	-658
2606	PFF1560c	rifin	2.14	0.0379	0.0651	0.0171	2.0	634	768	313	347	-795
2365	MAL13P1.160	conserved Plasmodium protein, unknown function	2.40	0.0259	0.0469	0.0123	1.4	980	595	677	362	-654
2608	PFB0160w	ERCC1 nucleotide excision repair protein, putative	2.14	0.0379	0.0651	0.0171	1.2	4582	1060	3768	1640	-1886

2215	PF11_0288	conserved Plasmodium protein, unknown function	2.56	0.0259	0.0469	0.0123	1.8	966	821	528	475	-858
621	PF13_0139	conserved Plasmodium protein, unknown function	4.90	0.0020	0.0055	0.0018	2.4	963	572	408	278	-295
2612	PF11_0167	conserved Plasmodium membrane protein, unknown fun	2.13	0.0479	0.0797	0.0209	1.1	7202	1110	6362	1685	-1956
2614	PF14_0634	conserved Plasmodium protein, unknown function	2.13	0.0299	0.0530	0.0139	1.2	4706	744	4064	1336	-1437
1315	MAL13P1.550	conserved Plasmodium protein, unknown function	3.66	0.0020	0.0055	0.0018	2.2	944	592	422	491	-561
2618	PF13_0155	conserved Plasmodium protein, unknown function	2.13	0.0499	0.0823	0.0216	1.3	3955	1014	3096	1787	-1942
352	MAL13P1.461	probable protein, unknown function	5.59	0.0020	0.0055	0.0018	3.5	889	546	253	318	-227
2621	MAL8P1.73	rhoptry neck protein 5, putative	2.12	0.0359	0.0621	0.0163	1.2	4845	907	4137	1444	-1643
2624	PF11_0353	conserved Plasmodium protein, unknown function	2.12	0.0359	0.0621	0.0163	1.0	9084	161	8975	212	-264
2626	PFL1465c	heat shock protein hslv	2.12	0.0539	0.0879	0.0231	1.3	2278	1047	1744	870	-1382
2627	PF11_0131	DNA helicase, putative	2.12	0.0519	0.0851	0.0223	1.5	1084	912	722	341	-891
2628	PF10_0261a	conserved Plasmodium protein, unknown function	2.11	0.0579	0.0935	0.0245	1.2	2704	1052	2171	863	-1381
1984	PFI1430w	conserved Plasmodium protein, unknown function	2.84	0.0120	0.0243	0.0064	1.6	846	616	513	251	-534
2630	PFI0225w	ubiquitin specific protease, putative	2.11	0.0359	0.0621	0.0163	1.0	7553	283	7205	771	-706
2255	PFD0640c	rifin	2.52	0.0240	0.0438	0.0115	4.5	821	1440	183	276	-1078
2632	PF13_0310	small subunit rRNA processing factor, putative	2.11	0.0279	0.0501	0.0132	1.1	7642	437	7260	806	-861
2634	MAL13P1.12	conserved Plasmodium membrane protein, unknown fun	2.11	0.0459	0.0767	0.0201	1.1	6218	696	5545	1450	-1473
2637	PF14_0641	1-deoxy-D-xylulose 5-phosphate reductoisomerase	2.11	0.0499	0.0823	0.0216	1.1	4708	858	4112	1186	-1447
2638	PF11_0114	actin-like protein homolog, ALP1 homolog	2.11	0.0539	0.0879	0.0231	1.1	6473	875	5827	1310	-1539
589	MAL13P1.230	conserved Plasmodium protein, unknown function	4.97	0.0020	0.0055	0.0018	2.3	817	455	350	255	-243
2640	PFE1130w	conserved protein, unknown function	2.10	0.0479	0.0797	0.0209	1.2	4742	697	3861	1966	-1782
2642	PF11_0275	conserved Plasmodium protein, unknown function	2.10	0.0339	0.0590	0.0155	1.0	8246	250	8081	321	-407
494	PF14_0622	potassium channel protein	5.20	0.0020	0.0055	0.0018	3.7	804	571	220	270	-257
1669	MAL13P1.41	conserved Plasmodium protein, unknown function	3.19	0.0040	0.0098	0.0026	1.8	784	497	437	331	-482
2650	PFC0190c	EH (Eps15 homology) protein	2.09	0.0439	0.0739	0.0194	1.0	9318	142	9245	122	-192
2653	PF11_0514	Plasmodium exported protein (PHISTa), unknown functio	2.09	0.0659	0.1047	0.0274	3.0	570	1032	189	214	-865
2171	PF11_0237		2.61	0.0180	0.0344	0.0090	1.7	761	524	439	413	-615
2657	PF14_0564	conserved Plasmodium protein, unknown function	2.08	0.0399	0.0680	0.0179	1.2	4196	1391	3503	1137	-1835
2659	PF14_0637	rhoptry protein, putative	2.08	0.0399	0.0680	0.0179	1.2	4044	879	3367	1412	-1614
2660	PFI1080w	dynein intermediate chain 2, ciliary	2.08	0.0459	0.0767	0.0201	1.2	3986	792	3430	1131	-1366
2662	MAL8P1.81	flavoprotein, putative	2.08	0.0299	0.0530	0.0139	1.0	8889	288	8580	689	-667
2665	MAL7P1.118	PelOta protein homologue, putative	2.07	0.0399	0.0680	0.0179	1.0	7010	331	6685	717	-723
2667	MAL13P1.38	conserved Plasmodium membrane protein, unknown fun	2.07	0.0579	0.0935	0.0245	1.1	6770	1058	6024	1525	-1838
2668	PF14_0521	conserved Plasmodium protein, unknown function	2.07	0.0599	0.0963	0.0252	1.4	1852	1131	1310	865	-1453
2669	PFI1485c	diacylglycerol kinase, putative	2.07	0.0399	0.0680	0.0179	1.1	6692	533	6337	714	-891
2670	PF11_0083	nucleic acid binding protein, putative	2.07	0.0479	0.0797	0.0209	1.2	4417	823	3825	1221	-1453
1726	PF08_0047	conserved Plasmodium protein, unknown function	3.11	0.0100	0.0208	0.0055	4.5	750	965	167	429	-811
2678	PFF0330w	coatomer alpha subunit, putative	2.06	0.0299	0.0530	0.0139	1.0	8648	256	8447	429	-484
928	MAL8P1.201	conserved Plasmodium protein, unknown function	4.31	0.0020	0.0055	0.0018	4.3	658	663	152	145	-302
2687	PF11_0272	40S ribosomal protein S18, putative	2.05	0.0399	0.0680	0.0179	1.0	9574	79	9517	120	-142
2690	PFF1345w	transportin	2.04	0.0559	0.0908	0.0238	1.0	9571	108	9512	108	-158
2691	PF08_0052	perforin like protein 5	2.04	0.0579	0.0935	0.0245	1.3	3880	1533	3068	1462	-2183
2694	PFL0330c	DNA-directed RNA polymerase III subunit, putative	2.04	0.0279	0.0501	0.0132	1.0	8292	266	8003	658	-635

2696	PF10_0337	ADP-ribosylation factor, putative	2.04	0.0539	0.0879	0.0231	1.1	5361	1010	4671	1425	-1745
2697	MAL13P1.242	step II splicing factor, putative	2.04	0.0339	0.0590	0.0155	1.1	7087	529	6656	935	-1034
1267	PFC0120w	cytoadherence linked asexual protein 3.1	3.74	0.0020	0.0055	0.0018	3.8	612	639	161	251	-438
2703	PF11_0154	conserved Plasmodium protein, unknown function	2.03	0.0679	0.1075	0.0282	1.2	3168	861	2683	920	-1296
2705	PFB0760w	MtN3-like protein	2.03	0.0439	0.0739	0.0194	1.0	7580	468	7263	656	-807
2707	PF11_0174	cathepsin C, homolog,dipeptidyl peptidase 1	2.03	0.0379	0.0651	0.0171	1.0	8318	426	7938	846	-892
2710	PF11_0043	60S ribosomal protein P1, putative	2.03	0.0379	0.0651	0.0171	1.0	9905	36	9885	38	-54
2712	PF14_0144	RNA guanylyltransferase	2.02	0.0519	0.0851	0.0223	1.1	7240	572	6745	1097	-1174
2713	MAL13P1.247	conserved Plasmodium protein, unknown function	2.02	0.0599	0.0963	0.0252	1.1	4946	898	4367	1182	-1500
2715	PF14_0016	early transcribed membrane protein 14.1	2.02	0.0439	0.0739	0.0194	1.0	9972	27	9950	49	-54
2716	PFF0395c	conserved protein, unknown function	2.02	0.0499	0.0823	0.0216	1.1	7061	926	6574	888	-1328
2722	PFA0520c	chromatin assembly factor 1 protein WD40 domain, puta	2.02	0.0479	0.0797	0.0209	1.1	5627	1004	4922	1493	-1791
2723	PF07_0102	immunoglobulin-binding protein 1-related, putative	2.02	0.0439	0.0739	0.0194	1.2	3673	826	3139	1098	-1389
2729	PFF0475w	hypothetical protein	2.01	0.0579	0.0935	0.0245	1.2	4576	770	3895	1530	-1619
2732	PF14_0643	conserved Plasmodium protein, unknown function	2.00	0.0419	0.0710	0.0187	1.4	2580	1153	1896	1364	-1833
2733	MAL13P1.49	conserved Plasmodium protein, unknown function	2.00	0.0519	0.0851	0.0223	1.1	6464	839	5690	1761	-1825
2735	PF11_0403	conserved Plasmodium protein, unknown function	2.00	0.0459	0.0767	0.0201	1.0	7070	322	6802	599	-653
2736	PF14_0293	conserved Plasmodium protein, unknown function	2.00	0.0459	0.0767	0.0201	1.1	5661	633	5266	813	-1050
2740	PF14_0466	Appr-1-p processing domain protein	1.99	0.0519	0.0851	0.0223	1.2	3146	885	2591	1143	-1473
2743	PFL0540w	GPI mannosyltransferase I	1.99	0.0619	0.0990	0.0260	1.1	6072	602	5526	1247	-1303
2744	MAL13P1.326	ferrochelatase	1.99	0.0599	0.0963	0.0252	1.2	3499	1030	3017	813	-1362
2745	PF11_0408	conserved Plasmodium protein, unknown function	1.98	0.0499	0.0823	0.0216	1.1	4378	697	3872	1104	-1295
1474	PF14_0112	plastid replication-repair enzyme	3.42	0.0020	0.0055	0.0018	2.4	607	555	250	209	-408
2747	PF08_0049	lsm3 homologue, putative	1.98	0.0339	0.0590	0.0155	1.0	8345	217	8020	780	-673
2748	PF14_0517	aminopeptidase P	1.98	0.0339	0.0590	0.0155	1.0	8477	263	8249	518	-553
2752	PF14_0239	conserved Plasmodium protein, unknown function	1.97	0.0459	0.0767	0.0201	1.1	7070	669	6232	2002	-1833
2753	PFA0695c	erythrocyte membrane protein 1 (PfEMP1), exon2, pseud	1.97	0.1078	0.1604	0.0420	1.7	640	655	367	394	-776
2755	PFF0455w	citrate synthase-like protein, putative	1.97	0.0459	0.0767	0.0201	1.1	4786	867	4259	1090	-1429
2756	PFL1065c	conserved Plasmodium protein, unknown function	1.97	0.0778	0.1214	0.0318	1.1	6067	549	5681	843	-1005
2759	PFD1015c	erythrocyte membrane protein 1, PfEMP1	1.97	0.0619	0.0990	0.0260	1.5	1112	863	741	571	-1063
2761	MAL7P1.137	kelch protein, putative	1.97	0.0699	0.1101	0.0289	1.1	4557	927	4008	1122	-1501
2762	PFE1105c	conserved Plasmodium protein, unknown function	1.97	0.1018	0.1525	0.0400	1.8	456	415	251	371	-582
2765	MAL13P1.91	conserved Plasmodium protein, unknown function	1.96	0.0439	0.0739	0.0194	1.2	2626	897	2111	1041	-1423
1147	PFI1055w	conserved Plasmodium protein, unknown function	3.93	0.0020	0.0055	0.0018	2.3	594	470	257	143	-276
2768	PFE0180w		1.96	0.0459	0.0767	0.0201	1.1	4925	849	4369	1193	-1485
2769	PFL0320w	rhodanese like protein, putative	1.96	0.0619	0.0990	0.0260	1.1	6244	859	5435	1888	-1938
2770	PFA0505c	DNA-directed RNA polymerase 2, putative	1.96	0.0379	0.0651	0.0171	1.0	9032	243	8802	536	-550
2771	MAL7P1.64	serpentine receptor, putative	1.96	0.0379	0.0651	0.0171	1.0	8830	292	8565	617	-643
2772	PF10_0111	20S proteasome beta subunit, putative	1.96	0.0459	0.0767	0.0201	1.1	6619	706	6106	1138	-1331
2774	PFL1660c	conserved Plasmodium protein, unknown function	1.96	0.0599	0.0963	0.0252	1.3	2186	1139	1681	827	-1461
2777	PFE1550w	conserved Plasmodium protein, unknown function	1.96	0.0659	0.1047	0.0274	1.4	1597	793	1175	819	-1190
2779	PFL0005w	erythrocyte membrane protein 1, PfEMP1	1.96	0.0958	0.1448	0.0380	1.4	2048	1444	1467	803	-1666
2781	PF10_0242	conserved Plasmodium protein, unknown function	1.95	0.0499	0.0823	0.0216	1.0	9939	27	9914	57	-60

1469	PFF1340w	conserved Plasmodium protein, unknown function	3.42	0.0020	0.0055	0.0018	3.3	584	654	174	202	-447
2783	PFF0295c	conserved Plasmodium protein, unknown function	1.95	0.0699	0.1101	0.0289	1.0	9535	156	9442	193	-256
2785	PFB0970c	Plasmodium exported protein, unknown function	1.95	0.0858	0.1318	0.0346	1.9	1141	1283	595	854	-1590
2789	MAL13P1.123		1.94	0.0739	0.1156	0.0303	1.2	3205	899	2643	1197	-1534
2791	PFI1270w	conserved Plasmodium protein, unknown function	1.94	0.0559	0.0908	0.0238	1.0	9939	47	9911	58	-77
2793	PF14_0715	conserved Plasmodium protein, unknown function	1.93	0.0479	0.0797	0.0209	1.2	2850	801	2356	1054	-1362
2796	PFL2010c	DEAD/DEAH box ATP-dependent RNA helicase, putative	1.93	0.0559	0.0908	0.0238	1.0	8974	241	8792	416	-475
2798	PF14_0264	protein kinase, putative	1.92	0.0499	0.0823	0.0216	1.2	4665	1196	4056	1182	-1768
2799	PFE1060c	conserved Plasmodium protein, unknown function	1.92	0.0699	0.1101	0.0289	1.3	1619	634	1259	748	-1021
2800	PFL0623c		1.92	0.0559	0.0908	0.0238	1.1	4442	836	3901	1187	-1482
2801	PFE1030c	phosphomethylpyrimidine kinase, putative	1.92	0.0699	0.1101	0.0289	1.4	1606	976	1169	756	-1295
2802	PF14_0100	cytidine triphosphate synthetase	1.92	0.0439	0.0739	0.0194	1.0	8594	227	8376	522	-531
2805	PFC0221c	conserved Plasmodium protein, unknown function	1.92	0.0699	0.1101	0.0289	1.5	1749	1140	1187	1070	-1648
2806	PFF0945c	acyl-CoA synthetase, PfACS12	1.92	0.0359	0.0621	0.0163	1.2	4604	1251	3813	1726	-2187
2810	PF14_0604a	conserved Plasmodium protein, unknown function	1.91	0.0639	0.1019	0.0267	1.2	1850	611	1528	648	-936
2814	PF14_0615	ATP synthase (C/AC39) subunit, putative	1.91	0.0679	0.1075	0.0282	1.6	1195	938	767	763	-1273
2815	PFF1030w	mRNA binding Pumilio-homology domain protein, putativ	1.91	0.0539	0.0879	0.0231	1.0	8919	175	8799	269	-324
2816	PFA0110w	ring-infected erythrocyte surface antigen	1.91	0.0399	0.0680	0.0179	1.1	9488	768	8648	2060	-1987
606	PF10_0083	zinc finger C-x8-C-x5-C-x3-H type, putative	4.94	0.0020	0.0055	0.0018	2.5	582	372	235	143	-168
2317	MAL7P1.160		2.46	0.0240	0.0438	0.0115	1.9	569	606	295	195	-528
2819	PF14_0388	conserved Plasmodium protein, unknown function	1.90	0.0958	0.1448	0.0380	1.3	2084	881	1649	840	-1286
2820	MAL8P1.128	proteasome subunit alpha, putative	1.90	0.0479	0.0797	0.0209	1.0	8174	355	7924	571	-676
2823	PF11_0206	conserved Plasmodium protein, unknown function	1.90	0.0479	0.0797	0.0209	1.0	7599	383	7283	750	-817
2825	PF07_0033	Cg4 protein	1.90	0.0619	0.0990	0.0260	1.0	9805	49	9776	62	-82
2826	PF13_0232	casein kinase II beta chain	1.90	0.0519	0.0851	0.0223	1.0	8775	245	8587	442	-498
2828	PFC0380w	dual specificity phosphatase	1.90	0.0559	0.0908	0.0238	1.0	8493	298	8216	673	-693
2829	PF11_0065	40S ribosomal protein S4, putative	1.89	0.0459	0.0767	0.0201	1.0	9869	31	9846	51	-60
2830	PFI1400c	conserved Plasmodium protein, unknown function	1.89	0.0459	0.0767	0.0201	1.2	4540	933	3870	1543	-1807
2831	PF13_0026	transcription factor with AP2 domain(s), putative	1.89	0.0599	0.0963	0.0252	1.2	3056	945	2619	804	-1312
2832	PFB0465c	monocarboxylate transporter, putative	1.89	0.0699	0.1101	0.0289	1.1	6909	944	6267	1462	-1763
2833	PFI0615w	conserved Plasmodium protein, unknown function	1.89	0.0918	0.1398	0.0367	1.4	1696	1194	1203	792	-1493
2834	PFI1090w	S-adenosylmethionine synthetase	1.89	0.0399	0.0680	0.0179	1.0	9859	47	9822	87	-97
2836	PFC1065w	conserved Plasmodium protein, unknown function	1.89	0.0559	0.0908	0.0238	1.1	6951	483	6440	1262	-1234
2837	PFD1165w	serine/threonine protein kinase, FIKK family	1.89	0.0599	0.0963	0.0252	1.0	8489	461	8214	600	-786
2838	PF11_0454	40S ribosomal protein S21e, putative	1.89	0.0419	0.0710	0.0187	1.0	9496	119	9406	211	-240
2842	MAL13P1.166	DEAD box helicase, putative	1.88	0.0679	0.1075	0.0282	1.1	7533	514	7163	862	-1006
2843	PF14_0721	cytochrome c oxidase assembly protein, putative	1.88	0.0719	0.1129	0.0296	1.2	3792	1418	3047	1533	-2205
2844	PF14_0241	basic transcription factor 3b, putative	1.88	0.0419	0.0710	0.0187	1.0	8741	238	8521	539	-556
2847	PF07_0121	60S ribosomal subunit export protein, putative	1.88	0.0858	0.1318	0.0346	1.0	9259	174	9162	208	-285
2848	PF07_0086	conserved Plasmodium membrane protein, unknown fun	1.87	0.0679	0.1075	0.0282	1.2	4552	1248	3789	1697	-2182
2849	PF10_0035	conserved Plasmodium protein, unknown function	1.87	0.0739	0.1156	0.0303	1.3	1613	850	1236	680	-1153
2854	PFI0060c	hypothetical protein, pseudogene	1.86	0.0838	0.1294	0.0339	2.6	445	701	170	419	-845
2855	PFI0240c	Cu2 -transporting ATPase, putative	1.86	0.0739	0.1156	0.0303	1.1	5023	891	4462	1268	-1599

2858	PFA0175w	conserved Plasmodium protein, unknown function	1.86	0.0739	0.1156	0.0303	1.1	3543	583	3140	940	-1121
2860	MAL8P1.9	u6 snRNA-associated Sm-like protein, putative	1.86	0.0539	0.0879	0.0231	1.1	7456	815	7011	951	-1322
2861	PF13_0330	ATP-dependent DNA helicase, putative	1.86	0.0619	0.0990	0.0260	1.1	6551	826	6056	1106	-1437
2865	PFF0370w	para-hydroxybenzoate--polyprenyltransferase (4- hydroxy	1.85	0.0539	0.0879	0.0231	1.1	5212	802	4768	961	-1319
2867	PF10_0038	40S ribosomal protein S20e, putative	1.85	0.0419	0.0710	0.0187	1.0	9185	223	8978	512	-529
2868	PF14_0757	Plasmodium exported protein (PHISTa), unknown functio	1.85	0.0699	0.1101	0.0289	1.2	3875	1131	3279	1258	-1793
2870	PFE0605c	glutathione synthetase	1.85	0.0958	0.1448	0.0380	1.2	5452	703	4655	2028	-1933
2266	MAL8P1.140	methionine aminopeptidase 1c, putative	2.51	0.0080	0.0173	0.0046	2.1	542	639	259	133	-489
2873	PFD0715c	conserved Plasmodium protein, unknown function	1.85	0.0998	0.1499	0.0393	1.5	844	567	582	507	-811
2876	PFB0655c	conserved Plasmodium protein, unknown function	1.84	0.0339	0.0590	0.0155	1.0	8428	235	8182	624	-612
2878	PF13_0238	kelch protein, putative	1.84	0.0699	0.1101	0.0289	1.0	9546	177	9414	319	-364
2879	MAL7P1.167	conserved Plasmodium protein, unknown function	1.84	0.0838	0.1294	0.0339	1.3	2459	1073	1920	1112	-1646
2880	PF14_0269	conserved protein, unknown function	1.84	0.0818	0.1267	0.0332	1.3	1472	840	1118	622	-1109
2881	PF13_0196	MSP7-like protein	1.84	0.0579	0.0935	0.0245	1.2	3540	1021	3002	1146	-1628
2882	PFC0785c	proteasome regulatory protein, putative	1.84	0.0619	0.0990	0.0260	1.1	7979	910	7470	1119	-1520
2885	PFE0410w	triose phosphate transporter	1.83	0.0739	0.1156	0.0303	1.1	5097	849	4592	1145	-1488
2886	PF11_0429	conserved Plasmodium protein, unknown function	1.83	0.0778	0.1214	0.0318	1.1	5463	810	4859	1463	-1669
2887	PFC0735w	40S ribosomal protein S15A, putative	1.83	0.0679	0.1075	0.0282	1.0	9776	46	9745	75	-90
2889	PF14_0019	hypothetical protein	1.83	0.0699	0.1101	0.0289	1.8	399	499	223	211	-535
2890	PF14_0626	dynein beta chain, putative	1.82	0.0818	0.1267	0.0332	1.1	2391	706	2100	509	-924
2891	PFF0900c	rhomboid protease ROM10	1.82	0.0838	0.1294	0.0339	1.1	6029	830	5404	1530	-1735
2892	PFF0625w	nucleolar GTP-binding protein 1, putative	1.82	0.0679	0.1075	0.0282	1.0	9262	165	9128	334	-365
2895	PFI0355c	ATP-dependent heat shock protein, putative	1.82	0.0798	0.1240	0.0325	1.2	3961	1252	3289	1476	-2056
2896	PF08_0115	DnaJ protein, putative	1.82	0.0659	0.1047	0.0274	1.0	7948	241	7769	440	-501
2897	PF10_0223	conserved Plasmodium membrane protein, unknown fun	1.81	0.0818	0.1267	0.0332	1.1	5923	617	5395	1328	-1417
2898	PF10_0335	conserved Plasmodium protein, unknown function	1.81	0.0858	0.1318	0.0346	1.1	7080	953	6445	1518	-1836
2901	PFI0395w	conserved Plasmodium protein, unknown function	1.81	0.0818	0.1267	0.0332	1.2	2584	566	2229	833	-1045
2902	PFF1410c	nicotinate phosphoribosyltransferase, putative	1.81	0.0619	0.0990	0.0260	1.0	8910	756	8594	579	-1018
2904	PF10_0279	conserved Plasmodium protein, unknown function	1.80	0.0619	0.0990	0.0260	1.3	2107	1104	1602	1010	-1609
2905	PF14_0023	conserved Plasmodium protein, unknown function	1.80	0.0599	0.0963	0.0252	1.2	3233	746	2735	1202	-1450
2909	PFE0855c	conserved Plasmodium protein, unknown function	1.80	0.1257	0.1825	0.0479	2.0	950	1389	470	594	-1503
2910	PFE1190c	conserved Plasmodium membrane protein, unknown fun	1.80	0.0459	0.0767	0.0201	1.1	7798	444	7407	998	-1051
2913	PFL2220w	conserved Plasmodium protein, unknown function	1.79	0.0818	0.1267	0.0332	1.0	7891	297	7619	700	-725
2917	PFI0455w	exoribonuclease, putative	1.79	0.0938	0.1424	0.0373	1.0	9185	154	9110	160	-238
2920	PFB0900c	Plasmodium exported protein (PHISTc), unknown functio	1.79	0.0639	0.1019	0.0267	1.0	9489	686	9122	823	-1143
2921	MAL8P1.54	conserved Plasmodium protein, unknown function	1.79	0.0918	0.1398	0.0367	1.1	6374	555	6062	718	-961
2922	PF14_0207	DNA-directed RNA polymerase III subunit C, putative	1.79	0.0599	0.0963	0.0252	1.1	6248	714	5937	605	-1009
2923	PF14_0251	null	1.79	0.0878	0.1345	0.0353	1.1	6052	1180	5471	1250	-1849
2925	MAL13P1.330	conserved Plasmodium protein, unknown function	1.78	0.0758	0.1186	0.0311	1.0	6400	398	6157	578	-732
2928	PF14_0143	atypical protein kinase, ABC-1 family, putative	1.78	0.0818	0.1267	0.0332	1.3	3332	1506	2665	1330	-2169
2932	MAL13P1.234	conserved Plasmodium protein, unknown function	1.78	0.0778	0.1214	0.0318	1.1	5713	1119	5058	1541	-2005
2933	PF08_0055	U3 small nucleolar ribonucleoprotein protein, putative	1.77	0.0699	0.1101	0.0289	1.0	8392	306	8155	603	-672
2935	PFD0590c	DNA polymerase alpha	1.77	0.0858	0.1318	0.0346	1.2	2620	780	2167	1066	-1393

2936	PFE0900w	conserved protein, unknown function	1.77	0.0719	0.1129	0.0296	1.1	6621	342	6282	891	-895
2937	PFF1435w	conserved Plasmodium protein, unknown function	1.77	0.0459	0.0767	0.0201	1.0	8323	206	8046	748	-676
2938	MAL8P1.94		1.77	0.0858	0.1318	0.0346	1.1	7170	599	6644	1369	-1443
2939	PF10_0235	RNA binding protein, putative	1.76	0.1118	0.1653	0.0434	1.3	1072	423	856	480	-688
2941	PFI1445w	high molecular weight rhoptry protein 2	1.76	0.0858	0.1318	0.0346	1.1	6686	1208	5869	2030	-2421
2942	MAL13P1.338	U1 small nuclear ribonucleoprotein, putative	1.76	0.0818	0.1267	0.0332	1.1	6085	586	5734	842	-1077
2943	PF11_0044	iron-sulfur assembly protein, sufD, putative	1.76	0.0978	0.1474	0.0387	1.3	1631	785	1296	658	-1108
2945	PFL0985c	conserved protein, unknown function	1.76	0.0599	0.0963	0.0252	1.1	6812	528	6462	867	-1045
2947	PFD0885c	conserved Plasmodium protein, unknown function	1.76	0.0798	0.1240	0.0325	1.1	8038	618	7518	1352	-1450
2951	PF14_0411	small nuclear ribonucleoprotein, putative	1.75	0.0639	0.1019	0.0267	1.0	8850	426	8524	837	-937
2952	MAL7P1.6	Plasmodium exported protein (hyp12), unknown functior	1.75	0.0778	0.1214	0.0318	1.0	9524	727	9191	703	-1097
2954	PF14_0307	conserved protein, unknown function	1.75	0.0539	0.0879	0.0231	1.1	7786	342	7395	1056	-1006
2956	PFC0390w	N2227-like protein, putative	1.75	0.0719	0.1129	0.0296	1.1	7593	534	7207	983	-1131
692	PFF1475c	conserved Plasmodium protein, unknown function	4.74	0.0020	0.0055	0.0018	2.9	504	375	173	132	-176
2960	MAL7P1.57	rifin	1.74	0.1277	0.1848	0.0484	2.3	822	1407	359	556	-1499
2961	PFI0235w	replication factor A-related protein, putative	1.74	0.1198	0.1751	0.0459	1.2	3633	985	3129	1158	-1639
2962	PF14_0077	plasmepsin II	1.74	0.0679	0.1075	0.0282	1.1	6671	627	6027	1739	-1722
2963	PFD1045c	erythrocyte membrane-associated antigen, putative	1.74	0.1158	0.1704	0.0447	1.5	1496	1098	1005	1035	-1641
2969	MAL13P1.243	elongation factor Tu, putative	1.73	0.1078	0.1604	0.0420	1.3	2034	1018	1615	820	-1419
2970	MAL13P1.298	conserved Plasmodium membrane protein, unknown fun	1.73	0.0719	0.1129	0.0296	1.1	5222	722	4783	1087	-1370
2972	PF08_0128	SYF2 splicing factor, putative	1.73	0.0918	0.1398	0.0367	1.0	8487	232	8339	370	-454
2973	PF10_0289	adenosine deaminase, putative	1.73	0.0938	0.1424	0.0373	1.0	9622	156	9534	215	-282
2974	PFL1255c	conserved Plasmodium protein, unknown function	1.72	0.0978	0.1474	0.0387	1.1	4639	740	4117	1343	-1563
2978	MAL7P1.27	chloroquine resistance transporter	1.72	0.0699	0.1101	0.0289	1.0	8260	465	7982	689	-876
2979	PFF0880c	conserved Plasmodium protein, unknown function	1.72	0.0499	0.0823	0.0216	1.0	8831	421	8412	1138	-1140
2981	PFD0150w	conserved Plasmodium protein, unknown function	1.72	0.0519	0.0851	0.0223	1.1	7823	577	7424	1029	-1207
2982	PFL0885w	adaptor protein subunit, putative	1.72	0.1178	0.1729	0.0454	1.0	7660	484	7394	640	-857
2984	PFA0415c	mitochondrial carrier protein, putative	1.72	0.0978	0.1474	0.0387	1.1	6658	1170	5960	1729	-2201
2985	PF07_0031	heat shock protein 86 family protein	1.71	0.1118	0.1653	0.0434	1.2	4756	1001	3937	2182	-2364
2989	PF11_0033	probable protein, unknown function	1.71	0.0938	0.1424	0.0373	1.1	8441	871	7791	1709	-1930
2990	PFC0535w	60S ribosomal protein L26, putative	1.71	0.0958	0.1448	0.0380	1.0	9700	150	9642	108	-199
2993	PFI0830c	conserved Plasmodium protein, unknown function	1.71	0.0958	0.1448	0.0380	1.3	2557	873	2016	1366	-1699
2994	PFE0570w	RNA pseudouridylate synthase, putative	1.71	0.0818	0.1267	0.0332	1.0	8803	345	8539	703	-783
2995	PF13_0279	transcription initiation factor TFIIH, putative	1.71	0.0878	0.1345	0.0353	1.1	4301	744	3798	1302	-1543
2997	PFF0765c	conserved Plasmodium protein, unknown function	1.70	0.1178	0.1729	0.0454	1.3	1877	735	1476	976	-1310
1737	PF10_0007	Plasmodium exported protein, unknown function, pseud	3.11	0.0020	0.0055	0.0018	2.4	404	415	168	130	-309
