# Supplementary material for: Transcriptional Profiling of Plasmodium falciparum Parasites from Patients with Severe Malaria Identifies Distinct Low vs. High Parasitemic Clusters
Source: PLoS One. 2012 Jul 18;7(7):e40739. doi: 10.1371/journal.pone.0040739 (PMC3399889; doi:10.1371/journal.pone.0040739)
Supplement: Table S2 — The peripheral blood parasitemias for all patients in the study by final cluster designation including both the log parasitemia and raw parasitemia (p/uL). (PDF) [file pone.0040739.s007.pdf]

| Malawi Patient Sample | Parasitaemia log10 scale | Parasitaemia natural scale | Malawi Cluster |
|---|---|---|---|
| Malawi2_86428 | 2.683 | 482 | A |
| Malawi2_86424 | 3.324 | 2107 | A |
| Malawi2_86508 | 3.542 | 3481 | A |
| Malawi2_86486 | 3.542 | 3484 | A |
| Malawi2_86436 | 3.905 | 8039 | A |
| Malawi2_86532 | 4.054 | 11320 | A |
| Malawi2_86448 | 4.102 | 12660 | A |
| Malawi2_86604 | 4.104 | 12720 | A |
| Malawi1_63072 | 4.122 | 13240 | A |
| Malawi2_86460 | 4.231 | 17040 | A |
| Malawi2_86594 | 4.246 | 17640 | A |
| Malawi2_86602 | 4.419 | 26240 | A |
| Malawi1_63100 | 4.504 | 31900 | A |
| Malawi2_86490 | 4.529 | 33778 | A |
| Malawi2_86568 | 4.569 | 37100 | A |
| Malawi2_86584 | 4.594 | 39300 | A |
| Malawi2_86488 | 4.595 | 39360 | A |
| Malawi2_86430 | 4.634 | 43095 | A |
| Malawi2_86560 | 4.699 | 49980 | A |
| Malawi2_86552 | 4.732 | 54000 | A |
| Malawi2_86476 | 4.847 | 70380 | A |
| Malawi2_86550 | 4.856 | 71800 | A |
| Malawi2_86576 | 4.859 | 72320 | A |
| Malawi2_86598 | 5.729 | 535620 | A |
| Malawi2_86544 | 4.498 | 31500 | B |
| Malawi2_86596 | 4.538 | 34500 | B |
| Malawi2_86462 | 4.577 | 37800 | B |
| Malawi2_86600 | 4.726 | 53227 | B |
| Malawi1_63012 | 4.742 | 55260 | B |
| Malawi2_86528 | 4.786 | 61120 | B |
| Malawi2_86440 | 4.993 | 98400 | B |
| Malawi2_86432 | 5.005 | 101176 | B |
| Malawi1_63114 | 5.059 | 114660 | B |
| Malawi2_86586 | 5.078 | 119600 | B |
| Malawi2_86452 | 5.138 | 137340 | B |
| Malawi2_86530 | 5.297 | 198000 | B |
| Malawi2_86534 | 5.320 | 209160 | B |
| Malawi2_86484 | 5.335 | 216494 | B |
| Malawi1_63074 | 5.428 | 267860 | B |
| Malawi2_86578 | 5.461 | 289380 | B |
| Malawi1_63020 | 5.513 | 326040 | B |
| Malawi2_86558 | 5.527 | 336160 | B |
| Malawi2_86556 | 5.528 | 337111 | B |
| Malawi2_86458 | 5.558 | 361380 | B |

| | | | |
|---|---|---|---|
| Malawi1_63078 | 5.571 | 372020 | B |
| Malawi2_86536 | 5.602 | 400400 | B |
| Malawi2_86480 | 5.607 | 404600 | B |
| Malawi2_86538 | 5.619 | 416000 | B |
| Malawi2_86500 | 5.723 | 528310 | B |
| Malawi2_86464 | 5.746 | 556920 | B |
| Malawi2_86426 | 5.805 | 638880 | B |
| Malawi1_63118 | 5.807 | 641920 | B |
| Malawi2_86492 | 5.858 | 721680 | B |
| Malawi1_63070 | 5.899 | 792300 | B |
| Malawi2_86612 | 5.899 | 792300 | B |
| Malawi1_63132 | 5.941 | 872920 | B |
| Malawi1_63040 | 6.145 | 1396200 | B |
| Malawi2_86446 | 6.145 | 1396200 | B |
