# Supplementary material for: Transcriptional Profiling of Plasmodium falciparum Parasites from Patients with Severe Malaria Identifies Distinct Low vs. High Parasitemic Clusters
Source: PLoS One. 2012 Jul 18;7(7):e40739. doi: 10.1371/journal.pone.0040739 (PMC3399889; doi:10.1371/journal.pone.0040739)
Supplement: Table S3 — The differential expression of genes by Cluster B for those that were not covered by yeast projections versus those that were covered by yeast projections. The distribution of the mean - standard deviation was computed for expression values of genes whose differential expression for non-yeast vs. yeast [yellow and white genes) and yeast vs. non-yeast [green and gray genes] was significant (FDR ≤0.05). “Highly induced” was defined by a positive value for [(mean of high) - (std of high)] - [(mean of low) - (std of low)] which represents a non-overlapping expression range. Genes shown in yellow are highly induced in non-yeast space relative to yeast space. Genes shown in gray are induced in non-yeast relative to yeast space. Genes shown in green are highly induced in non-yeast space relative to yeast space. Genes shown in white are induced in non-yeast relative to yeast space. (PDF) [file pone.0040739.s008.pdf]

Supplemental Table 3: The differential expression of genes by Cluster B for those that were not covered by yeast projections versus those that were covered by yeast projections. The distribution of the mean - standard deviation was computed for expression values of genes whose differential expression for non-yeast vs. yeast [yellow and white genes) and yeast vs. non-yeast [green and gray genes] was significant (FDR ≤ 0.05). "Highly induced" was defined by a positive value for [(mean of high) - (std of high)] - [(mean of low) - (std of low)] which represents a non-overlapping expression range. Genes shown in yellow are highly induced in non-yeast space relative to yeast space. Genes shown in gray are induced in non-yeast relative to yeast space. Genes shown in green are highly induced in non-yeast space relative to yeast space. Genes shown in white are induced in non-yeast relative to yeast space.

Rank	Feature	Description	Score	Feature P	Feature P Low	Feature P High	FDR(BH)	Q Value	Fold Change	Cluster B, no yeast, mean	Cluster B, no yeast, std	Cluster B, yeast, mean	Cluster B, yeast, std	Diff (MH-SH)-(ML+SL)
Genes highly induced in non-yeast vs. yeast covered space														
1	PF14_0215	ubiquitin ligase, putative	1.39	0.0020	0.0000	0.0030	0.0145	0.0080	1.8	4011	519	2287	720	486
12	PF14_0305	leucine-rich repeat protein	1.15	0.0020	0.0000	0.0030	0.0145	0.0080	1.5	4391	760	2926	510	195
17	PFF1035w	Pf77 protein	1.10	0.0020	0.0000	0.0030	0.0145	0.0080	1.7	4999	660	3020	1138	180
32	PF14_0119	p1/s1 nuclease, putative	1.05	0.0020	0.0000	0.0030	0.0145	0.0080	1.7	4519	631	2705	1096	87
23	MAL13P1.75	conserved Plasmodium protein, unknown function	1.08	0.0020	0.0000	0.0030	0.0145	0.0080	2.3	1603	530	689	315	70
33	PF13_0150	DNA-directed RNA polymerase 3 largest subunit	1.05	0.0020	0.0000	0.0030	0.0145	0.0080	1.1	8101	201	7576	300	25
13	PFE0865c	splicing factor, putative	1.13	0.0020	0.0000	0.0030	0.0145	0.0080	1.0	9865	42	9731	76	16
14	PFC0920w	histone H2A variant, putative	1.12	0.0020	0.0000	0.0030	0.0145	0.0080	1.0	9704	47	9578	65	14
38	PFE1530c	XAP-5 DNA binding protein, putative	1.02	0.0020	0.0000	0.0030	0.0145	0.0080	1.1	8367	193	7893	272	9
Genes induced in non-yeast vs. yeast covered space														
44	PF10_0115	QF122 antigen	1.00	0.0020	0.0000	0.0030	0.0145	0.0080	1.0	9943	15	9894	34	0
715	PFE0065w	skeleton-binding protein 1	0.58	0.0040	0.0003	0.0064	0.0219	0.0093	1.0	9989	2	9986	3	-2
348	PF08_0054	heat shock protein 70	0.71	0.0020	0.0000	0.0030	0.0145	0.0080	1.0	9982	4	9973	9	-4
386	PFI1755c	ring-exported protein 3	0.69	0.0020	0.0000	0.0030	0.0145	0.0080	1.0	9977	4	9965	13	-5
53	PFA0420w	conserved Plasmodium protein, unknown function	0.97	0.0020	0.0000	0.0030	0.0145	0.0080	1.0	9907	57	9670	187	-7
300	MAL13P1.23	conserved Plasmodium protein, unknown function	0.73	0.0020	0.0000	0.0030	0.0145	0.0080	1.0	9961	8	9939	21	-8
151	PFB0445c	DEAD box helicase	0.84	0.0020	0.0000	0.0030	0.0145	0.0080	1.0	9885	25	9828	43	-11
47	PF14_0114	GTP binding protein, putative	0.99	0.0020	0.0000	0.0030	0.0145	0.0080	1.3	4450	611	3422	430	-13
51	PFI0415c	ribosomal RNA methyltransferase, putative	0.98	0.0020	0.0000	0.0030	0.0145	0.0080	1.1	7149	378	6389	401	-18
177	PFL1170w	polyadenylate-binding protein, putative	0.82	0.0020	0.0000	0.0030	0.0145	0.0080	1.0	9913	24	9834	73	-18
962	PF11_0224	circumsporozoite-related antigen	0.52	0.0080	0.0024	0.0122	0.0345	0.0142	1.0	9951	17	9930	23	-19
448	PF13_0346	60S ribosomal protein L40/UBI, putative	0.66	0.0020	0.0000	0.0030	0.0145	0.0080	1.0	9876	27	9835	35	-21
116	PF07_0054	histone H2B variant, putative	0.88	0.0020	0.0000	0.0030	0.0145	0.0080	1.0	9677	83	9514	101	-21
1124	MAL7P1.170	Plasmodium exported protein, unknown function	0.48	0.0120	0.0050	0.0175	0.0456	0.0185	1.0	9963	14	9942	28	-22
60	PF10_0083	zinc finger C-x8-C-x5-C-x3-H type, putative	0.96	0.0020	0.0000	0.0030	0.0145	0.0080	2.6	823	317	317	211	-23
897	MAL7P1.76	conserved Plasmodium protein, unknown function	0.54	0.0040	0.0003	0.0064	0.0219	0.0093	1.0	9937	16	9908	38	-25
56	MAL7P1.68	zinc finger protein, putative	0.97	0.0020	0.0000	0.0030	0.0145	0.0080	1.1	7628	310	6872	471	-25
342	PF14_0434	conserved Plasmodium protein, unknown function	0.71	0.0020	0.0000	0.0030	0.0145	0.0080	1.0	9654	41	9588	52	-27
1002	PF07_0029	heat shock protein 90	0.51	0.0080	0.0024	0.0122	0.0345	0.0142	1.0	9949	22	9920	35	-28
149	PF10_0123	GMP synthetase	0.85	0.0020	0.0000	0.0030	0.0145	0.0080	1.0	9447	73	9291	111	-28
328	PFF0865w	histone H3 variant, putative	0.72	0.0020	0.0000	0.0030	0.0145	0.0080	1.0	9912	35	9838	68	-29
65	PFL0175c	conserved Plasmodium protein, unknown function	0.95	0.0020	0.0000	0.0030	0.0145	0.0080	1.1	8049	215	7484	380	-29

139	PFI1175c	RNA binding protein, putative	0.85	0.0020	0.0000	0.0030	0.0145	0.0080	1.0	9568	54	9395	149	-30
589	PFL2325c	conserved protein, unknown function	0.62	0.0020	0.0000	0.0030	0.0145	0.0080	1.0	9880	25	9831	53	-30
1231	PF08_0074	DNA/RNA-binding protein Alba, putative	0.46	0.0120	0.0050	0.0175	0.0456	0.0185	1.0	9948	16	9921	41	-31
620	PF07_0033	Cg4 protein	0.61	0.0060	0.0012	0.0094	0.0286	0.0120	1.0	9829	27	9778	56	-33
826	PFI1270w	conserved Plasmodium protein, unknown function	0.55	0.0040	0.0003	0.0064	0.0219	0.0093	1.0	9960	18	9919	57	-33
205	MAL8P1.69	14-3-3 protein, putative	0.79	0.0020	0.0000	0.0030	0.0145	0.0080	1.0	9842	47	9705	127	-37
80	PF14_0469		0.92	0.0020	0.0000	0.0030	0.0145	0.0080	1.1	9274	143	8822	346	-37
1018	PF08_0004	conserved Plasmodium protein, unknown function	0.51	0.0100	0.0036	0.0149	0.0399	0.0162	1.0	9909	31	9870	45	-38
93	PF08_0016	conserved Plasmodium protein, unknown function	0.90	0.0020	0.0000	0.0030	0.0145	0.0080	1.0	8762	164	8376	262	-41
230	PF13_0163	conserved Plasmodium protein, unknown function	0.77	0.0020	0.0000	0.0030	0.0145	0.0080	1.0	9697	85	9541	117	-46
64	PFL0560c	minichromosome maintenance protein, putative	0.95	0.0020	0.0000	0.0030	0.0145	0.0080	1.2	5717	449	4788	528	-47
129	PFL0035c	acyl-CoA synthetase, PfACS7	0.87	0.0020	0.0000	0.0030	0.0145	0.0080	1.0	9711	85	9400	274	-48
635	PFE0070w	interspersed repeat antigen	0.61	0.0020	0.0000	0.0030	0.0145	0.0080	1.0	9953	23	9877	103	-50
969	PFL2095w	translation initiation factor SUI1, putative	0.52	0.0100	0.0036	0.0149	0.0399	0.0162	1.0	9838	46	9782	62	-52
113	PFB0555c	conserved Plasmodium membrane protein, unknown fun	0.89	0.0020	0.0000	0.0030	0.0145	0.0080	1.1	8428	207	8017	258	-53
125	MAL13P1.80		0.87	0.0020	0.0000	0.0030	0.0145	0.0080	1.0	8731	195	8371	219	-54
1148	PF14_0068	fibrillarin, putative	0.48	0.0080	0.0024	0.0122	0.0345	0.0142	1.0	9703	48	9655	55	-54
800	PFI0580c	falstatin	0.56	0.0100	0.0036	0.0149	0.0399	0.0162	1.0	9861	48	9791	78	-55
1462	PFC0975c	peptidyl-prolyl cis-trans isomerase	0.42	0.0120	0.0050	0.0175	0.0456	0.0185	1.0	9878	34	9837	63	-57
132	PF14_0546	conserved Plasmodium protein, unknown function	0.86	0.0020	0.0000	0.0030	0.0145	0.0080	1.0	9149	197	8793	217	-57
1184	PF11_0245	translation elongation factor EF-1, subunit alpha, putative	0.47	0.0100	0.0036	0.0149	0.0399	0.0162	1.0	9585	52	9533	59	-59
604	PF10_0063	DNA/RNA-binding protein Alba, putative	0.61	0.0020	0.0000	0.0030	0.0145	0.0080	1.0	9713	60	9619	93	-59
717	PFD0950w	ran binding protein 1, putative	0.58	0.0020	0.0000	0.0030	0.0145	0.0080	1.0	9586	60	9501	86	-61
99	PF11_0115	conserved Plasmodium protein, unknown function	0.90	0.0020	0.0000	0.0030	0.0145	0.0080	1.1	7920	247	7388	347	-62
278	PFI0345w	GTPase activator, putative	0.74	0.0020	0.0000	0.0030	0.0145	0.0080	1.0	9293	78	9113	164	-62
473	PFF1345w	transportin	0.65	0.0020	0.0000	0.0030	0.0145	0.0080	1.0	9624	55	9506	126	-62
451	PF13_0138	MSF1-like protein, putative	0.66	0.0020	0.0000	0.0030	0.0145	0.0080	1.0	9526	108	9403	77	-62
590	PF11_0291	conserved Plasmodium protein, unknown function	0.62	0.0020	0.0000	0.0030	0.0145	0.0080	1.0	9468	82	9363	87	-65
228	PF14_0487	conserved Plasmodium protein, unknown function	0.78	0.0020	0.0000	0.0030	0.0145	0.0080	1.0	9715	113	9490	178	-65
868	PFL0185c	nucleosome assembly protein	0.54	0.0020	0.0000	0.0030	0.0145	0.0080	1.0	9847	42	9770	101	-65
668	PF14_0122	nuclear transport factor 2, putative	0.59	0.0020	0.0000	0.0030	0.0145	0.0080	1.0	9852	62	9756	99	-65
185	PF11_0156	serine/threonine protein kinase	0.81	0.0020	0.0000	0.0030	0.0145	0.0080	1.0	8194	186	7905	171	-68
608	PF08_0056	zinc finger protein, putative	0.61	0.0020	0.0000	0.0030	0.0145	0.0080	1.0	9689	76	9580	102	-69
593	PFF0220w	conserved Plasmodium protein, unknown function	0.62	0.0040	0.0003	0.0064	0.0219	0.0093	1.0	9659	67	9547	114	-69
633	PF10_0095	conserved Plasmodium membrane protein, unknown fun	0.61	0.0020	0.0000	0.0030	0.0145	0.0080	1.0	9322	82	9215	95	-70
372	PFL0145c	high mobility group protein	0.69	0.0020	0.0000	0.0030	0.0145	0.0080	1.0	9618	106	9455	130	-72
251	PFA0635c	Plasmodium exported protein (hyp1), unknown function	0.76	0.0020	0.0000	0.0030	0.0145	0.0080	1.0	9797	42	9569	259	-72
1183	PFI1696c	conserved protein, unknown function	0.47	0.0100	0.0036	0.0149	0.0399	0.0162	1.0	9531	64	9466	74	-73
547	PFI1085w	ubiquitin-like protein, putative	0.63	0.0020	0.0000	0.0030	0.0145	0.0080	1.0	9369	79	9243	121	-74
135	MAL7P1.150	cysteine desulfurase, putative	0.86	0.0020	0.0000	0.0030	0.0145	0.0080	1.1	8887	141	8435	385	-74
100	MAL13P1.41	conserved Plasmodium protein, unknown function	0.90	0.0020	0.0000	0.0030	0.0145	0.0080	2.5	1078	475	429	250	-76
1343	PFB0370c	RNA binding protein, putative	0.44	0.0120	0.0050	0.0175	0.0456	0.0185	1.0	9662	50	9602	86	-76
368	PFB0875c	chromatin-binding protein, putative	0.70	0.0020	0.0000	0.0030	0.0145	0.0080	1.0	9496	79	9310	188	-81
207	PFE1445c	conserved Plasmodium protein, unknown function	0.79	0.0020	0.0000	0.0030	0.0145	0.0080	1.0	8600	185	8298	199	-81
761	PFI0930c	nucleosome assembly protein	0.57	0.0040	0.0003	0.0064	0.0219	0.0093	1.0	9710	67	9601	124	-82
74	PFA0125c	erythrocyte binding antigen-181	0.93	0.0020	0.0000	0.0030	0.0145	0.0080	1.6	2949	549	1833	649	-82
531	PF13_0194	probable protein, unknown function	0.64	0.0020	0.0000	0.0030	0.0145	0.0080	1.0	9816	37	9668	195	-84

124	PFF0325c	conserved Plasmodium protein, unknown function	0.87	0.0020	0.0000	0.0030	0.0145	0.0080	1.1	9018	235	8441	426	-84
69	PFE0715w	aspartyl-tRNA synthetase, putative	0.94	0.0020	0.0000	0.0030	0.0145	0.0080	1.5	4262	614	2891	843	-86
206	PFE1215c	cytosolic preribosomal GTP-binding protein, putative	0.79	0.0020	0.0000	0.0030	0.0145	0.0080	1.0	8818	189	8493	224	-87
520	PF10_0327	Myb2 protein	0.64	0.0020	0.0000	0.0030	0.0145	0.0080	1.0	9087	115	8922	142	-92
719	PF10_0232	chromodomain-helicase-DNA-binding protein 1 homolog	0.58	0.0020	0.0000	0.0030	0.0145	0.0080	1.0	9667	82	9538	140	-93
73	PF10_0265	conserved Plasmodium protein, unknown function	0.94	0.0020	0.0000	0.0030	0.0145	0.0080	2.2	2509	973	1158	473	-94
1430	PFE1490c	RING zinc finger protein, putative	0.43	0.0080	0.0024	0.0122	0.0345	0.0142	1.0	9665	42	9596	121	-94
1265	MAL8P1.40	RNA binding protein, putative	0.45	0.0100	0.0036	0.0149	0.0399	0.0162	1.0	9543	87	9465	85	-94
396	PFL1345c	histone S-adenosyl methyltransferase, putative	0.68	0.0060	0.0012	0.0094	0.0286	0.0120	1.0	8857	119	8654	178	-94
1030	MAL13P1.83	exportin 1-like protein, putative	0.50	0.0040	0.0003	0.0064	0.0219	0.0093	1.0	9450	49	9353	144	-96
701	PF14_0655	helicase 45	0.59	0.0060	0.0012	0.0094	0.0286	0.0120	1.0	9644	105	9507	128	-96
148	PF10_0067	conserved Plasmodium protein, unknown function	0.85	0.0020	0.0000	0.0030	0.0145	0.0080	1.1	8258	320	7714	322	-98
140	MAL13P1.23	conserved Plasmodium protein, unknown function	0.85	0.0020	0.0000	0.0030	0.0145	0.0080	2.2	1050	371	483	294	-98
244	PFE1370w	hsp70 interacting protein, putative	0.76	0.0020	0.0000	0.0030	0.0145	0.0080	1.0	9114	115	8798	300	-99
524	PFL2480w	conserved Plasmodium protein, unknown function	0.64	0.0020	0.0000	0.0030	0.0145	0.0080	1.0	9319	126	9138	157	-102
304	PFD0450c	pre-mRNA splicing factor, putative	0.73	0.0020	0.0000	0.0030	0.0145	0.0080	1.0	9454	99	9178	280	-103
646	PFL1490w	atypical protein kinase, RIO family, putative	0.60	0.0060	0.0012	0.0094	0.0286	0.0120	1.0	9574	65	9415	201	-106
1009	PF14_0174	pseudouridine synthase, putative	0.51	0.0020	0.0000	0.0030	0.0145	0.0080	1.0	9278	81	9165	141	-109
553	PFB0490c	conserved Plasmodium protein, unknown function	0.63	0.0020	0.0000	0.0030	0.0145	0.0080	1.0	9338	115	9153	179	-109
122	PFC0120w	cytoadherence linked asexual protein 3.1	0.88	0.0020	0.0000	0.0030	0.0145	0.0080	4.8	986	658	205	233	-111
88	PFL2405c	osmiophilic body protein	0.91	0.0020	0.0000	0.0030	0.0145	0.0080	1.7	2738	727	1569	555	-112
685	PFF0295c	conserved Plasmodium protein, unknown function	0.59	0.0040	0.0003	0.0064	0.0219	0.0093	1.0	9608	107	9446	167	-112
365	PF14_0117	conserved Plasmodium protein, unknown function	0.70	0.0020	0.0000	0.0030	0.0145	0.0080	1.0	9387	99	9126	275	-113
607	PF10_0028		0.61	0.0020	0.0000	0.0030	0.0145	0.0080	1.0	9545	99	9364	196	-114
101	PFE1085w	DEAD/DEAH box ATP-dependent RNA helicase, putative	0.90	0.0020	0.0000	0.0030	0.0145	0.0080	1.2	5838	447	4856	650	-115
705	PF10_0268	merozoite capping protein 1	0.58	0.0020	0.0000	0.0030	0.0145	0.0080	1.0	9777	97	9615	179	-115
107	PFC0990c	conserved Plasmodium protein, unknown function	0.89	0.0020	0.0000	0.0030	0.0145	0.0080	1.7	2252	678	1313	377	-115
823	PFC0825c	cleavage and polyadenylation specifity factor, putative	0.55	0.0020	0.0000	0.0030	0.0145	0.0080	1.0	8960	92	8815	168	-116
412	PFF1110c	coronin binding protein, putative	0.68	0.0020	0.0000	0.0030	0.0145	0.0080	1.0	8712	150	8466	214	-118
421	PFC0970w	conserved Plasmodium membrane protein, unknown fun	0.67	0.0020	0.0000	0.0030	0.0145	0.0080	1.0	9221	100	8979	260	-118
115	PFC1060c	conserved Plasmodium protein, unknown function	0.89	0.0020	0.0000	0.0030	0.0145	0.0080	1.2	6995	457	6074	584	-119
654	MAL13P1.30	conserved Plasmodium protein, unknown function	0.60	0.0040	0.0003	0.0064	0.0219	0.0093	1.0	8462	162	8280	143	-122
729	PFL0060w	Plasmodium exported protein, unknown function	0.58	0.0020	0.0000	0.0030	0.0145	0.0080	1.0	9924	35	9754	259	-124
291	PF14_0372	conserved Plasmodium protein, unknown function	0.74	0.0040	0.0003	0.0064	0.0219	0.0093	1.0	7980	181	7636	288	-124
751	PFD0455w	40S ribosomal processing protein, putative	0.57	0.0020	0.0000	0.0030	0.0145	0.0080	1.0	9478	86	9309	209	-126
221	MAL8P1.122	ubiquitin regulatory protein, putative	0.78	0.0020	0.0000	0.0030	0.0145	0.0080	1.1	8874	251	8423	327	-126
360	MAL8P1.88	conserved Plasmodium protein, unknown function	0.70	0.0020	0.0000	0.0030	0.0145	0.0080	1.0	9697	128	9395	303	-129
217	MAL8P1.114	conserved Plasmodium protein, unknown function	0.78	0.0020	0.0000	0.0030	0.0145	0.0080	1.1	8211	330	7735	279	-133
606	PFF1370w	protein kinase PK4	0.61	0.0040	0.0003	0.0064	0.0219	0.0093	1.0	8615	166	8400	185	-136
90	PF10_0224	dynein heavy chain, putative	0.91	0.0020	0.0000	0.0030	0.0145	0.0080	2.1	2683	856	1267	697	-137
167	PF08_0003	tryptophan/threonine-rich antigen	0.83	0.0020	0.0000	0.0030	0.0145	0.0080	1.1	9168	307	8512	486	-137
263	MAL13P1.12	SET domain protein, putative	0.75	0.0020	0.0000	0.0030	0.0145	0.0080	1.0	9296	142	8876	416	-138
963	MAL8P1.104	CAF1 family ribonuclease, putative	0.52	0.0060	0.0012	0.0094	0.0286	0.0120	1.0	9631	106	9480	185	-139
819	PFA0515w	phosphatidylinositol-4-phosphate-5-kinase	0.56	0.0020	0.0000	0.0030	0.0145	0.0080	1.0	8908	102	8732	215	-141
953	PF10_0100	conserved Plasmodium protein, unknown function	0.52	0.0020	0.0000	0.0030	0.0145	0.0080	1.0	9937	58	9783	237	-141
169	PF10_0161a	Plasmodium exported protein, unknown function	0.83	0.0020	0.0000	0.0030	0.0145	0.0080	1.1	7650	265	6983	543	-141
533	PF14_0753	Plasmodium exported protein (hyp13), unknown functio	0.64	0.0020	0.0000	0.0030	0.0145	0.0080	1.0	9638	72	9389	320	-143

85	PF10_0181	conserved protein, unknown function	0.92	0.0020	0.0000	0.0030	0.0145	0.0080	1.4	5992	795	4405	938	-146
1119	PF11_0458	conserved Plasmodium protein, unknown function	0.48	0.0040	0.0003	0.0064	0.0219	0.0093	1.0	9130	92	8993	192	-147
1025	PFC0965w	conserved Plasmodium protein, unknown function	0.51	0.0100	0.0036	0.0149	0.0399	0.0162	1.0	9371	130	9220	169	-148
102	MAL7P1.25	cytoskeleton associated protein, putative	0.89	0.0020	0.0000	0.0030	0.0145	0.0080	2.6	2026	933	779	462	-148
758	PFF1500c	DEAD/DEAH box ATP-dependent RNA helicase	0.57	0.0040	0.0003	0.0064	0.0219	0.0093	1.0	8944	123	8744	226	-149
137	MAL8P1.149	conserved Plasmodium protein, unknown function	0.86	0.0020	0.0000	0.0030	0.0145	0.0080	2.0	1755	743	868	294	-150
836	PFL2565w	Plasmodium exported protein (PHISTa), unknown functio	0.55	0.0020	0.0000	0.0030	0.0145	0.0080	1.0	9870	45	9684	290	-150
964	PF13_0013	deoxyhypusine hydroxylase	0.52	0.0020	0.0000	0.0030	0.0145	0.0080	1.0	9073	119	8909	195	-151
712	PF14_0423	eukaryotic initiation factor 2alpha kinase 1	0.58	0.0040	0.0003	0.0064	0.0219	0.0093	1.0	9556	109	9345	253	-151
511	PF14_0076	plasmepsin I	0.64	0.0020	0.0000	0.0030	0.0145	0.0080	1.0	9717	169	9445	254	-151
750	PF11_0200	U2 snRNP auxiliary factor, small subunit, putative	0.57	0.0020	0.0000	0.0030	0.0145	0.0080	1.0	9267	161	9061	196	-152
241	PF11_0166	conserved Plasmodium protein, unknown function	0.76	0.0020	0.0000	0.0030	0.0145	0.0080	1.1	9530	197	9034	451	-153
192	PFE1175w	conserved Plasmodium protein, unknown function	0.80	0.0020	0.0000	0.0030	0.0145	0.0080	1.6	1683	380	1048	410	-155
108	PF08_0125	tubulin gamma chain	0.89	0.0020	0.0000	0.0030	0.0145	0.0080	1.3	5837	573	4573	848	-156
985	PF13_0238	kelch protein, putative	0.52	0.0120	0.0050	0.0175	0.0456	0.0185	1.0	9620	128	9453	194	-156
255	PF13_0318	RNA binding protein, putative	0.76	0.0020	0.0000	0.0030	0.0145	0.0080	1.1	8090	263	7604	379	-156
609	PFF0830w	alpha adaptin-like protein, putative	0.61	0.0060	0.0012	0.0094	0.0286	0.0120	1.0	8949	159	8702	244	-156
106	PF11_0087	Rad51 homolog	0.89	0.0020	0.0000	0.0030	0.0145	0.0080	1.2	6545	522	5245	935	-156
1146	MAL13P1.34:	ribosome biogenesis protein MRT4, putative	0.48	0.0100	0.0036	0.0149	0.0399	0.0162	1.0	8690	140	8547	160	-157
212	PFL1035w	conserved Plasmodium protein, unknown function	0.79	0.0020	0.0000	0.0030	0.0145	0.0080	1.8	1298	474	713	270	-159
173	PF07_0118	conserved Plasmodium membrane protein, unknown fun	0.82	0.0020	0.0000	0.0030	0.0145	0.0080	2.2	1343	686	614	202	-159
120	PFE1090w	nucleotide binding protein, putative	0.88	0.0020	0.0000	0.0030	0.0145	0.0080	2.2	2106	593	966	707	-159
231	PF14_0170	NOT family protein, putative	0.77	0.0020	0.0000	0.0030	0.0145	0.0080	1.1	8661	322	8120	380	-161
726	PFA0525w	transcription initiation factor TFIIB, putative	0.58	0.0060	0.0012	0.0094	0.0286	0.0120	1.0	8999	131	8772	260	-164
672	PFE1115c	S-adenosylmethionine-dependent methyltransferase, put	0.59	0.0040	0.0003	0.0064	0.0219	0.0093	1.0	9195	188	8952	220	-166
1045	PF13_0165	conserved Plasmodium protein, unknown function	0.50	0.0040	0.0003	0.0064	0.0219	0.0093	1.0	9348	93	9180	245	-170
890	PF07_0091	cell cycle control protein cwf15, putative	0.54	0.0080	0.0024	0.0122	0.0345	0.0142	1.0	9007	154	8808	215	-170
450	PF10_0293	transcription factor, putative	0.66	0.0020	0.0000	0.0030	0.0145	0.0080	1.0	8816	185	8479	323	-171
839	PF13_0245	conserved Plasmodium protein, unknown function	0.55	0.0020	0.0000	0.0030	0.0145	0.0080	1.0	8462	164	8248	224	-174
158	PF11_0211	alpha/beta hydrolase fold domain containing protein, put	0.83	0.0020	0.0000	0.0030	0.0145	0.0080	1.1	8439	272	7570	773	-175
189	PFL0040c	serine/threonine protein kinase, FIKK family	0.81	0.0020	0.0000	0.0030	0.0145	0.0080	1.1	8671	351	7928	569	-176
236	PF10_0278	nucleolar preribosomal assembly protein, putative	0.77	0.0020	0.0000	0.0030	0.0145	0.0080	1.1	8490	333	7909	425	-178
1106	PF13_0341	DNA-directed RNA polymerase 2, putative	0.48	0.0100	0.0036	0.0149	0.0399	0.0162	1.0	8829	148	8662	197	-178
126	MAL13P1.39(	aminomethyltransferase, putative	0.87	0.0020	0.0000	0.0030	0.0145	0.0080	1.3	4861	664	3690	685	-179
397	PF11_0144	Rpr2, RNAse P, putative	0.68	0.0040	0.0003	0.0064	0.0219	0.0093	1.1	7808	214	7427	347	-179
1145	PFB0295w	adenylosuccinate lyase	0.48	0.0100	0.0036	0.0149	0.0399	0.0162	1.0	9443	106	9279	238	-180
975	MAL13P1.36	PNAS-3 related protein, putative	0.52	0.0060	0.0012	0.0094	0.0286	0.0120	1.0	8686	179	8490	199	-182
740	PFD0745c	nonclathrin coat protein zeta2-cop-related protein, putat	0.58	0.0060	0.0012	0.0094	0.0286	0.0120	1.0	8662	183	8414	247	-182
747	PFL2010c	DEAD/DEAH box ATP-dependent RNA helicase, putative	0.58	0.0060	0.0012	0.0094	0.0286	0.0120	1.0	9085	193	8838	236	-182
261	PFL1340c	conserved Plasmodium protein, unknown function	0.75	0.0020	0.0000	0.0030	0.0145	0.0080	1.1	9280	177	8724	562	-183
276	PF10_0294	RNA helicase, putative	0.74	0.0020	0.0000	0.0030	0.0145	0.0080	1.1	7483	355	6948	364	-184
528	PFI1720w	gametocytogenesis-implicated protein	0.64	0.0020	0.0000	0.0030	0.0145	0.0080	1.0	9908	90	9578	427	-187
493	PFL1220w	conserved Plasmodium protein, unknown function	0.65	0.0020	0.0000	0.0030	0.0145	0.0080	1.0	8997	180	8651	355	-188
749	PFE1250w	acyl-CoA synthetase, PfACS10	0.58	0.0040	0.0003	0.0064	0.0219	0.0093	1.0	9711	124	9456	319	-188
677	PFI1695c	small nuclear ribonucleoprotein (snRNP), putative	0.59	0.0040	0.0003	0.0064	0.0219	0.0093	1.0	9281	172	9007	291	-189
744	PFB0865w	small nuclear ribonucleoprotein, putative	0.58	0.0040	0.0003	0.0064	0.0219	0.0093	1.0	8730	216	8473	229	-189
156	PFI0990c	CS-domain containing protein, conserved in Apicomplexa	0.83	0.0020	0.0000	0.0030	0.0145	0.0080	1.2	5512	509	4553	640	-190

645	PF13_0102	secretory complex protein 63	0.60	0.0060	0.0012	0.0094	0.0286	0.0120	1.0	8956	167	8664	319	-193
435	PF13_0106	conserved protein, unknown function	0.67	0.0020	0.0000	0.0030	0.0145	0.0080	1.0	9322	189	8926	404	-197
225	PF08_0092	histone-arginine methyltransferase, putative	0.78	0.0020	0.0000	0.0030	0.0145	0.0080	1.1	7844	268	7142	633	-198
369	PF13_0147	conserved Plasmodium protein, unknown function	0.70	0.0020	0.0000	0.0030	0.0145	0.0080	1.1	8420	187	7962	472	-201
623	PF10_0179a	PHF5-like protein, putative	0.61	0.0040	0.0003	0.0064	0.0219	0.0093	1.0	8666	218	8352	297	-202
191	PF11_0449	conserved Plasmodium protein, unknown function	0.80	0.0020	0.0000	0.0030	0.0145	0.0080	1.3	4046	536	3213	500	-203
253	PFL0880c	conserved Plasmodium protein, unknown function	0.76	0.0020	0.0000	0.0030	0.0145	0.0080	1.1	8367	407	7729	434	-204
775	PF08_0137	Plasmodium exported protein (PHISTc), unknown functio	0.57	0.0020	0.0000	0.0030	0.0145	0.0080	1.0	9309	112	9038	364	-205
710	PF11_0108	U5 snRNP-associated protein, putative	0.58	0.0040	0.0003	0.0064	0.0219	0.0093	1.0	8699	196	8409	299	-206
572	PFB0640c	sec31p	0.62	0.0040	0.0003	0.0064	0.0219	0.0093	1.0	8470	221	8127	328	-207
295	PF08_0034	histone acetyltransferase GCN5	0.73	0.0020	0.0000	0.0030	0.0145	0.0080	1.1	7023	448	6446	340	-211
275	PF11_0359	coatomer delta subunit, putative	0.75	0.0020	0.0000	0.0030	0.0145	0.0080	1.1	7717	276	7100	552	-211
627	PF13_0253	ethanolamine-phosphate cytidylyltransferase, putative	0.61	0.0040	0.0003	0.0064	0.0219	0.0093	1.0	9220	184	8894	354	-212
95	MAL8P1.91	phospholipase DDHD1, putative	0.90	0.0020	0.0000	0.0030	0.0145	0.0080	2.3	3379	1382	1474	735	-212
216	PFB0250w	conserved Plasmodium protein, unknown function	0.78	0.0020	0.0000	0.0030	0.0145	0.0080	1.5	2248	425	1470	568	-215
433	PF14_0247	conserved Plasmodium protein, unknown function	0.67	0.0020	0.0000	0.0030	0.0145	0.0080	1.1	8258	267	7821	387	-216
1125	PF14_0563	DEAD box helicase, putative	0.48	0.0060	0.0012	0.0094	0.0286	0.0120	1.0	8928	234	8726	185	-217
548	PF14_0365	conserved Plasmodium protein, unknown function	0.63	0.0020	0.0000	0.0030	0.0145	0.0080	1.0	8418	255	8042	343	-221
306	PF14_0094	conserved Plasmodium protein, unknown function	0.73	0.0020	0.0000	0.0030	0.0145	0.0080	1.1	8619	358	8024	458	-221
235	MAL13P1.21	chromosome condensation protein, putative	0.77	0.0020	0.0000	0.0030	0.0145	0.0080	1.6	1954	472	1224	480	-222
661	PFD1070w	eukaryotic initiation factor, putative	0.60	0.0020	0.0000	0.0030	0.0145	0.0080	1.0	8627	200	8300	349	-222
227	PFL2530w	lysophospholipase, putative	0.78	0.0020	0.0000	0.0030	0.0145	0.0080	1.1	8302	513	7523	489	-223
197	MAL13P1.21[illegible]	DNA helicase, putative	0.80	0.0020	0.0000	0.0030	0.0145	0.0080	1.1	7927	242	7042	870	-227
269	PF14_0476	serine/threonine protein kinase, putative	0.75	0.0020	0.0000	0.0030	0.0145	0.0080	1.1	7505	390	6819	525	-229
577	PFF1135w	transcription or splicing factor-like protein, putative	0.62	0.0020	0.0000	0.0030	0.0145	0.0080	1.0	8609	216	8229	395	-231
400	PF11_0231	conserved Plasmodium protein, unknown function	0.68	0.0020	0.0000	0.0030	0.0145	0.0080	1.1	8186	344	7693	381	-232
181	PFL0745c	conserved Plasmodium protein, unknown function	0.81	0.0020	0.0000	0.0030	0.0145	0.0080	1.3	4418	678	3385	591	-236
711	PFF0560c	SWI/SNF-related matrix-associated actin-dependent regu	0.58	0.0040	0.0003	0.0064	0.0219	0.0093	1.0	8339	239	8005	332	-238
968	PF14_0464	SNARE protein, putative	0.52	0.0040	0.0003	0.0064	0.0219	0.0093	1.0	7827	154	7567	346	-240
474	MAL7P1.28	rRNA/tRNA ribonuclease MRP/P subunit, putative	0.65	0.0040	0.0003	0.0064	0.0219	0.0093	1.1	8262	243	7804	458	-242
584	MAL7P1.4	Plasmodium exported protein (hyp4), unknown function	0.62	0.0020	0.0000	0.0030	0.0145	0.0080	1.0	9585	170	9185	476	-246
426	PFE1430c	cyclophilin, putative	0.67	0.0020	0.0000	0.0030	0.0145	0.0080	1.1	7240	304	6737	446	-247
503	PFC0875w	ABC transporter, putative	0.65	0.0020	0.0000	0.0030	0.0145	0.0080	1.1	8450	248	7989	465	-252
1144	PF10_0078	histone deacetylase, putative	0.48	0.0120	0.0050	0.0175	0.0456	0.0185	1.0	7750	225	7520	259	-253
935	PF11_0242	calcium-dependent protein kinase 7	0.53	0.0060	0.0012	0.0094	0.0286	0.0120	1.0	7867	166	7582	375	-256
549	MAL13P1.30[illegible]	SUMO ligase, putative	0.63	0.0040	0.0003	0.0064	0.0219	0.0093	1.1	8727	371	8290	322	-257
825	PF11_0052	syntaxin, Qa-SNARE family	0.55	0.0040	0.0003	0.0064	0.0219	0.0093	1.0	8813	238	8492	342	-258
114	PFD0325w	conserved Plasmodium protein, unknown function	0.89	0.0020	0.0000	0.0030	0.0145	0.0080	2.4	3457	1255	1461	999	-258
322	PFD1150c	reticulocyte binding protein homologue 4	0.72	0.0020	0.0000	0.0030	0.0145	0.0080	1.8	1498	488	822	447	-260
202	MAL13P1.33[illegible]	conserved Plasmodium protein, unknown function	0.79	0.0020	0.0000	0.0030	0.0145	0.0080	1.7	2449	738	1464	507	-261
413	PFL0885w	adaptor protein subunit, putative	0.68	0.0040	0.0003	0.0064	0.0219	0.0093	1.1	7919	337	7366	480	-265
846	PFI1425w	conserved Plasmodium protein, unknown function	0.55	0.0080	0.0024	0.0122	0.0345	0.0142	1.0	8414	243	8092	344	-265
1014	PFD0505c	protein phosphatase, putative	0.51	0.0100	0.0036	0.0149	0.0399	0.0162	1.0	8977	198	8703	343	-266
319	PFI0695c	phospholipid or glycerol acyltransferase, putative	0.72	0.0020	0.0000	0.0030	0.0145	0.0080	1.1	7761	334	7064	631	-267
482	PFI1115c	pre-mRNA splicing factor, putative	0.65	0.0020	0.0000	0.0030	0.0145	0.0080	1.1	7274	306	6772	463	-267
920	PF11_0375	conserved Plasmodium protein, unknown function	0.53	0.0040	0.0003	0.0064	0.0219	0.0093	1.0	8499	288	8196	284	-269
527	PF10_0090	conserved Plasmodium protein, unknown function	0.64	0.0020	0.0000	0.0030	0.0145	0.0080	1.1	8219	308	7743	438	-269

682	MAL13P1.32	splicing factor, putative	0.59	0.0040	0.0003	0.0064	0.0219	0.0093	1.1	7305	176	6914	485	-270
1228	PF11_0366		0.46	0.0120	0.0050	0.0175	0.0456	0.0185	1.5	674	206	438	303	-274
257	PF08_0083	conserved Plasmodium protein, unknown function	0.76	0.0020	0.0000	0.0030	0.0145	0.0080	1.1	7767	631	6914	497	-275
500	PFE1390w	RNA helicase 1	0.65	0.0020	0.0000	0.0030	0.0145	0.0080	1.1	7629	379	7119	411	-279
1337	PF10_0340	methionine-tRNA ligase, putative	0.44	0.0100	0.0036	0.0149	0.0399	0.0162	1.0	9619	60	9398	443	-281
1102	MAL8P1.83	eukaryotic translation initiation factor, putative	0.49	0.0100	0.0036	0.0149	0.0399	0.0162	1.0	8493	247	8223	309	-286
299	MAL13P1.15	ribonuclease P protein subunit rpr, putative	0.73	0.0020	0.0000	0.0030	0.0145	0.0080	1.1	6600	318	5812	757	-288
179	PF07_0031	heat shock protein 86 family protein	0.82	0.0020	0.0000	0.0030	0.0145	0.0080	1.3	5359	775	4076	798	-290
653	PF11_0418	conserved Plasmodium protein, unknown function	0.60	0.0040	0.0003	0.0064	0.0219	0.0093	1.1	8329	298	7895	426	-291
573	PF14_0505	conserved Plasmodium protein, unknown function	0.62	0.0040	0.0003	0.0064	0.0219	0.0093	2.1	919	538	437	236	-292
699	PFL1620w	asparagine and aspartate rich protein 1	0.59	0.0040	0.0003	0.0064	0.0219	0.0093	1.1	8545	307	8129	405	-295
691	PF14_0636	conserved Plasmodium protein, unknown function	0.59	0.0060	0.0012	0.0094	0.0286	0.0120	1.1	7337	310	6912	411	-296
1012	PFA0220w	ubiquitin carboxyl-terminal hydrolase, putative	0.51	0.0100	0.0036	0.0149	0.0399	0.0162	1.0	8857	253	8551	349	-296
934	PF10_0176	conserved Plasmodium protein, unknown function	0.53	0.0080	0.0024	0.0122	0.0345	0.0142	1.5	947	351	616	276	-296
1202	PF13_0019	sodium/hydrogen exchanger, Na , H antiporter	0.47	0.0040	0.0003	0.0064	0.0219	0.0093	1.0	8587	229	8328	328	-297
949	PFC0486c	conserved Plasmodium protein, unknown function	0.52	0.0080	0.0024	0.0122	0.0345	0.0142	1.0	9362	191	9035	434	-297
488	PFI1215w	splicing factor 3A	0.65	0.0020	0.0000	0.0030	0.0145	0.0080	1.1	7030	416	6474	437	-298
735	PF13_0261	nucleolar preribosomal associated cytoplasmic ATPase, p	0.58	0.0060	0.0012	0.0094	0.0286	0.0120	1.1	8006	296	7597	412	-299
876	PF10_0074	conserved Plasmodium protein, unknown function	0.54	0.0080	0.0024	0.0122	0.0345	0.0142	1.0	7811	284	7454	374	-301
888	PFE1400c	beta adaptin protein, putative	0.54	0.0080	0.0024	0.0122	0.0345	0.0142	1.0	7553	220	7200	435	-302
734	PF14_0146	ribonucleoprotein, putative	0.58	0.0060	0.0012	0.0094	0.0286	0.0120	1.1	6803	293	6389	423	-302
1235	PFI0635c	conserved Plasmodium protein, unknown function	0.46	0.0100	0.0036	0.0149	0.0399	0.0162	1.0	7965	266	7706	296	-303
332	PF14_0355	conserved Plasmodium membrane protein, unknown fun	0.72	0.0020	0.0000	0.0030	0.0145	0.0080	1.1	6456	515	5689	556	-303
762	PFE0430w	ATP-dependent RNA helicase, putative	0.57	0.0060	0.0012	0.0094	0.0286	0.0120	1.1	8208	350	7794	373	-310
745	PF08_0120	GTPase activator, putative	0.58	0.0020	0.0000	0.0030	0.0145	0.0080	1.1	7275	144	6853	589	-311
768	MAL8P1.20	conserved Plasmodium protein, unknown function	0.57	0.0040	0.0003	0.0064	0.0219	0.0093	2.0	829	339	413	390	-313
864	MAL8P1.38	alpha/beta-hydrolase, putative	0.54	0.0040	0.0003	0.0064	0.0219	0.0093	2.0	761	472	386	217	-314
1160	PF08_0041	ribosome biogenesis protein nep1 homologue, putative	0.47	0.0120	0.0050	0.0175	0.0456	0.0185	1.0	8640	200	8354	403	-317
447	MAL8P1.204	DnaJ protein, putative	0.66	0.0020	0.0000	0.0030	0.0145	0.0080	1.1	7865	454	7230	500	-320
196	MAL7P1.75	mitochondrial ATP synthase F1, epsilon subunit, putative	0.80	0.0020	0.0000	0.0030	0.0145	0.0080	1.3	5550	777	4277	817	-321
1004	PFF0970w	splicing factor 3a subunit, putative	0.51	0.0100	0.0036	0.0149	0.0399	0.0162	1.0	7893	292	7557	367	-323
1559	PFC0155c	DNA-directed RNA polymerase subunit I, putative	0.40	0.0100	0.0036	0.0149	0.0399	0.0162	1.0	8387	340	8170	202	-324
335	PFF0490w	conserved Plasmodium protein, unknown function	0.72	0.0020	0.0000	0.0030	0.0145	0.0080	1.1	8002	211	7185	931	-324
708	PF11_0205	alternative splicing factor ASF-1, putative	0.58	0.0060	0.0012	0.0094	0.0286	0.0120	1.1	6868	383	6410	400	-325
229	PF07_0024	inositol phosphatase, putative	0.78	0.0020	0.0000	0.0030	0.0145	0.0080	1.2	6499	614	5367	844	-326
1024	PFF1475c	conserved Plasmodium protein, unknown function	0.51	0.0080	0.0024	0.0122	0.0345	0.0142	2.0	665	392	331	270	-327
363	PFC0425w	conserved Plasmodium protein, unknown function	0.70	0.0020	0.0000	0.0030	0.0145	0.0080	1.1	8395	468	7632	623	-328
550	PFL1835w	conserved Plasmodium protein, unknown function	0.63	0.0020	0.0000	0.0030	0.0145	0.0080	1.1	9221	229	8663	658	-329
880	PFI0195c	GTPase activator, putative	0.54	0.0060	0.0012	0.0094	0.0286	0.0120	1.0	8260	384	7870	335	-329
755	PF14_0565	conserved Plasmodium protein, unknown function	0.57	0.0020	0.0000	0.0030	0.0145	0.0080	1.1	9068	255	8624	518	-330
499	PF11_0396	protein phosphatase 2c	0.65	0.0020	0.0000	0.0030	0.0145	0.0080	1.1	8108	433	7504	502	-331
977	PFF0080c	TRAP-like protein, putative	0.52	0.0120	0.0050	0.0175	0.0456	0.0185	2.0	699	490	342	197	-331
456	PFD0920w	conserved Plasmodium protein, unknown function	0.66	0.0020	0.0000	0.0030	0.0145	0.0080	1.5	1995	523	1345	459	-331
183	PF11_0405		0.81	0.0020	0.0000	0.0030	0.0145	0.0080	2.0	2888	985	1460	776	-332
262	PFA0585w	conserved Plasmodium protein, unknown function	0.75	0.0020	0.0000	0.0030	0.0145	0.0080	1.2	7330	472	6313	878	-333
147	MAL13P1.19	conserved Plasmodium protein, unknown function	0.85	0.0020	0.0000	0.0030	0.0145	0.0080	2.8	2932	1347	1066	854	-334
1191	PFD0750w	nuclear cap-binding protein, putative	0.47	0.0060	0.0012	0.0094	0.0286	0.0120	1.0	7956	307	7661	323	-335

575	PFL1760w	conserved Plasmodium protein, unknown function	0.62	0.0040	0.0003	0.0064	0.0219	0.0093	1.1	7674	335	7122	552	-335
981	PFL1815c	conserved Plasmodium protein, unknown function	0.52	0.0060	0.0012	0.0094	0.0286	0.0120	1.0	8259	319	7900	375	-335
525	PFF0790c	conserved Plasmodium protein, unknown function	0.64	0.0020	0.0000	0.0030	0.0145	0.0080	1.1	7447	387	6851	544	-336
1052	PFE0385w	conserved Plasmodium protein, unknown function	0.50	0.0080	0.0024	0.0122	0.0345	0.0142	1.0	8847	327	8515	342	-337
190	MAL8P1.141	conserved Plasmodium protein, unknown function	0.81	0.0020	0.0000	0.0030	0.0145	0.0080	1.3	6407	929	5001	813	-337
869	PF13_0321	conserved Plasmodium protein, unknown function	0.54	0.0040	0.0003	0.0064	0.0219	0.0093	1.0	8775	309	8372	432	-338
576	MAL8P1.64	conserved Plasmodium protein, unknown function	0.62	0.0040	0.0003	0.0064	0.0219	0.0093	1.1	8216	361	7655	541	-341
387	PFE0880c	f-actin capping protein beta subunit, putative	0.69	0.0020	0.0000	0.0030	0.0145	0.0080	1.1	6425	523	5671	575	-344
247	PFI0945w	thioredoxin, putative	0.76	0.0020	0.0000	0.0030	0.0145	0.0080	1.2	6857	594	5753	857	-347
829	PF13_0310	small subunit rRNA processing factor, putative	0.55	0.0040	0.0003	0.0064	0.0219	0.0093	1.1	7840	292	7409	486	-348
1169	PF14_0221	GTPase, putative	0.47	0.0080	0.0024	0.0122	0.0345	0.0142	1.0	7306	268	6992	396	-350
379	PFL0530c	conserved Plasmodium protein, unknown function	0.69	0.0040	0.0003	0.0064	0.0219	0.0093	1.1	8784	333	8008	793	-350
254	PFL1870c	sphingomyelin phosphodiesterase, putative	0.76	0.0020	0.0000	0.0030	0.0145	0.0080	1.4	3515	793	2424	647	-350
477	PFB0410c	phospholipase A2, putative	0.65	0.0020	0.0000	0.0030	0.0145	0.0080	1.1	7342	475	6679	540	-351
356	PFI0915w	conserved Plasmodium protein, unknown function	0.70	0.0020	0.0000	0.0030	0.0145	0.0080	1.1	9027	171	8196	1012	-352
815	PF14_0407	guanine nucleotide exchange factor, putative	0.56	0.0120	0.0050	0.0175	0.0456	0.0185	1.1	8834	379	8390	419	-354
769	PFL1930w	conserved Plasmodium protein, unknown function	0.57	0.0080	0.0024	0.0122	0.0345	0.0142	1.6	1268	468	797	356	-354
439	PFC0635c	translation initiation factor 4E	0.67	0.0040	0.0003	0.0064	0.0219	0.0093	1.1	7486	539	6775	526	-354
873	MAL7P1.112	conserved Plasmodium protein, unknown function	0.54	0.0040	0.0003	0.0064	0.0219	0.0093	1.1	7301	267	6879	509	-354
1213	PF10_0158	conserved Plasmodium protein, unknown function	0.46	0.0100	0.0036	0.0149	0.0399	0.0162	1.0	8362	294	8051	374	-357
410	PFL0245w	probable protein, unknown function	0.68	0.0040	0.0003	0.0064	0.0219	0.0093	1.3	3239	645	2490	462	-358
702	PFL0520c	conserved Plasmodium protein, unknown function	0.59	0.0040	0.0003	0.0064	0.0219	0.0093	1.1	8081	201	7574	665	-359
1254	PFD1170c	Plasmodium exported protein (PHISTb), unknown functio	0.46	0.0120	0.0050	0.0175	0.0456	0.0185	1.0	9609	312	9306	353	-362
436	PF11_0407	adrenodoxin reductase, putative	0.67	0.0040	0.0003	0.0064	0.0219	0.0093	1.1	6686	413	5959	676	-362
382	PF10_0040	conserved Plasmodium protein, unknown function	0.69	0.0020	0.0000	0.0030	0.0145	0.0080	1.7	1989	534	1190	628	-363
1301	MAL13P1.93	small ribosomal subunit nuclear export protein, putative	0.45	0.0100	0.0036	0.0149	0.0399	0.0162	1.0	7850	257	7556	399	-363
359	PFI1560c	conserved Plasmodium membrane protein, unknown fun	0.70	0.0020	0.0000	0.0030	0.0145	0.0080	1.1	7375	326	6526	887	-363
428	PF11_0219	conserved Plasmodium protein, unknown function	0.67	0.0040	0.0003	0.0064	0.0219	0.0093	1.5	2269	581	1529	524	-365
1313	PF13_0177	DEAD/DEAH box ATP-dependent RNA helicase, putative	0.45	0.0120	0.0050	0.0175	0.0456	0.0185	1.0	7738	341	7443	320	-366
767	PFL0290w	conserved Plasmodium protein, unknown function	0.57	0.0040	0.0003	0.0064	0.0219	0.0093	1.1	8636	403	8139	467	-373
374	PF10_0098	conserved Plasmodium protein, unknown function	0.69	0.0020	0.0000	0.0030	0.0145	0.0080	1.1	8260	350	7421	863	-373
200	PFI0815c	methyltransferase, putative	0.79	0.0020	0.0000	0.0030	0.0145	0.0080	1.4	5515	636	4055	1202	-378
219	PFC0830w	trophozoite stage antigen	0.78	0.0020	0.0000	0.0030	0.0145	0.0080	1.5	4307	1050	2945	693	-380
317	MAL13P1.60	erythrocyte binding antigen-140	0.72	0.0020	0.0000	0.0030	0.0145	0.0080	1.3	4490	755	3489	627	-381
1016	PFI1055w	conserved Plasmodium protein, unknown function	0.51	0.0060	0.0012	0.0094	0.0286	0.0120	2.0	775	563	382	211	-381
847	PF08_0110	Rab GTPase 18	0.55	0.0060	0.0012	0.0094	0.0286	0.0120	1.1	6753	357	6289	489	-382
1319	PFE0210c	conserved Plasmodium protein, unknown function	0.45	0.0100	0.0036	0.0149	0.0399	0.0162	1.5	913	384	605	307	-383
333	PF10_0024	Plasmodium exported protein (hyp2), unknown function	0.72	0.0020	0.0000	0.0030	0.0145	0.0080	1.1	8065	410	7087	955	-387
358	PF11_0044	iron-sulfur assembly protein, sufD, putative	0.70	0.0020	0.0000	0.0030	0.0145	0.0080	1.8	2056	717	1148	579	-388
415	PF13_0302	phosphatase 2A regulatory subunit-related protein, putat	0.67	0.0020	0.0000	0.0030	0.0145	0.0080	1.7	1926	749	1120	445	-388
865	PFF0125c	conserved Plasmodium protein, unknown function	0.54	0.0020	0.0000	0.0030	0.0145	0.0080	1.1	6461	505	5997	348	-389
716	MAL13P1.461	probable protein, unknown function	0.58	0.0040	0.0003	0.0064	0.0219	0.0093	1.9	1150	546	604	392	-393
394	PFI0250c	conserved Plasmodium membrane protein, unknown fun	0.68	0.0040	0.0003	0.0064	0.0219	0.0093	1.1	6897	781	6049	460	-393
210	PFD0695w	conserved Plasmodium protein, unknown function	0.79	0.0020	0.0000	0.0030	0.0145	0.0080	1.5	4336	765	2875	1091	-395
193	PFE1480c	conserved Plasmodium protein, unknown function	0.80	0.0020	0.0000	0.0030	0.0145	0.0080	1.8	3604	1062	2011	926	-395
655	MAL7P1.73	calcium/calmodulin-dependent protein kinase, putative	0.60	0.0040	0.0003	0.0064	0.0219	0.0093	1.6	1584	521	995	463	-395
463	PFL2155w	conserved Plasmodium protein, unknown function	0.66	0.0020	0.0000	0.0030	0.0145	0.0080	2.7	1202	953	442	203	-396

1067	PF13_0343	conserved Plasmodium protein, unknown function	0.49	0.0080	0.0024	0.0122	0.0345	0.0142	1.0	8691	328	8305	455	-397
707	PFC0260w	P-loop containing nucleoside triphospahte hydrolase, put	0.58	0.0040	0.0003	0.0064	0.0219	0.0093	1.5	1741	600	1182	356	-398
1026	PF10_0179	conserved Plasmodium protein, unknown function	0.50	0.0120	0.0050	0.0175	0.0456	0.0185	1.1	8233	217	7827	589	-399
1563	PFB0105c	Plasmodium exported protein (PHISTc), unknown functio	0.40	0.0020	0.0000	0.0030	0.0145	0.0080	1.0	9826	90	9558	578	-400
312	PF11_0416	myosin F, putative	0.73	0.0020	0.0000	0.0030	0.0145	0.0080	1.3	4254	526	3185	944	-402
519	PF13_0291	minchromosome maintenance (MCM) complex subunit	0.64	0.0020	0.0000	0.0030	0.0145	0.0080	2.5	1204	822	490	294	-402
1013	MAL8P1.57	C-13 antigen	0.51	0.0080	0.0024	0.0122	0.0345	0.0142	1.1	6621	320	6207	496	-402
1114	PF08_0061	conserved Plasmodium protein, unknown function	0.48	0.0060	0.0012	0.0094	0.0286	0.0120	1.1	7198	336	6820	447	-405
754	PFF1440w	SET domain protein, putative	0.57	0.0060	0.0012	0.0094	0.0286	0.0120	1.1	6791	356	6242	599	-407
352	PFI1725w	Plasmodium exported protein, unknown function	0.70	0.0020	0.0000	0.0030	0.0145	0.0080	1.1	9040	323	8068	1056	-407
296	PFC0060c	serine/threonine protein kinase, FIKK family	0.73	0.0020	0.0000	0.0030	0.0145	0.0080	1.2	7510	487	6394	1037	-407
834	PF11_0047	actin-like protein, putative	0.55	0.0060	0.0012	0.0094	0.0286	0.0120	1.1	5806	345	5302	565	-407
249	PF11_0142	ubiquitin domain containing protein	0.76	0.0020	0.0000	0.0030	0.0145	0.0080	1.3	5401	912	4108	790	-408
867	PFC0690c	conserved Plasmodium protein, unknown function	0.54	0.0040	0.0003	0.0064	0.0219	0.0093	1.1	7467	328	6979	568	-408
420	PFL1255c	conserved Plasmodium protein, unknown function	0.67	0.0020	0.0000	0.0030	0.0145	0.0080	1.2	5025	507	4180	749	-411
375	PF10_0172	conserved Plasmodium protein, unknown function	0.69	0.0020	0.0000	0.0030	0.0145	0.0080	1.2	6823	516	5899	820	-412
390	PFL1935c	vesicle fusion and protein sorting subunit 16, putative	0.69	0.0020	0.0000	0.0030	0.0145	0.0080	1.1	7002	375	6105	934	-412
634	PFA0200w	thrombospondin-related sporozoite protein	0.61	0.0060	0.0012	0.0094	0.0286	0.0120	1.3	3075	431	2440	616	-413
660	PF11_0337	mitochondrial ribosomal protein L2 precursor	0.60	0.0040	0.0003	0.0064	0.0219	0.0093	1.1	6228	484	5619	539	-413
1212	PFE0390w	conserved Plasmodium protein, unknown function	0.47	0.0100	0.0036	0.0149	0.0399	0.0162	1.1	7272	311	6911	464	-415
266	PF10_0296	conserved Plasmodium protein, unknown function	0.75	0.0020	0.0000	0.0030	0.0145	0.0080	1.2	8427	698	7164	981	-417
487	PF07_0066	conserved Plasmodium protein, unknown function	0.65	0.0020	0.0000	0.0030	0.0145	0.0080	1.1	7246	537	6465	662	-418
678	PFI1430w	conserved Plasmodium protein, unknown function	0.59	0.0060	0.0012	0.0094	0.0286	0.0120	2.2	1139	647	528	385	-421
619	PFD0885c	conserved Plasmodium protein, unknown function	0.61	0.0060	0.0012	0.0094	0.0286	0.0120	1.1	8334	551	7676	529	-422
951	PF10_0324	conserved Plasmodium protein, unknown function	0.52	0.0080	0.0024	0.0122	0.0345	0.0142	1.1	6287	387	5823	500	-423
455	PF14_0709	mitochondrial ribosomal protein L20 precursor, putative	0.66	0.0020	0.0000	0.0030	0.0145	0.0080	1.6	2271	772	1439	483	-423
770	MAL8P1.78	small heat shock protein, putative	0.57	0.0080	0.0024	0.0122	0.0345	0.0142	1.1	4568	471	4005	516	-424
944	PF11_0234	conserved Plasmodium protein, unknown function	0.53	0.0100	0.0036	0.0149	0.0399	0.0162	1.2	3315	478	2844	418	-425
667	PFL1915w	DNA gyrase subunit B	0.60	0.0040	0.0003	0.0064	0.0219	0.0093	1.2	3659	584	3034	466	-425
256	PFL1270w	cof-like hydrolase, had-superfamily, subfamily iib	0.76	0.0020	0.0000	0.0030	0.0145	0.0080	1.2	8008	669	6684	1081	-426
579	PF10_0259	conserved Plasmodium protein, unknown function	0.62	0.0020	0.0000	0.0030	0.0145	0.0080	1.2	5048	582	4350	544	-427
466	MAL8P1.111	JmjC domain containing protein	0.66	0.0040	0.0003	0.0064	0.0219	0.0093	1.1	7158	579	6335	674	-430
1007	PFC0395w	asparagine synthetase, putative	0.51	0.0100	0.0036	0.0149	0.0399	0.0162	1.1	9150	322	8702	558	-432
884	MAL7P1.157a	RNA binding protein, putative	0.54	0.0020	0.0000	0.0030	0.0145	0.0080	1.1	5565	424	5050	527	-436
290	PFB0895c	replication factor C subunit 1, putative	0.74	0.0020	0.0000	0.0030	0.0145	0.0080	1.2	6093	459	4878	1193	-437
1010	PFL2270w	conserved protein, unknown function	0.51	0.0100	0.0036	0.0149	0.0399	0.0162	1.1	6748	429	6291	469	-442
407	PF14_0774	mannose-1-phosphate guanyltransferase, putative	0.68	0.0060	0.0012	0.0094	0.0286	0.0120	1.2	4862	641	3924	741	-444
793	PFA0480w	phenylalanyl-tRNA synthetase, putative	0.56	0.0060	0.0012	0.0094	0.0286	0.0120	1.1	8333	435	7761	583	-446
636	PF13_0327	cytochrome c oxidase subunit 2, putative	0.61	0.0020	0.0000	0.0030	0.0145	0.0080	1.4	2410	696	1727	434	-446
686	PFF0415c	conserved Plasmodium protein, unknown function	0.59	0.0100	0.0036	0.0149	0.0399	0.0162	1.1	5099	660	4453	433	-447
1090	MAL8P1.200	acetyltransferase, putative	0.49	0.0040	0.0003	0.0064	0.0219	0.0093	1.1	6429	391	6005	481	-447
617	MAL7P1.149	conserved Plasmodium protein, unknown function	0.61	0.0020	0.0000	0.0030	0.0145	0.0080	1.3	3045	506	2344	642	-448
857	PFD0980w	holo-(acyl-carrier protein) synthase, putative	0.55	0.0060	0.0012	0.0094	0.0286	0.0120	1.5	1559	541	1019	447	-448
998	PFL0160w	signal recognition particle SRP14	0.51	0.0060	0.0012	0.0094	0.0286	0.0120	1.1	7405	461	6932	460	-449
827	MAL13P1.35	U1 small nuclear ribonucleoprotein a, putative	0.55	0.0080	0.0024	0.0122	0.0345	0.0142	1.1	6250	563	5691	446	-449
341	PF11_0125	conserved Plasmodium protein, unknown function	0.71	0.0020	0.0000	0.0030	0.0145	0.0080	1.5	3276	753	2162	812	-450
785	PF14_0401	tRNA binding protein, putative	0.56	0.0060	0.0012	0.0094	0.0286	0.0120	1.1	8220	412	7635	623	-450

687	PFD1115c	conserved Plasmodium protein, unknown function	0.59	0.0020	0.0000	0.0030	0.0145	0.0080	1.1	6841	549	6190	553	-451
930	PFL1735c	RNA-processing protein, putative	0.53	0.0020	0.0000	0.0030	0.0145	0.0080	1.1	7545	322	7041	633	-451
810	PF10_0299	glycoprotease, putative	0.56	0.0040	0.0003	0.0064	0.0219	0.0093	1.1	7361	303	6785	731	-458
259	PFL0850w	anaphase promoting complex subunit 10, putative	0.75	0.0020	0.0000	0.0030	0.0145	0.0080	1.5	4411	772	3001	1097	-459
383	PF13_0200	conserved Plasmodium protein, unknown function	0.69	0.0040	0.0003	0.0064	0.0219	0.0093	1.5	3231	729	2219	743	-460
940	PF13_0069	translation initiation factor IF-2, putative	0.53	0.0080	0.0024	0.0122	0.0345	0.0142	1.6	1344	637	832	336	-461
270	PF10_0314	dcp1 homologue, putative	0.75	0.0020	0.0000	0.0030	0.0145	0.0080	1.2	7065	906	5688	932	-461
338	MAL13P1.279	protein kinase 5	0.71	0.0020	0.0000	0.0030	0.0145	0.0080	1.3	5438	661	4282	957	-463
723	PF11_0393	ubiquitin related modifier homologue	0.58	0.0020	0.0000	0.0030	0.0145	0.0080	1.1	6832	541	6186	571	-466
772	PFC0265c	conserved Plasmodium protein, unknown function	0.57	0.0040	0.0003	0.0064	0.0219	0.0093	1.1	6451	388	5832	699	-467
787	PF14_0511	glucose-6-phosphate dehydrogenase-6-phosphogluconol	0.56	0.0040	0.0003	0.0064	0.0219	0.0093	1.1	6422	543	5816	532	-469
714	PF10_0311	protein phosphatase inhibitor, putative	0.58	0.0040	0.0003	0.0064	0.0219	0.0093	1.1	7930	418	7274	707	-469
736	PF07_0109	conserved Plasmodium protein, unknown function	0.58	0.0040	0.0003	0.0064	0.0219	0.0093	1.9	1374	740	733	369	-469
561	PFL0385c	blood stage antigen 41-3 precursor	0.63	0.0020	0.0000	0.0030	0.0145	0.0080	1.5	2362	697	1566	575	-476
171	PF14_0374	CCAAT-binding transcription factor, putative	0.82	0.0020	0.0000	0.0030	0.0145	0.0080	1.5	6727	531	4530	2145	-478
792	PFF1410c	nicotinate phosphoribosyltransferase, putative	0.56	0.0020	0.0000	0.0030	0.0145	0.0080	1.1	9234	120	8619	974	-479
486	PF08_0044	protein kinase 1	0.65	0.0020	0.0000	0.0030	0.0145	0.0080	1.1	7794	570	6897	807	-480
727	PF14_0533	transcription factor with AP2 domain(s), putative	0.58	0.0020	0.0000	0.0030	0.0145	0.0080	1.4	2397	604	1734	540	-482
596	PFF0260w	serine/threonine protein kinase, putative	0.62	0.0020	0.0000	0.0030	0.0145	0.0080	1.3	3022	581	2240	684	-484
877	PFB0675w	conserved Plasmodium membrane protein, unknown fun	0.54	0.0020	0.0000	0.0030	0.0145	0.0080	1.1	6686	500	6108	565	-487
967	PF14_0622	potassium channel protein	0.52	0.0080	0.0024	0.0122	0.0345	0.0142	2.0	1046	591	517	426	-487
440	PFI1170c	thioredoxin reductase	0.67	0.0020	0.0000	0.0030	0.0145	0.0080	1.2	5426	565	4443	907	-490
901	PF14_0591	conserved Plasmodium protein, unknown function	0.54	0.0080	0.0024	0.0122	0.0345	0.0142	1.7	1403	590	839	465	-490
554	PF10_0245	glucosamine-fructose-6-phosphate aminotransferase, pu	0.63	0.0040	0.0003	0.0064	0.0219	0.0093	1.1	8046	475	7215	848	-492
621	PFE0305w	transcription initiation factor TFiid, TATA-binding protein	0.61	0.0040	0.0003	0.0064	0.0219	0.0093	1.1	8034	500	7268	759	-493
850	PFI1180w	patatin-like phospholipase, putative	0.55	0.0060	0.0012	0.0094	0.0286	0.0120	1.1	6318	447	5719	647	-495
618	PF08_0108	plasmepsin X	0.61	0.0020	0.0000	0.0030	0.0145	0.0080	1.2	5935	615	5159	659	-497
759	PFD1060w	u5 small nuclear ribonucleoprotein-specific protein, putat	0.57	0.0020	0.0000	0.0030	0.0145	0.0080	1.1	6294	442	5627	723	-498
811	PF10_0025	PF70 protein	0.56	0.0040	0.0003	0.0064	0.0219	0.0093	1.1	8814	528	8184	603	-501
1116	MAL7P1.160		0.48	0.0120	0.0050	0.0175	0.0456	0.0185	2.5	789	751	321	218	-502
610	PFC0160w	palmitoyl transferase	0.61	0.0040	0.0003	0.0064	0.0219	0.0093	1.2	4503	656	3710	641	-504
336	PFD0600c	mitochondrial ribosomal protein S12 precursor, putative	0.72	0.0020	0.0000	0.0030	0.0145	0.0080	1.2	6651	701	5385	1069	-504
757	PF14_0287	conserved Plasmodium protein, unknown function	0.57	0.0040	0.0003	0.0064	0.0219	0.0093	1.4	2585	624	1906	559	-505
357	PF14_0311	SF-assemblin, putative	0.70	0.0040	0.0003	0.0064	0.0219	0.0093	1.5	3435	872	2248	821	-506
1171	PFB0630c	conserved Plasmodium protein, unknown function	0.47	0.0100	0.0036	0.0149	0.0399	0.0162	1.1	7403	441	6949	519	-506
925	PFB0700c	conserved Plasmodium protein, unknown function	0.53	0.0120	0.0050	0.0175	0.0456	0.0185	1.1	6869	443	6301	632	-507
516	PF11_0311	N-acetyl glucosamine phosphate mutase, putative	0.64	0.0020	0.0000	0.0030	0.0145	0.0080	1.2	5817	465	4909	950	-507
274	PFF0680c	thiamin-phosphate pyrophosphorylase, putative	0.75	0.0020	0.0000	0.0030	0.0145	0.0080	2.0	2954	986	1466	1009	-508
592	PFC0310c	ATP-dependent Clp protease proteolytic subunit	0.62	0.0020	0.0000	0.0030	0.0145	0.0080	1.2	6011	551	5186	785	-510
496	PFL0370w	conserved Plasmodium protein, unknown function	0.65	0.0020	0.0000	0.0030	0.0145	0.0080	1.4	3194	695	2250	765	-516
777	PFC0582c		0.57	0.0060	0.0012	0.0094	0.0286	0.0120	1.1	6690	409	6010	787	-516
859	PF14_0086	tRNA-dihydrouridine synthase, putative	0.55	0.0040	0.0003	0.0064	0.0219	0.0093	1.1	7061	436	6437	709	-521
1021	MAL13P1.13	conserved Plasmodium protein, unknown function	0.51	0.0060	0.0012	0.0094	0.0286	0.0120	1.1	7054	520	6520	535	-521
398	PFI1690c	conserved Plasmodium protein, unknown function	0.68	0.0020	0.0000	0.0030	0.0145	0.0080	1.2	6024	880	4908	761	-525
583	PF14_0164	NADP-specific glutamate dehydrogenase	0.62	0.0020	0.0000	0.0030	0.0145	0.0080	1.1	9149	254	8291	1132	-527
495	PF11_0397	beta-catenin-like protein 1, putative	0.65	0.0020	0.0000	0.0030	0.0145	0.0080	1.2	6721	564	5750	934	-527
273	PFL2300w	conserved Plasmodium protein, unknown function	0.75	0.0020	0.0000	0.0030	0.0145	0.0080	1.5	4661	966	3103	1119	-527

915	PF14_0039	conserved Plasmodium protein, unknown function	0.53	0.0040	0.0003	0.0064	0.0219	0.0093	1.4	2085	632	1483	499	-530
594	PFE0055c	heat shock protein 40, type II	0.62	0.0060	0.0012	0.0094	0.0286	0.0120	1.1	9048	579	8183	821	-535
614	PFI0120c	serine/threonine protein kinase, FIKK family	0.61	0.0020	0.0000	0.0030	0.0145	0.0080	1.1	8808	439	7968	938	-536
936	PF13_0185	histone H3 variant, putative	0.53	0.0040	0.0003	0.0064	0.0219	0.0093	1.4	2124	628	1520	517	-541
624	PF11_0131	DNA helicase, putative	0.61	0.0020	0.0000	0.0030	0.0145	0.0080	2.3	1483	1088	637	304	-546
1075	PFA0370w	conserved Plasmodium protein, unknown function	0.49	0.0100	0.0036	0.0149	0.0399	0.0162	1.5	1695	610	1165	468	-548
1100	PFA0110w	ring-infected erythrocyte surface antigen	0.49	0.0040	0.0003	0.0064	0.0219	0.0093	1.1	9789	132	9270	936	-549
248	PFC0610c	zinc finger protein, putative	0.76	0.0020	0.0000	0.0030	0.0145	0.0080	1.5	5162	961	3418	1331	-549
630	PFI0565w	conserved Plasmodium protein, unknown function	0.61	0.0020	0.0000	0.0030	0.0145	0.0080	1.3	4093	630	3243	773	-553
371	PF14_0204	conserved Plasmodium protein, unknown function	0.69	0.0020	0.0000	0.0030	0.0145	0.0080	1.2	9457	331	8197	1484	-555
858	PF14_0604	conserved Plasmodium protein, unknown function	0.55	0.0060	0.0012	0.0094	0.0286	0.0120	1.1	7986	424	7317	802	-557
559	PF11_0386	apicoplast ribosomal protein S14p/S29e precursor, putati	0.63	0.0020	0.0000	0.0030	0.0145	0.0080	1.2	6161	708	5223	786	-557
891	PF11_0168a	serine esterase, putative	0.54	0.0020	0.0000	0.0030	0.0145	0.0080	1.6	1725	564	1069	653	-561
766	PFL0955c	raf kinase inhibitor	0.57	0.0040	0.0003	0.0064	0.0219	0.0093	1.6	1944	777	1195	535	-562
403	MAL13P1.46	conserved Plasmodium protein, unknown function	0.68	0.0020	0.0000	0.0030	0.0145	0.0080	1.6	3082	998	1889	758	-563
242	PFD0160w	conserved Plasmodium protein, unknown function	0.76	0.0040	0.0003	0.0064	0.0219	0.0093	1.5	5731	1102	3918	1275	-563
896	MAL13P1.31	conserved Plasmodium protein, unknown function	0.54	0.0040	0.0003	0.0064	0.0219	0.0093	1.1	6506	706	5848	518	-565
489	MAL13P1.20	secreted ookinete protein, putative	0.65	0.0020	0.0000	0.0030	0.0145	0.0080	1.2	5774	869	4717	755	-567
795	PF14_0171	conserved Plasmodium protein, unknown function	0.56	0.0040	0.0003	0.0064	0.0219	0.0093	1.1	5849	425	5121	873	-569
641	PF13_0080	telomerase reverse transcriptase, putative	0.60	0.0020	0.0000	0.0030	0.0145	0.0080	1.1	6941	404	6072	1038	-572
861	PFF0520w	calcium-dependent protein kinase	0.54	0.0060	0.0012	0.0094	0.0286	0.0120	1.2	5225	535	4538	725	-574
1044	PFI1155w	conserved Plasmodium protein, unknown function	0.50	0.0100	0.0036	0.0149	0.0399	0.0162	1.3	2632	552	2060	594	-575
540	PF10_0142	conserved Plasmodium protein, unknown function	0.63	0.0020	0.0000	0.0030	0.0145	0.0080	1.3	4945	851	3942	731	-578
978	PFL0125c	conserved Plasmodium protein, unknown function	0.52	0.0100	0.0036	0.0149	0.0399	0.0162	1.1	6051	659	5430	541	-578
555	MAL8P1.97	hypothetical protein	0.63	0.0020	0.0000	0.0030	0.0145	0.0080	2.7	1553	1038	576	517	-578
830	PFC0765c	conserved Plasmodium protein, unknown function	0.55	0.0060	0.0012	0.0094	0.0286	0.0120	1.1	6793	451	6077	844	-578
731	PF11_0464a	conserved Plasmodium protein, unknown function	0.58	0.0040	0.0003	0.0064	0.0219	0.0093	1.1	7012	531	6213	851	-583
570	PFE1280w	conserved Plasmodium protein, unknown function	0.62	0.0020	0.0000	0.0030	0.0145	0.0080	1.7	2271	636	1301	919	-585
943	PF14_0660	protein phosphatase, putative	0.53	0.0080	0.0024	0.0122	0.0345	0.0142	1.3	2879	593	2228	645	-587
838	PFI1135c	OTU-like cysteine protease, putative	0.55	0.0060	0.0012	0.0094	0.0286	0.0120	1.4	2496	677	1766	646	-593
556	PF14_0413	CAF1 family ribonuclease, putative	0.63	0.0040	0.0003	0.0064	0.0219	0.0093	1.2	5358	765	4357	829	-593
644	PF10_0131	conserved Plasmodium protein, unknown function	0.60	0.0020	0.0000	0.0030	0.0145	0.0080	1.3	3999	749	3102	742	-593
991	PFE0895c	zinc finger protein, putative	0.52	0.0040	0.0003	0.0064	0.0219	0.0093	1.1	6131	567	5498	662	-596
491	PFE0415w	transcription factor IIb, putative	0.65	0.0020	0.0000	0.0030	0.0145	0.0080	1.3	4400	726	3290	981	-597
860	PF14_0430	mitochondrial ribosomal protein S29 precursor, putative	0.54	0.0040	0.0003	0.0064	0.0219	0.0093	1.3	2926	657	2208	661	-600
1133	MAL7P1.6	Plasmodium exported protein (hyp12), unknown functior	0.48	0.0100	0.0036	0.0149	0.0399	0.0162	1.1	9812	201	9258	957	-604
517	PFI0235w	replication factor A-related protein, putative	0.64	0.0020	0.0000	0.0030	0.0145	0.0080	1.4	4143	843	3062	844	-606
432	PF14_0483	conserved Plasmodium protein, unknown function	0.67	0.0020	0.0000	0.0030	0.0145	0.0080	1.8	2707	1029	1473	815	-611
588	PFL1195w	conserved Plasmodium protein, unknown function	0.62	0.0020	0.0000	0.0030	0.0145	0.0080	1.2	7560	461	6568	1143	-611
817	PFB0467w	50S ribosomal protein L33, putative	0.56	0.0060	0.0012	0.0094	0.0286	0.0120	1.3	3163	639	2392	746	-614
1459	PFB0900c	Plasmodium exported protein (PHISTc), unknown functio	0.42	0.0120	0.0050	0.0175	0.0456	0.0185	1.0	9709	86	9260	981	-618
907	PFI0970c	TLD domain containing protein	0.53	0.0040	0.0003	0.0064	0.0219	0.0093	1.1	7834	348	7127	978	-619
492	PF14_0521	conserved Plasmodium protein, unknown function	0.65	0.0020	0.0000	0.0030	0.0145	0.0080	1.9	2402	1256	1250	517	-620
1195	PFI0175w		0.47	0.0040	0.0003	0.0064	0.0219	0.0093	1.5	1719	584	1175	580	-620
794	PFB0765w	conserved Plasmodium protein, unknown function	0.56	0.0040	0.0003	0.0064	0.0219	0.0093	2.1	1533	754	736	665	-622
732	PF14_0460		0.58	0.0080	0.0024	0.0122	0.0345	0.0142	1.3	3450	626	2593	856	-625
871	PFC0305w	EB1 homolog, putative	0.54	0.0040	0.0003	0.0064	0.0219	0.0093	1.2	4271	604	3527	766	-625

1043	PF14_0139	conserved Plasmodium protein, unknown function	0.50	0.0100	0.0036	0.0149	0.0399	0.0162	1.1	8224	468	7600	784	-627
414	PFB0732c	conserved Plasmodium protein, unknown function	0.68	0.0020	0.0000	0.0030	0.0145	0.0080	1.2	7558	764	6249	1172	-628
746	MAL13P1.10(	conserved Plasmodium protein, unknown function	0.58	0.0020	0.0000	0.0030	0.0145	0.0080	1.5	2684	797	1831	685	-629
302	PFB0920w	DnaJ protein, putative	0.73	0.0020	0.0000	0.0030	0.0145	0.0080	1.3	7456	803	5749	1536	-631
852	PFE0450w	chromosome condensation protein, putative	0.55	0.0080	0.0024	0.0122	0.0345	0.0142	1.2	4542	566	3777	833	-633
791	PFD0385w	conserved Plasmodium protein, unknown function	0.56	0.0040	0.0003	0.0064	0.0219	0.0093	1.7	2001	624	1187	823	-633
462	MAL13P1.32	aldo-keto reductase, putative	0.66	0.0020	0.0000	0.0030	0.0145	0.0080	1.2	6564	757	5335	1107	-634
673	MAL7P1.125	conserved Plasmodium protein, unknown function	0.59	0.0040	0.0003	0.0064	0.0219	0.0093	1.6	2571	598	1641	967	-635
552	PFI0540w	conserved Plasmodium protein, unknown function	0.63	0.0060	0.0012	0.0094	0.0286	0.0120	1.3	4655	734	3578	980	-637
928	MAL7P1.204	conserved Plasmodium protein, unknown function	0.53	0.0060	0.0012	0.0094	0.0286	0.0120	1.1	6497	600	5777	763	-643
1174	PF14_0132	40S ribosomal protein S9A, putative	0.47	0.0080	0.0024	0.0122	0.0345	0.0142	1.3	2503	585	1924	640	-646
562	PF10_0053	methionine-tRNA ligase, putative	0.63	0.0020	0.0000	0.0030	0.0145	0.0080	1.2	5807	667	4725	1063	-647
902	PFF0475w	hypothetical protein	0.53	0.0080	0.0024	0.0122	0.0345	0.0142	1.2	4902	671	4159	720	-648
982	PFL1275c	conserved Plasmodium protein, unknown function	0.52	0.0100	0.0036	0.0149	0.0399	0.0162	1.2	3551	588	2855	758	-650
954	PF07_0022	conserved Plasmodium protein, unknown function	0.52	0.0060	0.0012	0.0094	0.0286	0.0120	1.2	4680	651	3968	713	-652
539	PF14_0451	mitochondrial ribosomal protein S14 precursor, putative	0.63	0.0020	0.0000	0.0030	0.0145	0.0080	1.4	3824	856	2692	929	-652
889	PFE1200w	conserved Plasmodium protein, unknown function	0.54	0.0080	0.0024	0.0122	0.0345	0.0142	1.1	7105	627	6341	790	-653
828	PFB0925w	DnaJ protein, putative	0.55	0.0040	0.0003	0.0064	0.0219	0.0093	2.0	1645	941	833	523	-653
481	PFF0800w	TRAP-like protein	0.65	0.0020	0.0000	0.0030	0.0145	0.0080	1.4	4175	665	2942	1223	-655
914	PF14_0030	conserved Plasmodium protein, unknown function	0.53	0.0060	0.0012	0.0094	0.0286	0.0120	1.5	2264	667	1506	759	-667
508	PFL1695c	conserved Plasmodium protein, unknown function	0.64	0.0020	0.0000	0.0030	0.0145	0.0080	1.3	5880	947	4661	944	-672
983	MAL13P1.303		0.52	0.0060	0.0012	0.0094	0.0286	0.0120	1.3	3136	802	2415	592	-674
680	PFE1030c	phosphomethylpyrimidine kinase, putative	0.59	0.0080	0.0024	0.0122	0.0345	0.0142	1.9	2075	995	1092	665	-677
603	PFI0925w	gamma-glutamylcysteine synthetase	0.61	0.0020	0.0000	0.0030	0.0145	0.0080	1.3	5333	572	4255	1183	-677
480	PFE1125w	mitochondrial ribosomal protein L17 precursor, putative	0.65	0.0060	0.0012	0.0094	0.0286	0.0120	1.6	3364	978	2083	983	-680
872	PFI1660w	conserved Plasmodium protein, unknown function	0.54	0.0060	0.0012	0.0094	0.0286	0.0120	1.2	5146	704	4336	786	-681
1167	PFF1375c		0.47	0.0040	0.0003	0.0064	0.0219	0.0093	1.1	9783	111	9166	1191	-686
1056	PF10_0244	formin 2, putative	0.49	0.0080	0.0024	0.0122	0.0345	0.0142	1.1	7649	613	6976	746	-686
405	PFA0135w	merozoite-associated tryptophan-rich antigen, putative	0.68	0.0020	0.0000	0.0030	0.0145	0.0080	1.2	8917	546	7466	1592	-687
1204	PFB0560w	conserved Plasmodium protein, unknown function	0.47	0.0060	0.0012	0.0094	0.0286	0.0120	1.1	6672	936	6069	358	-690
427	PFD1180w	Plasmodium exported protein (PHISTb), unknown functio	0.67	0.0020	0.0000	0.0030	0.0145	0.0080	1.2	8855	459	7455	1633	-691
955	PF11_0394	conserved Plasmodium membrane protein, unknown fun	0.52	0.0100	0.0036	0.0149	0.0399	0.0162	1.1	6765	478	6010	969	-691
1103	PFD1135c	probable protein, unknown function	0.48	0.0060	0.0012	0.0094	0.0286	0.0120	1.1	8334	362	7683	980	-691
395	PFB0020c	erythrocyte membrane protein 1 (PfEMP1), exon2	0.68	0.0020	0.0000	0.0030	0.0145	0.0080	1.3	5877	843	4388	1338	-692
674	PF13_0196	MSP7-like protein	0.59	0.0040	0.0003	0.0064	0.0219	0.0093	1.3	4048	578	3033	1130	-694
1179	MAL7P1.158	signal recognition particle SRP9	0.47	0.0100	0.0036	0.0149	0.0399	0.0162	1.1	6902	499	6281	817	-694
662	MAL13P1.28	glutamate--tRNA ligase, putative	0.60	0.0020	0.0000	0.0030	0.0145	0.0080	1.5	3259	915	2232	808	-696
536	MAL8P1.46	dynein light chain 1	0.64	0.0020	0.0000	0.0030	0.0145	0.0080	1.3	5671	985	4456	928	-698
1259	MAL7P1.177	Plasmodium exported protein (hyp9), unknown function	0.46	0.0100	0.0036	0.0149	0.0399	0.0162	1.1	5141	488	4557	795	-699
783	PFA0215w		0.57	0.0100	0.0036	0.0149	0.0399	0.0162	1.1	7510	816	6593	804	-703
837	MAL13P1.123		0.55	0.0100	0.0036	0.0149	0.0399	0.0162	1.3	3603	483	2737	1087	-704
657	PFB0355c	serine repeat antigen 2	0.60	0.0040	0.0003	0.0064	0.0219	0.0093	1.4	3778	828	2728	927	-705
788	PF13_0079	conserved Plasmodium protein, unknown function	0.56	0.0020	0.0000	0.0030	0.0145	0.0080	1.4	3422	696	2509	924	-707
1105	PF10_0162	Plasmodium exported protein (PHISTc), unknown functio	0.48	0.0100	0.0036	0.0149	0.0399	0.0162	1.1	7946	630	7282	742	-708
807	PFE0225w	3-methyl-2-oxobutanoate dehydrogenase (lipoamide), pu	0.56	0.0040	0.0003	0.0064	0.0219	0.0093	1.2	4934	802	4042	798	-708
1118	PFE1435c	nucleolar preribosomal GTPase, putative	0.48	0.0080	0.0024	0.0122	0.0345	0.0142	1.1	6875	597	6214	776	-711
958	PF14_0179	liver specific protein 1, putative	0.52	0.0060	0.0012	0.0094	0.0286	0.0120	2.0	1591	861	811	633	-714

993	PFL0635c	bromodomain protein, putative	0.51	0.0040	0.0003	0.0064	0.0219	0.0093	1.1	8878	538	8121	935	-716
894	MAL8P1.137	conserved Plasmodium protein, unknown function	0.54	0.0080	0.0024	0.0122	0.0345	0.0142	1.6	2262	834	1423	724	-720
798	PFB0360c	serine repeat antigen 1	0.56	0.0020	0.0000	0.0030	0.0145	0.0080	1.7	2201	1072	1275	580	-725
1094	PFI0105c	serine/threonine protein kinase, FIKK family	0.49	0.0120	0.0050	0.0175	0.0456	0.0185	1.1	7625	678	6938	734	-725
908	PF10_0277	nucleolar preribosomal assembly protein, putative	0.53	0.0040	0.0003	0.0064	0.0219	0.0093	1.1	6854	724	6024	832	-726
1063	PFI0760w	conserved Plasmodium protein, unknown function	0.49	0.0100	0.0036	0.0149	0.0399	0.0162	1.1	6788	580	6080	854	-727
987	PF14_0212	mitochondrial ribosomal protein L21 precursor, putative	0.52	0.0040	0.0003	0.0064	0.0219	0.0093	1.4	2741	865	1959	648	-732
756	PFF0290w	long chain polyunsaturated fatty acid elongation enzyme,	0.57	0.0040	0.0003	0.0064	0.0219	0.0093	1.1	9549	182	8562	1538	-733
698	PFI0470w	FHA domain protein, putative	0.59	0.0020	0.0000	0.0030	0.0145	0.0080	1.2	7280	780	6224	1021	-744
1490	PF14_0370	DEAD/DEAH box helicase, putative	0.42	0.0120	0.0050	0.0175	0.0456	0.0185	1.1	6590	519	6059	756	-744
854	PF11_0295	farnesyl pyrophosphate synthase, putative	0.55	0.0120	0.0050	0.0175	0.0456	0.0185	1.2	5431	468	4519	1198	-754
637	PF14_0694	protein disulfide isomerase	0.60	0.0040	0.0003	0.0064	0.0219	0.0093	1.4	4316	1032	3162	877	-754
1087	PF14_0106	ankyrin-repeat protein, putative	0.49	0.0060	0.0012	0.0094	0.0286	0.0120	1.3	3226	755	2505	723	-756
1295	PF14_0274	diphthamide synthesis protein, putative	0.45	0.0100	0.0036	0.0149	0.0399	0.0162	1.1	6623	690	6008	683	-757
1306	PF10_0182	conserved Plasmodium protein, unknown function	0.45	0.0100	0.0036	0.0149	0.0399	0.0162	1.1	6584	689	5970	684	-760
910	PF11_0286	protein disulfide isomerase, putative	0.53	0.0080	0.0024	0.0122	0.0345	0.0142	1.3	4133	543	3267	1084	-761
900	PFI1045w	conserved Plasmodium protein, unknown function	0.54	0.0040	0.0003	0.0064	0.0219	0.0093	1.4	3310	947	2427	700	-764
972	PFF0995c	merozoite surface protein 10	0.52	0.0100	0.0036	0.0149	0.0399	0.0162	1.2	5020	614	4188	988	-769
649	MAL13P1.18[illegible]	conserved Plasmodium protein, unknown function	0.60	0.0040	0.0003	0.0064	0.0219	0.0093	1.5	3709	1024	2521	952	-789
1284	PFL1770c	conserved Plasmodium protein, unknown function	0.45	0.0120	0.0050	0.0175	0.0456	0.0185	1.3	3214	661	2566	778	-790
1270	PFL2315c	conserved Plasmodium membrane protein, unknown fun	0.45	0.0120	0.0050	0.0175	0.0456	0.0185	1.1	6891	559	6233	890	-792
965	PF13_0193	MSP7-like protein	0.52	0.0120	0.0050	0.0175	0.0456	0.0185	1.2	5630	657	4766	1002	-794
806	PF10_0124	protein phosphatase, putative	0.56	0.0040	0.0003	0.0064	0.0219	0.0093	1.6	2734	944	1730	855	-795
895	PFL2290w	dipeptidyl peptidase 2, putative	0.54	0.0040	0.0003	0.0064	0.0219	0.0093	1.2	5180	706	4254	1015	-795
875	PF14_0560	conserved Plasmodium protein, unknown function	0.54	0.0040	0.0003	0.0064	0.0219	0.0093	1.2	5885	895	4939	846	-796
843	PFI0460w	conserved Plasmodium protein, unknown function	0.55	0.0020	0.0000	0.0030	0.0145	0.0080	1.2	6053	681	5084	1085	-796
856	PF13_0306	dynein light chain, putative	0.55	0.0060	0.0012	0.0094	0.0286	0.0120	1.8	2186	972	1227	784	-797
571	PF08_0119	conserved Plasmodium protein, unknown function	0.62	0.0040	0.0003	0.0064	0.0219	0.0093	1.2	9086	527	7756	1606	-803
1155	PF10_0163	Plasmodium exported protein (PHISTc), unknown functio	0.48	0.0040	0.0003	0.0064	0.0219	0.0093	1.1	8745	516	8017	1016	-804
822	PF07_0041	conserved Plasmodium protein, unknown function	0.56	0.0060	0.0012	0.0094	0.0286	0.0120	1.2	6220	709	5213	1102	-805
566	MAL13P1.26[illegible]	conserved Plasmodium protein, unknown function	0.62	0.0020	0.0000	0.0030	0.0145	0.0080	1.2	9043	423	7701	1726	-807
851	PFL0140w	conserved Plasmodium protein, unknown function	0.55	0.0080	0.0024	0.0122	0.0345	0.0142	1.2	5641	663	4660	1129	-810
832	PF11_0045	CPW-WPC family protein	0.55	0.0060	0.0012	0.0094	0.0286	0.0120	1.3	4206	1078	3195	751	-818
1038	PFA0305c	conserved Plasmodium protein, unknown function	0.50	0.0120	0.0050	0.0175	0.0456	0.0185	1.4	2870	596	2042	1056	-824
669	PF11_0218	conserved Plasmodium protein, unknown function	0.59	0.0040	0.0003	0.0064	0.0219	0.0093	1.2	6915	942	5704	1096	-826
816	PF11_0297	NOT family protein, putative	0.56	0.0020	0.0000	0.0030	0.0145	0.0080	1.2	5888	708	4851	1156	-827
366	PFF0285c	DNA repair-like protein, putative	0.70	0.0020	0.0000	0.0030	0.0145	0.0080	1.6	5252	1261	3319	1508	-837
1380	PFI1080w	dynein intermediate chain 2, ciliary	0.43	0.0120	0.0050	0.0175	0.0456	0.0185	1.2	4287	755	3639	733	-841
1085	PFF0555w	conserved Plasmodium protein, unknown function	0.49	0.0100	0.0036	0.0149	0.0399	0.0162	1.1	6740	841	5934	809	-844
771	PFI0890c		0.57	0.0040	0.0003	0.0064	0.0219	0.0093	1.3	4941	939	3820	1027	-844
1149	PF13_0188	conserved Plasmodium protein, unknown function	0.48	0.0120	0.0050	0.0175	0.0456	0.0185	1.2	5892	902	5121	720	-850
697	PFL2110c	conserved protein, unknown function	0.59	0.0020	0.0000	0.0030	0.0145	0.0080	1.6	3138	799	1924	1270	-855
683	PFL0170w	transporter, putative	0.59	0.0020	0.0000	0.0030	0.0145	0.0080	1.3	6055	895	4814	1202	-857
1008	PF11_0243	leucine-rich repeat protein	0.51	0.0100	0.0036	0.0149	0.0399	0.0162	1.1	7223	772	6334	974	-857
809	PFC0090w	Plasmodium exported protein, unknown function	0.56	0.0020	0.0000	0.0030	0.0145	0.0080	1.1	9779	147	8699	1790	-858
989	PFD0400w	conserved Plasmodium protein, unknown function	0.52	0.0080	0.0024	0.0122	0.0345	0.0142	1.5	2853	870	1938	903	-858
1230	PF11_0034	DnaJ protein, putative	0.46	0.0100	0.0036	0.0149	0.0399	0.0162	1.1	7234	838	6496	759	-859

898	MAL13P1.292	riboflavin kinase / FAD synthase family protein, putative	0.54	0.0100	0.0036	0.0149	0.0399	0.0162	1.2	5916	734	4921	1121	-859
1216	PFI1095w	conserved Plasmodium protein, unknown function	0.46	0.0100	0.0036	0.0149	0.0399	0.0162	1.2	4911	668	4164	938	-860
879	MAL7P1.203	conserved Plasmodium protein, unknown function	0.54	0.0020	0.0000	0.0030	0.0145	0.0080	1.3	4358	841	3335	1044	-862
863	MAL8P1.55	conserved Plasmodium protein, unknown function	0.54	0.0040	0.0003	0.0064	0.0219	0.0093	1.3	4278	786	3244	1112	-865
1049	MAL7P1.23	RAP protein, putative	0.50	0.0100	0.0036	0.0149	0.0399	0.0162	1.4	2950	805	2089	926	-870
1092	PFL2255w	conserved Plasmodium protein, unknown function	0.49	0.0100	0.0036	0.0149	0.0399	0.0162	1.2	4318	798	3491	900	-871
1129	PF14_0192	glutathione reductase	0.48	0.0100	0.0036	0.0149	0.0399	0.0162	1.4	2830	882	2025	794	-871
939	PFC0565w	GTP-binding protein EngA, putative	0.53	0.0080	0.0024	0.0122	0.0345	0.0142	1.3	3757	716	2787	1126	-872
485	PF08_0073	conserved Plasmodium protein, unknown function	0.65	0.0020	0.0000	0.0030	0.0145	0.0080	1.4	6213	1133	4576	1378	-874
1143	PF11_0092	mechanosensitive ion channel protein	0.48	0.0080	0.0024	0.0122	0.0345	0.0142	1.2	5442	537	4634	1157	-886
765	PFE0855c	conserved Plasmodium protein, unknown function	0.57	0.0100	0.0036	0.0149	0.0399	0.0162	5.3	1461	1707	278	363	-887
848	PF14_0594	conserved Plasmodium protein, unknown function	0.55	0.0060	0.0012	0.0094	0.0286	0.0120	1.2	6216	966	5140	997	-887
1066	PFL0195w	conserved Plasmodium protein, unknown function	0.49	0.0120	0.0050	0.0175	0.0456	0.0185	1.3	3596	947	2730	809	-890
1020	PFF1210w	phosphatidic acid phosphatase	0.51	0.0060	0.0012	0.0094	0.0286	0.0120	1.3	4385	799	3469	1012	-894
1079	PF11_0415	conserved Plasmodium protein, unknown function	0.49	0.0120	0.0050	0.0175	0.0456	0.0185	1.1	6855	847	5986	926	-904
1005	PF11_0371	conserved Plasmodium protein, unknown function	0.51	0.0080	0.0024	0.0122	0.0345	0.0142	1.2	4933	762	3992	1084	-905
676	PFE1255w	conserved Plasmodium protein, unknown function	0.59	0.0040	0.0003	0.0064	0.0219	0.0093	1.2	7687	974	6365	1255	-908
391	PF11_0041	Plasmodium exported protein (hyp11), unknown function	0.68	0.0020	0.0000	0.0030	0.0145	0.0080	1.5	5913	1064	3938	1818	-908
1327	PFL2435w	conserved Plasmodium protein, unknown function	0.44	0.0100	0.0036	0.0149	0.0399	0.0162	1.2	5458	617	4731	1022	-911
1061	PFB0670c	conserved Plasmodium protein, unknown function	0.49	0.0080	0.0024	0.0122	0.0345	0.0142	1.3	3457	626	2564	1179	-913
1107	PF10_0097	mitochondrial ribosomal protein L22/L43, putative	0.48	0.0040	0.0003	0.0064	0.0219	0.0093	1.1	7044	469	6189	1300	-914
689	PFE0035c	rifin, pseudogene	0.59	0.0060	0.0012	0.0094	0.0286	0.0120	1.9	2870	1196	1539	1060	-925
1022	PFL1465c	heat shock protein hslv	0.51	0.0120	0.0050	0.0175	0.0456	0.0185	1.5	2742	988	1793	887	-926
1086	MAL7P1.206	DNA mismatch repair protein, putative	0.49	0.0100	0.0036	0.0149	0.0399	0.0162	1.1	6944	732	6059	1079	-926
721	PF14_0610	zinc finger protein, putative	0.58	0.0060	0.0012	0.0094	0.0286	0.0120	1.3	5215	1122	3928	1095	-930
1128	PF11_0127	conserved Plasmodium protein, unknown function	0.48	0.0120	0.0050	0.0175	0.0456	0.0185	1.2	5235	730	4374	1061	-931
457	PFL2650w	Plasmodium exported protein, unknown function, fragme	0.66	0.0020	0.0000	0.0030	0.0145	0.0080	2.0	3700	1587	1865	1184	-936
518	PFE1500c	conserved Plasmodium protein, unknown function	0.64	0.0040	0.0003	0.0064	0.0219	0.0093	1.4	5940	852	4269	1757	-938
1019	MAL8P1.72	high mobility group protein	0.51	0.0040	0.0003	0.0064	0.0219	0.0093	1.2	6082	975	5118	928	-939
1000	PFE0875c	conserved Plasmodium protein, unknown function	0.51	0.0060	0.0012	0.0094	0.0286	0.0120	1.2	5464	886	4460	1070	-953
643	MAL7P1.66	mitochondrial ribosomal protein S5 precursor, putative	0.60	0.0020	0.0000	0.0030	0.0145	0.0080	2.5	2425	1594	972	818	-960
664	PFC0905c	oocyst capsule protein	0.60	0.0040	0.0003	0.0064	0.0219	0.0093	1.6	3958	1003	2538	1381	-964
605	PFC0695w	conserved Plasmodium protein, unknown function	0.61	0.0040	0.0003	0.0064	0.0219	0.0093	1.4	5151	966	3611	1541	-968
1082	PF10_0022	Plasmodium exported protein (PHISTc), unknown functio	0.49	0.0080	0.0024	0.0122	0.0345	0.0142	1.1	8134	652	7198	1261	-977
1157	PFL2540w	Plasmodium exported protein (PHISTb), unknown functio	0.47	0.0080	0.0024	0.0122	0.0345	0.0142	1.2	5863	1039	4980	821	-977
1040	PFD0970c	zinc finger protein, putative	0.50	0.0100	0.0036	0.0149	0.0399	0.0162	1.2	6043	1006	5066	951	-980
853	PF10_0333	conserved Plasmodium protein, unknown function	0.55	0.0060	0.0012	0.0094	0.0286	0.0120	1.3	5426	1026	4235	1150	-985
1006	PF11_0381	subtilisin-like protease 2	0.51	0.0120	0.0050	0.0175	0.0456	0.0185	1.3	4560	848	3532	1171	-991
866	PF14_0670	conserved Plasmodium protein, unknown function	0.54	0.0060	0.0012	0.0094	0.0286	0.0120	1.3	5590	1103	4407	1071	-991
1166	PF14_0245	conserved Plasmodium protein, unknown function	0.47	0.0100	0.0036	0.0149	0.0399	0.0162	1.2	5866	811	4973	1075	-993
942	PF13_0211	calcium dependent protein kinase 5	0.53	0.0040	0.0003	0.0064	0.0219	0.0093	1.3	4499	943	3390	1165	-999
612	PF11_0509	ring-infected erythrocyte surface antigen	0.61	0.0020	0.0000	0.0030	0.0145	0.0080	1.2	8164	832	6587	1750	-1005
688	MAL7P1.178	alpha/beta hydrolase, putative	0.59	0.0020	0.0000	0.0030	0.0145	0.0080	1.2	8415	417	6965	2038	-1005
924	PFL1110c	cAMP-dependent protein kinase regulatory subunit	0.53	0.0040	0.0003	0.0064	0.0219	0.0093	1.8	2579	1013	1446	1128	-1008
1136	PF08_0043	conserved Plasmodium protein, unknown function	0.48	0.0120	0.0050	0.0175	0.0456	0.0185	1.2	5357	869	4425	1083	-1020
801	PFL0550w	HSP20-like chaperone	0.56	0.0080	0.0024	0.0122	0.0345	0.0142	1.4	4824	1039	3527	1279	-1020
1203	PFI0115c	serine/threonine protein kinase, FIKK family	0.47	0.0120	0.0050	0.0175	0.0456	0.0185	1.2	6782	958	5888	960	-1024

459	PF14_0749	acyl-CoA binding protein	0.66	0.0040	0.0003	0.0064	0.0219	0.0093	1.5	6289	1222	4281	1812	-1026
430	PFA0610c	Plasmodium exported protein (hyp11), unknown functior	0.67	0.0020	0.0000	0.0030	0.0145	0.0080	1.4	7635	673	5525	2479	-1043
926	PFC10_API00	null	0.53	0.0040	0.0003	0.0064	0.0219	0.0093	1.2	7913	840	6715	1427	-1069
665	PF14_0120	conserved Plasmodium protein, unknown function	0.60	0.0020	0.0000	0.0030	0.0145	0.0080	2.8	2509	1785	909	901	-1086
728	PFB0645c	mitochondrial ribosomal protein L13 precursor, putative	0.58	0.0080	0.0024	0.0122	0.0345	0.0142	1.4	4940	1322	3423	1297	-1102
1032	PFI0170w	conserved Plasmodium protein, unknown function	0.50	0.0060	0.0012	0.0094	0.0286	0.0120	1.2	5895	1171	4783	1044	-1103
1460	PFB0440c	conserved Plasmodium protein, unknown function	0.42	0.0120	0.0050	0.0175	0.0456	0.0185	1.2	5763	801	4960	1109	-1107
706	PFB0110w	Plasmodium exported protein (hyp11), unknown functior	0.58	0.0100	0.0036	0.0149	0.0399	0.0162	4.4	2017	1869	460	797	-1108
718	PF08_0052	perforin like protein 5	0.58	0.0020	0.0000	0.0030	0.0145	0.0080	1.5	4530	1159	2980	1508	-1117
878	PF10_0239		0.54	0.0060	0.0012	0.0094	0.0286	0.0120	1.4	5025	1076	3689	1387	-1127
1229	PF11_0282	deoxyuridine 5'-triphosphate nucleotidohydrolase	0.46	0.0120	0.0050	0.0175	0.0456	0.0185	1.2	6235	1081	5260	1028	-1133
1088	PFA0130c	serine/threonine protein kinase, FIKK family	0.49	0.0060	0.0012	0.0094	0.0286	0.0120	1.5	3155	1095	2073	1123	-1136
1089	PFI0530c	DNA primase large subunit, putative	0.49	0.0080	0.0024	0.0122	0.0345	0.0142	1.3	5444	1204	4355	1030	-1145
911	PF14_0564	conserved Plasmodium protein, unknown function	0.53	0.0060	0.0012	0.0094	0.0286	0.0120	1.4	4786	1271	3451	1238	-1173
569	PF07_0006	sporozoite threonine and asparagine-rich protein	0.62	0.0040	0.0003	0.0064	0.0219	0.0093	1.6	5461	1445	3503	1694	-1181
586	PFE0860c	conserved Plasmodium protein, unknown function	0.62	0.0060	0.0012	0.0094	0.0286	0.0120	1.5	5560	1448	3623	1681	-1192
1426	MAL8P1.126	serine protease, putative	0.43	0.0120	0.0050	0.0175	0.0456	0.0185	1.2	6354	832	5468	1247	-1193
1188	PFE0130c	conserved Plasmodium protein, unknown function	0.47	0.0100	0.0036	0.0149	0.0399	0.0162	1.4	3774	1041	2715	1218	-1199
1153	PFL2505c	conserved Plasmodium protein, unknown function	0.48	0.0060	0.0012	0.0094	0.0286	0.0120	1.2	5702	872	4614	1418	-1202
918	MAL7P1.22	conserved Plasmodium protein, unknown function	0.53	0.0100	0.0036	0.0149	0.0399	0.0162	1.2	7952	750	6570	1857	-1224
842	PFL1190c	conserved Plasmodium protein, unknown function	0.55	0.0080	0.0024	0.0122	0.0345	0.0142	1.6	4054	1572	2526	1207	-1251
446	MAL7P1.7	Plasmodium exported protein (PHISTb), unknown functio	0.66	0.0020	0.0000	0.0030	0.0145	0.0080	1.4	8359	734	5868	3012	-1255
1109	PF13_0339	conserved Plasmodium protein, unknown function	0.48	0.0080	0.0024	0.0122	0.0345	0.0142	1.3	4840	1394	3658	1051	-1263
1197	PFA0270c	conserved Plasmodium protein, unknown function	0.47	0.0100	0.0036	0.0149	0.0399	0.0162	1.3	4455	1241	3342	1140	-1269
1222	PF14_0121	conserved Plasmodium protein, unknown function	0.46	0.0120	0.0050	0.0175	0.0456	0.0185	1.8	2543	1226	1436	1160	-1279
916	PFL1840w	conserved Plasmodium membrane protein, unknown fun	0.53	0.0080	0.0024	0.0122	0.0345	0.0142	1.6	3729	1380	2264	1375	-1291
1409	PF10_0017	Plasmodium exported protein (PHISTa), unknown functio	0.43	0.0100	0.0036	0.0149	0.0399	0.0162	1.6	2705	1402	1712	917	-1325
979	MAL7P1.176	erythrocyte binding antigen-175	0.52	0.0080	0.0024	0.0122	0.0345	0.0142	1.2	7590	1105	6165	1647	-1326
1115	PFF0683c	conserved Plasmodium protein, unknown function	0.48	0.0080	0.0024	0.0122	0.0345	0.0142	1.3	5642	1589	4397	990	-1335
849	PFD1200c	Plasmodium exported protein (hyp6), unknown function	0.55	0.0100	0.0036	0.0149	0.0399	0.0162	4.2	2147	2114	505	881	-1354
1072	PFA0105w	stevor, pseudogene	0.49	0.0100	0.0036	0.0149	0.0399	0.0162	2.0	2808	1779	1407	1067	-1446
1135	PFA0210c	conserved Plasmodium protein, unknown function	0.48	0.0100	0.0036	0.0149	0.0399	0.0162	1.2	7459	1299	6037	1678	-1555
887	PF08_0072	conserved Plasmodium protein, unknown function	0.54	0.0080	0.0024	0.0122	0.0345	0.0142	1.5	5856	1718	4015	1693	-1569
995	PF10_0015	acyl-CoA binding protein, isoform 1, ACBP1	0.51	0.0040	0.0003	0.0064	0.0219	0.0093	1.2	8797	663	7089	2661	-1616
658	PF10_0170	conserved Plasmodium protein, unknown function	0.60	0.0060	0.0012	0.0094	0.0286	0.0120	2.1	5114	2295	2456	2157	-1794
937	PFL2545c	Plasmodium exported protein, unknown function	0.53	0.0120	0.0050	0.0175	0.0456	0.0185	1.5	6383	1130	4373	2684	-1804
892	PFE1285w	membrane skeletal protein IMC1-related	0.54	0.0080	0.0024	0.0122	0.0345	0.0142	1.7	4953	1630	2834	2302	-1813
Genes highly induced in yeast vs. non-yeast covered space														
2	PF11_0058		-1.25	0.0020	0.0000	0.0030	0.0145	0.0080	1.8	3316	600	5863	1438	509
5	PF14_0315	conserved Plasmodium membrane protein, unknown fun	-1.20	0.0020	0.0000	0.0030	0.0145	0.0080	2.4	2130	1264	5057	1184	479
7	PFF0690c	organic anion transporter	-1.17	0.0020	0.0000	0.0030	0.0145	0.0080	1.5	5550	1181	8077	981	366
9	PF11_0503	Plasmodium exported protein (PHISTc), unknown functio	-1.16	0.0020	0.0000	0.0030	0.0145	0.0080	1.4	6310	1301	8864	900	353
3	PF08_0012	SET domain protein, putative	-1.23	0.0020	0.0000	0.0030	0.0145	0.0080	1.5	3701	876	5595	668	351
6	PF13_0272	thioredoxin-related protein, putative	-1.19	0.0020	0.0000	0.0030	0.0145	0.0080	1.3	6589	954	8670	788	339
19	PF14_0273	rRNA (adenosine-2'-O-)-methyltransferase, putative	-1.10	0.0020	0.0000	0.0030	0.0145	0.0080	1.9	3198	1306	6125	1361	260
15	PF10_0070	conserved Plasmodium membrane protein, unknown fun	-1.12	0.0020	0.0000	0.0030	0.0145	0.0080	1.5	4617	794	6781	1140	228
10	PFL2050w	protein geranylgeranyltransferase type II, alpha subunit,	-1.16	0.0020	0.0000	0.0030	0.0145	0.0080	1.3	4676	669	6147	602	200

11	PFF0090w	conserved Plasmodium protein, unknown function	-1.16	0.0020	0.0000	0.0030	0.0145	0.0080	1.2	8137	831	9609	441	200
4	PFL2075c	conserved Plasmodium protein, unknown function	-1.21	0.0020	0.0000	0.0030	0.0145	0.0080	1.1	6318	408	7215	334	156
16	PFI1745c	early transcribed membrane protein	-1.11	0.0020	0.0000	0.0030	0.0145	0.0080	1.3	5915	400	7428	962	151
8	PFL0700w	conserved Plasmodium protein, unknown function	-1.16	0.0020	0.0000	0.0030	0.0145	0.0080	1.2	5550	512	6606	396	149
24	PFD0535w	conserved Plasmodium protein, unknown function	-1.08	0.0020	0.0000	0.0030	0.0145	0.0080	2.8	1036	481	2926	1267	142
29	PF14_0020	choline kinase	-1.07	0.0020	0.0000	0.0030	0.0145	0.0080	2.7	1273	408	3378	1563	134
21	PF14_0341	glucose-6-phosphate isomerase	-1.09	0.0020	0.0000	0.0030	0.0145	0.0080	1.2	6964	703	8573	779	126
28	PF14_0124	actin II	-1.07	0.0020	0.0000	0.0030	0.0145	0.0080	1.3	5834	878	7701	864	125
26	PFE0050w	Plasmodium exported protein, unknown function	-1.08	0.0020	0.0000	0.0030	0.0145	0.0080	1.2	7479	1098	9236	535	124
18	PFC0070c	Plasmodium exported protein, unknown function	-1.10	0.0020	0.0000	0.0030	0.0145	0.0080	1.7	1899	586	3246	640	121
27	PF11_0085		-1.07	0.0020	0.0000	0.0030	0.0145	0.0080	1.3	4922	809	6611	766	114
25	PFC0581w	co-chaperone p23	-1.08	0.0020	0.0000	0.0030	0.0145	0.0080	1.3	5103	876	6561	476	105
34	PF13_0016	methyltransferase-like protein, putative	-1.04	0.0020	0.0000	0.0030	0.0145	0.0080	3.1	1304	698	4044	1941	100
22	PFB0080c	Plasmodium exported protein (PHISTb), unknown functio	-1.08	0.0020	0.0000	0.0030	0.0145	0.0080	1.2	7894	540	9173	639	100
31	PF10_0317	DER1-like protein, putative	-1.06	0.0020	0.0000	0.0030	0.0145	0.0080	1.5	3370	640	4954	860	83
20	PF08_0138	rifin	-1.09	0.0020	0.0000	0.0030	0.0145	0.0080	2.6	602	254	1562	627	78
40	PFL1745c	clustered-asparagine-rich protein	-1.02	0.0020	0.0000	0.0030	0.0145	0.0080	1.6	5021	1574	8099	1453	51
35	PF13_0319	conserved Plasmodium protein, unknown function	-1.03	0.0020	0.0000	0.0030	0.0145	0.0080	1.3	5037	947	6577	548	46
36	PFC0210c	circumsporozoite (CS) protein	-1.03	0.0020	0.0000	0.0030	0.0145	0.0080	1.9	1779	689	3327	818	41
30	PFF0695w	conserved Plasmodium protein, unknown function	-1.06	0.0020	0.0000	0.0030	0.0145	0.0080	1.1	8343	237	8928	313	34
37	PFA0160c	nucleoside transporter, putative	-1.02	0.0020	0.0000	0.0030	0.0145	0.0080	1.2	7116	1027	8591	415	34
41	PFC1030w	conserved Plasmodium protein, unknown function	-1.01	0.0020	0.0000	0.0030	0.0145	0.0080	1.8	2630	582	4632	1394	26
39	PFL0435w	conserved Plasmodium protein, unknown function	-1.02	0.0020	0.0000	0.0030	0.0145	0.0080	1.5	2702	645	3970	599	24
42	PF07_0115	cation transporting ATPase, putative	-1.01	0.0020	0.0000	0.0030	0.0145	0.0080	1.5	3095	712	4564	744	13
43	PF13_0281	conserved Plasmodium protein, unknown function	-1.00	0.0020	0.0000	0.0030	0.0145	0.0080	1.3	3390	379	4522	747	5
Genes induced in yeast vs. non-yeast covered space														
45	PF14_0036	phosphatase, putative	-1.00	0.0020	0.0000	0.0030	0.0145	0.0080	1.8	2974	939	5466	1561	-8
46	PF13_0287	adenylosuccinate synthetase	-0.99	0.0020	0.0000	0.0030	0.0145	0.0080	1.2	6777	743	7896	386	-11
104	PF14_0448	40S ribosomal protein S2, putative	-0.89	0.0020	0.0000	0.0030	0.0145	0.0080	1.0	9710	74	9807	34	-12
311	PF13_0049	60S ribosomal protein L24, putative	-0.73	0.0020	0.0000	0.0030	0.0145	0.0080	1.0	9854	48	9907	24	-20
194	PF13_0228	40S ribosomal protein S6, putative	-0.80	0.0020	0.0000	0.0030	0.0145	0.0080	1.0	9767	52	9848	50	-20
49	PFE0840c	transcription factor with AP2 domain(s), putative	-0.98	0.0020	0.0000	0.0030	0.0145	0.0080	1.5	2084	519	3212	632	-24
187	PF13_0275	Plasmodium exported protein, unknown function	-0.81	0.0020	0.0000	0.0030	0.0145	0.0080	1.0	9800	86	9912	51	-26
534	PF13_0116	rhoptry protein 2, putative	-0.64	0.0020	0.0000	0.0030	0.0145	0.0080	1.0	9746	41	9796	38	-29
722	PFE1005w	40S ribosomal protein S9, putative	-0.58	0.0080	0.0024	0.0122	0.0345	0.0142	1.0	9877	40	9917	29	-29
529	PF14_0141	60S ribosomal protein L10, putative	-0.64	0.0040	0.0003	0.0064	0.0219	0.0093	1.0	9873	59	9926	24	-30
52	PF14_0304	conserved Plasmodium protein, unknown function	-0.97	0.0020	0.0000	0.0030	0.0145	0.0080	2.3	880	309	2000	843	-31
50	PFA0090c	stevor	-0.98	0.0020	0.0000	0.0030	0.0145	0.0080	2.7	850	454	2305	1034	-33
417	PF10_0043	60S ribosomal protein L13, putative	-0.67	0.0040	0.0003	0.0064	0.0219	0.0093	1.0	9728	59	9798	46	-34
48	PF11_0210	metal ion channel - Mg2 , Co2 and Ni2	-0.98	0.0020	0.0000	0.0030	0.0145	0.0080	1.5	3457	791	5308	1098	-38
626	PF08_0019	receptor for activated c kinase	-0.61	0.0020	0.0000	0.0030	0.0145	0.0080	1.0	9814	67	9878	38	-41
119	PFL2515c	conserved Plasmodium protein, unknown function	-0.88	0.0020	0.0000	0.0030	0.0145	0.0080	1.0	9608	232	9909	111	-42
67	PFL1315w	potassium channel protein	-0.95	0.0020	0.0000	0.0030	0.0145	0.0080	1.1	6180	455	6937	346	-43
994	PFD1055w	40S ribosomal protein S19, putative	-0.51	0.0060	0.0012	0.0094	0.0286	0.0120	1.0	9732	45	9778	46	-44
91	MAL13P1.23:	Sec61 alpha subunit, PfSec61	-0.91	0.0020	0.0000	0.0030	0.0145	0.0080	1.1	8422	216	8886	293	-45
468	PFI0645w	elongation factor 1-beta	-0.66	0.0020	0.0000	0.0030	0.0145	0.0080	1.0	9696	89	9782	41	-45
54	PF13_0241	rhomboid protease ROM6, putative	-0.97	0.0020	0.0000	0.0030	0.0145	0.0080	1.4	3558	523	5024	988	-45

464	PF14_0230	60S ribosomal protein L5, putative	-0.66	0.0020	0.0000	0.0030	0.0145	0.0080	1.0	9684	84	9775	54	-47
970	MAL7P1.320	ribosomal protein, L37e, putative	-0.52	0.0080	0.0024	0.0122	0.0345	0.0142	1.0	9715	62	9770	44	-51
945	PF11_0313	60S ribosomal protein P0	-0.53	0.0080	0.0024	0.0122	0.0345	0.0142	1.0	9889	74	9947	36	-52
1185	PFL2335w	conserved Plasmodium protein, unknown function	-0.47	0.0100	0.0036	0.0149	0.0399	0.0162	1.0	9558	60	9608	46	-56
76	PF11_0508	Plasmodium exported protein, unknown function	-0.93	0.0020	0.0000	0.0030	0.0145	0.0080	1.1	8912	602	9666	208	-56
62	MAL13P1.17(	nucleotidyltransferase, putative	-0.95	0.0020	0.0000	0.0030	0.0145	0.0080	1.2	6256	585	7409	625	-57
307	PF11_0517	rifin	-0.73	0.0020	0.0000	0.0030	0.0145	0.0080	2.3	130	61	301	174	-64
180	PFF1025c	SNO glutamine amidotransferase family protein	-0.81	0.0020	0.0000	0.0030	0.0145	0.0080	1.0	9274	194	9570	169	-67
66	MAL7P1.94	prefoldin subunit 3, putative	-0.95	0.0020	0.0000	0.0030	0.0145	0.0080	1.2	6182	650	7502	739	-70
57	PFB1035w	rifin	-0.96	0.0020	0.0000	0.0030	0.0145	0.0080	4.9	528	469	2581	1658	-75
86	PF10_0121	hypoxanthine phosphoribosyltransferase	-0.91	0.0020	0.0000	0.0030	0.0145	0.0080	1.1	8991	724	9813	176	-78
818	PF07_0079	60S ribosomal protein L11a, putative	-0.56	0.0080	0.0024	0.0122	0.0345	0.0142	1.0	9721	115	9823	68	-81
58	PF11_0310	transporter, putative	-0.96	0.0020	0.0000	0.0030	0.0145	0.0080	1.4	5658	930	7668	1161	-82
55	PFE0595w	prefoldin subunit, putative	-0.97	0.0020	0.0000	0.0030	0.0145	0.0080	1.7	3834	1196	6453	1508	-86
443	MAL13P1.61	Plasmodium exported protein (hyp8), unknown function	-0.67	0.0020	0.0000	0.0030	0.0145	0.0080	1.0	9654	143	9832	124	-89
84	PFF0610c	PP-loop family protein, putative	-0.92	0.0020	0.0000	0.0030	0.0145	0.0080	1.2	4747	536	5762	569	-90
61	PFF0825c	mitochondrial import receptor subunit tom40, putative	-0.95	0.0020	0.0000	0.0030	0.0145	0.0080	1.5	3891	898	5740	1042	-91
63	PFA0065w	Pfmc-2TM Maurer's cleft two transmembrane protein	-0.95	0.0020	0.0000	0.0030	0.0145	0.0080	5.2	427	276	2214	1602	-91
162	PF10_0203	ADP-ribosylation factor	-0.83	0.0020	0.0000	0.0030	0.0145	0.0080	1.0	8901	339	9345	196	-91
286	PF14_0078	plasmepsin III,histo-aspartic protease	-0.74	0.0020	0.0000	0.0030	0.0145	0.0080	1.0	9258	195	9524	164	-93
835	PF14_0231	60S ribosomal protein L7-3, putative	-0.55	0.0040	0.0003	0.0064	0.0219	0.0093	1.0	9558	122	9679	96	-98
77	PFL2470c	conserved Plasmodium protein, unknown function	-0.93	0.0020	0.0000	0.0030	0.0145	0.0080	1.5	2489	700	3757	667	-99
166	PFF0835w	conserved Plasmodium protein, unknown function	-0.83	0.0020	0.0000	0.0030	0.0145	0.0080	1.1	7858	305	8345	283	-101
591	PF13_0005	rifin	-0.62	0.0020	0.0000	0.0030	0.0145	0.0080	2.9	85	33	249	232	-101
82	PF14_0712	conserved Plasmodium protein, unknown function	-0.92	0.0020	0.0000	0.0030	0.0145	0.0080	1.5	2635	579	3846	734	-102
1126	PF13_0171	60S ribosomal protein L23, putative	-0.48	0.0100	0.0036	0.0149	0.0399	0.0162	1.0	9582	115	9678	84	-103
458	PFI0165c	DEAD/DEAH box helicase, putative	-0.66	0.0040	0.0003	0.0064	0.0219	0.0093	1.0	8937	142	9142	167	-104
805	PFE0850c	60S ribosomal protein L12, putative	-0.56	0.0060	0.0012	0.0094	0.0286	0.0120	1.0	9474	124	9608	115	-106
1071	PF14_0360	eukaryotic translation initiation factor eIF2A, putative	-0.49	0.0040	0.0003	0.0064	0.0219	0.0093	1.0	9347	106	9452	106	-108
79	PF14_0454	conserved Plasmodium protein, unknown function	-0.93	0.0020	0.0000	0.0030	0.0145	0.0080	1.6	2124	492	3499	992	-110
78	PF13_0101	conserved Plasmodium protein, unknown function	-0.93	0.0020	0.0000	0.0030	0.0145	0.0080	1.4	3299	480	4748	1083	-115
309	PFI0500w	conserved Plasmodium protein, unknown function	-0.73	0.0020	0.0000	0.0030	0.0145	0.0080	1.0	6658	228	6971	202	-117
97	PF11_0339	glycine cleavage H protein	-0.90	0.0020	0.0000	0.0030	0.0145	0.0080	2.8	577	167	1640	1016	-121
72	PFE0325w		-0.94	0.0020	0.0000	0.0030	0.0145	0.0080	1.8	2212	880	4075	1104	-122
59	PFL1070c	endoplasmin homolog precursor, putative	-0.96	0.0020	0.0000	0.0030	0.0145	0.0080	2.5	2096	1094	5153	2087	-124
337	PF14_0228	conserved Plasmodium protein, unknown function	-0.72	0.0020	0.0000	0.0030	0.0145	0.0080	1.0	8833	307	9163	156	-132
429	PF14_0649	conserved Plasmodium protein, unknown function	-0.67	0.0020	0.0000	0.0030	0.0145	0.0080	1.0	9156	263	9427	140	-133
393	PF13_0208	exoribonuclease, putative	-0.68	0.0020	0.0000	0.0030	0.0145	0.0080	1.0	7721	267	8011	156	-134
94	PFE0645w	conserved Plasmodium protein, unknown function	-0.90	0.0020	0.0000	0.0030	0.0145	0.0080	1.4	3474	528	4735	869	-136
298	MAL8P1.205	Plasmodium exported protein, unknown function	-0.73	0.0040	0.0003	0.0064	0.0219	0.0093	1.8	455	145	832	369	-138
389	PFI0875w	heat shock protein 70	-0.69	0.0020	0.0000	0.0030	0.0145	0.0080	1.0	9363	264	9663	173	-138
83	MAL8P1.62	conserved Plasmodium protein, unknown function	-0.92	0.0020	0.0000	0.0030	0.0145	0.0080	1.3	5037	979	6677	803	-141
1205	PF14_0261	proliferation-associated protein 2g4, putative	-0.47	0.0080	0.0024	0.0122	0.0345	0.0142	1.0	9374	96	9497	168	-141
112	PFA0680c	Pfmc-2TM Maurer's cleft two transmembrane protein	-0.89	0.0020	0.0000	0.0030	0.0145	0.0080	2.9	605	226	1735	1045	-142
841	PF11_0409	conserved Plasmodium protein, unknown function	-0.55	0.0080	0.0024	0.0122	0.0345	0.0142	1.0	8929	143	9103	174	-143
71	PFF0435w	ornithine aminotransferase	-0.94	0.0020	0.0000	0.0030	0.0145	0.0080	1.5	4181	888	6407	1481	-143
68	PF11_0516	stevor	-0.94	0.0020	0.0000	0.0030	0.0145	0.0080	2.5	1593	1040	3969	1479	-143

709	PF08_0079	translation initiation factor SUI1, putative	-0.58	0.0020	0.0000	0.0030	0.0145	0.0080	1.0	8624	168	8826	177	-143
133	PFB0450w	secretory complex protein 61 gamma subunit	-0.86	0.0020	0.0000	0.0030	0.0145	0.0080	1.1	7374	459	8267	577	-143
223	PFD0675w	apicoplast ribosomal protein L10 precursor, putative	-0.78	0.0020	0.0000	0.0030	0.0145	0.0080	1.5	939	210	1449	444	-144
144	MAL13P1.53	rifin	-0.85	0.0020	0.0000	0.0030	0.0145	0.0080	3.2	363	192	1180	769	-144
92	PF11_0285	conserved Plasmodium protein, unknown function	-0.91	0.0020	0.0000	0.0030	0.0145	0.0080	1.7	2183	572	3605	995	-145
578	PF13_0215	conserved Plasmodium protein, unknown function	-0.62	0.0020	0.0000	0.0030	0.0145	0.0080	1.0	9082	194	9324	196	-148
121	PFI0430c	conserved Plasmodium protein, unknown function	-0.88	0.0020	0.0000	0.0030	0.0145	0.0080	1.3	3515	647	4580	567	-149
478	PF11_0208	phosphoglycerate mutase, putative	-0.65	0.0020	0.0000	0.0030	0.0145	0.0080	1.0	9444	308	9729	128	-151
465	PF07_0053	conserved Plasmodium protein, unknown function	-0.66	0.0020	0.0000	0.0030	0.0145	0.0080	1.0	8756	254	9049	192	-153
75	PF11_0309	conserved Plasmodium protein, unknown function	-0.93	0.0020	0.0000	0.0030	0.0145	0.0080	1.5	4049	1224	6197	1084	-160
184	PF13_0048	NUDIX hydrolase, putative	-0.81	0.0020	0.0000	0.0030	0.0145	0.0080	1.1	8534	559	9227	296	-162
1289	PF10_0155	enolase	-0.45	0.0100	0.0036	0.0149	0.0399	0.0162	1.0	9487	140	9623	164	-167
948	PFF1435w	conserved Plasmodium protein, unknown function	-0.52	0.0040	0.0003	0.0064	0.0219	0.0093	1.0	8232	216	8417	137	-168
208	PFI1680w	UBX domain, putative	-0.79	0.0020	0.0000	0.0030	0.0145	0.0080	1.1	7148	456	7775	338	-168
153	PF13_0033	26S proteasome regulatory subunit, putative	-0.84	0.0020	0.0000	0.0030	0.0145	0.0080	1.1	7281	589	8158	457	-169
814	PF13_0062	clathrin-adaptor medium chain, putative	-0.56	0.0060	0.0012	0.0094	0.0286	0.0120	1.0	9209	195	9421	186	-169
110	PFL0595c	glutathione peroxidase	-0.89	0.0020	0.0000	0.0030	0.0145	0.0080	1.5	2539	562	3905	976	-171
134	PF13_0054	conserved Plasmodium protein, unknown function	-0.86	0.0020	0.0000	0.0030	0.0145	0.0080	1.5	2279	407	3381	874	-178
117	MAL13P1.440		-0.88	0.0020	0.0000	0.0030	0.0145	0.0080	1.6	2172	643	3522	885	-178
320	PFB0050c	stevor, pseudogene	-0.72	0.0040	0.0003	0.0064	0.0219	0.0093	3.2	223	136	706	532	-185
209	PFC0911c	conserved Plasmodium protein, unknown function	-0.79	0.0020	0.0000	0.0030	0.0145	0.0080	1.6	1233	398	1924	479	-186
70	PF13_0073	Plasmodium exported protein (hyp12), unknown functior	-0.94	0.0020	0.0000	0.0030	0.0145	0.0080	1.5	5723	1872	8666	1258	-186
301	PFD0810w	small GTP-binding protein sar1	-0.73	0.0040	0.0003	0.0064	0.0219	0.0093	1.1	8817	467	9323	227	-187
98	PF13_0099	conserved Plasmodium protein, unknown function	-0.90	0.0020	0.0000	0.0030	0.0145	0.0080	1.4	4312	656	5973	1195	-190
343	PF13_0280	ER lumen protein retaining receptor	-0.71	0.0020	0.0000	0.0030	0.0145	0.0080	1.1	8368	277	8837	383	-191
912	PF13_0224	60S ribosomal protein L18, putative	-0.53	0.0080	0.0024	0.0122	0.0345	0.0142	1.0	9171	225	9389	186	-192
131	PF14_0090	DNA-damage inducible protein, putative	-0.86	0.0020	0.0000	0.0030	0.0145	0.0080	2.2	1046	402	2264	1011	-194
103	MAL13P1.25	phosphatidylinositol transfer protein, putative	-0.89	0.0020	0.0000	0.0030	0.0145	0.0080	1.3	5580	901	7210	923	-195
174	PFC0035w	rifin	-0.82	0.0020	0.0000	0.0030	0.0145	0.0080	4.3	269	130	1159	953	-195
81	MAL13P1.15	conserved Plasmodium protein, unknown function	-0.92	0.0020	0.0000	0.0030	0.0145	0.0080	2.7	1357	566	3704	1980	-198
143	PF14_0377	vesicle-associated membrane protein, putative	-0.85	0.0020	0.0000	0.0030	0.0145	0.0080	1.2	6288	584	7440	771	-202
130	PF07_0130	stevor	-0.87	0.0020	0.0000	0.0030	0.0145	0.0080	2.7	784	543	2090	966	-203
109	PFE0790c	BolA-like protein, putative	-0.89	0.0020	0.0000	0.0030	0.0145	0.0080	1.3	4733	1142	6370	698	-203
886	PF11_0106	apicoplast ribosomal protein L36e precursor, putative	-0.54	0.0060	0.0012	0.0094	0.0286	0.0120	1.0	8614	250	8856	198	-206
141	PFI1265w	conserved Plasmodium protein, unknown function	-0.85	0.0020	0.0000	0.0030	0.0145	0.0080	1.8	1472	397	2673	1013	-210
280	MAL8P1.142	20S proteasome beta subunit	-0.74	0.0020	0.0000	0.0030	0.0145	0.0080	1.1	8485	408	9094	413	-211
138	PFF1235w	conserved Plasmodium membrane protein, unknown fun	-0.85	0.0020	0.0000	0.0030	0.0145	0.0080	1.4	2940	657	4188	803	-212
201	PF08_0011	leucine-tRNA ligase	-0.79	0.0020	0.0000	0.0030	0.0145	0.0080	1.3	3233	360	4046	665	-212
471	PF07_0090a	conserved Plasmodium protein, unknown function	-0.66	0.0040	0.0003	0.0064	0.0219	0.0093	1.1	7329	230	7733	386	-213
160	PF07_0007	conserved Plasmodium protein, unknown function	-0.83	0.0020	0.0000	0.0030	0.0145	0.0080	1.1	8395	689	9446	576	-214
694	PFI0755c	6-phosphofructokinase	-0.59	0.0040	0.0003	0.0064	0.0219	0.0093	1.0	8942	326	9253	203	-218
118	PFA0435w	conserved Plasmodium protein, unknown function	-0.88	0.0020	0.0000	0.0030	0.0145	0.0080	1.3	5085	901	6699	933	-220
507	PF10_0366	ADP/ATP transporter on adenylate translocase	-0.64	0.0020	0.0000	0.0030	0.0145	0.0080	1.0	9076	366	9479	258	-222
313	PFE0500c	conserved Plasmodium protein, unknown function	-0.73	0.0020	0.0000	0.0030	0.0145	0.0080	1.8	785	185	1374	626	-222
157	PF13_0065	vacuolar ATP synthase subunit a	-0.83	0.0020	0.0000	0.0030	0.0145	0.0080	1.2	7334	880	8458	467	-223
128	MAL13P1.23	conserved Plasmodium protein, unknown function	-0.87	0.0020	0.0000	0.0030	0.0145	0.0080	1.2	5931	822	7387	859	-226
1059	PF14_0205	40S ribosomal protein S25, putative	-0.49	0.0060	0.0012	0.0094	0.0286	0.0120	1.0	9137	243	9358	204	-226

1028	PF13_0014	40S ribosomal protein S7, putative	-0.50	0.0100	0.0036	0.0149	0.0399	0.0162	1.0	8809	195	9042	266	-229
380	PFI0070w	rifin	-0.69	0.0020	0.0000	0.0030	0.0145	0.0080	3.8	182	124	690	613	-229
165	PFF1565c	rifin	-0.83	0.0020	0.0000	0.0030	0.0145	0.0080	2.3	871	327	1988	1021	-231
264	PF13_0331	conserved protein, unknown function	-0.75	0.0020	0.0000	0.0030	0.0145	0.0080	1.1	5990	380	6694	555	-231
152	PFE0915c	proteasome subunit beta type 1, putative	-0.84	0.0020	0.0000	0.0030	0.0145	0.0080	1.2	7357	947	8594	525	-236
656	PFI0935w	DnaJ protein, putative	-0.60	0.0020	0.0000	0.0030	0.0145	0.0080	1.0	8608	287	8960	300	-236
778	PF14_0728	conserved Plasmodium protein, unknown function	-0.57	0.0040	0.0003	0.0064	0.0219	0.0093	1.0	8760	305	9072	243	-237
602	PF14_0644	conserved Plasmodium protein, unknown function	-0.61	0.0020	0.0000	0.0030	0.0145	0.0080	4.6	105	49	483	567	-238
283	MAL13P1.335	phosphatidylserine synthase I, putative	-0.74	0.0020	0.0000	0.0030	0.0145	0.0080	1.1	6975	275	7656	644	-238
289	MAL8P1.219	rifin	-0.74	0.0020	0.0000	0.0030	0.0145	0.0080	3.0	338	155	1003	748	-239
663	PFL0670c	bifunctional aminoacyl-tRNA synthetase, putative	-0.60	0.0020	0.0000	0.0030	0.0145	0.0080	1.0	7706	281	8060	313	-240
161	PF11_0440	conserved Plasmodium protein, unknown function	-0.83	0.0040	0.0003	0.0064	0.0219	0.0093	1.7	1755	513	2937	911	-241
89	PF10_0310	conserved Plasmodium protein, unknown function	-0.91	0.0020	0.0000	0.0030	0.0145	0.0080	2.1	2334	1263	4862	1508	-243
123	PFC0270w	activator of Hsp90 ATPase, putative	-0.87	0.0020	0.0000	0.0030	0.0145	0.0080	1.6	3020	957	4717	983	-243
340	PFD0730w	conserved Plasmodium protein, unknown function	-0.71	0.0020	0.0000	0.0030	0.0145	0.0080	1.1	7245	463	7855	391	-245
150	MAL8P1.330		-0.84	0.0020	0.0000	0.0030	0.0145	0.0080	2.1	1216	485	2530	1074	-245
373	PFC0925w	conserved Plasmodium protein, unknown function	-0.69	0.0020	0.0000	0.0030	0.0145	0.0080	1.1	8000	411	8557	394	-247
1227	PF14_0543	signal peptide peptidase	-0.46	0.0060	0.0012	0.0094	0.0286	0.0120	1.0	9363	259	9576	202	-247
159	PF14_0790	conserved Plasmodium protein, unknown function	-0.83	0.0020	0.0000	0.0030	0.0145	0.0080	1.2	5334	590	6563	887	-249
326	PFL1720w	serine hydroxymethyltransferase	-0.72	0.0020	0.0000	0.0030	0.0145	0.0080	1.6	996	215	1642	681	-250
362	PF07_0093	conserved Plasmodium protein, unknown function	-0.70	0.0020	0.0000	0.0030	0.0145	0.0080	1.1	6736	392	7323	448	-253
452	PFI1665w	transcription factor with AP2 domain(s), putative	-0.66	0.0020	0.0000	0.0030	0.0145	0.0080	1.8	660	213	1160	541	-253
454	PF14_0064	phosphatase, putative	-0.66	0.0020	0.0000	0.0030	0.0145	0.0080	2.6	328	134	843	643	-262
367	PF11_0020	rifin	-0.70	0.0020	0.0000	0.0030	0.0145	0.0080	2.9	325	241	944	646	-268
279	MAL13P1.267	conserved Plasmodium protein, unknown function	-0.74	0.0020	0.0000	0.0030	0.0145	0.0080	1.3	2722	369	3507	687	-271
214	PF11_0198	tRNA m(1)G methyltransferase, putative	-0.78	0.0020	0.0000	0.0030	0.0145	0.0080	1.1	7295	768	8285	495	-273
512	MAL8P1.17	protein disulfide isomerase	-0.64	0.0020	0.0000	0.0030	0.0145	0.0080	1.1	9073	480	9566	287	-274
198	PF13_0221	conserved Plasmodium protein, unknown function	-0.80	0.0020	0.0000	0.0030	0.0145	0.0080	1.7	1547	531	2618	815	-275
164	PFD0795w	histone acetyltransferase, putative	-0.83	0.0020	0.0000	0.0030	0.0145	0.0080	1.4	3252	754	4589	858	-276
105	PFE1605w	Plasmodium exported protein (PHISTb), unknown functio	-0.89	0.0020	0.0000	0.0030	0.0145	0.0080	1.4	6238	1229	8543	1353	-277
423	PFE0245c	conserved Plasmodium membrane protein, unknown fun	-0.67	0.0020	0.0000	0.0030	0.0145	0.0080	1.1	7727	413	8296	435	-278
399	PF08_0089	conserved Plasmodium protein, unknown function	-0.68	0.0020	0.0000	0.0030	0.0145	0.0080	1.1	7537	526	8129	345	-279
195	PFD0990w	ribosome recycling factor, putative	-0.80	0.0020	0.0000	0.0030	0.0145	0.0080	1.3	3321	606	4441	793	-279
243	PF08_0117	AAA family ATPase, putative	-0.76	0.0020	0.0000	0.0030	0.0145	0.0080	1.2	4082	527	4981	651	-280
331	PFF1590w	rifin	-0.72	0.0020	0.0000	0.0030	0.0145	0.0080	2.3	549	235	1271	770	-283
526	PF11_0121	acylphosphatase, putative	-0.64	0.0020	0.0000	0.0030	0.0145	0.0080	1.1	5895	430	6402	363	-286
1134	PF08_0087	karyopherin alpha	-0.48	0.0120	0.0050	0.0175	0.0456	0.0185	1.0	8342	286	8604	262	-286
679	PFF1550w	stevor	-0.59	0.0040	0.0003	0.0064	0.0219	0.0093	2.3	331	181	749	525	-288
168	PF14_0058	conserved Plasmodium protein, unknown function	-0.83	0.0020	0.0000	0.0030	0.0145	0.0080	1.3	4835	872	6203	784	-288
381	PFD0275w	conserved Plasmodium membrane protein, unknown fun	-0.69	0.0020	0.0000	0.0030	0.0145	0.0080	1.1	6521	525	7158	400	-289
323	PF11_0062	histone H2B	-0.72	0.0020	0.0000	0.0030	0.0145	0.0080	1.1	8498	587	9260	467	-293
461	PF13_0243	conserved Plasmodium protein, unknown function	-0.66	0.0020	0.0000	0.0030	0.0145	0.0080	1.1	7799	485	8373	384	-295
220	PF08_0131	1-cys peroxiredoxin	-0.78	0.0020	0.0000	0.0030	0.0145	0.0080	1.2	5896	584	6959	776	-297
218	MAL8P1.77	conserved Plasmodium protein, unknown function	-0.78	0.0020	0.0000	0.0030	0.0145	0.0080	1.8	1300	391	2368	975	-298
155	PFL1525c	pre-mRNA splicing factor RNA helicase, putative	-0.84	0.0020	0.0000	0.0030	0.0145	0.0080	1.5	2787	744	4318	1087	-299
1321	PFF1155w	hexokinase	-0.44	0.0120	0.0050	0.0175	0.0456	0.0185	1.0	8629	241	8869	298	-299
695	PF07_0070	drug metabolite transporter, putative	-0.59	0.0060	0.0012	0.0094	0.0286	0.0120	1.1	7659	389	8088	341	-301

175	PF11_0101	conserved Plasmodium protein, unknown function	-0.82	0.0020	0.0000	0.0030	0.0145	0.0080	1.6	2265	681	3640	996	-301
504	PFF0400w	conserved Plasmodium protein, unknown function	-0.65	0.0020	0.0000	0.0030	0.0145	0.0080	1.1	6616	518	7174	346	-306
281	PFA0620c	glutamic acid-rich protein	-0.74	0.0020	0.0000	0.0030	0.0145	0.0080	1.1	8624	823	9508	368	-308
587	MAL8P1.103	conserved Plasmodium protein, unknown function	-0.62	0.0020	0.0000	0.0030	0.0145	0.0080	1.1	8271	344	8772	466	-309
355	PFD0280w		-0.70	0.0020	0.0000	0.0030	0.0145	0.0080	1.1	7030	620	7761	420	-309
404	PFI1281w	conserved Plasmodium protein, unknown function	-0.68	0.0040	0.0003	0.0064	0.0219	0.0093	1.1	8239	635	8895	331	-310
773	PFI0995w	conserved Plasmodium protein, unknown function	-0.57	0.0040	0.0003	0.0064	0.0219	0.0093	1.9	482	146	894	576	-311
136	PF14_0279	conserved Plasmodium protein, unknown function	-0.86	0.0020	0.0000	0.0030	0.0145	0.0080	1.4	4846	1307	6695	854	-313
353	PF14_0300	syntaxin, Qa-SNARE family	-0.70	0.0020	0.0000	0.0030	0.0145	0.0080	1.2	3998	354	4747	711	-315
146	PFI1750c	Plasmodium exported protein (hyp11), unknown functior	-0.85	0.0020	0.0000	0.0030	0.0145	0.0080	1.9	1940	638	3723	1464	-319
314	PFA0300c	vacuolar ATP synthase subunit c, putative	-0.73	0.0020	0.0000	0.0030	0.0145	0.0080	1.1	6716	779	7561	386	-320
282	PFL0485w	conserved Plasmodium protein, unknown function	-0.74	0.0020	0.0000	0.0030	0.0145	0.0080	1.2	5764	702	6682	536	-320
840	PF11_0246	conserved Plasmodium protein, unknown function	-0.55	0.0080	0.0024	0.0122	0.0345	0.0142	1.1	7766	269	8160	446	-321
96	PFE0630c	orotate phosphoribosyltransferase	-0.90	0.0020	0.0000	0.0030	0.0145	0.0080	2.3	2137	1413	4973	1747	-323
582	PFI0420c	tRNA pseudouridine synthase, putative	-0.62	0.0020	0.0000	0.0030	0.0145	0.0080	1.1	6202	443	6730	410	-324
971	PFB0770c	conserved Plasmodium membrane protein, unknown fun	-0.52	0.0100	0.0036	0.0149	0.0399	0.0162	1.3	1058	271	1408	405	-324
145	PFD1035w	steroid dehydrogenase, putative	-0.85	0.0020	0.0000	0.0030	0.0145	0.0080	2.1	1738	891	3569	1267	-327
409	PFB0090c	RESA-like protein with PHIST and DnaJ domains	-0.68	0.0020	0.0000	0.0030	0.0145	0.0080	1.1	8139	485	8825	528	-327
833	PFI0630w	26S proteasome regulatory subunit, putative	-0.55	0.0080	0.0024	0.0122	0.0345	0.0142	1.0	8596	392	9003	345	-330
557	PFB0260w	proteasome 26S regulatory subunit, putative	-0.63	0.0020	0.0000	0.0030	0.0145	0.0080	1.1	7391	519	7953	375	-333
250	MAL13P1.34	conserved Plasmodium protein, unknown function	-0.76	0.0020	0.0000	0.0030	0.0145	0.0080	1.2	5699	759	6751	626	-333
142	PFB0685c	acyl-CoA synthetase, PfACS9	-0.85	0.0020	0.0000	0.0030	0.0145	0.0080	1.9	2179	525	4097	1728	-335
232	PF14_0719	conserved Plasmodium protein, unknown function	-0.77	0.0020	0.0000	0.0030	0.0145	0.0080	1.1	7558	864	8685	600	-336
288	MAL7P1.86	transcription initiation factor IIE, alpha subunit, putative	-0.74	0.0020	0.0000	0.0030	0.0145	0.0080	1.2	5961	574	6907	709	-337
258	PF14_0028	pre-mRNA splicing factor, putative	-0.76	0.0020	0.0000	0.0030	0.0145	0.0080	1.1	7008	756	8058	633	-338
287	MAL13P1.26	conserved Plasmodium protein, unknown function	-0.74	0.0020	0.0000	0.0030	0.0145	0.0080	2.2	803	361	1761	937	-340
163	PF14_0266	conserved protein, unknown function	-0.83	0.0020	0.0000	0.0030	0.0145	0.0080	1.6	3012	671	4671	1330	-341
211	PF14_0412	conserved Plasmodium protein, unknown function	-0.79	0.0020	0.0000	0.0030	0.0145	0.0080	1.9	1371	430	2634	1174	-341
226	PFD1120c	early transcribed membrane protein 4	-0.78	0.0020	0.0000	0.0030	0.0145	0.0080	1.2	6706	587	7914	963	-342
188	PFL2275c	FK506-binding protein (FKBP)-type peptidyl-propyl isome	-0.81	0.0020	0.0000	0.0030	0.0145	0.0080	1.2	6113	963	7575	846	-346
172	PFE0250w	conserved Plasmodium protein, unknown function	-0.82	0.0020	0.0000	0.0030	0.0145	0.0080	1.4	4015	799	5605	1138	-347
170	PF11_0188	heat shock protein 90, putative	-0.82	0.0020	0.0000	0.0030	0.0145	0.0080	1.4	3967	858	5580	1104	-349
305	MAL13P1.44	protein phosphatase 2c-like protein, putative	-0.73	0.0020	0.0000	0.0030	0.0145	0.0080	1.1	7602	775	8543	516	-350
203	PF10_0211	conserved Plasmodium membrane protein, unknown fun	-0.79	0.0020	0.0000	0.0030	0.0145	0.0080	1.3	3792	714	5115	960	-351
354	PF14_0705	conserved Plasmodium protein, unknown function	-0.70	0.0020	0.0000	0.0030	0.0145	0.0080	1.4	2130	571	2963	613	-351
267	PF14_0022	conserved Plasmodium protein, unknown function	-0.75	0.0040	0.0003	0.0064	0.0219	0.0093	1.3	3818	804	4881	610	-353
182	MAL13P1.28	pyrroline carboxylate reductase	-0.81	0.0020	0.0000	0.0030	0.0145	0.0080	1.3	4402	591	5931	1291	-353
271	PF13_0344	ubiquitin-activating enzyme	-0.75	0.0040	0.0003	0.0064	0.0219	0.0093	1.2	6221	656	7278	754	-354
224	PF14_0606	mitochondrial ribosomal protein S6-2 precursor, putative	-0.78	0.0020	0.0000	0.0030	0.0145	0.0080	1.4	2793	740	4047	869	-354
696	PF13_0350	signal recognition particle receptor alpha subunit	-0.59	0.0020	0.0000	0.0030	0.0145	0.0080	1.1	7865	526	8370	335	-356
449	PFF0620c	6-cysteine protein	-0.66	0.0020	0.0000	0.0030	0.0145	0.0080	1.1	6031	452	6737	610	-356
804	PF14_0478	DNA polymerase delta interacting protein, putative	-0.56	0.0020	0.0000	0.0030	0.0145	0.0080	1.1	8020	503	8474	308	-358
546	MAL13P1.25	conserved Plasmodium protein, unknown function	-0.63	0.0060	0.0012	0.0094	0.0286	0.0120	1.1	6237	433	6854	544	-359
237	PF10_0051	ADP/ATP carrier protein, putative	-0.77	0.0020	0.0000	0.0030	0.0145	0.0080	1.9	1285	407	2466	1135	-362
87	PF14_0730	Plasmodium exported protein (PHISTb), unknown functio	-0.91	0.0020	0.0000	0.0030	0.0145	0.0080	2.2	3278	1710	7085	2459	-362
510	PFI1530c	conserved Plasmodium protein, unknown function	-0.64	0.0040	0.0003	0.0064	0.0219	0.0093	1.1	5825	547	6482	473	-363
339	PF13_0227	vacuolar ATP synthase subunit d, putative	-0.71	0.0020	0.0000	0.0030	0.0145	0.0080	1.1	6981	683	7895	598	-367

310	PF14_0328	mitochondrial import inner membrane translocase subun	-0.73	0.0020	0.0000	0.0030	0.0145	0.0080	1.4	2356	490	3334	855	-367
799	PF14_0301	conserved protein, unknown function	-0.56	0.0060	0.0012	0.0094	0.0286	0.0120	1.1	6986	491	7458	350	-370
494	PFC0605w	conserved Plasmodium protein, unknown function	-0.65	0.0020	0.0000	0.0030	0.0145	0.0080	1.1	6243	702	6931	359	-373
215	PFF0175c	conserved Plasmodium protein, unknown function	-0.78	0.0020	0.0000	0.0030	0.0145	0.0080	1.7	1815	532	3165	1191	-373
532	PF14_0428	histidine--tRNA ligase, putative	-0.64	0.0020	0.0000	0.0030	0.0145	0.0080	1.1	7151	382	7809	651	-375
1001	PFI1260c	histone deacetylase	-0.51	0.0040	0.0003	0.0064	0.0219	0.0093	1.0	8296	394	8696	386	-381
234	PFI1370c	phosphatidylserine decarboxylase	-0.77	0.0020	0.0000	0.0030	0.0145	0.0080	1.2	6114	953	7372	687	-381
460	PF11_0273		-0.66	0.0020	0.0000	0.0030	0.0145	0.0080	1.2	4292	512	5036	614	-381
127	PF10_0323	early transcribed membrane protein 10.2	-0.87	0.0020	0.0000	0.0030	0.0145	0.0080	1.5	5141	1229	7638	1652	-384
385	MAL8P1.51	secretory complex protein 61 beta subunit	-0.69	0.0020	0.0000	0.0030	0.0145	0.0080	1.1	7369	638	8216	594	-385
376	PF13_0230	conserved Plasmodium membrane protein, unknown fun	-0.69	0.0020	0.0000	0.0030	0.0145	0.0080	1.3	3000	667	3862	582	-387
996	PF10_0037	conserved Plasmodium protein, unknown function	-0.51	0.0080	0.0024	0.0122	0.0345	0.0142	1.4	1068	303	1476	493	-387
176	PFL1295w	conserved Plasmodium protein, unknown function	-0.82	0.0020	0.0000	0.0030	0.0145	0.0080	1.5	3625	1000	5365	1128	-388
325	PF13_0029	conserved Plasmodium protein, unknown function	-0.72	0.0020	0.0000	0.0030	0.0145	0.0080	1.3	2917	490	3924	906	-390
361	PF14_0280	phosphotyrosyl phosphatase activator, putative	-0.70	0.0020	0.0000	0.0030	0.0145	0.0080	1.3	3087	710	3994	588	-390
111	PF08_0132	glutamate dehydrogenase, putative	-0.89	0.0020	0.0000	0.0030	0.0145	0.0080	1.7	4301	1396	7413	2107	-390
933	PF13_0322	falcilysin	-0.53	0.0040	0.0003	0.0064	0.0219	0.0093	1.1	7979	342	8417	487	-392
406	MAL13P1.39!	krox-like protein. putative	-0.68	0.0020	0.0000	0.0030	0.0145	0.0080	1.1	6487	762	7319	464	-394
1029	PFL1030w	membrane skeletal protein, putative	-0.50	0.0100	0.0036	0.0149	0.0399	0.0162	1.5	786	347	1188	452	-396
321	PFL0095c	conserved protein, unknown function	-0.72	0.0040	0.0003	0.0064	0.0219	0.0093	1.2	4414	662	5454	776	-398
535	PF10_0104	dolichyl-phosphate-mannose protein mannosyltransferas	-0.64	0.0040	0.0003	0.0064	0.0219	0.0093	1.1	7817	566	8513	529	-399
650	PF11_0412	vacuolar ATP synthase subunit f, putative	-0.60	0.0060	0.0012	0.0094	0.0286	0.0120	1.1	7880	495	8481	506	-400
324	PF11_0160	SET domain protein, putative	-0.72	0.0020	0.0000	0.0030	0.0145	0.0080	1.2	6198	712	7238	728	-400
268	MAL13P1.67	methionyl-tRNA formyltransferase, putative	-0.75	0.0020	0.0000	0.0030	0.0145	0.0080	2.9	649	336	1853	1269	-401
472	PF14_0270	apicoplast ribosomal protein L15 precursor, putative	-0.65	0.0020	0.0000	0.0030	0.0145	0.0080	1.1	5589	549	6355	621	-404
347	PF11_0098	endoplasmic reticulum-resident calcium binding protein	-0.71	0.0020	0.0000	0.0030	0.0145	0.0080	1.1	7897	805	8878	582	-406
893	PF11_0227	serine/threonine protein kinase, putative	-0.54	0.0080	0.0024	0.0122	0.0345	0.0142	1.1	3296	364	3771	518	-407
560	PF11_0511	Plasmodium exported protein, unknown function	-0.63	0.0020	0.0000	0.0030	0.0145	0.0080	4.1	220	167	905	925	-407
580	PF14_0099	alpha/beta hydrolase, putative	-0.62	0.0040	0.0003	0.0064	0.0219	0.0093	1.2	3724	398	4392	679	-409
502	PFL1795c	conserved Plasmodium protein, unknown function	-0.65	0.0020	0.0000	0.0030	0.0145	0.0080	1.2	4432	507	5180	652	-410
742	PFF0255c	conserved Plasmodium protein, unknown function	-0.58	0.0080	0.0024	0.0122	0.0345	0.0142	1.2	3267	620	3827	353	-413
204	PF11_0076	conserved Plasmodium protein, unknown function	-0.79	0.0020	0.0000	0.0030	0.0145	0.0080	1.7	2102	830	3651	1131	-413
738	PF14_0242	arginine methyltransferase 1	-0.58	0.0040	0.0003	0.0064	0.0219	0.0093	1.1	8595	615	9167	377	-420
574	PFI0965w	pyridoxal 5'-phosphate dependent enzyme class III, putat	-0.62	0.0020	0.0000	0.0030	0.0145	0.0080	3.7	261	170	954	943	-420
693	PF14_0018	Plasmodium exported protein (PHISTb), unknown functio	-0.59	0.0060	0.0012	0.0094	0.0286	0.0120	1.1	8196	448	8797	575	-421
222	PFA0080c	rifin	-0.78	0.0020	0.0000	0.0030	0.0145	0.0080	2.6	955	467	2473	1478	-427
213	PFE1600w	Plasmodium exported protein (PHISTb), unknown functio	-0.78	0.0020	0.0000	0.0030	0.0145	0.0080	1.2	7868	1259	9430	731	-428
651	PFD0495c	conserved Plasmodium protein, unknown function	-0.60	0.0020	0.0000	0.0030	0.0145	0.0080	1.1	8456	600	9104	481	-433
154	PF13_0358	mitochondrial import inner membrane translocase, putat	-0.84	0.0020	0.0000	0.0030	0.0145	0.0080	2.7	1321	1038	3569	1645	-435
501	PFI1325w	CS domain protein, putative	-0.65	0.0040	0.0003	0.0064	0.0219	0.0093	1.1	5679	791	6475	441	-436
725	PF11_0320	RNA-binding protein s1, putative	-0.58	0.0040	0.0003	0.0064	0.0219	0.0093	1.2	3137	501	3742	542	-438
470	MAL13P1.19(	proteasome regulatory subunit, putative	-0.66	0.0040	0.0003	0.0064	0.0219	0.0093	1.1	7686	791	8530	497	-444
824	PFI0685w	pseudouridylate synthase, putative	-0.55	0.0060	0.0012	0.0094	0.0286	0.0120	1.1	6641	519	7194	478	-444
1104	MAL13P1.54(	heat shock protein 70, putative	-0.48	0.0040	0.0003	0.0064	0.0219	0.0093	1.1	7545	316	7967	554	-448
378	PFI0400c	conserved Plasmodium membrane protein, unknown fun	-0.69	0.0020	0.0000	0.0030	0.0145	0.0080	1.4	2670	481	3666	964	-449
199	PFI1570c	M18 aspartyl aminopeptidase	-0.79	0.0020	0.0000	0.0030	0.0145	0.0080	1.3	6070	1074	7807	1112	-449
479	PF13_0345	glycine cleavage T protein, putative	-0.65	0.0020	0.0000	0.0030	0.0145	0.0080	1.2	4550	684	5397	612	-449

327	PF14_0097	cytidine diphosphate-diacylglycerol synthase	-0.72	0.0020	0.0000	0.0030	0.0145	0.0080	1.2	6890	860	8046	745	-450		
950	PF11_0357	zinc finger protein, putative	-0.52	0.0040	0.0003	0.0064	0.0219	0.0093	2.5	336	154	831	791	-450		
796	PFI1645c	histidyl-tRNA synthetase, putative	-0.56	0.0020	0.0000	0.0030	0.0145	0.0080	1.1	5409	543	5987	486	-451		
882	PFB0615c	conserved Plasmodium protein, unknown function	-0.54	0.0040	0.0003	0.0064	0.0219	0.0093	1.3	1849	446	2381	538	-451		
563	PF14_0432	conserved Plasmodium protein, unknown function	-0.63	0.0020	0.0000	0.0030	0.0145	0.0080	1.1	6298	548	7053	659	-452		
297	PF11_0107	conserved Plasmodium protein, unknown function	-0.73	0.0020	0.0000	0.0030	0.0145	0.0080	1.2	4979	1026	6224	673	-454		
506	MAL8P1.1	surface-associated interspersed gene 8.1, (SURFIN8.1)	-0.65	0.0040	0.0003	0.0064	0.0219	0.0093	1.4	2001	441	2829	843	-455		
490	PF14_0338		-0.65	0.0080	0.0024	0.0122	0.0345	0.0142	1.4	1919	430	2768	876	-456		
308	PFD0545w	conserved Plasmodium protein, unknown function	-0.73	0.0020	0.0000	0.0030	0.0145	0.0080	1.2	7211	656	8434	1024	-457		
831	PFD0435c	conserved Plasmodium protein, unknown function	-0.55	0.0060	0.0012	0.0094	0.0286	0.0120	1.4	1309	410	1881	624	-462		
497	PF10_0113	conserved Plasmodium protein, unknown function	-0.65	0.0020	0.0000	0.0030	0.0145	0.0080	1.2	4606	631	5456	684	-465		
881	PFL1445w	conserved Plasmodium protein, unknown function	-0.54	0.0060	0.0012	0.0094	0.0286	0.0120	1.1	7576	575	8127	443	-467		
720	PF10_0013	Plasmodium exported protein (hyp12), unknown functior	-0.58	0.0060	0.0012	0.0094	0.0286	0.0120	2.9	346	166	994	949	-468		
906	PFC0025c	stevor	-0.53	0.0120	0.0050	0.0175	0.0456	0.0185	2.8	301	177	837	827	-468		
666	PF11_0099	heat shock protein DnaJ homologue Pfj2	-0.60	0.0020	0.0000	0.0030	0.0145	0.0080	1.1	6693	436	7384	725	-470		
303	PF11_0413	conserved Plasmodium protein, unknown function	-0.73	0.0020	0.0000	0.0030	0.0145	0.0080	1.3	4389	436	5657	1302	-470		
585	PF14_0516	serine/threonine protein kinase, putative	-0.62	0.0100	0.0036	0.0149	0.0399	0.0162	1.2	4937	449	5702	786	-471		
294	PFF0465c	mitochondrial cardiolipin synthase, PfCLS	-0.73	0.0020	0.0000	0.0030	0.0145	0.0080	1.7	1744	591	3041	1178	-472		
370	MAL13P1.22	aspartate carbamoyltransferase	-0.70	0.0020	0.0000	0.0030	0.0145	0.0080	1.2	5953	742	7033	811	-474		
484	PFL1785c	conserved Plasmodium protein, unknown function	-0.65	0.0060	0.0012	0.0094	0.0286	0.0120	1.6	1607	455	2499	911	-475		
350	MAL13P1.32	mitochondrial ribosomal protein S17 precursor, putative	-0.71	0.0020	0.0000	0.0030	0.0145	0.0080	1.7	1589	590	2734	1030	-475		
601	MAL8P1.32	nucleoside transporter 2	-0.61	0.0020	0.0000	0.0030	0.0145	0.0080	1.1	6485	664	7251	581	-480		
402	MAL8P1.155		-0.68	0.0020	0.0000	0.0030	0.0145	0.0080	1.4	2673	549	3694	953	-481		
293	PF10_0173	conserved Plasmodium protein, unknown function	-0.73	0.0020	0.0000	0.0030	0.0145	0.0080	1.4	3384	801	4715	1012	-483		
905	PF14_0792	conserved Plasmodium protein, unknown function	-0.53	0.0080	0.0024	0.0122	0.0345	0.0142	1.4	1393	488	1947	550	-484		
647	PFF0120w	geranylgeranyltransferase, putative	-0.60	0.0060	0.0012	0.0094	0.0286	0.0120	1.1	6272	704	7002	510	-485		
233	PFL0780w	glycerol-3-phosphate dehydrogenase, putative	-0.77	0.0020	0.0000	0.0030	0.0145	0.0080	1.3	6370	1264	7986	838	-485		
870	PF07_0005	lysophospholipase, putative	-0.54	0.0040	0.0003	0.0064	0.0219	0.0093	1.1	7625	337	8207	733	-489		
349	PFL2640c	rifin	-0.71	0.0020	0.0000	0.0030	0.0145	0.0080	2.9	608	528	1789	1143	-490		
628	PF14_0089		-0.61	0.0040	0.0003	0.0064	0.0219	0.0093	1.7	1076	392	1831	855	-491		
545	PF13_0335	conserved Plasmodium protein, unknown function	-0.63	0.0020	0.0000	0.0030	0.0145	0.0080	1.1	6520	1003	7366	334	-491		
724	MAL13P1.314		-0.58	0.0040	0.0003	0.0064	0.0219	0.0093	1.7	969	366	1654	814	-495		
475	PF14_0190	conserved Plasmodium protein, unknown function	-0.65	0.0020	0.0000	0.0030	0.0145	0.0080	1.5	2004	617	2948	826	-499		
1037	PFA0360c	hypothetical protein conserved in P. falciparum	-0.50	0.0060	0.0012	0.0094	0.0286	0.0120	1.3	1542	328	2045	675	-500		
1070	PF11_0021	rifin	-0.49	0.0120	0.0050	0.0175	0.0456	0.0185	2.2	404	272	890	716	-502		
615	PFC0065c	alpha/beta hydrolase, putative	-0.61	0.0020	0.0000	0.0030	0.0145	0.0080	1.1	5848	400	6635	888	-502		
752	PF10_0291	RAP protein, putative	-0.57	0.0040	0.0003	0.0064	0.0219	0.0093	1.3	2574	400	3251	780	-502		
1279	MAL13P1.34	proteasome regulatory subunit, putative	-0.45	0.0080	0.0024	0.0122	0.0345	0.0142	1.1	7174	405	7590	513	-503		
445	PF14_0746	Plasmodium exported protein (PHISTb), unknown functio	-0.67	0.0020	0.0000	0.0030	0.0145	0.0080	1.1	7688	497	8691	1012	-505		
652	PFD0965W	phosphatidylinositol 4-kinase, putative	-0.60	0.0020	0.0000	0.0030	0.0145	0.0080	1.1	7288	434	8045	830	-507		
1046	MAL7P1.107	conserved Plasmodium protein, unknown function	-0.50	0.0060	0.0012	0.0094	0.0286	0.0120	1.1	7978	663	8482	350	-508		
821	PF14_0276	mitochondrial ribosomal protein L15 precursor, putative	-0.56	0.0040	0.0003	0.0064	0.0219	0.0093	1.1	7674	593	8316	562	-513		
992	PF14_0092	conserved Plasmodium membrane protein, unknown fun	-0.51	0.0100	0.0036	0.0149	0.0399	0.0162	2.1	481	192	1025	866	-514		
178	PFE0580w	kinase binding protein CGI-121, putative	-0.82	0.0020	0.0000	0.0030	0.0145	0.0080	1.9	2641	1553	4928	1248	-514		
913	PFL2030w	queuine tRNA-ribosyltransferase, putative	-0.53	0.0040	0.0003	0.0064	0.0219	0.0093	1.1	7548	619	8134	482	-515		
316	PF11_0269	conserved Plasmodium protein, unknown function	-0.72	0.0020	0.0000	0.0030	0.0145	0.0080	1.3	4483	876	5839	995	-516		
1048	PF08_0111	RNA helicase, putative	-0.50	0.0080	0.0024	0.0122	0.0345	0.0142	1.1	7054	484	7567	547	-518		

245	PFB0075c	Plasmodium exported protein (hyp9), unknown function	-0.76	0.0020	0.0000	0.0030	0.0145	0.0080	1.2	6752	923	8418	1263	-520
565	PF11_0292	cochaperone prefoldin complex subunit, putative	-0.63	0.0060	0.0012	0.0094	0.0286	0.0120	1.1	6812	646	7684	749	-523
469	PFI1030c		-0.66	0.0020	0.0000	0.0030	0.0145	0.0080	1.2	6265	888	7262	634	-524
923	PFD1105w	asparagine-rich protein	-0.53	0.0020	0.0000	0.0030	0.0145	0.0080	2.5	387	112	977	1003	-525
1023	PF14_0176	conserved Plasmodium protein, unknown function	-0.51	0.0080	0.0024	0.0122	0.0345	0.0142	1.1	7180	514	7717	549	-525
1097	PF14_0060	glycerophodiester phosphodiesterase, putative	-0.49	0.0120	0.0050	0.0175	0.0456	0.0185	1.1	6742	581	7242	448	-529
498	PFB0870w	conserved Plasmodium protein, unknown function	-0.65	0.0020	0.0000	0.0030	0.0145	0.0080	1.2	6011	791	6979	706	-529
240	PF11_0010	rifin	-0.76	0.0020	0.0000	0.0030	0.0145	0.0080	2.0	1667	856	3387	1394	-530
419	PFF0665c	syntaxin binding protein, putative	-0.67	0.0020	0.0000	0.0030	0.0145	0.0080	1.2	5049	585	6148	1048	-534
315	PFI1525w	conserved Plasmodium protein, unknown function	-0.73	0.0020	0.0000	0.0030	0.0145	0.0080	1.3	5548	923	6966	1032	-538
790	PFD1175w	serine/threonine protein kinase, FIKK family	-0.56	0.0040	0.0003	0.0064	0.0219	0.0093	1.1	8524	674	9218	560	-540
277	PF13_0300	mitochondrial inner membrane translocase, putative	-0.74	0.0020	0.0000	0.0030	0.0145	0.0080	1.3	4891	885	6460	1225	-541
431	PF11_0265	mitochondrial inner membrane translocase subunit tim44	-0.67	0.0040	0.0003	0.0064	0.0219	0.0093	1.2	5831	890	6926	746	-542
514	PF11_0461	Rab GTPase 6	-0.64	0.0040	0.0003	0.0064	0.0219	0.0093	1.2	4411	910	5393	619	-546
408	PFB0391c		-0.68	0.0020	0.0000	0.0030	0.0145	0.0080	1.2	7154	1032	8304	666	-548
272	PFL2655w	rifin	-0.75	0.0020	0.0000	0.0030	0.0145	0.0080	2.3	1285	625	2916	1554	-548
748	PFB0125c	conserved Plasmodium protein, unknown function	-0.58	0.0020	0.0000	0.0030	0.0145	0.0080	1.6	1282	306	2032	997	-554
1245	PF13_0040	DNA-directed RNA polymerase alpha chain, putative	-0.46	0.0080	0.0024	0.0122	0.0345	0.0142	1.1	3816	385	4288	641	-554
976	PF13_0133	plasmepsin V	-0.52	0.0060	0.0012	0.0094	0.0286	0.0120	1.1	7846	479	8443	672	-554
956	PFD0735c	conserved Plasmodium protein, unknown function	-0.52	0.0040	0.0003	0.0064	0.0219	0.0093	1.6	1025	600	1632	563	-556
541	PFI0125c		-0.63	0.0020	0.0000	0.0030	0.0145	0.0080	1.2	5209	493	6171	1025	-556
530	PF11_0527	conserved Plasmodium protein, unknown function	-0.64	0.0020	0.0000	0.0030	0.0145	0.0080	1.3	2998	723	3982	818	-557
453	PF11_0257	ethanolamine kinase, putative	-0.66	0.0040	0.0003	0.0064	0.0219	0.0093	1.3	4297	690	5401	973	-560
779	PFL1185c	cytochrome c heme lyase, putative	-0.57	0.0040	0.0003	0.0064	0.0219	0.0093	1.1	7130	517	7868	784	-563
780	PFC1055w	conserved Plasmodium protein, unknown function	-0.57	0.0020	0.0000	0.0030	0.0145	0.0080	1.2	4832	584	5572	719	-564
781	PF11_0059	metabolite/drug transporter, putative	-0.57	0.0060	0.0012	0.0094	0.0286	0.0120	1.2	4741	670	5481	635	-565
515	PFL2245w	signal recognition particle receptor, beta subunit	-0.64	0.0020	0.0000	0.0030	0.0145	0.0080	1.2	4825	665	5849	930	-571
802	PF07_0060	conserved Plasmodium protein, unknown function	-0.56	0.0100	0.0036	0.0149	0.0399	0.0162	1.3	2489	685	3215	612	-571
334	PF11_0351	heat shock protein 70	-0.72	0.0020	0.0000	0.0030	0.0145	0.0080	1.3	5818	1107	7273	925	-577
442	PF10_0318	conserved protein, unknown function	-0.67	0.0040	0.0003	0.0064	0.0219	0.0093	1.4	3136	720	4290	1012	-577
416	PF14_0399	ADP-ribosylation factor, putative	-0.67	0.0040	0.0003	0.0064	0.0219	0.0093	2.0	1260	395	2458	1380	-578
434	MAL13P1.318	mitochondrial ribosomal protein L9 precursor, putative	-0.67	0.0020	0.0000	0.0030	0.0145	0.0080	1.5	2452	723	3619	1022	-578
581	PFE0110w	conserved Plasmodium membrane protein, unknown fun	-0.62	0.0020	0.0000	0.0030	0.0145	0.0080	1.2	5686	559	6636	972	-582
629	PFE0340c	rhomboid protease ROM4	-0.61	0.0020	0.0000	0.0030	0.0145	0.0080	1.2	4937	899	5833	580	-582
959	PF11_0063	conserved Plasmodium protein, unknown function	-0.52	0.0060	0.0012	0.0094	0.0286	0.0120	1.3	2216	569	2852	651	-584
1353	PFI0670w	conserved Plasmodium protein, unknown function	-0.44	0.0040	0.0003	0.0064	0.0219	0.0093	1.7	629	110	1088	933	-584
239	PFE0520c	topoisomerase I	-0.76	0.0020	0.0000	0.0030	0.0145	0.0080	1.5	3989	1046	5892	1443	-586
999	MAL8P1.203	serine/threonine protein kinase, FIKK family	-0.51	0.0080	0.0024	0.0122	0.0345	0.0142	1.2	2566	448	3183	756	-587
625	PFL0010c	rifin	-0.61	0.0040	0.0003	0.0064	0.0219	0.0093	2.3	689	470	1598	1027	-589
938	PFF0425w	conserved Plasmodium protein, unknown function	-0.53	0.0060	0.0012	0.0094	0.0286	0.0120	1.3	1968	499	2628	754	-593
523	PFD0035c	stevor	-0.64	0.0020	0.0000	0.0030	0.0145	0.0080	1.9	1180	678	2235	971	-594
377	PFB0926c	Plasmodium exported protein (hyp2), unknown function	-0.69	0.0020	0.0000	0.0030	0.0145	0.0080	1.4	3006	841	4333	1084	-597
1652	PFF1325c	c3h4-type ring finger protein, putative	-0.39	0.0060	0.0012	0.0094	0.0286	0.0120	1.1	7469	803	7846	172	-598
186	PFB0620w	conserved protein, unknown function	-0.81	0.0020	0.0000	0.0030	0.0145	0.0080	1.9	2702	1436	5245	1706	-599
252	PF13_0235	transcription factor with AP2 domain(s), putative	-0.76	0.0020	0.0000	0.0030	0.0145	0.0080	1.5	3977	1183	5868	1310	-602
922	PFC10_API00	null	-0.53	0.0080	0.0024	0.0122	0.0345	0.0142	1.9	797	290	1475	990	-603
1241	PF11_0195	conserved Plasmodium protein, unknown function	-0.46	0.0100	0.0036	0.0149	0.0399	0.0162	1.3	1990	482	2505	637	-604

763	PF14_0439	M17 leucyl aminopeptidase	-0.57	0.0060	0.0012	0.0094	0.0286	0.0120	1.1	8009	684	8817	729	-605
483	PFL0730w	conserved Plasmodium protein, unknown function	-0.65	0.0020	0.0000	0.0030	0.0145	0.0080	1.3	4215	758	5361	999	-610
424	MAL13P1.24	conserved Plasmodium protein, unknown function	-0.67	0.0020	0.0000	0.0030	0.0145	0.0080	1.6	2181	822	3426	1034	-611
632	PF11_0473	conserved Plasmodium protein, unknown function	-0.61	0.0040	0.0003	0.0064	0.0219	0.0093	1.5	1853	663	2791	886	-611
997	PF14_0161	conserved protein, unknown function	-0.51	0.0060	0.0012	0.0094	0.0286	0.0120	1.2	2870	549	3514	705	-611
1364	PFF1010c	DnaJ protein, putative	-0.44	0.0120	0.0050	0.0175	0.0456	0.0185	1.4	1255	456	1733	636	-614
613	PFE1170w	DnaJ protein, putative	-0.61	0.0040	0.0003	0.0064	0.0219	0.0093	1.5	2024	513	2987	1064	-614
733	PF13_0098	conserved Plasmodium protein, unknown function	-0.58	0.0040	0.0003	0.0064	0.0219	0.0093	1.1	6594	713	7440	751	-618
544	PFE0320w	conserved Plasmodium protein, unknown function	-0.63	0.0020	0.0000	0.0030	0.0145	0.0080	1.8	1335	533	2401	1152	-619
476	PFI0002w		-0.65	0.0040	0.0003	0.0064	0.0219	0.0093	1.3	4604	667	5774	1123	-619
703	PF14_0674	conserved Plasmodium protein, unknown function	-0.59	0.0020	0.0000	0.0030	0.0145	0.0080	3.0	446	288	1329	1221	-626
904	PFL0465c	zinc finger transcription factor (krox1)	-0.53	0.0060	0.0012	0.0094	0.0286	0.0120	1.4	1695	675	2414	671	-627
1091	MAL13P1.64	ubiquitin-like protein nedd8 homologue, putative	-0.49	0.0120	0.0050	0.0175	0.0456	0.0185	1.1	5762	609	6358	616	-628
753	PF14_0666	conserved Plasmodium protein, unknown function	-0.57	0.0080	0.0024	0.0122	0.0345	0.0142	1.4	2210	581	3062	901	-631
743	PFF0115c	elongation factor G, putative	-0.58	0.0020	0.0000	0.0030	0.0145	0.0080	1.3	2745	606	3608	893	-636
568	MAL7P1.126	conserved Plasmodium protein, unknown function	-0.62	0.0020	0.0000	0.0030	0.0145	0.0080	1.2	5393	794	6452	903	-638
659	PFE0560c	MORN repeat protein, putative	-0.60	0.0040	0.0003	0.0064	0.0219	0.0093	2.0	989	458	1934	1125	-638
692	PF11_0374	tudor staphylococcal nuclease	-0.59	0.0060	0.0012	0.0094	0.0286	0.0120	1.1	6570	1016	7494	554	-646
364	PFL1395c	conserved Plasmodium protein, unknown function	-0.70	0.0020	0.0000	0.0030	0.0145	0.0080	1.3	5521	1493	7029	667	-652
700	PFI1375w		-0.59	0.0040	0.0003	0.0064	0.0219	0.0093	1.2	5358	550	6283	1029	-654
441	MAL7P1.32	nucleotide excision repair protein, putative	-0.67	0.0020	0.0000	0.0030	0.0145	0.0080	1.5	2706	868	4019	1100	-655
690	PFC0955w	ATP-dependent RNA helicase, putative	-0.59	0.0040	0.0003	0.0064	0.0219	0.0093	1.2	3930	626	4873	971	-655
973	PFI0155c	Rab GTPase 7	-0.52	0.0060	0.0012	0.0094	0.0286	0.0120	1.6	1171	395	1879	969	-655
642	PFD0470c	replication protein A large subunit	-0.60	0.0020	0.0000	0.0030	0.0145	0.0080	1.7	1336	411	2333	1244	-658
1170	MAL7P1.98	conserved Plasmodium protein, unknown function	-0.47	0.0080	0.0024	0.0122	0.0345	0.0142	2.2	503	306	1097	948	-661
444	PF14_0471	transcription factor with AP2 domain(s), putative	-0.67	0.0020	0.0000	0.0030	0.0145	0.0080	1.5	2822	976	4143	1009	-664
1208	PF10_0045	conserved Plasmodium membrane protein, unknown fun	-0.47	0.0060	0.0012	0.0094	0.0286	0.0120	1.1	6786	730	7368	521	-668
1172	PFL0080c	NIMA related kinase 3	-0.47	0.0060	0.0012	0.0094	0.0286	0.0120	2.4	420	294	1021	977	-670
961	PFE1160w	conserved Plasmodium protein, unknown function	-0.52	0.0020	0.0000	0.0030	0.0145	0.0080	1.1	5566	607	6296	794	-671
1290	PFD0835c	LETM1-like protein, putative	-0.45	0.0120	0.0050	0.0175	0.0456	0.0185	1.1	5894	610	6443	613	-673
509	MAL8P1.139	conserved Plasmodium membrane protein, unknown fun	-0.64	0.0040	0.0003	0.0064	0.0219	0.0093	1.2	5478	1034	6697	860	-674
558	PF07_0087	conserved Plasmodium protein, unknown function	-0.63	0.0020	0.0000	0.0030	0.0145	0.0080	1.4	3139	758	4283	1065	-678
764	PF14_0272	CPW-WPC family protein	-0.57	0.0020	0.0000	0.0030	0.0145	0.0080	1.3	3514	707	4423	883	-681
505	MAL8P1.80	conserved Plasmodium protein, unknown function	-0.65	0.0020	0.0000	0.0030	0.0145	0.0080	1.8	1486	817	2725	1104	-682
670	PFI1365w	cytochrome c oxidase subunit, putative	-0.59	0.0060	0.0012	0.0094	0.0286	0.0120	1.3	3315	551	4315	1131	-683
284	PF08_0095	dihydropteroate synthetase	-0.74	0.0020	0.0000	0.0030	0.0145	0.0080	2.6	1196	606	3160	2044	-685
438	PFF1415c	DnaJ protein, putative	-0.67	0.0040	0.0003	0.0064	0.0219	0.0093	1.3	4475	778	5852	1286	-687
542	PF10_0399	rifin	-0.63	0.0040	0.0003	0.0064	0.0219	0.0093	2.0	1162	672	2353	1208	-689
1164	MAL8P1.25	conserved Plasmodium protein, unknown function	-0.47	0.0080	0.0024	0.0122	0.0345	0.0142	1.5	1340	469	1962	846	-692
437	MAL7P1.216	rifin	-0.67	0.0020	0.0000	0.0030	0.0145	0.0080	2.0	1363	828	2760	1265	-696
671	MAL13P1.54	conserved Plasmodium protein, unknown function	-0.59	0.0040	0.0003	0.0064	0.0219	0.0093	1.2	6033	931	7052	784	-696
318	MAL8P1.107	conserved Plasmodium protein, unknown function	-0.72	0.0020	0.0000	0.0030	0.0145	0.0080	1.9	2035	754	3858	1766	-696
392	PFA0465c	N-acetyltransferase, putative	-0.68	0.0040	0.0003	0.0064	0.0219	0.0093	1.4	3870	994	5382	1214	-697
246	PF14_0257	conserved protein, unknown function	-0.76	0.0020	0.0000	0.0030	0.0145	0.0080	1.6	3894	1685	6128	1247	-698
931	PFL1920c	hydroxyethylthiazole kinase, putative	-0.53	0.0060	0.0012	0.0094	0.0286	0.0120	1.3	2705	544	3487	937	-700
1211	PF13_0205	tryptophan--tRNA ligase, putative	-0.47	0.0080	0.0024	0.0122	0.0345	0.0142	1.1	7528	787	8136	521	-700
760	PFL1775c	s-adenosyl-methyltransferase, putative	-0.57	0.0080	0.0024	0.0122	0.0345	0.0142	1.2	5822	846	6761	796	-703

648	PF14_0363	metacaspase-like protein	-0.60	0.0060	0.0012	0.0094	0.0286	0.0120	1.3	3536	833	4597	932	-704
599	MAL7P1.223	stevor	-0.62	0.0040	0.0003	0.0064	0.0219	0.0093	1.6	2004	849	3135	988	-706
616	PFC0725c	formate-nitrite transporter, putative	-0.61	0.0020	0.0000	0.0030	0.0145	0.0080	1.2	7254	796	8368	1029	-711
1054	PF14_0440	conserved Plasmodium membrane protein, unknown fun	-0.50	0.0100	0.0036	0.0149	0.0399	0.0162	1.1	5150	659	5856	765	-718
1083	PFB0610c	conserved Plasmodium protein, unknown function	-0.49	0.0120	0.0050	0.0175	0.0456	0.0185	1.2	4060	790	4754	630	-726
1093	MAL13P1.82	phosphatidylinositol synthase	-0.49	0.0060	0.0012	0.0094	0.0286	0.0120	1.2	4566	589	5254	826	-726
903	PF11_0186	conserved Plasmodium protein, unknown function	-0.53	0.0020	0.0000	0.0030	0.0145	0.0080	1.5	1523	488	2359	1076	-729
1723	PF10_0363	pyruvate kinase 2, putative	-0.37	0.0100	0.0036	0.0149	0.0399	0.0162	2.7	254	84	687	1079	-730
330	PF10_0274	methyltransferase, putative	-0.72	0.0020	0.0000	0.0030	0.0145	0.0080	1.4	4789	1150	6657	1449	-732
797	PFI0380c	peptidyl deformylase	-0.56	0.0040	0.0003	0.0064	0.0219	0.0093	1.5	1832	727	2774	952	-736
1031	MAL13P1.59	Plasmodium exported protein (PHISTa), unknown functio	-0.50	0.0080	0.0024	0.0122	0.0345	0.0142	2.0	716	232	1463	1256	-740
741	PF11_0258	co-chaperone GrpE, putative	-0.58	0.0020	0.0000	0.0030	0.0145	0.0080	1.6	1610	483	2622	1272	-744
411	PF11_0164	peptidyl-prolyl cis-trans isomerase,cyclophilin	-0.68	0.0020	0.0000	0.0030	0.0145	0.0080	1.3	5518	1098	7076	1205	-745
543	PFB0886c	conserved Plasmodium protein, unknown function	-0.63	0.0040	0.0003	0.0064	0.0219	0.0093	1.7	1749	778	3038	1256	-745
921	PFE0765w	phosphatidylinositol 3-kinase	-0.53	0.0080	0.0024	0.0122	0.0345	0.0142	1.1	6430	1020	7275	576	-751
941	PFF0640w	conserved Plasmodium protein, unknown function	-0.53	0.0120	0.0050	0.0175	0.0456	0.0185	1.3	3212	866	4050	726	-754
401	PFB0035c	rifin	-0.68	0.0020	0.0000	0.0030	0.0145	0.0080	1.8	1905	1170	3504	1184	-754
990	PFA0195w	parasite-infected erythrocyte surface protein	-0.52	0.0080	0.0024	0.0122	0.0345	0.0142	1.1	5901	678	6706	885	-757
952	PF11_0328	conserved Plasmodium protein, unknown function	-0.52	0.0080	0.0024	0.0122	0.0345	0.0142	1.3	2986	580	3820	1014	-760
820	PF10_0036	N-acetyltransferase, putative	-0.56	0.0040	0.0003	0.0064	0.0219	0.0093	1.2	5959	697	6911	1016	-761
344	PF14_0673	RAP protein, putative	-0.71	0.0020	0.0000	0.0030	0.0145	0.0080	1.9	2166	1166	4044	1475	-763
238	PF14_0545	thioredoxin, putative	-0.76	0.0020	0.0000	0.0030	0.0145	0.0080	2.0	2492	1254	4980	1999	-765
974	PF13_0170	glutaminyl-tRNA synthetase, putative	-0.52	0.0040	0.0003	0.0064	0.0219	0.0093	1.1	6522	928	7348	663	-765
1081	MAL8P1.131	gas41 homologue, putative	-0.49	0.0100	0.0036	0.0149	0.0399	0.0162	1.1	6062	807	6796	693	-766
538	PF13_0017	conserved Plasmodium protein, unknown function	-0.63	0.0020	0.0000	0.0030	0.0145	0.0080	1.3	3872	805	5208	1299	-769
909	PF11_0264	DNA-dependent RNA polymerase	-0.53	0.0020	0.0000	0.0030	0.0145	0.0080	1.5	1651	663	2528	983	-769
521	PFF0945c	acyl-CoA synthetase, PfACS12	-0.64	0.0040	0.0003	0.0064	0.0219	0.0093	1.3	3953	1052	5322	1088	-770
1015	PF10_0373	GDP dissociation inhibitor domain containing protein	-0.51	0.0040	0.0003	0.0064	0.0219	0.0093	2.3	624	276	1417	1287	-771
285	PFL0430w	tim10 homologue, putative	-0.74	0.0020	0.0000	0.0030	0.0145	0.0080	1.5	4651	1415	6859	1565	-772
776	MAL8P1.130	conserved Plasmodium membrane protein, unknown fun	-0.57	0.0040	0.0003	0.0064	0.0219	0.0093	1.2	4368	709	5388	1084	-773
388	PFL0865w	conserved protein, unknown function	-0.69	0.0020	0.0000	0.0030	0.0145	0.0080	2.2	1463	919	3156	1548	-774
260	MAL13P1.405	conserved protein, unknown function	-0.75	0.0020	0.0000	0.0030	0.0145	0.0080	1.5	4468	964	6839	2182	-776
1077	PFB0535w	GDP-fructose:GMP antiporter, putative	-0.49	0.0080	0.0024	0.0122	0.0345	0.0142	1.3	2684	627	3433	899	-777
739	PFA0530c	adenylate kinase, putative	-0.58	0.0060	0.0012	0.0094	0.0286	0.0120	1.2	4863	916	5924	924	-779
292	MAL8P1.214	stevor, pseudogene	-0.74	0.0020	0.0000	0.0030	0.0145	0.0080	1.7	3003	1445	5180	1517	-785
346	PFD0669c	conserved Plasmodium protein, unknown function	-0.71	0.0020	0.0000	0.0030	0.0145	0.0080	1.5	4097	1200	6002	1494	-788
595	PFE0640w	conserved Plasmodium protein, unknown function	-0.62	0.0020	0.0000	0.0030	0.0145	0.0080	1.7	1881	593	3167	1488	-795
1053	PF14_0658	translation initiation factor EF-1, putative	-0.50	0.0080	0.0024	0.0122	0.0345	0.0142	1.4	2149	517	2935	1068	-799
855	PF08_0002	surface-associated interspersed gene 8.2 (SURFIN8.2)	-0.55	0.0020	0.0000	0.0030	0.0145	0.0080	1.9	1078	676	2041	1087	-800
265	PF14_0386	adaptor complexes medium subunit family	-0.75	0.0020	0.0000	0.0030	0.0145	0.0080	2.1	2188	868	4629	2373	-800
803	PF13_0356	conserved Plasmodium protein, unknown function	-0.56	0.0040	0.0003	0.0064	0.0219	0.0093	1.2	6089	1314	7111	514	-806
1594	MAL13P1.86	cholinephosphate cytidylyltransferase	-0.40	0.0020	0.0000	0.0030	0.0145	0.0080	2.1	503	92	1032	1243	-806
713	PF14_0237	conserved Plasmodium protein, unknown function	-0.58	0.0040	0.0003	0.0064	0.0219	0.0093	1.2	5735	897	6874	1055	-813
622	PF14_0226	conserved Plasmodium protein, unknown function	-0.61	0.0020	0.0000	0.0030	0.0145	0.0080	1.2	6503	773	7781	1328	-822
737	PFC0330w	conserved Plasmodium protein, unknown function	-0.58	0.0060	0.0012	0.0094	0.0286	0.0120	1.2	5337	1088	6460	860	-824
597	PFE1335c	conserved Plasmodium protein, unknown function	-0.62	0.0040	0.0003	0.0064	0.0219	0.0093	1.2	6297	1252	7623	899	-825
919	PF11_0338	aquaglyceroporin	-0.53	0.0060	0.0012	0.0094	0.0286	0.0120	1.3	3577	788	4512	975	-829

1215	PFL0270c	conserved Plasmodium protein, unknown function	-0.46	0.0100	0.0036	0.0149	0.0399	0.0162	1.2	4469	732	5194	829	-836
927	PFI0835c	N-glycosylase/DNA lyase, putative	-0.53	0.0080	0.0024	0.0122	0.0345	0.0142	1.2	4802	952	5743	829	-840
611	PF07_0008	Plasmodium exported protein, unknown function	-0.61	0.0040	0.0003	0.0064	0.0219	0.0093	1.2	6778	725	8101	1440	-842
1192	MAL13P1.27	proteasome subunit, putative	-0.47	0.0060	0.0012	0.0094	0.0286	0.0120	1.1	6854	1082	7603	520	-852
1154	PFD0365c	conserved Plasmodium protein, unknown function	-0.48	0.0080	0.0024	0.0122	0.0345	0.0142	1.2	4488	831	5267	807	-860
1304	PFL0910c		-0.45	0.0100	0.0036	0.0149	0.0399	0.0162	1.4	1880	449	2578	1110	-862
1298	PFE0825w	metabolite/drug transporter, putative	-0.45	0.0100	0.0036	0.0149	0.0399	0.0162	1.5	1463	458	2165	1109	-865
567	PF10_0169	phosphomannomutase, putative	-0.62	0.0020	0.0000	0.0030	0.0145	0.0080	1.2	6356	1230	7798	1079	-867
537	PFL1790w	ubiquitin-activating enzyme, putative	-0.63	0.0040	0.0003	0.0064	0.0219	0.0093	1.5	3102	1169	4617	1217	-871
1158	PF13_0146	conserved Plasmodium protein, unknown function	-0.47	0.0040	0.0003	0.0064	0.0219	0.0093	1.2	3172	714	3959	943	-871
1178	PF14_0038	cytochrome c, putative	-0.47	0.0080	0.0024	0.0122	0.0345	0.0142	1.1	7070	854	7850	797	-871
782	PFI1815c	rifin	-0.57	0.0080	0.0024	0.0122	0.0345	0.0142	2.7	680	785	1827	1240	-878
774	PFL1155w	GTP cyclohydrolase I	-0.57	0.0020	0.0000	0.0030	0.0145	0.0080	9.4	139	90	1300	1949	-878
844	PFB0095c	erythrocyte membrane protein 3	-0.55	0.0040	0.0003	0.0064	0.0219	0.0093	1.1	7765	817	8840	1143	-885
812	MAL13P1.34	conserved Plasmodium protein, unknown function	-0.56	0.0060	0.0012	0.0094	0.0286	0.0120	1.3	3910	968	5024	1032	-886
1110	MAL13P1.13	conserved Plasmodium protein, unknown function	-0.48	0.0080	0.0024	0.0122	0.0345	0.0142	1.3	2764	686	3592	1029	-887
1069	MAL13P1.73	conserved Plasmodium protein, unknown function	-0.49	0.0060	0.0012	0.0094	0.0286	0.0120	1.2	3835	903	4696	845	-887
351	MAL8P1.36	conserved Plasmodium protein, unknown function	-0.71	0.0020	0.0000	0.0030	0.0145	0.0080	1.6	3747	1711	5877	1309	-890
564	PFD0165w	ubiquitin specific protease, putative	-0.63	0.0020	0.0000	0.0030	0.0145	0.0080	1.7	2016	1013	3504	1365	-891
929	PF11_0346	conserved Plasmodium protein, unknown function	-0.53	0.0060	0.0012	0.0094	0.0286	0.0120	1.2	4969	1142	5974	761	-899
1096	PFD0775c	RNA binding protein, putative	-0.49	0.0080	0.0024	0.0122	0.0345	0.0142	1.3	2891	656	3754	1121	-913
980	PF14_0259	conserved Plasmodium protein, unknown function	-0.52	0.0020	0.0000	0.0030	0.0145	0.0080	1.7	1371	601	2360	1310	-922
1036	PFE1615c	Plasmodium exported protein, unknown function	-0.50	0.0080	0.0024	0.0122	0.0345	0.0142	1.9	1077	418	2007	1436	-923
784	PFI1310w	NAD synthase, putative	-0.57	0.0040	0.0003	0.0064	0.0219	0.0093	1.2	4995	1336	6206	806	-931
1130	PFC1100w	rifin	-0.48	0.0060	0.0012	0.0094	0.0286	0.0120	9.5	101	70	960	1720	-931
425	PFD0985w	transcription factor with AP2 domain(s), putative	-0.67	0.0020	0.0000	0.0030	0.0145	0.0080	1.4	4564	1437	6466	1400	-935
957	PFI1190w	conserved Plasmodium protein, unknown function	-0.52	0.0060	0.0012	0.0094	0.0286	0.0120	1.4	2517	762	3542	1201	-938
1064	PF14_0662	nucleoside transporter, putative	-0.49	0.0020	0.0000	0.0030	0.0145	0.0080	1.2	5343	759	6269	1118	-951
1047	PFD0865c	cdc2-related protein kinase 1	-0.50	0.0100	0.0036	0.0149	0.0399	0.0162	1.4	2364	900	3309	998	-953
704	PFF0160c	dihydroorotate dehydrogenase, mitochondrial precursor	-0.59	0.0060	0.0012	0.0094	0.0286	0.0120	1.4	3305	1082	4649	1215	-953
422	PFE0330w	conserved Plasmodium protein, unknown function	-0.67	0.0020	0.0000	0.0030	0.0145	0.0080	1.7	2619	1044	4575	1868	-956
329	MAL7P1.38	regulator of chromosome condensation, putative	-0.72	0.0020	0.0000	0.0030	0.0145	0.0080	1.8	3050	1626	5500	1781	-957
1297	PFA0430c	secreted ookinete protein, putative	-0.45	0.0080	0.0024	0.0122	0.0345	0.0142	1.1	5884	824	6663	913	-959
345	PF08_0001	Plasmodium exported protein, unknown function	-0.71	0.0020	0.0000	0.0030	0.0145	0.0080	1.9	2584	1102	4935	2209	-960
899	MAL7P1.67	conserved Plasmodium protein, unknown function	-0.54	0.0040	0.0003	0.0064	0.0219	0.0093	2.1	1035	741	2149	1336	-963
640	PFL2120w	conserved Plasmodium protein, unknown function	-0.60	0.0020	0.0000	0.0030	0.0145	0.0080	1.3	4429	1177	5910	1280	-975
1142	PFI1275w	protein kinase, putative	-0.48	0.0120	0.0050	0.0175	0.0456	0.0185	1.4	2236	840	3133	1041	-984
1177	PF11_0370	3-oxo-5-alpha-steroid 4-dehydrogenase, putative	-0.47	0.0080	0.0024	0.0122	0.0345	0.0142	1.2	5892	867	6776	1003	-986
789	PFE0290c	conserved Plasmodium protein, unknown function	-0.56	0.0040	0.0003	0.0064	0.0219	0.0093	1.7	1783	908	3058	1358	-991
1421	PFC0335c	conserved Plasmodium protein, unknown function	-0.43	0.0120	0.0050	0.0175	0.0456	0.0185	1.4	1800	609	2539	1121	-992
1137	PF10_0159	glycophorin binding protein	-0.48	0.0120	0.0050	0.0175	0.0456	0.0185	1.1	6767	644	7679	1267	-999
946	MAL7P1.142	conserved Plasmodium membrane protein, unknown fun	-0.53	0.0100	0.0036	0.0149	0.0399	0.0162	1.3	3890	1057	5001	1057	-1003
1383	PF10_0380	serine/threonine protein kinase, FIKK family	-0.43	0.0120	0.0050	0.0175	0.0456	0.0185	1.1	7273	1026	8043	747	-1004
600	PFD0565c	DEAD box ATP-dependent RNA helicase, putative	-0.61	0.0020	0.0000	0.0030	0.0145	0.0080	1.4	4549	1181	6154	1429	-1005
862	PF10_0144	prohibitin, putative	-0.54	0.0020	0.0000	0.0030	0.0145	0.0080	1.6	2079	824	3287	1395	-1011
917	PF11_0327	oxysterol-binding protein-related protein 2	-0.53	0.0020	0.0000	0.0030	0.0145	0.0080	1.4	2670	787	3821	1378	-1014
638	PFB0930w	Plasmodium exported protein (hyp9), unknown function	-0.60	0.0040	0.0003	0.0064	0.0219	0.0093	1.3	5807	985	7376	1614	-1030

639	PF11_0013	stevor, pseudogene	-0.60	0.0080	0.0024	0.0122	0.0345	0.0142	1.4	3885	1536	5475	1100	-1045
1168	PFD0462w	heat shock protein 40	-0.47	0.0040	0.0003	0.0064	0.0219	0.0093	1.2	5133	1072	6074	916	-1047
988	MAL13P1.15	conserved Plasmodium protein, unknown function	-0.52	0.0040	0.0003	0.0064	0.0219	0.0093	1.2	4576	1081	5694	1084	-1048
874	PFD0540c	conserved Plasmodium protein, unknown function	-0.54	0.0080	0.0024	0.0122	0.0345	0.0142	1.4	2859	1055	4121	1269	-1062
681	MAL13P1.90	conserved Plasmodium protein, unknown function	-0.59	0.0020	0.0000	0.0030	0.0145	0.0080	1.3	4991	1305	6549	1327	-1074
1074	PF14_0650	conserved protein, unknown function	-0.49	0.0020	0.0000	0.0030	0.0145	0.0080	1.9	1125	528	2174	1607	-1085
1121	PFF0770c	conserved Plasmodium protein, unknown function	-0.48	0.0060	0.0012	0.0094	0.0286	0.0120	1.5	2109	452	3136	1682	-1107
986	PF10_0214	RNA binding protein, putative	-0.52	0.0080	0.0024	0.0122	0.0345	0.0142	2.1	1126	1024	2312	1272	-1110
522	PF14_0544	conserved Plasmodium protein, unknown function	-0.64	0.0020	0.0000	0.0030	0.0145	0.0080	2.8	1125	994	3105	2101	-1115
467	PFD0310w	sexual stage-specific protein precursor	-0.66	0.0020	0.0000	0.0030	0.0145	0.0080	1.4	5663	1606	7805	1657	-1122
631	PFD0890w	conserved Plasmodium protein, unknown function	-0.61	0.0060	0.0012	0.0094	0.0286	0.0120	3.2	812	1144	2566	1750	-1140
845	PFL0105w	conserved Plasmodium protein, unknown function	-0.55	0.0040	0.0003	0.0064	0.0219	0.0093	1.3	4564	1491	5970	1072	-1158
1161	PFE0060w	parasite-infected erythrocyte surface protein	-0.47	0.0080	0.0024	0.0122	0.0345	0.0142	1.2	5964	671	7012	1539	-1162
598	PFI0495w	conserved Plasmodium protein, unknown function	-0.62	0.0060	0.0012	0.0094	0.0286	0.0120	1.6	3295	1455	5191	1624	-1182
883	PF11_0284	methyltransferase, putative	-0.54	0.0100	0.0036	0.0149	0.0399	0.0162	1.4	3164	1340	4562	1243	-1185
675	PF10_0350	probable protein, unknown function	-0.59	0.0020	0.0000	0.0030	0.0145	0.0080	1.3	5207	816	6962	2144	-1205
418	PFD0225w	conserved Plasmodium membrane protein, unknown fun	-0.67	0.0020	0.0000	0.0030	0.0145	0.0080	21.5	122	393	2610	3304	-1208
1033	PFC0595c	serine/threonine protein phosphatase, putative	-0.50	0.0080	0.0024	0.0122	0.0345	0.0142	1.2	5007	1224	6235	1223	-1219
551	MAL13P1.58	Plasmodium exported protein (PHISTa-like), unknown fun	-0.63	0.0020	0.0000	0.0030	0.0145	0.0080	1.5	3968	1744	6056	1575	-1231
1299	PFL1845c	calcyclin binding protein, putative	-0.45	0.0120	0.0050	0.0175	0.0456	0.0185	1.2	4579	1039	5578	1193	-1232
813	PF14_0741	hypothetical protein	-0.56	0.0040	0.0003	0.0064	0.0219	0.0093	1.3	5119	1199	6675	1595	-1238
1271	PF14_0126	AAA family ATPase, putative	-0.45	0.0040	0.0003	0.0064	0.0219	0.0093	1.2	4842	831	5876	1450	-1246
684	PFC1025w	conserved Plasmodium protein, unknown function	-0.59	0.0020	0.0000	0.0030	0.0145	0.0080	1.5	3661	1146	5469	1911	-1250
932	PFE1245w	conserved Plasmodium protein, unknown function	-0.53	0.0100	0.0036	0.0149	0.0399	0.0162	1.4	3478	993	4901	1704	-1274
808	MAL7P1.174	Plasmodium exported protein (PHISTb), unknown functio	-0.56	0.0060	0.0012	0.0094	0.0286	0.0120	1.2	6599	1716	8212	1177	-1281
1068	PF14_0447	glutaminyl-peptide cyclotransferase, putative	-0.49	0.0080	0.0024	0.0122	0.0345	0.0142	1.4	3574	903	4838	1663	-1302
1098	PFE1355c	ubiquitin carboxyl-terminal hydrolase, putative	-0.49	0.0080	0.0024	0.0122	0.0345	0.0142	1.5	2425	1255	3662	1291	-1309
513	PF11_0091	transcription factor with AP2 domain(s), putative	-0.64	0.0020	0.0000	0.0030	0.0145	0.0080	1.4	5356	1516	7731	2181	-1321
1078	PFD0070c		-0.49	0.0100	0.0036	0.0149	0.0399	0.0162	1.3	5154	2013	6471	672	-1368
1011	PFL0280c	conserved Plasmodium protein, unknown function	-0.51	0.0060	0.0012	0.0094	0.0286	0.0120	1.8	1793	976	3232	1857	-1394
786	PF10_0257	conserved Plasmodium protein, unknown function	-0.56	0.0060	0.0012	0.0094	0.0286	0.0120	3.3	813	963	2675	2338	-1439
1041	PFD0405c	zinc finger, RAN binding protein, putative	-0.50	0.0080	0.0024	0.0122	0.0345	0.0142	1.4	3373	1343	4858	1633	-1491
384	PF14_0731		-0.69	0.0020	0.0000	0.0030	0.0145	0.0080	1.8	4003	2141	7294	2646	-1496
1111	PF14_0225	conserved Plasmodium membrane protein, unknown fun	-0.48	0.0120	0.0050	0.0175	0.0456	0.0185	1.4	3705	914	5104	1981	-1497
984	PF08_0060	asparagine-rich antigen	-0.52	0.0080	0.0024	0.0122	0.0345	0.0142	1.3	6131	1373	7742	1744	-1506
1281	PF10_0212		-0.45	0.0120	0.0050	0.0175	0.0456	0.0185	1.4	3163	1325	4417	1447	-1518
1300	PF10_0047	RNA binding protein, putative	-0.45	0.0080	0.0024	0.0122	0.0345	0.0142	1.8	1480	861	2715	1900	-1525
1316	PF08_0032	DnaJ protein, putative	-0.45	0.0120	0.0050	0.0175	0.0456	0.0185	1.3	4108	1075	5349	1710	-1544
730	PF11_0037	Plasmodium exported protein (PHISTb), unknown functio	-0.58	0.0040	0.0003	0.0064	0.0219	0.0093	2.0	2047	1001	4178	2682	-1553
1423	MAL13P1.14	conserved Plasmodium protein, unknown function	-0.43	0.0100	0.0036	0.0149	0.0399	0.0162	1.3	3460	1361	4654	1437	-1605
1258	PFF0040c	rifin, pseudogene	-0.46	0.0120	0.0050	0.0175	0.0456	0.0185	1.9	1479	1124	2829	1840	-1614
