# Supplementary material for: Transcriptional Profiling of Plasmodium falciparum Parasites from Patients with Severe Malaria Identifies Distinct Low vs. High Parasitemic Clusters
Source: PLoS One. 2012 Jul 18;7(7):e40739. doi: 10.1371/journal.pone.0040739 (PMC3399889; doi:10.1371/journal.pone.0040739)
Supplement: Table S4 — Clinically relevant variables compared between the Malawi samples for which there was no projection of yeast experiments (No Projection) compared with the Malawi Cluster B samples for which there is projection (Projection) demonstrates that temperature and parasitemia remain significant after correction by multivariate logistic regression. (PDF) [file pone.0040739.s009.pdf]

Supplemental Table 4: Clinically relevant variables compared between the Malawi samples for which there was no projection of yeast experiments (No Projection) compared with the Malawi Cluster B samples for which there is projection (Projection) demonstrates that temperature and parasitemia remain significant after correction by multivariate logistic regression.

	No Projection	Projection	P-value*	OR	P-value**
Univariate Regression					
**Temperature (°C)**	39.3 ± 0.9	38.7 ± 1.3	**0.0369**	1.7	0.0790
**Parasitemia (p x 10^3/uL)**	13.1 ± 0.6	10.6 ± 1.5	**0.0000**	7.2	**0.0000**
**Hematocrit (%)**	25 ± 7.3	21.4 ± 7.3	**0.0381**	1.1	0.0820
**WBC count (c x10^3/uL)**	8 [5.9 - 10.3]	12.3 [7.5 - 19.3]	**0.0419**	1.0	**0.0500**
**Gametocyte (% Positive)**	7%	39%	**0.0240**	0.1	0.0520
Multivariate Regression***					
**Temperature**				4.11 [1.27 - 13.36]	**0.0190**
**Parasitemia**				11.03 [2.78 - 43.7]	**0.0100**
			**AUC**	**0.9576**	

*P-value is for T-test of means (normal data), Wilcoxon rank sum (non-normal data), or Fishers's Exact (categorical data)
**P-value of univariate logistic regression for outcome "No Projection" = 1
*** Multivariate logistic regression for outcome "No Projection" = 1 using manual backward selection (by P-value)
