# Supplementary material for: Transcriptional Profiling of Plasmodium falciparum Parasites from Patients with Severe Malaria Identifies Distinct Low vs. High Parasitemic Clusters
Source: PLoS One. 2012 Jul 18;7(7):e40739. doi: 10.1371/journal.pone.0040739 (PMC3399889; doi:10.1371/journal.pone.0040739)
Supplement: Table S5 — After supervised clustering of samples which only have positive signs of malaria retinopathy, two resultant clusters emerged (I and II) for which clinical variables were compared. The retinopathy negative patients are shown for reference. The p-values are for the difference between Retinopathy I and II. The variables values shown in bold text were significantly different (less than 0.05) from the retinopathy negative group. (PDF) [file pone.0040739.s010.pdf]

Supplemental Table 5: After supervised clustering of samples which only have positive signs of malaria retinopathy, two resultant clusters emerged (I and II) for which clinical variables were compared. The retinopathy negative patients are shown for reference. The p-values are for the difference between Retinopathy I and II. The variables values shown in bold text were significantly different (less than 0.05) from the retinopathy negative group.

Variable	Retinopathy + (I)	Retinopathy + (II)	Retinopathy - (A & B)	p-value
Parasitemia (x 10^3 p/ul)	26.4 [12.7 - 39.4]	361.4 [137.3 - 556.9]	84.4 [43.1 - 337.1]	<0.001
WBC (x 10^3 cells/mL)	16.5 ± 17.2	10.7 ± 5.6	12.6 ± 7.0	0.071
Mortality (% Died)	29%	13%	22%	0.189
Lactate (g/dL)	7.6 ± 5.2	6.4 ± 4.1	7.2 ± 3.7	0.211
History of Antimalarials (% Yes)	53%	68%	56%	0.261
Hemaglobin (g/dL)	**7.0 ± 1.9**	**7.3 ± 2.1**	9.0 ± 3.0	0.343
Hematocrit (%)	**20.2 ± 5.8**	**21.0 ± 6.4**	26.9 ± 8.6	0.348
Temp (^{o}C)	38.9 ± 0.9	38.8 ± 1.5	38.9 ± 1.1	0.389
Platelets (x 10^3 cells/mL)	**70 [38.5 - 116]**	**59 [40 - 88]**	121 [31.5 - 264.5]	0.400
Glucose (g/dL)	**6.6 ± 2.8**	**6.4 ± 2.5**	8.7 ± 4.1	0.414
Seisure Duration (Hours)	7 [3 - 18]	6 [3-10]	5 [3 - 6]	0.432
HIV (% Positive)	**18%**	**11%**	0%	0.444
History of LA (% Yes)	**29%**	**23%**	17%	0.456
Fever Duration	52 ± 25	53 ± 23	50 ± 28	0.460
Age (Months)	50 ± 29	38 ± 17	47.5 ± 23.2	0.568
Gender (% Male)	**47%**	**43%**	61%	0.569
Bednet (% used)	**71%**	**73%**	44%	0.581
